# The disability-adjusted life years attributable to mental disorders and self-harm in China from 1990–2021: Findings from the global burden of disease study 2021

**DOI:** 10.1371/journal.pmen.0000146

**Published:** 2025-04-09

**Authors:** Shuang Hu, Ivo Mueller, Hao Yao, Yang Wang, Liming Pan, Xinyi Cao, Pengpeng Ye, Wenyi Jin, Queran Lin

**Affiliations:** 1 Shanghai Mental Health Center, Shanghai Jiao Tong University School of Medicine, Shanghai, China; 2 School of Global Health, Chinese Centre for Tropical Diseases Research, Shanghai Jiao Tong University School of Medicine, Shanghai, China; 3 Population Health and Immunity, The Walter and Eliza Hall Institute of Medical Research, Parkville, Australia; 4 Department of Medical Biology, The University of Melbourne, Parkville, Australia; 5 School of Cyber Science and Technology, University of Science and Technology of China, Hefei, China; 6 The George Institute for Global Health, University of New South Wales, Sydney, Australia; 7 National Centre for Non-Communicable Disease Control and Prevention, Chinese Centre for Disease Control and Prevention, Beijing, China; 8 Department of Orthopaedics, Renmin Hospital of Wuhan University, Wuhan, China; 9 Department of Biomedical Sciences, City University of Hong Kong, Kowloon, Hong Kong SAR China; 10 WHO Collaborating Centre for Public Health Education and Training, Department of Primary Care and Public Health, School of Public Health, Faculty of Medicine, Imperial College London, London, United Kingdom; University of Milano–Bicocca: Universita degli Studi di Milano-Bicocca, ITALY

## Abstract

Over the past three decades, China’s rapid economic development, social transformation, and the COVID-19 pandemic, have reshaped the landscape of mental disorders and self-harm burden. The raw data were sourced from the Global Burden of Diseases, Injuries, and Risk Factors Study 2021. Our study presented the burden by disability-adjusted life-years (DALY) of mental disorders and self-harm at both national and provincial levels in China from 1990 to 2021, by age and sex. We also analysed the association between economic situation and disease burden by Pearson’s correlation. While the overall age-standardised DALY rates of mental disorders exhibited minimal change (-0.3%) over this period, that of self-harm declined dramatically (-69.8%). From 1990 to 2019, both depressive and anxiety disorders saw significant reductions, particularly among working-age adults. However, depression disorders increased significantly among the elderly. The pandemic reversed the gains in anxiety disorders burden, especially in young adults, but only transiently increased depressive disorders burden. With increasing economic development, eating disorders burden rose substantially (+70.4%). These findings underscore the necessity for tailored interventions targeting high-risk groups, including addressing depression among the elderly, anxiety in young adults following the pandemic, and eating disorders in economically prosperous regions.

## Introduction

Mental disorders are increasingly recognised as a leading contributor to the global burden of disease [1]. A nationwide cross-sectional epidemiological survey in China in 2019 reported a lifetime prevalence of mental disorders of 16.6%, CI_95_ [13·0, 20·2] [2]. Over the past three decades, China has experienced dramatic economic development and social transformations. Meanwhile, the population has faced various challenges, like prolonged life expectancy, insufficient care for the elderly, and an increasingly competitive work environment, all of which have contributed to the rise in mental health disorders [3]. The emergence of the COVID-19 pandemic at the end of 2019 has further complicated the mental health landscape, raising significant concerns about its psychological impact as well as the long-term economic and social consequences [4]. The pandemic affected many determinants of mental health, including lockdowns, social restrictions, regular school and work closures, and reduced economic activities [5]. An in-depth understanding of the temporal trends in various mental disorders and self-harm burden in China, especially at the province levels, is vital for strengthening and improving its mental healthcare system.

The Global Burden of Diseases, Injuries, and Risk Factors (GBD) studies are global, population-based epidemiological modeling efforts. They integrate data from diverse sources, adjust for missing or inconsistent information, and employ standardised metrics to facilitate cross-regional and temporal comparisons. GBD 2021 encompasses the measurements of 12 mental disorders and self-harm, facilitating a systematic comparison of diseases and injuries burdens across populations. The GBD 2021 incorporates new data and methodological improvements to previous editions and employs the disability-adjusted life-years (DALY) to measure the discrepancy between the actual population health and a normative standard life expectancy during full health. Based on a recent global systematic analysis of GBD 2021, age-standardised DALY rates increased most substantially for anxiety disorders (16·7%, CI_95_[14·0, 19·8]), followed by depressive disorders (16·4%, CI_95_[11·9, 21·3]) from 2010 to 2021, among the 25 leading level 3 causes of diseases and injuries [6]. Another review, also on GBD 2020 [5], indicated that the global cases of major depressive disorder increased by 27·6% due to the COVID-19 pandemic, while those of anxiety disorders increased by 25·6% [5], underscoring the tremendous burden the pandemic has imposed on mental health.

In China, mental disorders constitute a substantial societal burden, accounting for 5.31% of total DALYs in 2019 [7]. This high burden presents considerate challenges to the Chinese health system. To date, four analyses have been conducted to investigate the burden of mental disorders in China, based on GBD studies from 2013 [8] and 2019 [7,9,10]. However, a comprehensive review assessing the impact of the COVID-19 pandemic on mental health burden in China remains absent. Additionally, no existing review compares the temporal shifts in the burden of mental disorders and self-harm on the same scale. Therefore, it is crucial to undertake a thorough, in-depth analysis of the mental disorders burden since the pandemic, alongside an assessment of long-term trends. Although mental disorders are often linked to high rates of self-harm, including suicide, the GBD analyses calculated the fatal burden only for eating disorders among all mental disorders, as each death can only be allocated to one underlying cause [1]. Therefore, it is crucial to assess trends in the burden of mental disorders and self-harm in parallel.

In the present study, we aimed to investigate the burden estimates (DALY) from GBD 2021 in China for 12 mental disorders and self-harm, males and females, 23 age groups, and 34 provincial administrative units from 1990 and 2021 and provide scientific insights that can inform the development of tailored interventions and policies aimed at vulnerable populations affected by specific mental disorders, as well as for optimising resource allocation during the post-pandemic era in China.

## Methodology

### Overview of burden of disease estimation

The GBD 2021 study is a global, population-based, epidemiological modeling study. Full methods of GBD 2021, including data retrieving, data quality and comparability enhancement, statistical modelling and metrics, have been reported elsewhere [6,11]. In summary, systematic reviews of community representative epidemiological studies were conducted. The inclusion criteria for the GBD 2021 study are: 1) conducted from 1980 onward; 2) based on DSM or ICD diagnostic systems; 3) sufficient information on study methods and sample characteristics for quality assessment; and 4) study samples representative of the general population. Then, adjusted estimates were modelled using DisMod-MR 2.1, a Bayesian meta-regression instrument. DisMod-MR 2.1 pools data from different sources and produces internally consistent estimates of incidence, point prevalence (referred to as prevalence), mortality, years lived with disability (YLDs), years of life lost (YLLs), and DALYs, of 371 diseases and injuries, for males and females, 25 age groups (from birth to age 95 years and older) [6]. Initial estimates are made at the global level then sequentially revised down to the national and subnational levels using data that are progressively more detailed with respect to geography and time. Years lived with disability (YLDs) were calculated by multiplying DisMod-MR prevalence by the corresponding disability weights, derived from community-based surveys and open-web-based surveys [1,6]. Further details regarding the disability weights used in GBD 2021 have been described in previous literature [1,6]. YLLs were calculated by multiplying the cause-specific deaths at a particular age by the standard life expectancy. Normative life expectancy values in GBD 2021 have been published elsewhere [11,12]. DALYs represent a summary measure of population health loss, combining YLLs and YLDs [6].

There are 34 provincial administrative units in China, including 31 mainland provinces, autonomous regions, municipalities, two special administrative regions of Hong Kong and Macao, and Taiwan [12]. In GBD, the overall China estimates include data from 31 mainland provinces, Hong Kong, and Macao. Burden estimation methods have been explained comprehensively in the previous literature [6,11]. In conducting our analysis, we adhered to the Guidelines for Accurate and Transparent Health Estimates Reporting (S1 GATHER Checklist).

### Case definitions

The GBD 2021 study employs a four-level cause hierarchy, where each cause within a level is mutually exclusive, and comorbidity was adjusted during estimation. The mental disorders included in GBD 2021 are depressive disorders (major depressive disorder and dysthymia), anxiety disorders (a combined estimate of all subtypes), bipolar disorder, schizophrenia, autism spectrum disorders (ASD), conduct disorder, attention-deficit/hyperactivity disorder (ADHD), eating disorders (anorexia nervosa and bulimia nervosa), idiopathic developmental intellectual disability (IDID), and a residual category of other mental disorders (including personality disorders). Case definitions are aligned with diagnostic criteria from the DSM (DSM-III, DSM-III-R, DSM-IV, DSM-IV-TR, DSM-5, and DSM-5-TR) or ICD (ICD-9, ICD-10, and ICD-11). Self-harm, defined broadly as an intentional act of causing harm to oneself, ranging from non-suicidal self-harm to attempted suicide to suicide. It is classified as a level 3 cause, including two sub-categories: self-harm by firearm and self-harm by other specified means (for detailed definitions of mental disorders and self-harm see S1 Data).

### Data sources

GBD 2021 study exclusively utilises secondary data from established sources. Mortality data for mental disorders and self-harm in GBD 2021 China were mainly sourced from official records, including the Chinese National Disease Surveillance Points system, China Mortality Registration and Reporting System, Hong Kong Vital Registration - Deaths, Macau Vital Registration - Deaths, Taiwan Vital Registration - Deaths, and national surveys. Non-fatal data were obtained from population-based surveys, studies, and reports. References meeting quality control criteria were included in the model estimation (S2 Data). Among all mental disorders, only anorexia nervosa of eating disorders has associated mortality data. Further details about data sources can be found in previous papers published [11,12] and S2 Data. Regional Gross Domestic Product (GDP) per capita data were retrieved from the World Bank and National Bureau Statistics of China. Flow diagrams of YLLs and/or YLDs estimates for each mental disorders and self-harm are provided in S1 Data.

### Statistics analysis

We analysed the DALY numbers and age-standardised DALY rates (per 100,000 individuals) to quantify the burden of mental disorders and self-harm in China. The analysis was stratified by sex, 20 age groups (age 0–95 years and older), and 34 provincial administrative units. We evaluated the burden in 2021 and examined trends from 1990 to 2021. All estimates were obtained from the DisMod-MR 2.1 Bayesian meta-regression models and presented with 95% uncertainty intervals (UI_95_). To explore the relationship between economic development and disease burden, we employed Pearson’s correlation to assess the association between regional GDP per capita in 2021 or the fold increase of GDP per capita (1990-2021) and age-standardised DALY rates in 2021 or age-standardised DALY rates change (1990-2021). We used StataSE 15 (StataCorp, College Station, TX, USA) for these analyses. For data visualisation, we utilised the R package and GraphPad Prism 9 (GraphPad Software, San Diego, CA, USA).

## Results

### Patterns of different mental disorders and self-harm in China in 2021

In 2021, mental disorders accounted for 23,208,948 DALYs and 23,207,415 YLDs, corresponding to 5.76% and 14.98% of total DALYs and YLDs in China, respectively, whereas self-harm accounted for 4,375,868 (1.09%) DALYs and 138,543 (0.09%) of YLDs. These burdens are relatively low compared to other countries with China ranked 34 out of 204 countries for mental health (1,631.3, Global DALY rates: median 2,006.6, range [1,315.3, 3050.4]) and 88 for self-harm (307.6, Global: median 348.6, range [47.2, 2,858.5]) DALYs per 100,000 population, respectively.

**Fig 1:**
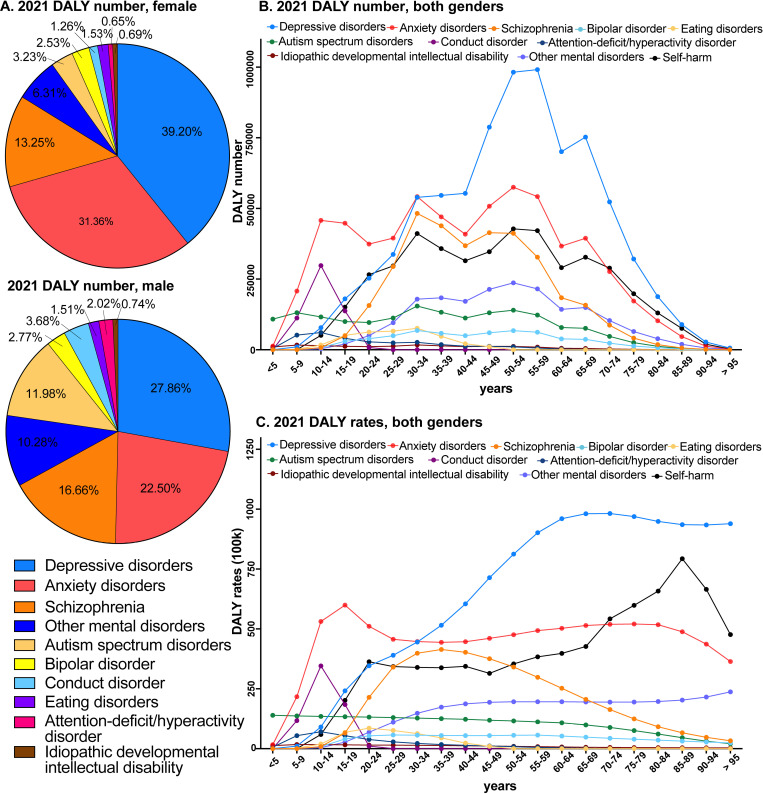
Sex and age specific disability-adjusted life years (DALY) of mental disorders and self-harm in 2021 in China. (A) Proportions of DALY numbers explained by each mental disorders for male and female. (B) DALY numbers for each mental disorder and self-harm, by age. (C) DALY rates per 100,000 individuals for each mental disorder and self-harm, by age.

Among all mental disorders, depressive disorders (total DALYs: 7,865,943; percentage of both genders: 33.9%, of female: 39.2%, of male: 27.9%), anxiety disorders (6,314,450, 27.2%, 31.4%, 22.5%) and schizophrenia (3,445,845, 14.8%, 13.3%, 16.7%) accounted for 75.9% of the total DALYs of all mental disorders, followed by ASD, bipolar disorder, conduct disorder, eating disorders, ADHD and IDID (Fig 1A, B, Table 1). The age-standardised DALY rates of anxiety disorders and depressive disorders were significantly higher in females compared to males, whereas the burden of ASD, ADHD and conduct disorders were relatively higher in males than females (Table 1).

**Table 1:** Changing DALYs of all mental disorders and self-harm in China 1990-2021.

	DALY numbers [95% UI]			Age-standardised DALY rates [95% UI]		
	2021	1990 vs. 2021	2019 vs. 2021	2021	1990 vs. 2021	2019 vs. 2021
*Self-harm*						
male	2647375[2067912, 3422302]	-45.8%[-60.3, -13.6]	0.6%[-25.8, 36.9]	316.2[248.9, 406.1]	-61.7%[-71.8, -39.4]	-0.8%[-25.9, 33.4]
female	1728492[1338614, 2344272]	-67.1%[-76.7, -48.2]	1.5%[-26.1, 43.4]	203.5[157.3, 277.4]	-77.5%[-84.1, -64]	-0.3%[-26.9, 40.4]
both	4375868[3589786, 5401700]	-56.8%[-66.2, -33.9]	0.9%[-18.5, 25.1]	260.0[213.5, 320.2]	-69.8%[-76.3, -52.8]	-0.5%[-19.2, 22.5]
*Mental disorders*						
Male	10868647[8338349, 13586827]	34.6%[29.9, 39.5]	4.2%[2, 6.4]	1355.6[1040.2, 1705.9]	1.8%[-0.6, 4.1]	2.9%[0.7, 5.2]
Female	12340302[9266536, 15879288]	36.7%[30, 43.6]	5.6%[3.1, 8.6]	1537.4[1153.4, 1969.9]	-2.3%[-5.2, 0.8]	4.2%[1.5, 7.3]
Both	23208948[17695799, 29428287]	35.7%[30.2, 41.6]	4.9%[2.7, 7.3]	1446.8[1102.9, 1826.7]	-0.3%[-3.1, 2.4]	3.5%[1.3, 6]
*Depressive disorders*						
Male	3028508[2136876, 4116004]	51.9%[40.7, 65]	4.6%[0.4, 9]	330.6[233.4, 450.4]	-3.8%[-8.3, 0.9]	2%[-2, 6.5]
Female	4837434[3418220, 6553138]	40.9%[29.1, 54.2]	1.5%[-2.7, 6]	533.0[378.2, 723.9]	-12.4%[-17, -7.7]	-1%[-5, 3.2]
Both	7865943[5560591, 10696833]	44.9%[33.4, 57.7]	2.7%[-1.3, 6.6]	430.6[305.2, 586.2]	-9%[-13.4, -4.7]	0.1%[-3.7, 3.9]
*Anxiety disorders*						
Male	2445121[1698437, 3323374]	26.4%[15.8, 38.4]	10.3%[3.4, 17.9]	318.8[219.0, 438.6]	1.4%[-4.6, 8]	8.9%[1.8, 16.4]
Female	3869329[2705314, 5246192]	30.6%[19, 44.1]	16.4%[9.1, 24.3]	525.6[367.2, 715.9]	3.4%[-2.5, 9.7]	13.4%[5.6, 21.7]
Both	6314450[4367592, 8566618]	29%[17.9, 41.9]	14%[6.9, 21.2]	418.9[291.5, 573.2]	2.4%[-3.6, 8.6]	11.7%[4.3, 19]
*Schizophrenia*						
Male	1810465[1348131, 2267721]	46%[39.3, 53.3]	1.3%[-1.2, 3.8]	209.4[156.6, 262.3]	3.7%[0.8, 6.3]	1.4%[-1.2, 3.8]
Female	1635380[1217248, 2050005]	50.1%[42.3, 57.8]	1.1%[-1.3, 3.5]	198.2[148.0, 249.2]	4.9%[2.2, 7.7]	1.8%[-0.7, 4.2]
Both	3445845[2572495, 4306768]	47.9%[41, 55]	1.2%[-0.5, 2.8]	203.9[152.5, 255.7]	4.2%[2, 6.2]	1.6%[-0.2, 3.1]
*Bipolar disorder*						
Male	301516[196658, 427893]	33.4%[24.5, 44]	0.2%[-2.8, 3.9]	36.4[23.8, 52.4]	-0.2%[-3.4, 3.4]	-0.2%[-3.5, 3.7]
Female	312223[202969, 444083]	36.1%[26.5, 47.5]	-0.2%[-3.2, 3.2]	39.0[25.5, 56.3]	-0.1%[-3.1, 3.5]	-0.2%[-3.3, 3.4]
Both	613740[399627, 869324]	34.8%[26.3, 46]	0%[-2.3, 2.5]	37.7[24.6, 54.3]	0%[-2.5, 2.4]	-0.2%[-2.6, 2.3]
*Eating disorders*						
Male	164511[96765, 265351]	50.8%[36.3, 68.6]	3%[-1.7, 7.6]	26.3[15.5, 42.2]	77.5%[65.5, 90.1]	5.7%[0.8, 10.6]
Female	188259[116837, 298006]	36.5%[24.7, 48.2]	0.3%[-3.4, 3.7]	33.2[20.3, 52.0]	65.5%[55.8, 76]	4.4%[0.5, 8]
Both	352770[214581, 558323]	42.9%[31.1, 55.4]	1.5%[-1.3, 4.8]	29.6[17.9, 47.0]	70.4%[62.6, 78.8]	5%[1.7, 8.2]
*Autism spectrum disorders*						
Male	1301567[884402, 1838596]	24.1%[17.8, 30]	0.7%[-1.9, 3.4]	183.4[124.7, 258.8]	8.6%[3.4, 13.5]	0.4%[-2.4, 3.2]
Female	399172[265348, 566943]	16.1%[10.8, 21]	0.3%[-2.2, 3.1]	60.0[39.9, 85.3]	1.4%[-3.1, 5.6]	0.3%[-2.2, 3]
Both	1700739[1146590, 2397647]	22.1%[17.1, 26.9]	0.6%[-1.4, 2.6]	124.2[83.7, 175.1]	7.2%[3.1, 11.5]	0.5%[-1.5, 2.6]
*Attention-deficit and hyperactivity disorder*						
Male	219407[124741, 348445]	-3.7%[-12.3, 5.5]	3.3%[-5.6, 12.3]	37.3[20.8, 59.2]	8.5%[-0.5, 17.9]	2.2%[-6.7, 11.2]
Female	79705[44360, 129684]	-4.8%[-14, 6.4]	1.9%[-5.9, 10.2]	14.8[8.1, 24.3]	10.4%[0.8, 21.8]	1.1%[-7, 9.5]
Both	299112[169780, 474761]	-4%[-11.3, 4.4]	2.9%[-3.9, 9.6]	26.7[15.0, 42.8]	10.1%[1.7, 18.7]	2%[-5, 8.7]
*Conduct disorder*						
Male	400129[220545, 623278]	-18.2%[-24.1, -11.2]	4.3%[2.2, 6.6]	78.9[43.6, 123.0]	3.2%[-4, 11.6]	0.1%[-2, 2.2]
Female	154921[82175, 253223]	-21.7%[-28.4, -12.9]	5.1%[1.5, 9]	35.0[18.5, 57.0]	5%[-3.9, 16.1]	0.1%[-3.2, 3.7]
Both	555050[306262, 870973]	-19.2%[-24.5, -13.7]	4.5%[2.6, 6.7]	58.5[32.2, 91.7]	5.2%[-1.2, 11.9]	0%[-1.8, 2]
*Idiopathic developmental intellectual disability*						
Male	80495[14591, 171844]	-31.7%[-55.7, -21.5]	-2.6%[-7.9, 1.4]	12.2[2.2, 26.0]	-33.8%[-55.5, -25.2]	-2.4%[-7.8, 1.4]
Female	85312[30003, 158256]	-18%[-31.6, -4.5]	-1.5%[-4.3, 1.6]	13.3[4.6, 24.9]	-24.8%[-37.9, -13.7]	-1.3%[-4.4, 1.5]
Both	165807[45253, 332026]	-25.3%[-40.8, -14.2]	-2%[-4.9, 0.5]	12.8[3.4, 25.8]	-29.5%[-44.6, -20.3]	-1.9%[-4.9, 0.5]
*Other mental disorders*						
Male	1116927[715740, 1697977]	62%[54.4, 70.7]	1.4%[-0.1, 2.9]	122.2[77.8, 186.5]	-0.1%[-1.7, 1.6]	-0.1%[-1.6, 1.5]
Female	778565[499263, 1175264]	73.3%[64.1, 82.9]	1.5%[-0.3, 3.2]	85.2[54.6, 128.7]	0.3%[-1.4, 2]	-0.1%[-1.9, 1.7]
Both	1895492[1220533, 2871836]	66.5%[58.4, 75.4]	1.4%[0.3, 2.7]	104.0[66.8, 158.2]	-0.1%[-1.3, 1.1]	0%[-1.3, 1.2]

The burden of most mental disorders showed a strong but variable age dependency. In 2021, the burden of depressive disorders increased continuously with age: DALY rates were low in children and adolescents, increased through adulthood and peaked in over 55-year-old people (Fig 1C, S1 Table). Rates of anxiety also increased through adult life, but the highest rates were observed in adolescents (10-19 years, Fig 1C, S1 Table). The burden of schizophrenia and bipolar disorder was highest in working-age people, while that of ASD, ADHD, conduct disorder, and IDID were mainly in children and adolescents (Fig 1C, S1 Table).

### Changes in the overall burden of mental disorders and self-harm in China from 1990-2021

In the last 3 decades, China has seen substantial changes in the burden of different mental disorders. Overall, total DALY numbers related to mental disorders increased from 17,105,604 in 1990 to 22,116,775 (+29.3%) in 2019 and 23,208,948 (+35.7%) in 2021, with mental disorders accounting for 4.1% of all DALYs in 1990, 5.6% in 2019 and 5.8% in 2021. There were comparable increases in DALYs both for females (1990 to 2021: +36.7%, UI_95_ [30, 43.6]) and males (+34.6% [29.9, 39.5]), with females consistently experiencing higher burdens of mental disorders (1990: 52.8%, 2019: 52.8%, 2021: 53.1% of the total). Despite increasing total mental disorders burden, dramatic decreases in the burden of self-harm were observed, with total DALYs dropping from 10,136,574 in 1990 to 4,334,847 in 2019 (-57.2%) and 4,375,868 (-56.8%) in 2021 with the proportion of all DALYs due to self-harm dropping from 2.43%, 1.11% and 1.09%. Reduction in self-harm was particularly pronounced for females (1990 to 2021: -67.1% [-76.4, -48.2]) than males (-45.8% [-60.3, -13.6]), with females contributing 51.9% and 39.5% to self-harm burden in 1990 and 2021, respectively. The first two pandemic years, 2020 and 2021, saw a significant increase in the burden of mental disorders (2019 to 2021: +4.9% [2.7, 7.3]), while rates of self-harm changed little (+0.9% [-18.5, 25.1]) with comparable changes for females (mental disorders: +5.6% [3.1, 8.6] and self-harm: +1.5% [-26.1, 43.4]) and males (+4.2% [2, 6.4] and +0.6% [-25.8, 36.9], all in Table 1).

**Fig 2:**
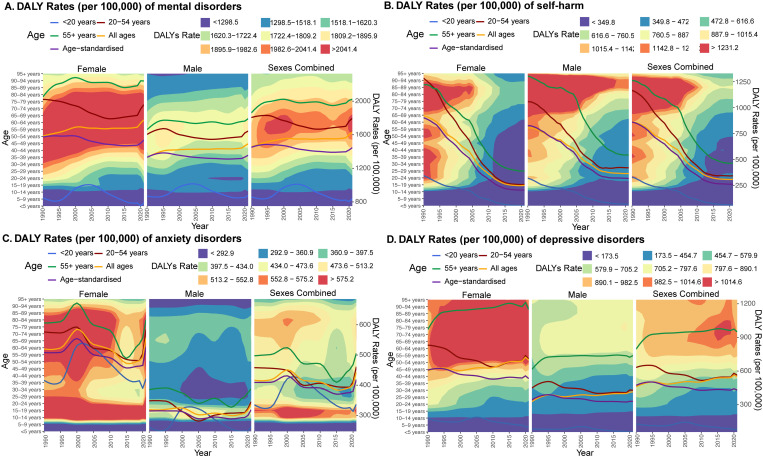
Trends of age-sex-specific DALY rates (per 100,000 individuals) in China, 1990-2021. (A) mental disorders, (B) self-harm, (C) anxiety disorders, and (D) depressive disorders.

Given the significant change in age distributions in China from 1990 to 2021, age-standardised DALY rates more appropriately described these changes. Over this 1990-2019 period, the age-standardised DALY rates (per 100,000) for all mental disorders dropped from 1,451.1 in 1990 to 1,397.3 (-3.7%) in 2019, before rising again to 1,418.4 in 2020 (+1.5%) and 1,446.8 (+3.5%, both compared with 2019) in 2021 (Fig 2A, Table 1). In parallel, rates for self-harm dropped by 860.2 in 1990 to 261.4 (-69.6%) in 2019 and 260 (-69.8%) in 2021 (Fig 2B, Table 1). These changes were not uniform across the age groups (S1 Table): while DALY rates of mental disorders decreased significantly in people aged 20-44 years (1990 to 2021 range: [-8.9% to -3.6%]), they increased in those 50-89 years (range: [4.4% to 10.3%]). Similarly, reductions in self-harm were most substantial in adolescents (10-19 years, range [-80.2% to -78.1%]) and least pronounced for elderly people (70+ years, range [-62.1% to -52.3%]). During the pandemic years, mental disorders burden increased significantly in working-age people (20-64 years, range [3.2% to 8.3%], peak 25-29 years) but not children 5-19 years and those 65 years and above. The rate of self-harm remained relatively unchanged for all age groups during 2019-2021 (S1 Table).

**Fig 3:**
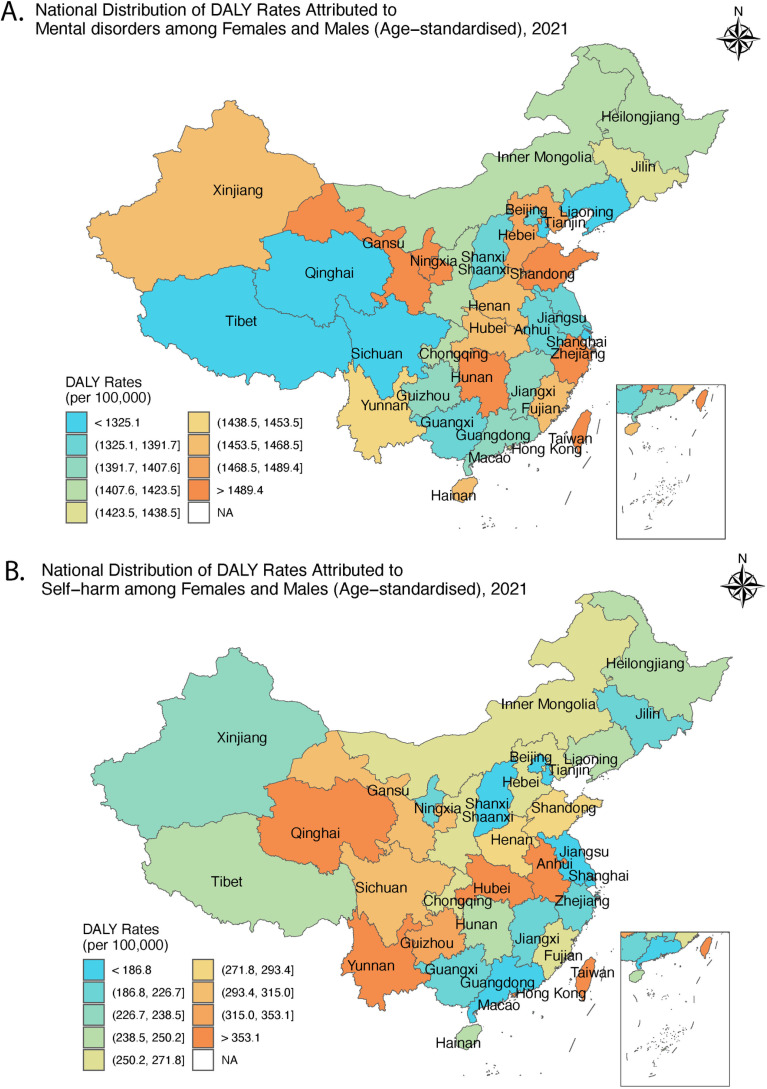
Spatial distribution of age-standardised DALY rates (per 100,000 individuals) in China, 2021. (A) mental disorders and (B) self-harm. The base layer of the map is obtained from Aliyun (http://datav.aliyun.com/portal/school/atlas/area_selecto).

**Fig 4:**
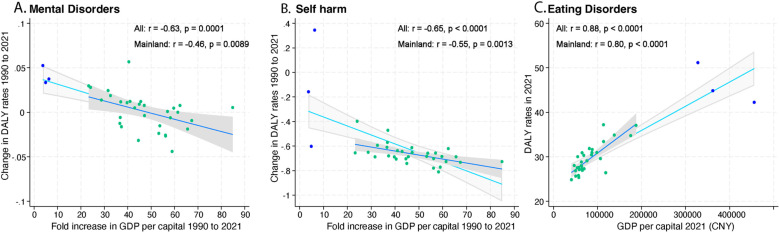
Associations between economic development and DALY rates. (A) Gross Domestic Product (GDP) per capita fold increase (1990-2021) vs. age-standardised DALY rates change (1990-2021) of mental disorders, (B) GDP per capita fold increase (1990-2021) vs. age-standardised DALY rates change (1990-2021) of self-harm, and (C) GDP per capita in 2021 vs. age-standardised DALY rates in 2021 of eating disorders, among 31 mainland provinces (in green spots) or 34 provincial units (including Taiwan, Hong Kong, and Macao, in blue spots). We used Pearson correlation for statistics.

There was also substantial spatial heterogeneity across China in the burden of mental disorders (Fig 3A, S2 Table): In 2021, the highest age-standardised DALY rates of mental disorders were observed in Hunan, Shandong, Gansu and Ningxia, while Qinghai, Liaoning, Shanghai and Tianjin had the lowest burden. The largest decreases in mental health burden from 1990 to 2021 (S2 Table) were seen in Anhui (-4.4% UI_95_ [-12.1, 5.5]), and Tibet (-3.2% [-11.8, 6.7]), while the largest increases in Beijing (+5.7% [-1.7, 14.9]), Hongkong (+5.2% [-3.4, 13.8]), Taiwan (+3.7% [-6, 15.5]) and Macao (+3.3% [-5.9, 14.8]). The burden of mental disorders showed no association with GDP per capita (All: p = 0.9957; Mainland China only: p = 0.1388). However, there was a robust positive correlation between the relative change in mental disorders burden and the fold increase in GDP per capita between 1990 and 2021 (Fig 4A, All: r = -0.63, p = 0.0001; Mainland China only: r = -0.46, p = 0.0089). Similarly, there was substantial spatial heterogeneity in the changes in the burden of mental disorders among different Chinese provinces during the pandemic period: the largest increases in the burden of mental disorders were seen in Hunan (+7.8% [-3.3, 19.5]), Heilongjiang (+7.0% [-2.1, 17.2]), Xinjian (6.6% [-3, 17.6]) and Hainan (6.1% [-3.6, 16.3]), whereas increases were smallest in Zhejiang (-0.3% [-8.8, 10.3]), Qinghai (+0.0% [-7.3, 7.5]) and Tibet (0.8% [-7.9, 11.1]).

Much larger spatial heterogeneity was observed in age-standardised DALY rates of self-harm in 2021 (Fig 3B, S2 Table), with the lowest rates observed in Beijing and Shanghai and the highest rates in Yunnan, Hubei and Taiwan. The most significant reduction in self-harm from 1990 to 2021 was observed in Jiangxi (-81.1%, UI_95_ [-86.1, -66.6]), Hunan (-78.0% [-84.1, -60.8]) and Anhui (-77.2% [-83.0, -61.2]), while more minor reduction was made in Hebei (-47.2% [-63.1, -26.0]), Liaoning (-39.8% [-55.9, -19.2]) and Hong Kong (-15.8% [-43.9, 7.6]). The burden of self-harm increased from 1990 to 2021 in Taiwan (+34.4% [24.2, 42.7]). The degree of reduction in the burden of self-harm was strongly associated with fold increase in GDP per capita (Fig 4B, All: r = -0.65, p < 0.0001; Mainland China only: r = -0.55, p = 0.0013). Self-harm burden remained stable during the pandemic among provinces (S2 Table).

### Change in different mental disorders pre- and during the pandemic

In the pre-pandemic period (1990-2019), the burden of anxiety was consistently much higher in women despite different changes over time between sexes (Fig 2C). In females, anxiety increased strongly from 1995 to 2000 (age-standardised DALY rates: +9.1%, 508.1 to 554.7) with the strongest increases seen in adolescents (+13.3%, 711.9 to 806.5). After the peak in 2000, anxiety decreased in females of all ages until 2015 (-14.1%, 431.1 to 370.1), when it started to increase again in older women only (55+: +9.9%). In adult males (20-54), anxiety decreased from 1990 to 2005 (-19.3%, 348.1 to 280.8), after which it tended to increase modestly (2005-2019: +9.9%). Adolescent males showed a comparable pre-pandemic time trend to female adolescents. During the pandemic, anxiety increased dramatically (age-standardised DALY rates: +11.7% [4.3, 19.0], Table 1), more significant in 2021 (+10.3%) than in 2020 (+1.3%), with the most substantial increases in young adults (25-29 years: +30.4% [18.7, 42.3], S1 Table). However, there were strong differences between provinces, with the strongest increase seen in Heilongjiang (20.2% [-8.2, 53.4]), Hainan (19.1% [-10.6, 52.6]) and Fujian (17.4% [-12.1, 50.4]) and the smallest in Tianjin (0.3% [-21.7, 26.5]), Zhejiang (1.1% [-21.6, 29.3]), and Ningxia (2.4% [-21.4, 33.4]. These pandemic increases brought the national overall anxiety burden back to the levels last seen in the early 1990s (1990: 409.2, 2021: 418.9, +2.4% [-3.6, 8.6]).

The burden of depressive disorders in China was consistently highest in the elderly (55+) and affected females more than males from 1990 to 2021 (Fig 2D). Overall, the age-standardised DALY rates of depressive disorders decreased by 9.0% [-13.4, -4.7]) from 1990 to 2021 (Table 1). Strikingly, DALY rates of depressive disorders increased significantly in elderly Chinese people (55+), while dropped strongly in working-age adults (20-54 years, Fig 2D), with the largest decrease in young adults (20-24 years, S1 Table). There was strong spatial heterogeneity in age-standardised DALY rates of depression in 2021 (S2 Table): the overall highest DALY rates were reported from Hongkong (547.8 [350.1, 776.9]), Zhejiang (518.9 [215.3, 339.9]) and Shandong (494 [330.0, 715.3]) while the lowest in Tianjin (323.2 [215.3, 445.3]), Shanghai (327.3 [222.6, 448.4]) and Beijing (356.7 [235.1, 497.6]). Overall, the burden of depressive disorders did not change during the pandemic years (2019 to 2021: +0.1% [-3.7, 3.9]) and only increased significantly in working-age males (20-54 yrs: +5.1% [0.3, 10.2]). However, there were substantial differences between 2020 (compared to 2019: +2.9%) and 2021 (compared to 2020: -2.7%).

For eating disorders, age-standardised DALY rates increased dramatically between 1990 and 2021 (+70.4% [62.6, 78.8], Table 1). These increases were heterogeneous across China (median +63.3%, range [+15.7% to +86.4%]). Increases were larger for bulimia nervosa (+79.0% [69.1, 91.2]) than for anorexia nervosa (+48.8% [40.1, 57.9]) (S1 Fig). A significantly larger overall increase was noticed in males for bulimia nervosa (1990 to 2021, males: +86.1% [72.6, 102.8] vs. females: +72.3% [59.3,86.3]) but not anorexia nervosa (males: +47.0% [33.4, 61.1] vs. females: +52.3% [41.7, 63.6], S1 Fig). The burden of eating disorders in 2021 was strongly associated with GDP in different regions (Fig 4C, r = 0.88, p < 0.0001), with the highest rates observed in Macao, Hong Kong, Taiwan, Tianjin and Beijing and lowest in Yunnan and Gansu (S2 Table). The pandemic did not impact on the continued growth in eating disorder DALY rates (2019 to 2021: 5% [1.7, 8.2], Table 1, S2 Fig).

In parallel with the improvement of antenatal screening services and improved levels of environmental pollution, China has seen substantial decreases in the overall burden of IDID since 1990 (-29.5% [-44.6, -20.3], S1 Fig). However, these decreases were not uniform (median -26.2%, range [-57.9%, +28.1%]). In 2021, IDID burden in China is very strongly negatively associated with GDP (r = -0.79, p < 0.0001). The pandemic had no impact on the continued decrease in IDID DALY rates (2019 to 2021: -1.9% [-4.9, 0.5], Table 1).

The age-standardised DALY rate from 1990-2021 stayed stable for bipolar disorder (0% [-2.5, 2.4]), increased modestly for schizophrenia (+4.2% [2.0, 6.2]) and for ASD (+7.3% [3.1, 11.5]), and increased larger for ADHD (10.1% [1.7, 18.7], see Table 1 for detailed estimates). The pandemic had no significant impact on change patterns of these disorders.

## Discussion

To the best of our knowledge, the current study presents the first comprehensive report on DALYs for mental disorders and self-harm in China, covering the period from 1990 to 2021, including the first two years affected by the COVID-19 pandemic. Before the pandemic, the total DALYs of mental disorders increased (29.3%) from 1990 to 2019, while the age-standardised DALY rates decreased (-3.7%), consistent with a previous study on GBD 2019 in China [3]. This suggests that the increase in total DALYs is predominantly due to population growth and aging [6,7,13]. The reduction in age-standardised DALYs can largely be attributed to two key factors. First, China’s remarkable economic growth over the past three decades has played a pivotal role. Implementing economic reforms propelled the nation to an average GDP growth rate of 9% annually before the pandemic, positioning China as one of the fastest-growing economies worldwide [14]. Our analysis suggests a strong positive association between the relative reduction in the burden of mental disorders and the GDP growth rate from 1990 to 2021 across provinces. This indicates that regions with more rapid economic expansion experienced a more pronounced decrease in mental disorder burden. Despite some heterogeneity, most studies suggested economic growth is generally associated with improvements in mental health, contrasting with the adverse effects typically seen during economic recessions [15–18]. Second, the Chinese government's increasing emphasis on mental health care has contributed to mitigating the burden of mental disorders. Since 2000, the Chinese government has included mental disorders as one of the priority non-communicable diseases [3]. In 2004, the “686 Program” was launched, aiming to scale up community-based mental health services for people with severe mental disorders [19]. In 2013, China’s Mental Health Law was implemented to further safeguard the rights of people with mental disorders in China [20,21]. In 2019, the government further integrated mental health improvement into the Healthy China Action (2019–2030) initiative, underscoring its commitment to this critical public health issue [22].

Our study confirms the nationwide downward trend in the burden of self-harm, revealing that the age-standardised DALY rates for self-harm in China declined by two-thirds from 1990 to 2021, with females exhibiting an even more pronounced decline by nearly three-quarters during this period [23,24]. A multitude of socioeconomic reasons were related to this trend, including the stringent implementation of nationwide suicide prevention strategies (e.g., the management of lethal pesticides, which have historically been the most common suicide method in China) [25], the rapid urbanisation of Chinese society, which has benefited rural females with more educational and economic opportunities to escape from psychologically oppressive environments [14], and the improvement of mental health services which could mitigate the effect of mental disorders on the risk of self-harm [26]. However, despite this overall decline, the elderly population continues to exhibit the highest DALY rates of self-harm in China in 2021, as well as the smallest reduction in the DALY rates of self-harm from 1990 to 2021. In China, the elderly are particularly susceptible to self-harm [23] and should remain a primary focus of prevention and control measures. Like previous studies [14,24,27], our study also identifies substantial spatial heterogeneity in the burden of self-harm, with the highest burden observed in the central and southwest regions of China. Various socioeconomic, cultural, and geographical factors, including rurality, shifts in family structure, and prevailing attitudes toward life, may underlie the inter-provincial disparity in self-harm burden across China [24].

We observed that the age-specific DALY rates of anxiety disorders in China exhibited a double-peak pattern in 2021, with the highest peak in adolescence and the second in old age. This pattern contrasts with the global trend, which typically shows a single peak that rises from infancy through adolescence, plateaus from early adolescence to middle age, and then declines [28,29]. Due to rapid social changes, Chinese adolescents face various kinds of pressure. One such example is the often-overwhelming academic pressure thanks to the highly competitive nature of the entrance examinations for high schools and universities, which may be an essential stimulus of anxiety in Chinese adolescents. In July 2021, the Chinese government introduced the “Double Reduction” Policy [30], which is said to be the strictest policy on reducing adolescents’ academic pressure in history, to reduce the burden of excessive homework and the burden of off-campus training for Chinese adolescents. Preliminary research showed that the overall anxiety level of Chinese adolescents significantly decreased after this policy [30]. It will be interesting to see whether the peak of the DALY rates of anxiety disorders in adolescence has been flattened since 2021.

Although the overall age-standardised DALY rates for depressive disorders dropped from 1990 to 2019, there was a notable increase in the burden among the elderly (55+), consistent with previous studies in China [31,32]. A similar trend was observed in other middle and low-middle sociodemographic index (SDI) regions, where a decline in depression incidence among younger populations contrasted with an increase among the elderly. Conversely, high SDI regions showed the opposite trend [33,34]. In China, socioeconomic and demographic changes, especially the unprecedented urbanisation experienced over the last three decades, might account for the rising depression burden on the elderly [35,36]. Unlike the Western countryside, elders in rural China traditionally relied heavily on their children, especially for financial support, due to the lack of a social retirement system [37]. The massive migration of young adults to urban areas has forced the left-behind elders in rural places to adapt to an unfamiliar society and lifestyle, exacerbating loneliness and social isolation [35,38,39]. In contrast, working-age individuals have benefitted from economic development, leading to lower levels of poverty and mental disorders, including depression [40]. Similar factors explain the high rates of anxiety in the elderly [41]. Therefore, greater attention is needed to address the mental health needs of China’s elderly population. Further studies into the effect of socio-economic status on mental health outcomes are warranted both in the elderly and general population.

Our study reveals a significant and continuous increase in DALY rates for eating disorders across China from 1990 to 2021, with notable regional disparities. In 2021, DALY rates for eating disorders were closely correlated with regional GDP, with the highest rates observed in economically prosperous areas such as Macao, Hong Kong, Taiwan, Beijing, and Tianjin, and the lowest in less developed areas like Yunnan and Gansu. Globally, the burden of eating disorders is markedly higher in high-income countries compared with low- and middle-income countries [42,43]. Additionally, a rising trend is evident worldwide, particularly in densely populated Asian areas [9,44], which aligns with our findings. Notably, there was a significant increase in bulimia nervosa among males, consistent with a recent review [45]. The rising trend may be linked to enhanced recognition and evolving diagnostic practices in male patients.

The pandemic has been an unprecedented health and social emergency globally since late 2019. Anxiety and depressive disorders were particularly impacted globally by the pandemic, as supported by numerous studies [5,6,46–48]. Notably, the burden of mental disorders, anxiety disorders, and depressive disorders all increased most significantly among young adults during the pandemic [48–51]. Young adults were more likely to experience increased job insecurity and financial strain [49,52], heightened intolerance to uncertainty [51], and greater exposure to negative COVID-19-related content on social media [53]. In China, age-standardised DALY rates for all mental disorders increased by 1.5% in 2020 and by 3.5% in 2021 compared with 2019, reverting the burden to levels not seen since the 1990s, after nearly 30 years of an overall declining trend. However, unlike the global trend, which saw a significant increase in the burden of anxiety disorders as early as 2020 [6], China experienced only a modest rise (+1.3%) in anxiety disorder burden in 2020, with the majority of the increase occurring in 2021 (+10.3%). A systematic review showed that China had one of the lowest increases in anxiety disorder prevalence globally in 2020 [5]. This review also identified a significant association between the rise in anxiety disorder prevalence and decreased human mobility, as well as daily SARS-CoV-2 infection rates [5]. Globally, high daily infectious rates were also associated with increased depression prevalence (regression coefficient 18·1) [5]. Due to proactive control measures, China successfully contained the first large-scale outbreak of COVID-19 within a few months since its outbreak at the beginning of 2020 and maintained daily new cases at a low level nationwide compared with other countries in the remaining months of 2020 and 2021. Additionally, since early 2020, Chinese government also implemented a series of targeted measures to mitigate the potential mental impacts of the pandemic [54,55]. These measures included requiring all local authorities to include psychological crisis interventions into epidemic response plans, developing a four-tiered triage strategy for psychological crisis management, establishing a nation-wide system of online mental health services, and launching 24/7 psychological assistance hotlines across the country [54,55]. The success in controlling the pandemic's spread combined with these effective mental health interventions likely explains the absence of a substantial increase in anxiety disorder burden in China in 2020. Nevertheless, further research is required to explore the reasons behind the significant rise in anxiety disorder burden in 2021 and to understand the subsequent changes. While the depression burden increased substantially from 2019 to 2020 (+2.9%), it declined from 2020 to 2021 (-2.7%). The rapid and effective initial control coupled with the lowest infection rates worldwide throughout 2021 and the ability to resume regular work activities [56], may explain the reduced depression burden in 2021.

Intriguingly, the growth trajectories of depression and anxiety in China diverged, with the depression burden decreasing in 2021 while the anxiety burden increased significantly. The disparity may be attributable to differing methodological approaches in the GBD 2021 models for depressive and anxiety disorders. Specifically, the model for depressive disorders incorporated remission rates derived from five longitudinal studies, whereas the model for anxiety disorders did not include remission rates due to a lack of relevant source literature (S1 Data). This methodological difference might explain the observed inconsistencies in the trends for these mental health conditions.

In addition, our study found that the initial two years of the pandemic did not substantially alter the epidemiological pattern of self-harm in China. Early projections suggested that the mental health consequences of the pandemic might lead to a subsequent suicide epidemic [57,58]. However, subsequent empirical studies did not demonstrate a significant rise in suicide rates worldwide [59,60]. A more recent meta-analysis also indicated that there was no evidence to suggest that suicide rates changed significantly during the pandemic, irrespective of gender, age group, and region [61]. From the outset of the pandemic, China implemented a package of measures to mitigate the mental health consequences of COVID-19, including incorporating psychological crisis interventions into the epidemic response plans of all local authorities and the establishing a nationwide system of online mental health services [54]. Additionally, China has implemented various supportive monetary and fiscal policies to encourage economic recovery from the pandemic [62]. These factors, among others, might explain the lack of a significant effect of COVID-19 on the burden of self-harm in China in 2020 and 2021.

These findings call for the need for an enhanced response to address the mental health ramifications of the pandemic in China, especially among young adults, through improving service access and financial commitment. Burden trends may continue to evolve over extended periods, and the full scale of economic consequences and their effects might emerge much later after the lockdowns lifted at the end of 2022. Currently, insufficient data limits our ability to draw definitive conclusions, and additional data beyond 2021 are warranted to fully understand the evolving trends of mental health in China.

The current study has several limitations. First, the inclusion of studies relying on web-based self-evaluation questionnaires during the pandemic in GBD analysis raises concerns about the reliability of its findings. Many studies conducted during COVID-19, which documented a substantial increase in mental health burden, were cross-sectional and web-based, frequently employing screening tools such as the Patient Health Questionnaire-9. These studies were included in GBD 2021 analyses due to insufficient available data. However, a 2023 global meta-analysis of 134 cohort studies investigating the pandemic's impact found that general mental health and anxiety symptoms remained largely unchanged, while depression symptoms worsened only minimally among general populations [63]. This finding contradicts the global GBD estimates regarding anxiety disorders and depressive disorders [5], as well as our findings, suggesting that the inclusion of these web-based studies might result in an over-estimation of disease burden. High-quality longitudinal studies, with standardised diagnosis process conducted by psychiatrists, are urgently needed to better understand the pandemic's true impact on mental health and resolve these differences.

Secondly, the GBD estimates for burden associated with mental disorders in China were notably lower compared to most other countries [1]. Several cultural and social factors might contribute to this low estimate. China’s mental health system still remains predominantly hospital-based [19]. While the establishment of community and primary mental health services is a long-term goal, progress has been sluggish, with only a limited number of community and township health centres currently providing mental health care. Restricted access to formal mental health services in China, particularly in rural areas, significantly hinders help-seeking behaviour among patients with mental health conditions. Additionally, stigma and low levels of mental health literacy further suppress the willingness to seek professional help [64–66]. Some patients with mental health issues also turn to traditional Chinese medicine practitioners for assistance [67,68]. As a result, an estimated 80% to 90% of individuals with diagnosable mental illnesses never receiving psychiatric care [67,69,70]. There are substantial doubts whether culture-specific ways of experiencing and manifesting psychological symptoms might make the DSM or ICD criteria less valid in China than in other countries [70,71], even though the GBD methodology accounts for these low rates of health seeking by only including studies that used standardised DMS or ICD criteria to determine the prevalence/burden of mental illness in the general population [6,11]. Some Chinese studies suggest participants with milder symptom and with significant morbidity are not captured by DSM-IV criteria, thus leading to a potential underestimation of the mental health burden [69,72,73]. Using semi-structured interviews (e.g., SCID) conducted by interviewers familiar with local cultural contexts could partially address this gap. Substantially higher total burdens have been identified in studies using these instruments [69,73]. Moreover, fear of stigmatisation might make Chinese individuals more likely to deny transient psychological ailments or report them in term of somatic symptoms rather than a mental condition [74,75]. Further in-depth studies, combining quantitative surveys with qualitative research exploring individuals’ lived experience with mental health disorders [76] are thus essential to establish the true burden of mental disorders in China and assess whether changes in health-seeking, mental health literacy or changing perception of stigma and discrimination have influenced the observed temporal and regional trends in GBD burden estimates for various mental disorders.

Thirdly, the generalisability of GBD estimates is limited by the lack of high-quality, regional-specific studies that provide provincial data. This is particularly true for in less developed areas. In these cases, regional data had to be extrapolated using the sequential DisMod MR 2.1 models, due to insufficient reliable source data. This limitation is exemplified by nearly identical DALY estimates for ADHD across several Chinese provinces. Additionally, data from most provinces during the pandemic exhibit wide uncertainty intervals, underscoring the inconclusive nature of the findings and significantly limiting their interpretability. Our data also showed the incidence of bulimia nervosa for males was nearly three times of females in 2021 (S2 Fig), despite limited surveys in China. Only one national epidemiological survey included in GBD study reported four individuals with bulimia nervosa and one with anorexia nervosa among 32552 adults in China [2]. This survey focused solely on adults, excluding teenagers (the peak age group for eating disorders), thereby significantly underestimating the prevalence. There are no specific epidemiology studies on bulimia nervosa, especially among males, published in China. These factors raise concerns about the quality of the data available in the GBD study concerning mental health. More robust and comprehensive data are needed to obtain more reliable results.

Lastly, given the cross-sectional nature of many data sources used in modelling GBD burden estimates, strong caveats must apply when trying to infer causality into and relationship between temporal and spatial patterns of GBD mental health burden and important social, economic and health policy settings.

## Conclusion

Our study revealed that despite a 30-year trend of overall declining mental health burden in China, the pandemic caused a reversal, bringing the burden back to levels observed in the 1990s. Self-harm burden decreased dramatically and remained stable during the pandemic. The burden of depressive disorders increased notably among the elderly, despite an overall decrease among all ages. Eating disorders showed a substantial rise correlated with economic development. During the pandemic, the burden of anxiety disorders surged in 2021, particularly among young adults, and the burden of depressive disorder rose transiently in 2020. These findings highlight the need for tailored interventions aimed at high-risk groups, like depression among the elderly, eating disorders in economically prosperous regions, and anxiety in young adults following the pandemic, to address the evolving mental health challenges effectively.

## Supporting information

S1 Checklist Guidelines for Accurate and Transparent Health Estimates Reporting (GATHER) compliance. (DOCX)

S1 Data Summary of mental disorders and self-harm case definitions and modelling. (PDF)

S2 Data Input sources by GBD for mental disorders and self-harm. (PDF)

S1 Table Changing DALYs of all mental disorders and self-harm in China in 5 years age group, 1990-2021.
(PDF)

S2 Table Changing DALYs of all mental disorders and self-harm in 34 provincial units in China, 1990-2021.
(PDF)

S1 Fig Trends of age-sex-specific mortality, DALYs, YLDs, YLLs, prevalence and incidence rate of all mental disorders and self harm in China, 1980-2021.
(PDF)

S2 Fig Spatial distribution of mortality, DALYs, YLDs, YLLs, prevalence and incidence rate of all mental disorders and self harm in China, 2021.
(PDF)
