# Supplementary material for: The disability-adjusted life years attributable to mental disorders and self-harm in China from 1990–2021: Findings from the global burden of disease study 2021
Source: PLOS Ment Health. 2025 Apr 9;2(4):e0000146. doi: 10.1371/journal.pmen.0000146 (PMC12798377; doi:10.1371/journal.pmen.0000146)
Supplement: S1 Data — (PDF) [file pmen.0000146.s002.pdf]

S1 Data. Summary of mental disorders and self-harm case definitions and modelling.

Mental disorders and self-harm case definitions and modelling

Cause	Definition	DSM-IV-TR and ICD-10 codes	
Major depressive disorder (MDD)	The presence of at least one major depressive episode, which is the experience of either depressed mood or loss of interest/pleasure, for most of every day, for at least two weeks. This must represent a change from the person's baseline and impaired functioning observed across social, occupational, and educational domains.	DSM-IV-TR: 296.21–24, 296.31–34<br>ICD-10: F32.0–9, F33.0–9<br><br>In 2020 and 2021, studies with screening measures were included.	Nonfatal Health Outcomes: https://www.healthdata.org/gbd/methods-appendices-2021/major-depressive-disorder
Dysthymia	A mood disorder involves chronically depressed mood for most of the day, less severe but longer lasting symptoms than MDD for at least two years (or at least one year in children and adolescents).	DSM-IV-TR: 300.4<br>ICD-10: F34.1	Nonfatal Health Outcomes https://www.healthdata.org/gbd/methods-appendices-2021/dysthymia
Anxiety disorders	Characterised by intense fear and distress, typically in combination with other physiological symptoms. Panic disorder, agoraphobia, specific phobia, social phobia, obsessive-compulsive disorder (OCD), post-traumatic stress disorder (PTSD), generalised anxiety disorder (GAD) including overanxious disorder in childhood, separation anxiety disorder (SAD), and anxiety disorder "not otherwise specified" (NOS) were specific anxiety disorders and included. Epidemiological estimates reporting an outcome for "any" or "total" anxiety disorders were included if they reported on at least three anxiety disorders.	DSM-IV-TR: 300.0-300.3, 208.3, 309.21, 309.81<br>ICD-10: F40-42, F43.0, F43.1, F93.0-93.2, F93.8	Nonfatal Health Outcomes https://www.healthdata.org/gbd/methods-appendices-2021/anxiety-disorders
Schizophrenia	Involves the experience of positive symptoms (e.g., delusions, hallucinations, thought disorder) and negative symptoms (e.g., flat affect, loss of interest, and emotional withdrawal).	DSM-IV-TR: 295.10-295.30, 295.60, 295.90<br>ICD 10: F20	Nonfatal Health Outcomes https://www.healthdata.org/gbd/methods-appendices-2021/schizophrenia
Bipolar disorder	Type I Bipolar is characterised by at least one manic episode, which can also alternate with a major depressive episode. Type II Bipolar is characterised by hypomanic episodes alternating with major depressive episodes. Cyclothymia is characterised by subsyndromal	DSM-IV-TR: 296.0–296.7, 296.89, 301.13<br>ICD-10: F30.0-F30.9, F31.0–F31.6, F31.8–F31.9, F34.0	Nonfatal Health Outcomes https://www.healthdata.org/gbd/methods-appendices-2021/bipolar-disorder

	hypomanic and major depressive episodes.<br>The burden for the entire spectrum of bipolar disorder was analysed simultaneously, rather than individually for each subtype of the disorder. At a minimum, epidemiological studies are needed to report on bipolar I and bipolar II.		
Anorexia nervosa	Characterised by refusal to maintain body weight at or above a minimally normal weight for age and height, intense fear of gaining weight, and disturbance in the way in which one's body weight or shape is experienced. In postmenarcheal females, amenorrhoea, ie, the absence of at least three consecutive menstrual cycles (this criterion was removed in DSM-5).	DSM-IV-TR: 307.1<br>ICD-10: F50.0-50.1	Nonfatal Health Outcomes https://www.healthdata.org/gbd/methods-appendices-2021/anorexia-nervosa-0<br><br>Causes of Death https://www.healthdata.org/gbd/methods-appendices-2021/anorexia-nervosa
Bulimia nervosa	Characterised by recurrent episodes of binge eating and inappropriate compensatory behaviour to prevent weight gain such as self-induced vomiting; misuse of laxatives, diuretics, enemas, or other medications; fasting; or excessive exercise. The binge eating and inappropriate compensatory behaviours both occur, on average, at least twice a week for three months (changed to once a week for three months in DSM-5). Self-evaluation is unduly influenced by body shape and weight. The disturbance does not occur exclusively during episodes of anorexia nervosa.	DSM-IV-TR: 307.51<br>ICD-10: F50.2	Nonfatal Health Outcomes https://www.healthdata.org/gbd/methods-appendices-2021/bulimia-nervosa
Conduct disorder	An externalising behaviour disorder characterised by a pattern of antisocial behaviour that violates the basic rights of others or major age-appropriate societal norms. Only childhood CD (ie, cases prior to 18 years of age) was modelled in GBD.	DSM-IV-TR: 312.81-312.89<br>ICD-10: F91	Nonfatal Health Outcomes https://www.healthdata.org/gbd/methods-appendices-2021/conduct-disorder
Attention-deficit/hyperactivity disorder	An externalising disorder characterised by persistent inattention and/or hyperactivity-impulsivity. Diagnosis requires six or more symptoms of inattention or hyperactivity- impulsivity to have persisted for at least six months, in two or more settings; with at least	DSM-IV-TR: 314.0, 314.01<br>ICD-10: F90	Nonfatal Health Outcomes<br><br>https://www.healthdata.org/gbd/methods-appendices-2021/attention-

	some impairing symptoms being present prior to 7 years of age (12 years of age in DSM-5)		deficithyperactivity-disorder
Autism Spectrum Disorder	A group of neurodevelopmental disorders with onset occurring in early childhood. Characterised by pervasive impairment in several areas of development, including social interaction and communication skills, along with restricted and repetitive patterns of behaviours and/or interests. Symptoms must be present in the early developmental period, cause clinically significant impairment, and not be better explained by intellectual impairment or global developmental delay.	DSM-IV-TR: 299.00, 299.80, 299.8, 299.8, 299.10<br><br>ICD-10: F84.0, F84.1, F84.2, F84.3, F84.4, F84.5, F84.8, F84.9<br><br>Amalgamated into a single disorder in the DSM-5.	Nonfatal Health Outcomes https://www.healthdata.org/gbd/methods-appendices-2021/autism-spectrum-disorders
Idiopathic developmental intellectual disability	Idiopathic developmental intellectual disability modelled as part of the Developmental intellectual disability Idiopathic developmental intellectual disability arises from any unknown source after the prevalence of all other sources of Intellectual Disability is accounted for.	Involves borderline disability (IQ 70-84), mild (IQ score 50 to 69), moderate (IQ Score 35-49), severe (IQ score 20-34), and profound (IQ score 0-19) idiopathic intellectual disability.	Nonfatal Health Outcomes for Developmental intellectual disability: https://www.healthdata.org/gbd/methods-appendices-2021/developmental-intellectual-disability
Other mental disorders	This is made up of an aggregate group of personality disorders. Personality disorders are characterised by pervasive, inflexible and maladaptive patterns of behaviour and inner experience which are markedly different from what is considered to be acceptable in the individual's culture. These disorders tend to be chronic and are associated with significant distress or disability.<br>• Paranoid personality disorder<br>• Schizoid personality disorder<br>• Schizotypal personality disorder<br>• Antisocial personality disorder<br>• Borderline personality disorder<br>• Histrionic personality disorder<br>• Narcissistic personality disorder<br>• Avoidant personality disorder<br>• Dependent personality disorder<br>• Obsessive-compulsive personality disorder<br>• Personality disorder not otherwise specified	DSM-IV-TR: 300.3, 301.0; 301.2, 301.22, 301.5–301.9<br><br>ICD-10: F60	Nonfatal Health Outcomes https://www.healthdata.org/gbd/methods-appendices-2021/other-mental-disorders

self-harm	Including self-harm by firearm and self-harm by other specified means	ICD-10: X60-X64.9, X66-X84.9, Y87.0	Nonfatal Health Outcomes of injuries: https://www.healthdata.org/gbd/methods-appendices-2021/injuries-0<br><br>Causes of Death of injuries https://www.healthdata.org/gbd/methods-appendices-2021/injuries

Note: DSM-IV-TR: Diagnostic and Statistical Manual of Mental Disorders Fourth Edition, Text Revision; ICD-10: International Classification of Diseases and Related Health Problems 10th Revision.

Major depressive disorder

This flowchart shows the analytical strategy for the non-fatal health outcome estimation process of major depressive disorder.

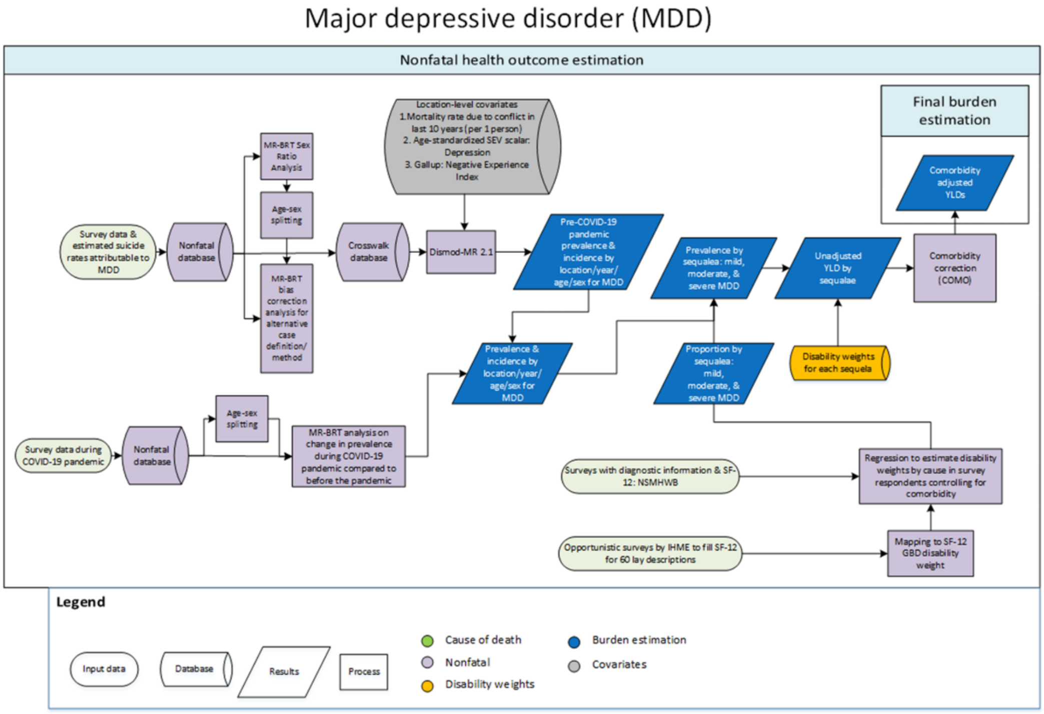

The epidemiological systematic literature review for MDD was conducted involving electronic searches of the peer-reviewed literature, the grey literature, and expert consultation. The grey literature searches and expert consultation were included in GBD 2021. The GBD inclusion criteria are: 1) from 1980 onward; 2) based on the clinical threshold by the DSM or ICD; 3) enough information on study method and sample characteristics to assess the quality of the study; and 4) study samples must be representative of the general population. No limitation was set on the language of publication. Estimates were further split by sex and age based on the available data, a meta-regression—Bayesian, regularised, trimmed (MR-BRT) analysis, or DisMod-MR 2.1. Estimates with known biases were adjusted. During the pandemic (2020 and 2021), an update to the systematic literature review was carried out in two stages. Longitudinal studies with samples representative of the general population were preferred, but cross-sectional studies during the pandemic were also accepted if comparable pre-COVID prevalence data were identified. Studies reporting on probable depressive disorders using established screening measures (eg, the Patient Health Questionnaire-9) were also included, due to lack of available data. Additionally, studies using screening measures of psychological distress or both depression and anxiety together were included. The modelling strategy used in GBD 2021 was the same as GBD 2019, except for COVID-19 adjustment. DisMod-MR 2.1 was used to model the (pre-COVID-19) epidemiological data for MDD. The 2020 and 2021 age-, sex-, and location-specific MDD prevalence estimates were then adjusted by the predicted logit change from the MR-BRT model for every day of 2020 and 2021. Then, the point prevalence estimates for 2020 and 2021 were calculated as the average daily prevalence (https://www.healthdata.org/gbd/methods-appendices-2021/major-depressive-disorder)

Dysthymia

This flowchart shows the analytical strategy for the non-fatal health outcome estimation process of dysthymia.

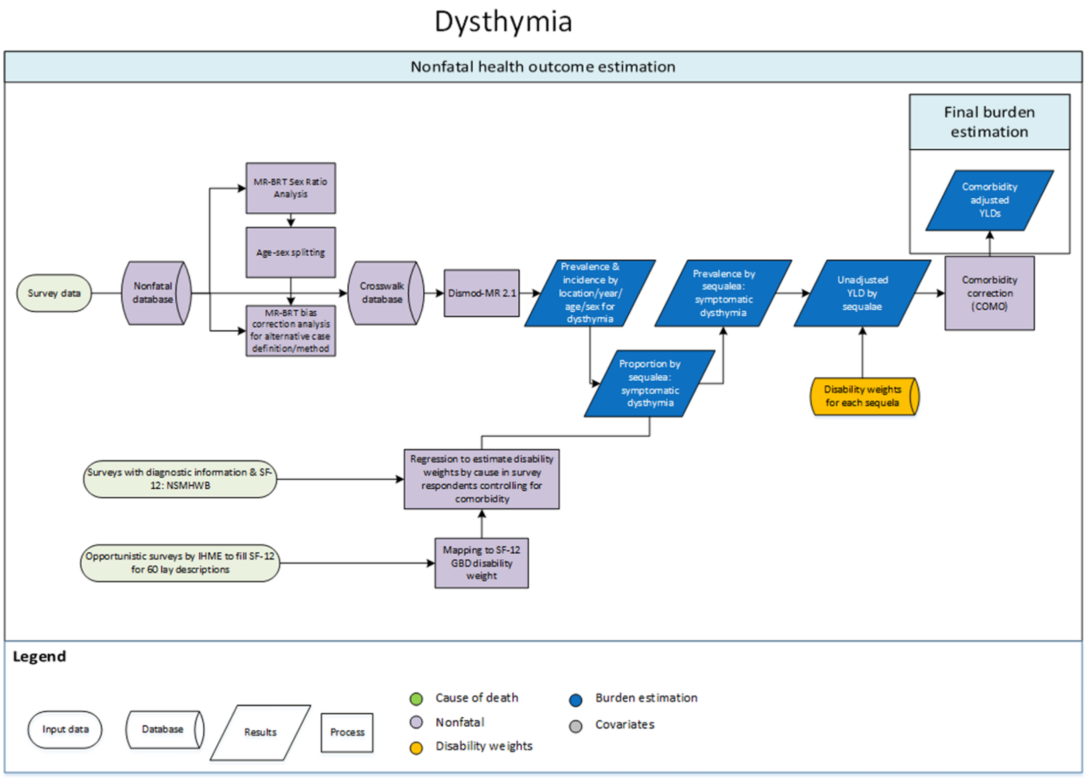

The epidemiological systematic literature review for dysthymia was conducted involving electronic searches of the peer-reviewed literature, the grey literature, and expert consultation. The grey literature searches and expert consultation were included in GBD 2021. Estimates were further split by sex and age based on the available data, a meta-regression—Bayesian, regularised, trimmed (MR-BRT) analysis, or DisMod-MR 2.1. Estimates with known biases were adjusted. Then, DisMod MR 2.1 was used to model the data for dysthymia. GBD 2021 and GBD 2019 are similar in modelling strategy (https://www.healthdata.org/gbd/methods-appendices-2021/dysthymia).

Anxiety disorders

This flowchart shows the analytical strategy for the non-fatal health outcome estimation process of anxiety disorders.

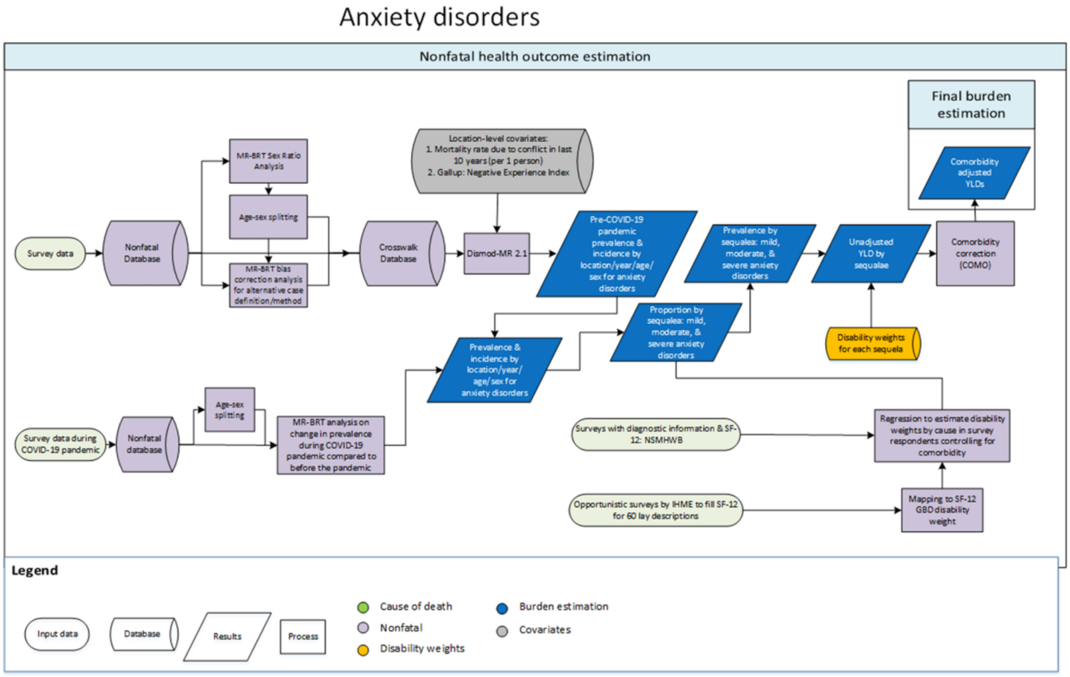

The epidemiological systematic literature review for anxiety disorders was conducted involving electronic searches of the peer-reviewed literature, the grey literature, and expert consultation. The grey literature searches and expert consultation were included in GBD 2021. Estimates were further split by sex and age based on the available data, a meta-regression—Bayesian, regularised, trimmed (MR-BRT) analysis, or DisMod-MR 2.1. Estimates with known biases were adjusted. During the pandemic (2020 and 2021), an update to the systematic literature review was carried out in two stages. Longitudinal studies with samples representative of the general population were preferred, but cross-sectional studies during the pandemic were also accepted if comparable pre-COVID prevalence data were identified. Studies reporting on probable anxiety disorders using established screening measures (eg, the General Anxiety Disorder-7) were also included, due to the lack of available data. Additionally, studies using screening measures of psychological distress or both depression and anxiety together were included. The modelling strategy used in GBD 2021 was the same as GBD 2019, except for COVID-19 adjustment. DisMod-MR 2.1 was used to model the (pre-COVID-19) epidemiological data for MDD. The 2020 and 2021 age-, sex-, and location-specific MDD prevalence estimates were then adjusted by the predicted logit change from the MR-BRT model for every day of 2020 and 2021. Then, the point prevalence estimates for 2020 and 2021 were calculated as the average daily prevalence (https://www.healthdata.org/gbd/methods-appendices-2021/anxiety-disorders).

Schizophrenia

This flowchart shows the analytical strategy for the non-fatal health outcome estimation process of schizophrenia.

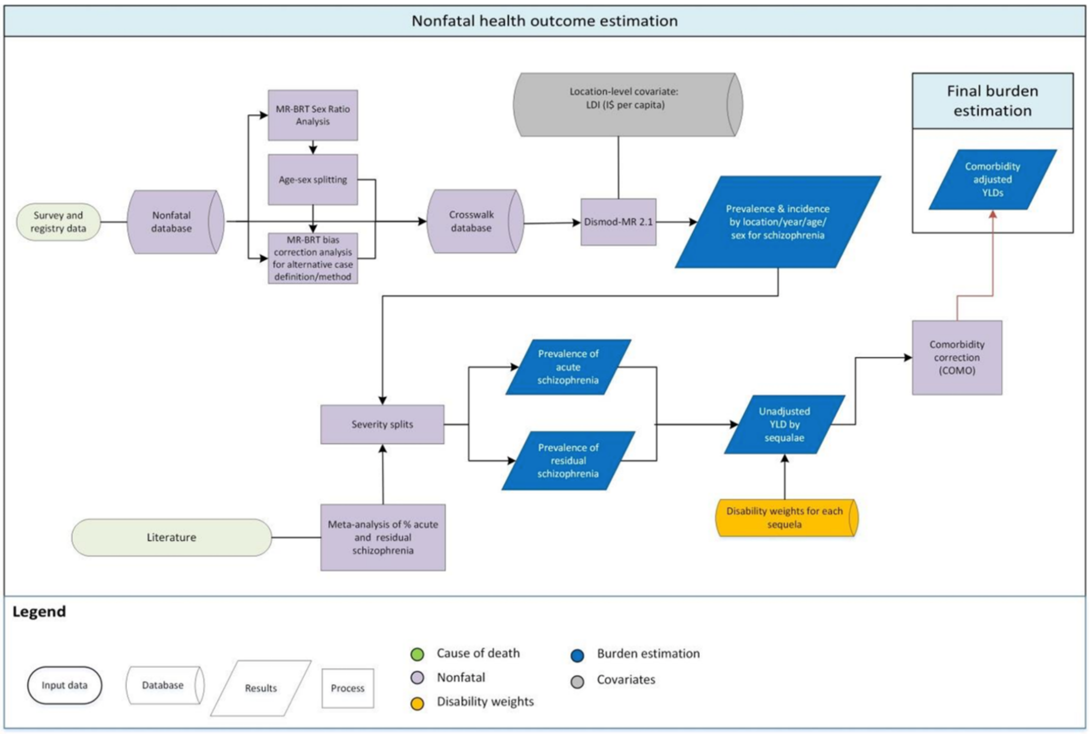

The epidemiological systematic literature review for schizophrenia was conducted involving electronic searches of the peer-reviewed literature, the grey literature, and expert consultation. The grey literature searches and expert consultation were included in GBD 2021. Estimates were further split by sex and age based on the available data, a meta-regression—Bayesian, regularised, trimmed (MR-BRT) analysis, or DisMod-MR 2.1. Estimates with known biases were adjusted. Then, DisMod MR 2.1 was used to model the data. GBD 2021 and GBD 2019 are similar in modelling strategy (https://www.healthdata.org/gbd/methods-appendices-2021/schizophrenia).

Bipolar disorder

This flowchart shows the analytical strategy for the non-fatal health outcome estimation process of schizophrenia.

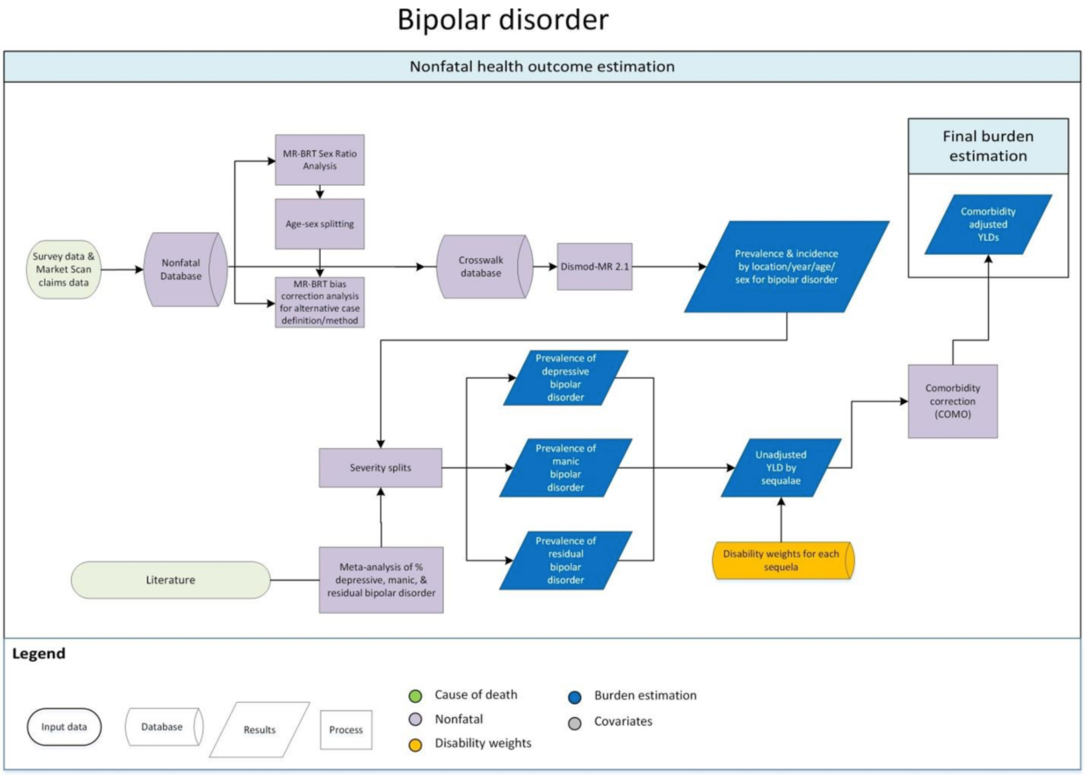

The epidemiological systematic literature review for bipolar disorder was conducted involving electronic searches of the peer-reviewed literature, the grey literature, and expert consultation. The grey literature searches and expert consultation were included in GBD 2021. Estimates were further split by sex and age based on the available data, a meta-regression—Bayesian, regularised, trimmed (MR-BRT) analysis, or DisMod-MR 2.1. Estimates with known biases were adjusted. United States (US) MarketScan data were utilised to incorporate detailed prevalence estimates by state, sex, and age in the modelling. MarketScan estimates were compared with National Comorbidity Survey Replication (NCS-R, a survey representative of the general US population) to adjust all MarketScan estimates before entering the bipolar disorder model. Then, DisMod MR 2.1 was used to model the data. GBD 2021 and GBD 2019 are similar in modelling strategy (https://www.healthdata.org/gbd/methods-appendices-2021/bipolar-disorder).

Anorexia nervosa (AN)

This flowchart shows the analytical strategy for the non-fatal health outcome estimation process of Anorexia nervosa.

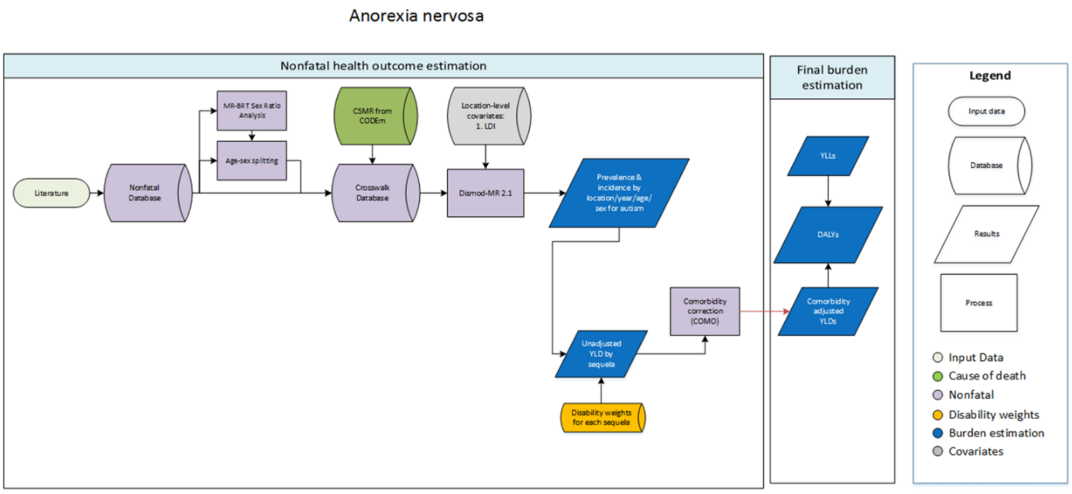

The epidemiological systematic literature review for AN was conducted involving electronic searches of the peer-reviewed literature, the grey literature, and expert consultation. The grey literature searches and expert consultation were included in GBD 2021. Estimates were further split by sex and age based on the available data, a meta-regression—Bayesian, regularised, trimmed (MR-BRT) analysis, or DisMod-MR 2.1. Estimates with known biases were adjusted. Mortality data (standardised mortality ratios and relative risks) were excluded. Then, DisMod MR 2.1 was used to model the data. (https://www.healthdata.org/gbd/methods-appendices-2021/anorexia-nervosa-0).

This flowchart shows the analytical strategy for the cause-of-death outcome estimation process of Anorexia nervosa.

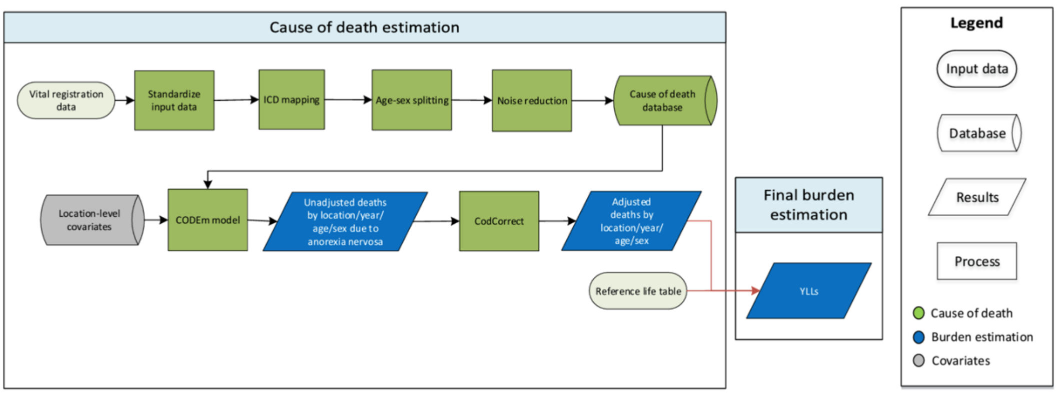

Data used to estimate anorexia nervosa mortality included centrally prepped vital registration data from the cause of death database. AN was modelled with the standard CODEm approach and came under the eating disorders parent model. Several covariates were applied to this model. In AN model, age was restricted to deaths occurring between 5 and 49 years. In GBD 2021, a decision was made to remove bulimia nervosa as a cause of death due to the limited data available. (https://www.healthdata.org/gbd/methods-appendices-2021/anorexia-nervosa)

Bulimia nervosa (BN)

This flowchart shows the analytical strategy for the non-fatal health outcome estimation process of bulimia nervosa.

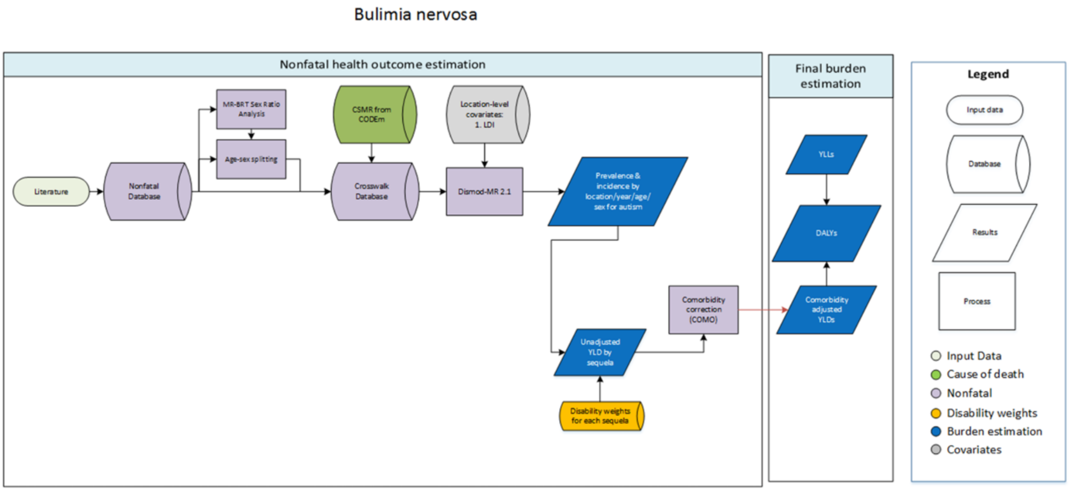

The epidemiological systematic literature review for BN was conducted involving electronic searches of the peer-reviewed literature, the grey literature, and expert consultation. The grey literature searches and expert consultation were included in GBD 2021. Estimates were further split by sex and age based on the available data, a meta-regression—Bayesian, regularised, trimmed (MR-BRT) analysis, or DisMod-MR 2.1. Estimates with known biases were adjusted. Then, DisMod MR 2.1 was used to model the data. Incidence prior to 10 years of age or onward from 40 years of age were not included. In GBD 2021, BN was removed as a cause of death due to the limited data available (https://www.healthdata.org/gbd/methods-appendices-2021/bulimia-nervosa)

Conduct disorder

This flowchart shows the analytical strategy for the non-fatal health outcome estimation process of dysthymia.

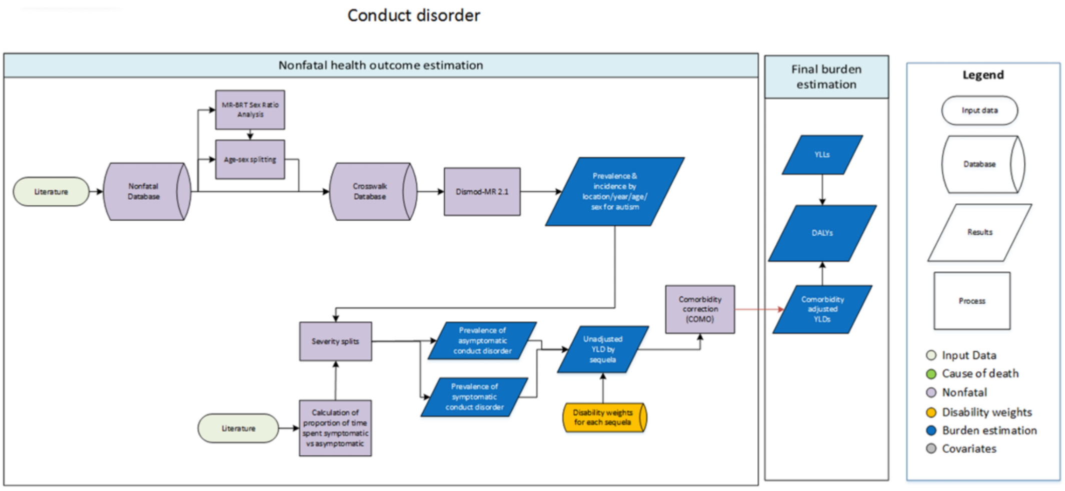

The epidemiological systematic literature review for conduct disorder was conducted involving electronic searches of the peer-reviewed literature, the grey literature, and expert consultation. The grey literature searches and expert consultation were included in GBD 2021. Estimates were further split by sex and age based on the available data or a meta-regression—Bayesian, regularised, trimmed (MR-BRT) analysis. Estimates with known biases were adjusted. Then, DisMod MR 2.1 was used to model the data. GBD 2021 and GBD 2019 are similar in modelling strategy (https://www.healthdata.org/gbd/methods-appendices-2021/conduct-disorder).

Attention-deficit/hyperactivity disorder (ADHD)

This flowchart shows the analytical strategy for the non-fatal health outcome estimation process of Attention-deficit/hyperactivity disorder.

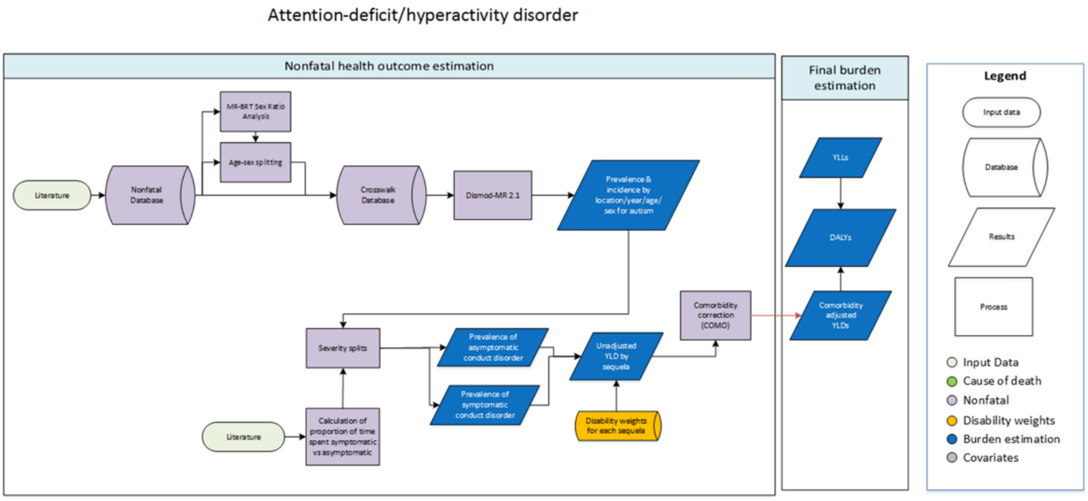

The epidemiological systematic literature review for ADHD was conducted involving electronic searches of the peer-reviewed literature, the grey literature, and expert consultation. The grey literature searches and expert consultation were included in GBD 2021. Estimates were further split by sex and age based on the available data or a meta-regression—Bayesian, regularised, trimmed (MR-BRT) analysis. Estimates with known biases were adjusted. Then, DisMod MR 2.1 was used to model the data. GBD 2021 and GBD 2019 are similar in modelling strategy (https://www.healthdata.org/gbd/methods-appendices-2021/attention-deficithyperactivity-disorder)

Autism Spectrum Disorder (ASD)

This flowchart shows the analytical strategy for the non-fatal health outcome estimation process of Attention-deficit/hyperactivity disorder.

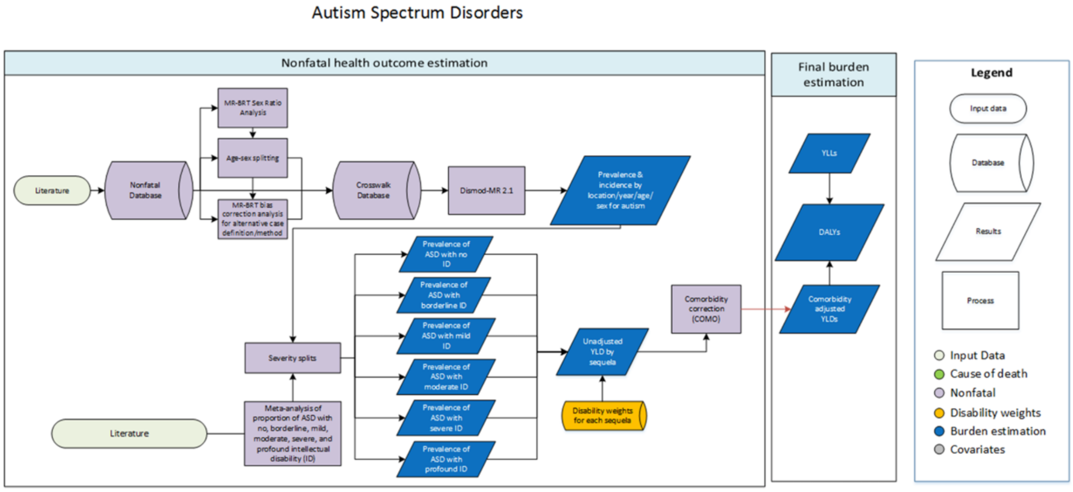

The epidemiological systematic literature review for ASD was conducted involving electronic searches of the peer-reviewed literature, the grey literature, and expert consultation. The grey literature searches and expert consultation were included in GBD 2021. Estimates of the prevalence of sub-disorders in the Autism spectrum based on the previous diagnostic guide. Estimates were further split by sex and age based on the available data or a meta-regression—Bayesian, regularised, trimmed (MR-BRT) analysis. Estimates with known biases were adjusted. Then, DisMod MR 2.1 was used to model the data for ASD. Two changes were made in GBD 2021 compared with GBD 2019. First, estimates derived from administrative prevalence, which would lead to an underestimation of prevalence, were excluded since administrative prevalence. Second, estimates of the disability weights for each sequelae of ASD were implemented. The two changes increased ASD prevalence and burden estimates. (https://www.healthdata.org/gbd/methods-appendices-2021/attention-deficithyperactivity-disorder)

Idiopathic developmental intellectual disability

This flowchart shows the analytical strategy for the non-fatal health outcome estimation process of Developmental intellectual disability.

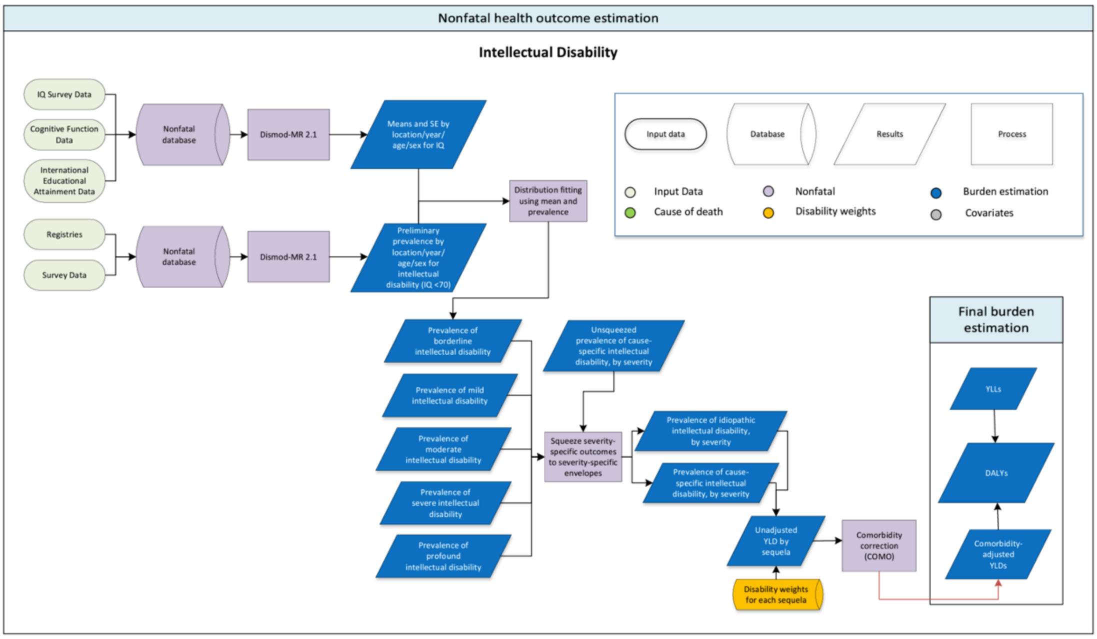

The epidemiological systematic literature review for Idiopathic developmental intellectual disability was modelled as part of the Developmental intellectual disability. The prevalence of intellectual disability (IQ score <70) is estimated from the literature since January 1, 1990. Studies that estimated the general population prevalence of intellectual disability were included. Studies did not include a case definition based on intelligence quotient (IQ) and did not investigate non-representative groups (e.g. hospital patients or people of a specific ethnicity) were excluded. MR-BRT were used to split both-sex data points into sex-specific data. DisMod MR 2.1 was used to model the total prevalence of intellectual disability of level IQ <70. Then, the total prevalence of idiopathic intellectual disability was split into four severity levels: mild (IQ 50-69), moderate (IQ 35-49), severe (IQ 20-34), and profound (IQ below 20). The final severity level, borderline disability (IQ 70-84) was estimated via another random-effects meta-analysis. Third, the estimated prevalence of each aetiology-specific intellectual disability using models of the different causes. Prevalence of idiopathic intellectual disability was calculated by subtracting all severity- and aetiology-specific intellectual disability from the severity-specific envelope assuming the residuals to represent idiopathic disability(https://www.healthdata.org/gbd/methods-appendices-2021/developmental-intellectual-disability).

Other mental disorders

This flowchart shows the analytical strategy for the non-fatal health outcome estimation process of other mental disorders

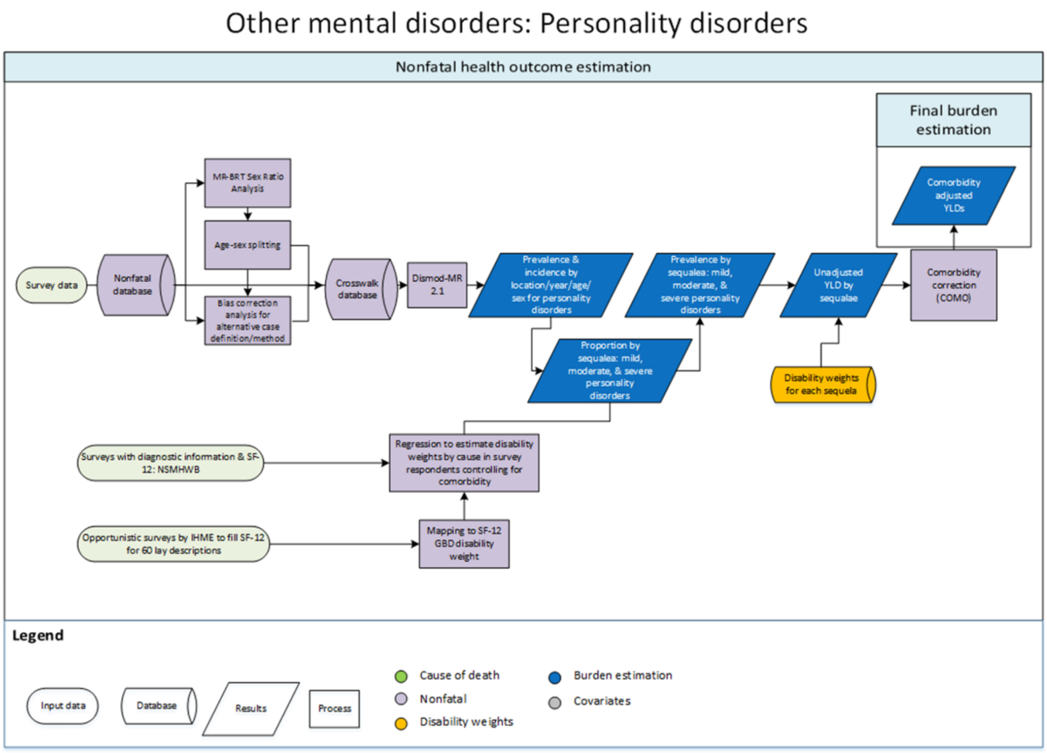

The resource data for other mental disorders come from two surveys: the US National Epidemiological Survey on Alcohol and Related Conditions (NESARC, conducted in two waves from 2001–2002 and 2004–2005) and the Australian National Survey of Mental Health and Wellbeing of Adults (NSMHWB, conducted in 1997). An adjustment for comorbidity was used. Estimates with known biases were adjusted. Then, DisMod MR 2.1 was used to model the data for personality disorders. GBD 2021 and GBD 2019 are similar in modelling strategy. (https://www.healthdata.org/gbd/methods-appendices-2021/other-mental-disorders)

Self-harm

This flowchart shows the analytical strategy for the non-fatal health outcome estimation process of injuries

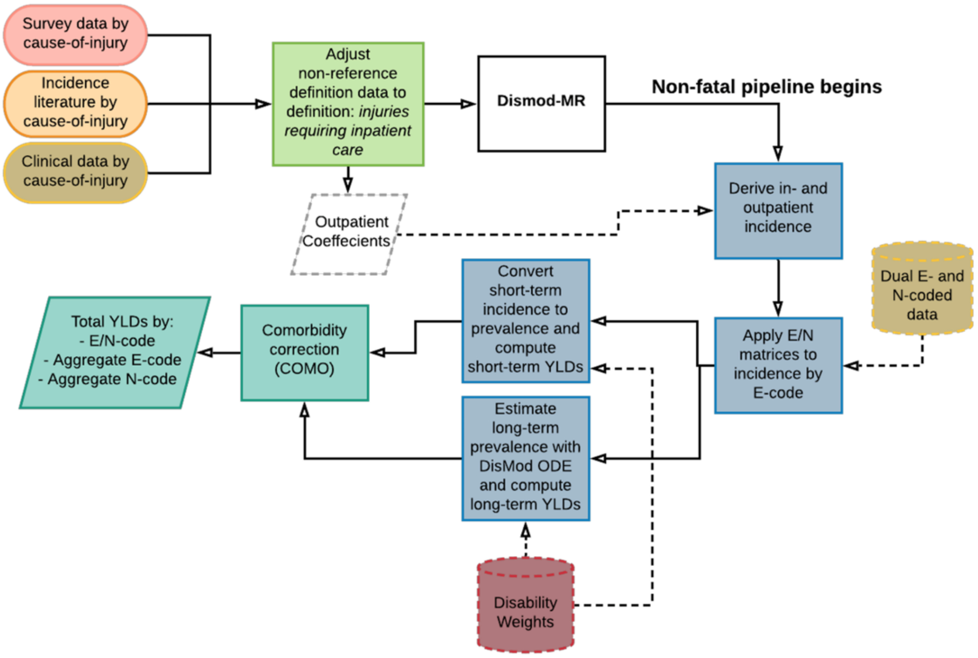

Self-harm was modelled as part of the Injuries. Data from hospital records, emergency department records, insurance claims, and population-representative surveys were used to estimate morbidity from injuries. Incidence for cause-of-injury categories was modelled using the Bayesian meta-regression method DisMod-MR 2.1. Two categories of injury severity, inpatient and outpatient injuries, were separately estimated. For injuries with large fatal and non-fatal inconsistencies (including self-harm), the final MR-BRT predictions were uploaded as EMR input data to DisMod-MR 2.1 models. Then data were adjusted. Details can be seen on website: https://www.healthdata.org/gbd/methods-appendices-2021/injuries-0

This flowchart shows the analytical strategy for the cause-of-death outcome estimation process of injuries.

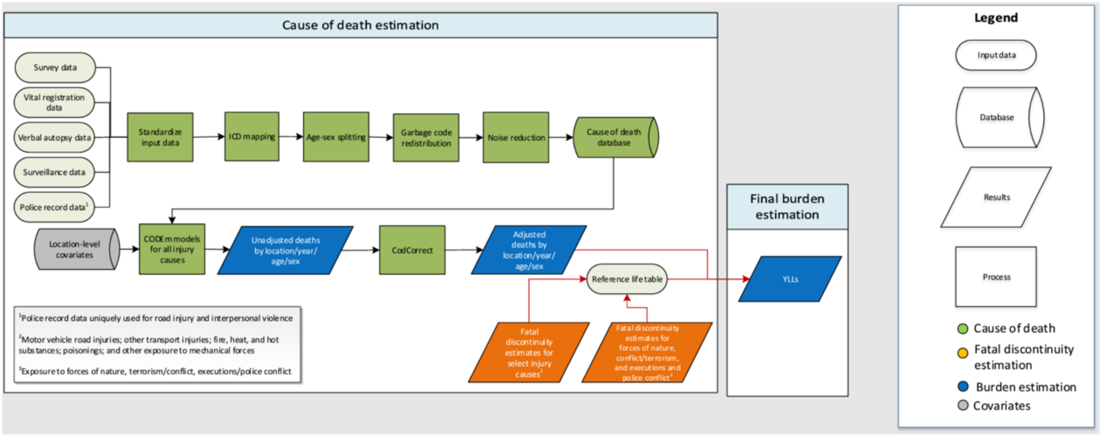

Self-harm was modelled as part of the Injuries. Mortality of self-harm includes data from vital registration, verbal autopsy, mortality surveillance, censuses, and surveys. The standard the Cause Of Death Ensemble Model (CODEm) approach was applied to estimate deaths due to all causes of injury in GBD 2021. Several covariates were applied to this model. (https://www.healthdata.org/gbd/methods-appendices-2021/injuries)
