# Supplementary material for: The disability-adjusted life years attributable to mental disorders and self-harm in China from 1990–2021: Findings from the global burden of disease study 2021
Source: PLOS Ment Health. 2025 Apr 9;2(4):e0000146. doi: 10.1371/journal.pmen.0000146 (PMC12798377; doi:10.1371/journal.pmen.0000146)
Supplement: S2 Data — (PDF) [file pmen.0000146.s003.pdf]

S2 Data. Input sources by GBD for mental disorders and self-harm
(https://ghdx.healthdata.org/gbd-2021/sources?components=5&causes=558&locations=6)

Studies from 34 province units in China used in Nonfatal Health Outcomes for Mental disorders

	**Citation**
1	Huang L-S, Chen Y-F, Lin X-Z, Wu Y-T. A 10 year investigation on incidence of schizophrenia in community. Chin J Nerv Ment Dis. 1990; 16(2): 100-103.
2	Lee S, Ng KL, Tsang A. A community survey of the twelve-month prevalence and correlates of bipolar spectrum disorder in Hong Kong. J Affect Disord. 2009; 117(1-2): 79-86.
3	Chen R, Hu Z, Qin X, Xu X, Copeland JRM. A community-based study of depression in older people in Hefei, China – the GMS-AGECAT prevalence, case validation and socio-economic correlates. Int J Geriatr Psychiatry. 2004; 19(5): 407-13.
4	Guerra M, Prina AM, Ferri CP, Acosta D, Gallardo S, Huang Y, Jacob KS, Jimenez-Velazquez IZ, Llibre Rodriguez JJ, Liu Z, Salas A, Sosa AL, Williams JD, Uwakwe R, Prince M. A comparative cross-cultural study of the prevalence of late life depression in low and middle income countries. J Affect Disord. 2016; 190: 362–8.
5	Zhang J-X, Weng Z, Qin Q-L, Chai X-S, Ma D-D. A comparison study of epidemiology between Type I and Type II schizophrenia in Shandong. Chin Ment Health J. 2001; 15(2): 100-102.
6	Huang J-M, Lv J-J, Xu J-Q, Chen X-Q, Huo B-Q, Jiang L-Y, Zeng J-S, Liu L-H. A Cross-sectional Study and Quality of Life Evaluation for Mental Disorders in a Community. J Prev Med Inf. 2001; 17(6): 420-2.
7	Huon GF, Mingyi Q, Oliver K, Xiao G. A large-scale survey of eating disorder symptomatology among female adolescents in the People's Republic of China. Int J Eat Disord. 2002; 32(2): 192-205.
8	Guo Y-S. A Survey of Main Hereditary Diseases in Shandong Province. J Shandong Univ Health Sci. 1993; 31(4): 271-4.
9	Zhang G, Xu Y, Huang C, Yang H, Lu Y. An epidemiolgoical survey of autism in the Yunyan district of Guiyang city. J Guiyand Med Coll. 2009; 34(4): 463–465.
10	Song W-H. An epidemiologic investigation of mental disorders in the rural area of Meishan County. West China Med J. 1988; 3(1): 10-1.
11	Wong Z, Zhang J-X, Ma D-D, Ma S-P, Li X-F, Jiang Y-Q, Xu L-Y, Chen D-C, Cao X-Y, Meng G-Y, He J-Y, Sun L-M, Zhang S-J, Zhu B-J, Cui W-C, Gong P-J, Hu B-W, Liu Z-X, Mo Z-P, Li X-Q. An epidemiological investigation of mental disorders in Shandong province in 1984 and 1994. Chin J Psychiatry. 1998; 31(4): 222-224.
12	Shen YC, Wang YF, Yang XL. An epidemiological investigation of minimal brain dysfunction in six elementary schools in Beijing. J Child Psychol Psychiatry. 1985; 26(5): 777-87.
13	Hou G-M, Wang S-R. An epidemiological study of mental disorders in Wuxi city of Jiangsu Province. J Clin Psychiatry. 1996; 6(4): 206-208.
14	Qh Z, Zw L, Zx Z. An epidemiological study on etiology of mental retardation. Nat Med J Chin. 1994; 74(3): 134-7.
15	Zhou K-Y, Gao M-H, Yang C-H, Zhang J-N, Chen Y-Z, Song J-Z, Zhuang Y-Y, Zhang X-Y, Zhang W, Wen F-Q. An Epidemiological Survey of Attention Deficit Hyperactivity Disorder in School-age Children in Shenzhen. Chin J Contemp Pediatr. 2012; 14(9): 689-92.
16	Zhao Z-H, Huang Q-Y, Li J, Deng H-H, Huang X-M, Su J-H, Dang W-M, Yang L, Huang J-K, Zhang W-M, Deng Y, Zhou W-C, Qiu C, Lu W-C, Chen Y-W, Zhong S-J, Chen B-Y, Zeng Q-M, Mei F. An Epidemiological survey of mental disorders in Guangzhou area. Chin J Nerv Ment Dis. 2009; 35(9): 530-534.
17	Wang S-J, Wei Z-Y, Niu F Zhang G, Wei X-B, Zhang K-M, Cheng X-F, Xi X-Y, Wu Q-Y, Liu Q, Ning N-Y, Yu S-J, Gao K, Liu H-F, Zhang X-G, Guo H, Guo C-L, Liu X-H, Cai H-Y, Peng X-F. An epidemiological survey on schizophrenia in Fuyang, Anhui. J

	Clin Psychol Med. 2002; 12(1): 3-4.
18	Li X, Zhu Z, Zhu J, Lu W, Zhu J. An Epidemiological Survey on schizophrenia in Yancheng of Jiangsu Province. Nerv Dis Mental Hyg. 2003; 3(5): 356-57.
19	Wang W, Zhai L, Zheng L, Zhu J, Qu X, Feng Q, et al. An epidemiology survey of autism in Jiangsu province. Chin J Behav Med Sci. 2003; 12(2): 173–174.
20	Sun D-F, Yi J-M, Li M, Li Y-L. An investigation of ADHD and family environment in 8235 school children aged 4-16 years in Northern Shangdong. Chin J Nerv Ment Dis. 2009; 35(11): 650-654.
21	Zhu J-Y. An Investigation of Mental Disorders for 6-16 years old Children and Adolescents in Jiangmen City. J Psychiatry. 2016; 29(1): 16-8.
22	Zhang Y, Li H-H, Li C-J, Yang Z, Xiong L, Chen L, Huang Y, Nong Z, Zeng D-Y. Analysis of Risk Factors and Prevalence of ADHD among Elementary School Students in Liuzhou City. Chin J Sch Health. 2017; 38(2): 242-8.
23	Liang M, Chen Q, Liu C, Gu Z, Mai M, Jiang Y. Analysis of screening for autism in preschool children in Maoming. Matern Child Health Care China. 2011; 26: 1164-1165.
24	Wang YJ, Wang WR. Analysis on Application of DSM-V in Early Screening of Autism Spectrum Disorder Children. Matern Child Health Care China. 2015; 13: 2011-4.
25	Diao W-L, Wang Z-P, Fu Z-G, Yu Q-F, Yu C-Y, Na J. Analysis on Prevalences of Mood Disorders, Anxiety and Alcohol Abuse Among Different Occupational Populations in Liaoning Province. Chin J Ind Med. 2008; 21(5): 325-7.
26	Huang L-C, Zheng S-M, Luo W-Y, Chen J-Y. Analysis on the Epidemic Condition about Psychosis Disease of the Local People in Huojie Town Dongguan City. Int Med Health Guid News. 2006; 12(18): 128-30.
27	Luo W-Y, Huang X-M, Gong Z-Y, Huang W-J. Analysis on the epidemic condition about severe psychiatric diseases in Houjie Town of Dongguan in 2012. Chin Prim Health Care. 2014; 28(1): 100-102.
28	Gao L-J, Wqu Q-H, Ning N, Sun H, Cui Y. Analysis on the prevalence and causes of mental disabilities of population in Heilongjiang province. Med Soc. 2008; 21(9): 4-6.
29	Zhang L, Zhang D, Fang J, Wan Y, Tao F, Sun Y. Assessment of Mental Health of Chinese Primary School Students Before and After School Closing and Opening During the COVID-19 Pandemic. JAMA Netw Open. 2020; 3(9): e2021482.
30	Sun GZ, Ye N, Zhang NJ, Li Y, Chen S, Chang Y, Li Z, Sun YX. Association between CHADS2 score, depressive symptoms, and quality of life in a general population. BMC Psychiatry. 2017; 17(1): 80.
31	Lu L, Shi Q-J, Zhong Y-F, Wang Z-M, Chen Z-Y. Attention deficit hyperactivity disorder and the related factors in children in Wuhan city: an analysis of 2199 questionnaires coming from 12 grades. Chin J Clin Rehab. 2005; 9(12): 116-118.
32	Huang Y, Zheng S, Xu C, Lin K, Wu K, Zheng M, Zhang J, Xu H. Attention-deficit hyperactivity disorder in elementary school students in Shantou, China: prevalence, subtypes, and influencing factors. Neuropsychiatr Dis Treat. 2017; 13: 785-792.
33	Zhang X, Ji C-Y. Autism and mental retardation of young children in China. Biomed Environ Sci. 2005; 18(5): 334–40.
34	Sun X, Allison C, Wei L, Matthews FE, Auyeung B, Wu YY, Griffiths S, Zhang J, Baron-Cohen S, Brayne C. Autism prevalence in China is comparable to Western prevalence. Mol Autism. 2019; 10: 7.
35	Feng L, Li P, Lu C, Tang W, Mahapatra T, Wang Y, Wang X, Ma Y, Ben Y, Cao X, Mahapatra S, Ling M, Gou A, Wang Y, Xiao J, Hou M, Wang X, Lin B, Chen R, Wang F, Hu Z. Burden and correlates of geriatric depression in the Uyghur elderly population, observation from Xinjiang, China. PLoS One. 2014; 9(12): e114139.
36	World Health Organization (WHO). China World Health Survey 2002. Geneva, Switzerland: World Health Organization (WHO), 2005.
37	Li TW, Liang L, Ho PL, Yeung ETF, Hobfoll SE, Hou WK. Coping resources mediate the prospective associations between disrupted daily routines and persistent psychiatric symptoms: A population-based cohort study. J Psychiatr Res. 2022; 152: 260-268.
38	Wang GX, Li X, Fang LY, Xu H. Correlation analysis and investigation of autism spectrum disorder in 2-6 years old children of Zaozhuang. Chin J Child Health Care.

	2015; 23(12): 1322-1324.
39	Bromet E, Andrade LH, Hwang I, Sampson NA, Alonso J, de Girolamo G, de Graaf R, Demyttenaere K, Hu C, Iwata N, Karam AN, Kaur J, Kostyuchenko S, Lépine J-P, Levinson D, Matschinger H, Mora MEM, Browne MO, Posada-Villa J, Viana MC, Williams DR, Kessler RC. Cross-national epidemiology of DSM-IV major depressive episode. BMC Med. 2011; 9: 90.
40	Xu Y, Yang J, Gao J, Zhou Z, Zhang T, Ren J, Li Y, Qian Y, Lai S, Chen G. Decomposing socioeconomic inequalities in depressive symptoms among the elderly in China. BMC Public Health. 2016; 16: 1214.
41	Choi EPH, Hui BPH, Wan EYF. Depression and Anxiety in Hong Kong during COVID-19. Int J Environ Res Public Health. 2020; 17(10).
42	Chen X, Cheng HG, Huang Y, Liu Z, Luo X. Depression symptoms and chronic pain in the community population in Beijing, China. Psychiatry Res. 2012; 200(2-3): 313-7.
43	Hou F, Cerulli C, Wittink MN, Caine ED, Qiu P. Depression, social support and associated factors among women living in rural China: a cross-sectional study. BMC Womens Health. 2015; 15: 28.
44	Zhong B, Ding J, Chen H, Li Y, Xu H, Tong J, Wang A, Tang G, Zhu J, Yang D, Liu B, Wang Q, Cheng W, Yin E, Xu M, Zhang T, Hu T, Feng X, Li H, Dan T, Cheng G, Zhang J, Li H, Zhu J. Depressive disorders among children in the transforming China: an epidemiological survey of prevalence, correlates, and service use. Depress Anxiety. 2013; 30(9): 881-92.
45	Wang L, Feng Z, Yang G, Yang Y, Wang K, Dai Q, Zhao M, Hu C, Zhang R, Liu K, Guang Y, Xia F. Depressive symptoms among children and adolescents in western china: An epidemiological survey of prevalence and correlates. Psychiatry Res. 2016; 246: 267-274.
46	Zhou M, Zhang G, Rozelle S, Kenny K, Xue H. Depressive Symptoms of Chinese Children: Prevalence and Correlated Factors among Subgroups. Int J Environ Res Public Health. 2018; 15(2).
47	Fang M, Yang Y, Wang X-Q, Tang Y, Zhuang X-L. Diagnosis of puerile attention deficit hyperactivity disorder in Xichang City, Liangshan Yi Autonomous Prefecture, Sichuan Privince. Chin J Obstet Gynecol Pediatr (Electron Ed). 2012; 8(6): 721-724.
48	Ni MY, Li TK, Pang H, Chan BH, Yuan BY, Kawachi I, Schooling CM, Leung GM. Direct Participation in and Indirect Exposure to the Occupy Central Movement and Depressive Symptoms: A Longitudinal Study of Hong Kong Adults. Am J Epidemiol. 2016; 184(9): 636-643.
49	Duan G, Yao M, Jin Y, Pan K, Li Z. Early Detection of Autism Spectrum Disorders in Infants and Young Children. Henan J Prev Med. 2013; 24(4): 255-260.
50	Liu J. Early screening and a clinical records study of autism spectrum disorder [master's thesis]. Shanghai, China: Fudan University Shanghai Medical College, 2011.
51	Wei Z, Ren L, Weng L, Gao W, He H, Wang W, Wan G. Early screening on autism spectrum disorders in 18-24 months old children in Shenzhen: 3 years follow up study. Chin J Child Health Care. 2012; 20(4): 354–357.
52	Zhu X-G, Wan C, Lan Z-S, Chen J-P. Epidemic investigation of the mental disorders in Yichun city, Jiangxi province. Chin J Epidemiol. 2004; 6: 526.
53	Li Z, Zhao LL, Zhong YF. Epidemiolgical Investigation on 10,000 Children of 0-6 Years with Autism in Changchun city. J Community Med. 2014; 12(18): 11–3.
54	Zhang M-L, Yuan G-Z, Mao J-Y, Yao J-J, Chu X, Qian Y-C, Ji Q, Zhang F-J, Qi S-G, Xu W-W, Zhang X, Tang R-C, Liu X-W, Huang Y-P, Zhou D-X, Jiang Q-Y, Bao Z-H. Epidemiologic Features of the Prevalence Rates of Various Mental Disorders and their Related Factors in Wuxi City: a Cross-sectional Survey. Chin J Clin Rehab. 2004; 8(24): 4950-2.
55	Shu C-B, Chen C-A, Hu A-R, Geng D-L. Epidemiological Analysis of Severe Mental Illness in Suizhou Economic Development District. Pract Prev Med. 2013; 20(3): 305-7.
56	Guo R. Epidemiological investigation analysis of 5000 Chinese between 0 and 6 years old with childhood autism in Tianjin city. Chin J Clin Rehab. 2004; 8(6): 1122–1123.

57	Ding Z-J, Wang G-P, Pei G-X, Zhang Y-L, Wang D-M, Hong G-Z, Du H-Y, Bai A-P, Li G. Epidemiological investigation of mental disorders among adults in Tianshui City, Gansu Province. Health Occupational Education. 2015; 33(8): 128-131.
58	Liu H-Y, Zheng F-L, Shi S-T, Shi M-Z, Ni L-X, Zhou J-J, Li L-J. Epidemiological Investigation of the Association between Serum Elements and Children with ADHD in four Ethnic Groups in Yunnan Province. Chin J Sch Dr. 2006; 20(4): 349-51.
59	Liu F, Liao L-H, Jiang Z-S. Epidemiological Investigation on ADHD Among Children aged 6-12 in Liuzhou City. Contemp Med Forum. 2014; 12(16): 179-181.
60	Du D-Y, Li M-W, Chen J-S. Epidemiological Investigation on ADHD among Children in Urban Area of Guangzhou. Guangdong Med J. 2003; 24(3): 299-300.
61	Lin Y-C, Zhuang H-C, Deng Y-T. Epidemiological Investigation on ADHD among Pre-scholars in Putian Area. Fujian Med J. 1999; 21(4): 105-7.
62	Wang Y, Liang D, Tang C-H, Zhang W-L, Zhou F-J. Epidemiological Investigation on ADHD among Students aged from 6-8 in Changchun City. Aerosp Med. 2010; 21(2): 239-40.
63	Han L-T, Han J-Y, Huang G-Y, Li J-W. Epidemiological investigation on adhd in Liaoyang city, Liaoning. Med J Chin Peoples Health. 2011; 23(7): 883-7.
64	Cheng W, Lue LQ, Qian YY, Hu YL, Hu ZJ, Ling CY, Gao YJ, Hu ZY. Epidemiological investigation on autism spectrum disorders in children aged from 1 to 6 years old in Ningbo city. Prev Med. 2016; 28(11): 1168-1171.
65	Luo W, Lin L, Chen R, Cheng W, Huang Y, Hu T, et al. Epidemiological investigation on autistic disorder in Fujian province. Shanghai Arch Psychiatry. 2000; 12(1): 3–5.
66	Zhang G-Y, Yang Y-C, Huang Y, Liu S-J, Sun X-L. Epidemiological investigation on depression among 6-16 years old children and adolescents in Chengdu. Chin J Psychiatry. 2010; 24(3): 211-214.
67	Meng Q-Z, Yu Z-G, Liu J-P, He P-H, Zhang X-P, Liu J, Ma H-Z, He P-E. Epidemiological investigation on mental disorders among rural population. Med J Chin Civ Adm. 2001; 13(1): 31-2.
68	Wei G, Liu S-M, Zhang W, Xiang Y, Huang X-Q, Yang C, Huang W-J, Xie W-J, He X, Su X-F, Wang J, Ciren P-C, Baima Z-G, Ci-P, Zha S, Liu X-H. Epidemiological investigation on mental disorders at Tibet in China: I major psychiatric disorders. Chin J Nerv Ment Dis. 2008; 34(10): 601-604.
69	Peng T, Wang W-G, Zhao Y-Y, Liu A-S. Epidemiological investigation on mental disorders in Daowai District of Harbin. Chin Ment Health J. 1999; 13(3): 158-159.
70	Zhan R-T, Lu W-X, Li S-Z, Xiang X-H, Chen W-M, Li T, Xiao J-M. Epidemiological investigation on mental disorders in Puer city. Med J Chin Peoples Health. 2011; 23(8): 955-1012.
71	Zhang J-H. Epidemiological investigation on mental disorders in Shaoxing city. Med J Chin Peoples Health. 2003; 15(5): 313-4.
72	Chen Y-Q, Shen D-Y. Epidemiological Investigation on Mental Disorders in Shaoxing County. Strait J Prev Med. 2004; 10(4): 7-9.
73	Tu Y, Zhou D-Y, Zheng W-M. Epidemiological investigation on mental disorders in Yichang area. Med J Chin Civ Adm. 1995; 7(6): 328-30.
74	Hu J-M, Li Z, Chen Y-H, Zhou X-M, M Y-H, Huang H-F, Yan H-R, Wang X-L, Guan L-Y, Wang W-B. Epidemiological investigation on mental disorders in Zhongshan city, Guangzhou Province. Chin J Nerv Ment Dis. 2002; 28(6): 456-458.
75	Cheng C-A, Ai C-Q, Cheng J-L, He J-P, Ma G-Z, He Y-M. Epidemiological investigation on mental health in Danjiangkou city in 1998. Med J Chin Civ Adm. 2001; 13(6): 334-5.
76	Sheng J-L, Bai S-Z, Zhao L, Cai D-X, Wang Y-M, Wang A-J, Wang Y-Y. Epidemiological investigation on mental illness in Xinshi area, Xinjiang Province. Med J Chin Civ Adm. 2000; 12(1): 33-5.
77	Zhao Z-S, Wu Y-T, Zhou J-Q, Zhao Y-L, Wang B-Z, Wang J-B, Li H-P, Li M-F, Zhao J-C, Shen J-X. Epidemiological Investigation on Schizophrenia in Dali Area in Yunnan Province. Mod Rehabil. 1997; 1(1): 78-9.
78	Gao L-L. Epidemiological investigation on schizophrenia in shihezi city, Xinjiang

	province. Med Innov China. 2010; 7(12): 139-40.
79	Xiong X-Z. Epidemiological investigation on severe mental disorders in Ziyang city Sichuan province. Med Innov China. 2013; 10(5): 117-9.
80	Luo X-R, Wan G-B, Su L-Y, Yang Z-W. Epidemiological study of conduct disorder (CD) of 7-16-year-olds in Hunan province, China. Chin Ment Health J. 1994; 8(5): 227-228.
81	Zhang X-G, Zhuang X-H, Chen J-F, Zhao D-Q. Epidemiological study of schizophrenia in metropolitan Shantou. J Clin Psychol Med. 1997; 7(2): 83-85.
82	Yan J, Huang Y-Q, Ruan Y, Lu J, Gao C-Q, Dang W-M, Luo C. Epidemiological survey of affective disorder in Kunming City. Chin Ment Health J. 2010; 24(2): 110-115.
83	Jiang L, Chang W-J, Su W, Liu W-H, Cao G-W. Epidemiological Survey of Attention-deficit Hyperactivity Disorder in Pupils of Urban Districts in Zhenjiang. Acad J Second Mil Med Univ. 2004; 25(11): 1238-40.
84	Ruan Y, Huang Y, Xu Y, Wu M, Yang J, Yao J, Dang W, Gao C, Luo C. Epidemiological Survey of Mental and Behavior Disorders in Kunming. Mod Prev Med. 2010; 37(4): 628-32.
85	Fang X, Chen Y, Chen C, Xiong D, Ji J, Shi G, Zheng J, Chen B, Chen T, Weng S. Epidemiological survey of mental disorder in Fujian province. Chin J Psychiatry. 2011; 44(2): 103–7.
86	Yang X-C, He C-G, Su J, Zhang Q-H, Qi W-G, Wang Q-F, Zhang J-G, Zhu C-Q, Shi W-F, Zhong M. Epidemiological survey of mental disorders among people aged 15 and over in Laiwu city of Shandong province. J Clin Psychiatry. 2017; 27(1): 56-58.
87	Ding Z-J, Wang G-P, Pei G-X, Guan R-C, He R-F, Phillips Michael R, Li X-Y, Zhang Y-L, Wang D-M, Hong G-Z, Du H-Y, Bai A-P, Li G, Wang X-Q, Wei C-L, Li H, Wang J. Epidemiological survey of mental disorders in people aged 18 and older in Tianshui City of Gansu Province. Chin Ment Health J. 2010; 24(3): 183-190.
88	Wang W-Q, Ding L-J, Wen C, Liao Z-H, Hong X, Chen Y, et al. Epidemiological Survey of Mental Disorders in People aged 18 years and older in Xiamen City. Chin J Psychiatry. 2013; 46(1): 43-9.
89	Song Z-Q, Du X-B, Han G-L, Jian Y-L, Liu C, Phillips Michael R, Li X-Y, An H-S, Shen B-L, Zheng T, Liu L-X, E H-H. Epidemiological survey of mental disorders in persons aged 18 and older in Qinghai Province. Chin Ment Health J. 2010; 24(3): 168-174.
90	Zhang J-X, Lu C-H, Tang J-S, Qiu H-M, Liu L-F, Wang S-B, Wang A-Z, Zhang T-L, Phillips M-R, Li X-Y, Zhang S-D, Jiang Y-H, Zhao C-Y, Hu B-W, Cao X-Y, Zhang Y, Gao C-N. Epidemiological survey of mental disorders in persons aged 18 years and older in Shandong Province. Chin Ment Health J. 2010; 24(3): 161-167.
91	Tao L-G, Huang F, Zhou Y, Li Q-B, Liang H-M, Li G-L, Wei H-R, Zhou L-J, Feng Q-M. Epidemiological survey of mental disorders in rural in Guilin. J Clin Psychiatry. 2011; 21(6): 381-383.
92	Keqing L, Ze C, Lijun C, Qinpu J, Guang S, Haoran W, Jing H, Wuwen Z, Jianguo X, Yanping Z, Ben Z, Jianxun J, Xueyi W, Jun T, Yufu Z, Haishan H, Jianping G, Enyi Z. Epidemiological survey of mental disorders in the people aged 18 and older in Hebei Province. Asian J Psychiatr. 2008; 1(2): 51-5.
93	Yu J, Tang M. Epidemiological survey of mental disorders in urban and rural areas of Guangzhou [Master's thesis]. Guangzhou, China: Guangzhou Medical College, 2010.
94	Pan G-W, Jiang C, Yang C-L, Na J, Fu Z-G, Yu F-Q, Yu C-Y, Liu M-J, Liu H, Wang P, Li N, Yan T-H, Zhang S-J. Epidemiological survey of mental disorders in urban and rural areas of Liaoning province. Chin J Public Health. 2006; 22(12): 1505-1507.
95	Dong A-L, Zhang G, Wang S-S, Sun J-G, Zhang L-X, Zhang C-H, Gong Z-Y, Tang S-J, Bi J-H, Gu D-H, Zhang X-R, Niu N, Mu J-M, Cui S-K. Epidemiological survey of mental disorders in Weihai. J Clin Psychiatry. 2008; 18(4): 241-243.
96	Pang S-T, Wang G-J, Kong L-L, Zhang Y-D, Sun B, Yin S, Li X-Y, Phillips Michael R. Epidemiological survey of mental illnesses in persons aged 18 years and older in Qingdao City. Chin Ment Health J. 2010; 24(3): 175-182.
97	Shi Q-C, Zhang J-M, Xu F-Z et al. Epidemiological survey of mental illnesses in the people aged 15 and older in Zhejiang Province. Chin J Prev Med. 2005; 39(4): 229-236.
98	Zhang S-J, Jiang C, Wang P, Liu M-J, Liu H, Yu C-Y, Hu Z-G, Yu F-Q, Yang X-L, N J,

	Li N, Yan T-M, Pan G-W. Epidemiological Survey of Mood Disorders Among Urban and Rural Residents in Liaoning Province. Chin J Prev Control Chronic Dis. 2008; 16(4): 378-81.
99	Tao L, Huang F, Zhou Y, Li Q, Liang H, Li G, Wei H, Zhou L, Feng Q. Epidemiological survey of schizophrenia in urban and rural in Guilin, China. J Guangxi Med Univ. 2011; 6.
100	Liu D-M, Yang Y-H, Wang Z-Q, Xiang D-H, Wang S-Y, Yang C-M, Li H-M, Lin J. Epidemiological survey of severe depression among adults in Yibin city. Chin Prev Med. 2012; 13(7): 507-510.
101	Sun X-L, Li K-Q, Cui L-J, Jiang Q-P, Gao L-H, Liu Y-Q, Han Y-C, Yang L-H, Li J-F, Yan B-P, Zhang Y, Lv H. Epidemiological Survey on Anxiety Disorder in of Hebei Province. Cap Med. 2009; 7: 37-9.
102	Chen Y, Huang H, Zhao Y, Li D, Li J, Rao L, Zhu M, Ren X. Epidemiological survey on autism spectrum disorders in 2 to 6 years old children in Ranghulu district in Daqing city. China J Child Health Care. 2010; 18(4): 331–333.
103	Duan C-F, Xu X-Q, Yuan H-H, Mo F, Zhang X-S. Epidemiological survey on mental disorders in 5 jurisdictional streets in central urban of Chongqing city. Sichuan Ment Health. 1989; 2(1): 1-5.
104	Tang W, Liu Q-J, Wen X-T, Zhang C-Y, Yan X-L, Zhang B-Q, Sha B, Ye T-M, Lin C-C, Tu X-D, Chen D-S, Chen G-D, Ye J-C, Sun H-B. Epidemiological survey on mental disorders in Wenzhou area. Shanghai Arch Psychiatry. 2005; 17(4): 197-9.
105	Xiaoyong L, Helong C, Bin H. Epidemiological survey on prevalence of schizophrenia in Jiangxi province. Shanghai Arch Psychiatry. 2004; 16(4): 014.
106	Chen C, Shen Y, Zhang W. Epidemiological survey on schizophrenia in 7 areas of China. Chin J Psychiatry. 1998; 31(2): 72-4.
107	Zhang S-Q. Epidemiology Investigation of Mental Health in Hangzhou City. Zhejiang Med. 1990; 12(2): 9-10.
108	Chen Z-S, Zhu W-F, Hu G-T, Qi B-Z, Zhu Q-K, Dong G. Epidemiology of psychiatric disease in residents in Qiaoxia community in Yongjia county, Zhejiang Province. Dis Surveillance. 2012; 27(2): 131-133.
109	Wang D, Ma J, Tan L, Chen Y, Li X, Tian X, Zhou X, Liu X. Epidemiology of severe mental illness in Hunan province in central China during 2014-2015: A multistage cross-sectional study. PLoS One. 2017; 12(11).
110	Qu Z-W, Yang Z-D, Jiang Q, Yuan J, Qin J, Tang Q, Lu Y, Sun Y-Y, Xi Y, Min J, Liu Y-J, Lu W, Qin H-Y, Pei Y, Wang H-J. Epidemological Characteristics of Anxiety Disorders in Elderly in Pudong New District, Shanghai: A Community-based Study. Chin J Clin (Electron Edit). 2011; 5(5): 1346-50.
111	Zhang A, Li J, Zhang Y, Jin X, Ma J. Epilepsy and Autism Spectrum Disorder: An Epidemiological Study in Shanghai, China. Front Psychiatry. 2019; 10: 658.
112	Sun X, Allison C, Matthews FE, Zhang Z, Auyeung B, Baron-Cohen S, Brayne C. Exploring the Underdiagnosis and Prevalence of Autism Spectrum Conditions in Beijing. Autism Res. 2015; 8(3): 250–60.
113	Chen C-Y, Liu C-Y, Su W-C, Huang S-L, Lin K-M. Factors associated with the diagnosis of neurodevelopmental disorders: a population-based longitudinal study. Pediatrics. 2007; 119(2): e435-443.
114	Ran M-S, Mao W-J, Chan Cecilia L-W, Chen Eric Y-H, Conwell Y. Gender differences in outcomes in people with schizophrenia in rural China: 14-year follow-up study. Br J Psychiatry. 2015; 206(4): 283-288.
115	Zhu X-X, Li C-L, Meng G-R, Zhang Y-F, Zhou G-F, Chen Q-M. Genetic investigation on schizophrenia. J Clin Psychol Med. 1992; 2(4): 193-195.
116	Sun X-R, Qu Z-W, Jiang Q, Yuan J. Min J, Qin H-Y. Investigation into the epidemiology of severe depression in a commmunity in Pudong New area Shanghai Province. Med J Chin Peoples Health. 2011; 23(9): 1078-80.
117	Kulibahan, Li D-M, Yeerken, Ruo M, Panggejiapu. Investigation of attention deficit hyperactivity disorder among the ethnic Han and Kazakh students from Kuntun City of Xinjiang. Chin J Contemp Pediatr. 2005; 7(4): 366-368.

118	Deng QW, Chen YG, Huang QS, Liao B, Wang ML. Investigation of Autism Spectrum Disorders for Preschool Children in Hengyang. Chin Prim Health Care. 2014; 28(3): 92-4.
119	Wu X, Lu Y, Wang Y, Zheng Q, Wang T, Lin J, Chen J. Investigation of childhood autism status in Lianyungang city. J Mod Med Health. 2010; 26: 3724–3726.
120	Li Y, Huang M-X, Yu Q, Zhao B. Investigation of Children and Adolescents with ADHD Combined with Conduct Disorder. J Int Psychiatry. 2015; 42(5): 32-5.
121	Xu G-L,Wang C-H,Yang G-F,Xu G-M,Zhen L. Investigation on children's attention and disruptive behavior disorder in the town of Cangtai, Henan province. J Clin Psychiatry. 2011; 21(4): 255-257.
122	Su L-Y, Luo X-R, Yang Z-W, Wan G-B, Li X-R. Investigation on prevalence of mental disorders and their influential factors in 13-16-year-old adolescents in Hunan province. Chin Ment Health J. 1993; 7(5): 221-224.
123	Lu J, Ruan Y, Huang Y, Yao J, Dang W, Gao C. Major depression in Kunming: prevalence, correlates and co-morbidity in a south-western city of China. J Affect Disord. 2008; 111(2-3): 221-6.
124	Chen W-H, Lu S-W, Ma Q, Zhuang Y-G, Lin J-G, Luo W-W. Mental disease epidemiology research in 5 areas of Fujian I, research methods and data analysis. Fujian Med J. 1988; 10(8): 45-46.
125	Ran M-S, Chen EY-H, Conwell Y, Chan CL-W, Yip PS, Xiang M-Z, Caine ED. Mortality in people with schizophrenia in rural China 10-year cohort study. Br J Psychiatry. 2007; 190(3): 237–42.
126	Jin W, Du Y, Zhong X, David C. Prevalence and contributing factors to attention deficit hyperactivity disorder: a study of five- to fifteen-year-old children in Zhabei District, Shanghai. Asia Pac Psychiatr. 2014; 6(4): 397–404.
127	Merikangas KR, Jin R, He J-P, Kessler RC, Lee S, Sampson NA, Viana MC, Andrade LH, Hu C, Karam EG, Ladea M, Medina-Mora ME, Ono Y, Posada-Villa J, Sagar R, Wells JE, Zarkov Z. Prevalence and correlates of bipolar spectrum disorder in the World Mental Health Survey Initiative. Arch Gen Psychiatry. 2011; 68(3): 241-51.
128	Ran M-S, Xiang M-Z, Li S-X, Shan Y-H, Huang M-S, Li S-G, Liu Z-R, Chen EY-H, Chan CL-W. Prevalence and course of schizophrenia in a Chinese rural area. Aust N Z J Psychiatry. 2003; 37(4): 452-7.
129	HUANG JP, CUI SS, HAN Y, IRVA H-P, QI LH, ZHANG X. Prevalence and Early Signs of Autism Spectrum Disorder (ASD) among 18–36 Month Old Children in Tianjin of China. Biomed Environ Sci. 2014; 27(6): 453–61.
130	Jin W-L, Ayinuer W-M-E, Du Y-S, Zhong X-Y, David Coghill. Prevalence and infludential factors of ADHD in children aged 5-15 years old in Zhabei district in Shanghai. Shanghai Arch Psychiatry. 2010; 22(4): 211-216.
131	Tian J, Wang L, Zhang X. Prevalence and Influencing Factors of Autism Spectrum Disorders in Public Primary School. Chin J Clin Psychol. 2016; 24(2): 277–81.
132	Yang J, Kang C, Zeng Y, Li J, Li P, Wan W, Zhao X, Guo W, Xu X, Yang X. Prevalence and prognosis of schizophrenia in Jinuo people in China: A prospective 30-year follow-up study. Int J Soc Psychiatry. 2014; 60(5): 482–8.
133	Wong CKM, Liang J, Chan ML, Chan YH, Chan L, Wan KY, Ng MS, Chan DCC, Wong SYS, Wong MCS. Prevalence and psychosocial correlates of depressive symptoms in urban Chinese women during midlife. PLoS One. 2014; 9(11): e110877.
134	Su Y.Y., Zhang X., Li A.Y., Li Y.M., Liu G.M. Prevalence and risk factors of infantile autism in Tianjin. Matern Child Health Care China. 2011; 26: 5004-5007.
135	Ma X, Xiang Y-T, Cai Z-J, Li S-R, Xiang Y-Q, Guo H-L, Hou Y-Z, Li Z-B, Li Z-J, Tao Y-F, Dang W-M, Wu X-M, Deng J, Wang C-Y, Lai Kelly Y-C, Ungvari Gabor S. Prevalence and Socio-demographic Correlates of Major Depressive Episode in Rural and Urban Areas of Beijing. J Affect Disord. 2009; 115(3): 323-33.
136	Yang S, Hu Y, Han Y. Prevalence investigation of autism in children. J Appl Clin Pediatr. 2007; 22(24): 1872–1873.
137	Prina A-M, Ferri C-P, Guerra M, Brayne C, Prince M. Prevalence of anxiety and its correlates among older adults in Latin America, India and China: cross-cultural study. Br

	J Psychiatry. 2011; 199(5): 485-491.
138	Fung Ada W-T, Chan W-C, Wong Corine S-M, Chen Eric Y-H, Ng Roger M-K, Lee Edwin H-M, Chang W-C, Hung S-F, Cheung Eric F-C, Sham P-C, Chiu Helen F-K, Lam M, Chiang T-P, Van Os J, Lau Joseph T-F, Lewis Glyn, Bebbington Paul, Lam Linda C-W. Prevalence of anxiety disorders in community dwelling older adults in Hong Kong. Int Psychogeriatr. 2017; 29(2): 259-267.
139	Wang S-Y, Ye X-H, Chen H, Ou G-S, Meng N-N, Mu J, Zhang Q. Prevalence of Attention Deficit Hypperactivity Disorder and its Related Factors among 6-13 years old School Children in Lanzhou City. Pract Clin Med. 2015; 16(5): 90-5.
140	Pu Y, Zhang J, Luo Y, Xie X, Wu P, Zhang Q, Yang QY. Prevalence of autism among young children aged 2-6 years in Yunyan district of Guiyang city. Chin J Public Health. 2014; 30(1): 89–91.
141	Zhou H, Xu X, Yan W, Zou X, Wu L, Luo X, Li T, Huang Y, Guan H, Chen X, Mao M, Xia K, Zhang L, Li E, Ge X, Zhang L, Li C, Zhang X, Zhou Y, Ding D, Shih A, Fombonne E, Zheng Y, Han J, Sun Z, Jiang YH, Wang Y, LATENT-NHC Study Team. Prevalence of Autism Spectrum Disorder in China: A Nationwide Multi-center Population-based Study Among Children Aged 6 to 12 Years. Neurosci Bull. 2020; 36(9): 961-71.
142	Wang X, Yang W-H, Jin Y, Jing J, Huang X, Li X-H, Wei W, Bao P, Wang H, Hu M, Li S-Y, Xiu L-J, Guo J, Liang H-N, Ning J, Cheng S-Y, Fan Y-B. Prevalence of autism spectrum disorders in preschool children of Guangzhou kindergartens. Chin Ment Health J. 2011; 25(6): 401–408.
143	Tang M-N, Liu X-H, Han H-Y, Tang M-M, Wang Y-F, Zhang L-X. Prevalence of depressive disorders among residents aged 55 or above in Chengdu area. Chin Ment Health J. 2001; 15(2): 103-106.
144	Jin Z, Yang Y, Liu S, Huang H, Jin X. Prevalence of DSM-5 Autism Spectrum Disorder Among School-Based Children Aged 3-12Years in Shanghai, China. J Autism Dev Disord. 2018; 48(7): 2434-2443.
145	Leung P, Hung S, Ho T, Lee C, Liu W, Tang C, Kwong S. Prevalence of DSM-IV disorders in Chinese adolescents and the effects of an impairment criterion: a pilot community study in Hong Kong. Eur Child Adolesc Psychiatry. 2008; 17(7): 452-61.
146	Tao L-G, Huang F, Zhou Y, Li Q-B, Liang H-M, Li Guang L, Wei H-R, Zhou L-J, Feng Q-M. Prevalence of major depression disorder among urban and rural residents in Guilin municipality, China. Chin J Public Health. 2012; 28(10): 1268-1271.
147	Liu J, Yan F, Ma X, Guo H-L, Tang Y-L, Rakofsky JJ, Wu X-M, Li X-Q, Zhu H, Guo X-B, Yang Y, Li P, Cao X-D, Li H-Y, Li Z-B, Wang P, Xu Q-Y. Prevalence of major depressive disorder and socio-demographic correlates: Results of a representative household epidemiological survey in Beijing, China. J Affect Disord. 2015; 179: 74–81.
148	Wei B, Chen Q, Pan R-D, Feng Q-M, Chen Q-M, Huang G-G, Luo H-Y, Su L, Tang Z-H, Tang H-N, Chen N-S, Chen F-Q, Li H-J. Prevalence of major depressive disorder in urban and rural residents of Guangxi Zhuang Autonomous Region, China. Chin J Public Health. 2011; 27(4): 399-401.
149	Xu G, Chen G, Zhou Q, Li N, Zheng X. Prevalence of Mental Disorders among Older Chinese People in Tianjin City. Can J Psychiatry. 2017; 62(11): 778-786.
150	Qu Y, Jiang H, Zhang N, Wang D, Guo L. Prevalence of Mental Disorders in 6–16-Year-Old Students in Sichuan Province, China. Int J Environ Res Public Health. 2015; 12(5): 5090–107.
151	Huang Y, Wang Y, Wang H, Liu Z, Yu X, Yan J, Yu Y, Kou C, Xu X, Lu J, Wang Z, He S, Xu Y, He Y, Li T, Guo W, Tian H, Xu G, Xu X, Ma Y, Wang L, Wang L, Yan Y, Wang B, Xiao S, Zhou L, Li L, Tan L, Zhang T, Ma C, Li Q, Ding H, Geng H, Jia F, Shi J, Wang S, Zhang N, Du X, Du X, Wu Y. Prevalence of mental disorders in China: a cross-sectional epidemiological study. Lancet Psychiatry. 2019; 6(3): 211-24.
152	Wang Z, Wang L, Jing J, Hu C. Prevalence of mental disorders in migrants compared with original residents and local residents in Ningxia, China. BMC Psychiatry. 2016; 16.
153	Ma Z-Y, Feng Q-M, Chen Q, Pan Y-D, Luo H-Y, Su L, Chen F-Q, Li H-J, Wei B. Prevalence of mood disorders among residents in Guangxi Zhuang Autonomous region. Chin J Public Health. 2012; 28(2): 1565-1568.

154	Xiaoli Y, Chao J, Wen P, Wenming X, Fang L, Ning L, Huijuan M, Jun N, Ming L, Xiaoxia A, Chuanyou Y, Zenguo F, Lili L, Lianzheng Y, Lijuan T, Guowei P. Prevalence of psychiatric disorders among children and adolescents in northeast China. PLoS One. 2014; 9(10): e111223.
155	Wei B, Chen Q, Feng Q. Prevalence of schizophrenia in residents of Guangxi Zhuang Autonomous Region. Chin J Public Health. 2011; 1245–7.
156	Cui L, Li K, Cui Z, Jiang Q, Gao L, Zhang Y, Li J. Prevalence, demographic characteristics and function status of the schizophrenia in Hebei province. Chin J Nerv Ment Dis. 2007; 33(3).
157	Lam LC-W, Wong CS-M, Wang M-J, Chan W-C, Chen EY-H, Ng RM-K, Hung S-F, Cheung EF-C, Sham P-C, Chiu HF-K, Lam M, Chang W-C, Lee EH-M, Chiang T-P, Lau JT-F, van Os J, Lewis G, Bebbington P. Prevalence, psychosocial correlates and service utilization of depressive and anxiety disorders in Hong Kong: the Hong Kong Mental Morbidity Survey (HKMMS). Soc Psychiatry Psychiatr Epidemiol. 2015; 50(9): 1379–88.
158	Phillips MR, Zhang J, Shi Q, Song Z, Ding Z, Pang S, Li X, Zhang Y, Wang Z. Prevalence, treatment, and associated disability of mental disorders in four provinces in China during 2001-05: an epidemiological survey. Lancet. 2009; 373(9680): 2041-53.
159	Tang Jennifer Y-M, Chang W-C, Hui Christy L-M, Wong Gloria H-Y, Chan Sherry K-W, Lee Edwin H-M, Yeung W-S, Wong C-K, Tang W-N, Chan W-F, Pang Edwin P-F, Tso Steve, Ng Roger M-K, Hung S-F, Dunn Eva L-W, Sham P-C, Chen Eric Y-H. Prospective relationship between duration of untreated psychosis and 13-year clinical outcome: A first-episode psychosis study. Schizophr Res. 2014; 153(1-3): 1-8.
160	Chen H-M, Guo H, Pan H-J, Zou Y-J, Luo A-J, Zhu H-Y, Liu X-J, Sun X-L. Qinhuangdao Epidemiological Survey of 18 Years of Age and People Over Mental Illness. Shanxi Med J. 2012; 41(21): 1191-4.
161	Harrison G, Hopper K, Craig T, Laska E, Siegel C, Wanderling J, Dube KC, Ganev K, Giel R, an der Heiden W, Holmberg SK, Janca A, Lee PW, León CA, Malhotra S, Marsella AJ, Nakane Y, Sartorius N, Shen Y, Skoda C, Thara R, Tsirkin SJ, Varma VK, Walsh D, Wiersma D. Recovery from psychotic illness: a 15- and 25-year international follow-up study. Br J Psychiatry. 2001; 178: 506-17.
162	Chen Q, Huang LX, Xu WJ, Chen H, Zhong JQ, Zeng CZ. Research on the prevalence and the risk factors of autism sepctrum disorder from 1.5 to 3 years old in Zhuhai city. Chin J Child Health Care. 2014; 22(6): 649-651.
163	Xie Z-H, Bo S-Y, Zhang X-T, Liu M, Zhang Z-X, Yang X-L, Ji S-R, Yan H, Sui X-L, Na X, Guo S-H, Wu Z-L. Sampling survey on intellectual disability in 0 approximately 6-year-old children in China. J Intellect Disabil Res. 2008; 52(12): 1029-38.
164	Liu T, Zhang L, Pang L, Li N, Chen G, Zheng X. Schizophrenia-related disability in China: prevalence, gender, and geographic location. Psychiatr Serv. 2015; 66(3): 249–57.
165	Lu N-W, Zhou G-Y. Status analysis of patients with schizophrenia in Nantong economic development zone. Med J Chin Peoples Health. 2014; 26(24): 92-93+96.
166	Han H-Y, Zhang H-Q, Zhang H-M, Gao X-N, Zhang W. Study on the correlation between the incidence of anxiety disorder and family factors in Shijiazhuang. Chin J Practical Nerv Dis. 2016; 19(3): 8-10.
167	Hu Y-Z, Wu H-R, Yu J-Q. Subtyping and Inquiring for Ethology of Children ADHD Aged 6 to 12 Years. Chin J Sch Dr. 1998; 12(5): 321-4.
168	Zhang B-C, Zhu Y, Deng B, Wen Q-S, Sun Y, Wang X-R. Survey of ADHD among primary and middle school students in urban area of Guiyang city. Matern Child Health Care China. 2011; 26: 4892-4894.
169	Yu C, Xia W, Sun A, Zhou X, Liu L, Li J, Zhou X, Wu L. Survey on autistic spectrum disorders in 2 to 6 years old children in Harbin city. Chin J Child Health Care. 2010; 18: 750-753.
170	Cheng Z-R, Gao H, Zhang X, Tang Z-R, Lu Y-W, Yang W-Q, et al. Survey on mental disorder epidemiology and mental health service situation in Shenzhen city. Med J Chin Civ Adm. 1999; 11(1): 32-5.
171	Hu J-Z, Hu C-Y, Duan W-D, Gao H, Zhang X, Tang Z-R, Lu Y-W, Zhang F-X, Jin D,

	Yang K-J, Lin X-B, Yang H, Shu M-Y, Zhang Y-H, Liu T-B, Shen Q-J. Survey on Mental Disorders among Registered Residents and Non-registered Residents in Shenzhen. Chin J Epidemiol. 2009; 30(6): 543-8.
172	Liu J, Yang X, Jia M, Qu C, Shi J, Liu G, et al. Survey on pervasive developmental disorder in 2-6 year-old children in Beijing. Chin Ment Health J. 2007; 21(5): 290–293.
173	Mo X-M, Oyang W-P, Chen J-R. Survey on Prevalence of Psychopathy in Hualin Street, Liwan District, Guangzhou. South China J Prev Med. 2003; 29(1): 48-9.
174	Ge X-L, Zhou J-L. Survey on schizophrenia among people over 15 years old in Changsha. J Clin Psychiatry. 2014; 24(5): 292-295.
175	Chang W-C, Hui Christy L-M, Wong Gloria H-Y, Chan Sherry K-W, Lee Edwin H-M, Chen Eric Y-H. Symptomatic remission and cognitive impairment in first-episode schizophrenia: A prospective 3-year follow-up study. J Clin Psychiatry. 2013; 74(11): 1046-1053.
176	Fang Y. The Analysis of the Result of the Census of Mental Disorders in Fenghua City. Mod Pract Med. 2008; 20(10): 816-20.
177	Xiao S, Lewis M, Mellor D, McCabe M, Byrne L, Wang T, Wang J, Zhu M, Cheng Y, Yang C, Dong S. The China longitudinal ageing study: overview of the demographic, psychosocial and cognitive data of the Shanghai sample. J Ment Health. 2016; 25(2): 131-6.
178	Zhu Y-Z, Jia Y, Liu R, Liu Z-F, Wang X. The Condition of Mental Disorders and the Analysis of the Recovery Management in the Community. Guide Chin Med. 2012; 10(24): 452-4.
179	Leung PW, Luk SL, Ho TP, Taylor E, Mak FL, Bacon-Shone J. The diagnosis and prevalence of hyperactivity in Chinese schoolboys. Br J Psychiatry. 1996; 168(4): 486-96.
180	Ye H-S, Luo X-R, Zhou X, Shen X-Y, Wang X-H. The Epidemiological Investigation of Anxiety Disorders in Urban-rural Primary and Middle School Students in Huaihua. J Clin Psychosom Dis. 2008; 14(6): 501-4.
181	Wei B-X, Hu X-L, Chen J-M. The epidemiological investigation on mental disorders in Mianyang city. Sichuan Ment Health. 1994; 7(1): 50-1.
182	Chen C-Z. The epidemiological investigation on mental disorders in Shandong province. Shandong Psychiatry. 1989; 2: 1-6.
183	Yuan J, Qu, Z-F, Jiang Q, Yang Z-D, Bo W-Z. The epidemiological survey of depression disorder and cognitive disorder of the elderly in Shanghai Pudong community. J Clin Psychiatry. 2013; 23(3): 86-88.
184	Lee S, Tsang A, Huang Y-Q, He Y-L, Liu ZR, Zhang M-Y, Shen Y-C, Kessler RC. The epidemiology of depression in metropolitan China. Psychol Med. 2009; 39(5): 735-47.
185	Lee S, Chan YYL, Hsu LKG. The intermediate-term outcome of Chinese patients with anorexia nervosa in Hong Kong. Am J Psychiatry. 2003; 160(5): 967-72.
186	Tang W-B, Yan P-Q, Ding Z-P, Zhang X, Hou J-Q, Mao X-W. The investigation of prevalence on ADHD among elementary school students in Hexi area in Tianjin Province. Chin J Public Health. 1993; 9(2): 90.
187	Zhang F, Sui Q, Wang J, Xue S, Lu X, Ma X, et al. The latest investigation of autism epidemic of children aged from 1 to 6 years old in Wuxi city. Matern Child Health Care China. 2008; 23(27): 3878–3880.
188	Lau JTF, Kim Y, Wu AMS, Wang Z, Huang B, Mo PKH. The Occupy Central (Umbrella) movement and mental health distress in the Hong Kong general public: political movements and concerns as potential structural risk factors of population mental health. Soc Psychiatry Psychiatr Epidemiol. 2017; 52(5): 525-536.
189	Zhou X, Bi B, Zheng L, Li Z, Yang H, Song H, Sun Y. The prevalence and risk factors for depression symptoms in a rural Chinese sample population. PLoS One. 2014; 9(6): e99692.
190	Wang X-L, Yang M-F, Yang H, Jiang R-F, Liu Y-D, Bai S-F, Lin Y-Z, Wu L-Z, Liu A-C, Lv M-Y. The Prevalence of ADHD Among Pupils in Xiamen. Chin J Sch Health. 2007; 28(11): 995-6.
191	Zhang W, Tang M-N, Qiu C-J. Han H-Y, Dai L. Lu J, Wu S, Wang S-H, Chen J-M, Guo

	L-J. Ding Y-Q, Li S-X, Liu X-H. The Prevalence of Depressive Disorder among Residents aged 55 or over in Chengdu Area. Chin J Geriatr. 2004; 23(12): 883-5.
192	Qiu P, Caine E-D, Hou F, Cerulli C, Wittink M-N, Li J. The Prevalence of Distress and Depression among Women in Rural Sichuan Province. PLoS One. 2016; 11(8): e0161099.
193	Liu Z, Huang Y, Chen X, Cheng H, Luo X. The prevalence of mood disorder, anxiety disorder and substance use disorder in community residents in Beijing: A cross-sectional study. Chin Ment Health J. 2013; 27(2): 102-10.
194	Shen YM, Chan BSM, Liu JB, Zhou YY, Cui XL, He YQ, Fang YM, Xiang YT, Luo XR. The prevalence of psychiatric disorders among students aged 6~16years old in central Hunan, China. BMC Psychiatry. 2018; 18(1): 243.
195	Yin H, Xu G, Tian H, Yang G, Wardenaar KJ, Schoevers RA. The prevalence, age-of-onset and the correlates of DSM-IV psychiatric disorders in the Tianjin Mental Health Survey (TJMHS). Psychol Med. 2018; 48(3): 473-487.
196	Deng QW. The Research of Prevalence and Correlative Factors of Autism Disorders Among Preschool Children in Hengyang [dissertation]. Hengyang, China: Nanhua Univeristy, 2014.
197	Wen H, Wang D, Wang W, Yang X-M, Peng R, Yao F-Q. The State of Death and the Cause of Death in Severe Psychotic Patients who were Managed in Sichuan, 2011-2013. Sichuan Ment Health. 2014; 4: 337-40.
198	Shen YC ZM, Huang YQ, He YL, Liu ZR, Cheng H, Tsang A, Lee S, Kessler RC. Twelve-month prevalence, severity, and unmet need for treatment of mental disorders in metropolitan China. Psychol Med. 2006; 36(2): 257-67.
199	Simon GE, Goldberg DP, Von Korff M, Ustün TB. Understanding cross-national differences in depression prevalence. Psychol Med. 2002; 32(4): 585-94.
200	World Health Organization (WHO). WHO World Mental Health Surveys: Global Perspectives on the Epidemiology of Mental Disorders. Cambridge, United Kingdom: Cambridge University Press, 2008.
201	Zhou L, Shi J. [A clinical diagnostic analysis of infant and child autism]. China Mod Dr. 2012; 50(17): 50-51.
202	Guo C. [An epidemiological survey of psychiatric disability among children 2-6 years of age in Dingxi county]. Chin J Rehabil Theory Pract. 2004; 10(2): 118–119.
203	Yu Z, Gu Y, Xiao S, Hu M, Zhou L. [Association between loneliness and risks of depressive episode among rural older people]. J Cent South Univ Med Sci. 2017; 42(3): 298-302.
204	Li L, Li XL, Wu WX, Cai XF, Fan XL, Wei XH, Sun TT. [Cross-sectional survey of autism spectrum disorders in children aged 0-6 years in Hainan province]. Chin J Epidemiol. 2017; 38(9): 1187-90.
205	Jiang L, Li G, Hao L, Guo R, Yang C, Du Y. [Epidemiological investigation on autism spectrum disorders among preschool children in Shanghai]. Chin J Epidemiol. 2015; 36(12): 1365–8.
206	Guan B-Q, Luo X-R, Deng Y-L, Wei Z, Ye H-S, Yuan X-H, Ning Z-J, Yang W, Ding J. [Prevalence of psychiatric disorders in primary and middle school students in Hunan Province]. Chin J Contemp Pediatr. 2010; 12(2): 123-7.
207	Gau SSF, Chong MY, Chen THH, Cheng ATA. A 3-year panel study of mental disorders among adolescents in Taiwan. Am J Psychiatry. 2005; 162(7): 1344-50.
208	Tsai SJ, Chang WH, Cheng CM, Liang CS, Bai YM, Hsu JW, Huang KL, Su TP, Chen TJ, Chen MH. All-cause mortality and suicide mortality in autistic individuals: An entire population longitudinal study in Taiwan. Autism. 2023; 13623613231167287.
209	Hou JW, Wang TR, Chuang SM. An epidemiological and aetiological study of children with intellectual disability in Taiwan. J Intellect Disabil Res. 1998; 42 (Pt 2): 137-43.
210	Yang CF, Yang CC, Wang IJ. Association between allergic diseases, allergic sensitization and attention-deficit/hyperactivity disorder in children: A large-scale, population-based study. J Chin Med Assoc. 2018; 81(3): 277-283.
211	Chen YY, Chen YL, Gau SS. Attention-deficit hyperactivity disorder and suicidality: The mediating effects of psychiatric comorbidities and family function. J Affect Disord.

	2019; 242: 96-104.
212	Chong MY, Tsang HY, Chen CS, Tang TC, Chen CC, Yeh TL, Lee YH, Lo HY. Community study of depression in old age in Taiwan: prevalence, life events and socio-demographic correlates. Br J Psychiatry. 2001; 178(1): 29-35.
213	Tsai KW, Lin SC, Koo M. Correlates of depressive symptoms in late middle-aged Taiwanese women: findings from the 2009 Taiwan National Health Interview Survey. BMC Womens Health. 2017; 17(103).
214	Lai D-C, Tseng Y-C, Hou Y-M, Guo H-R. Gender and geographic differences in the prevalence of intellectual disability in children: analysis of data from the national disability registry of Taiwan. Res Dev Disabil. 2012; 33(6): 2301-7.
215	Lin CE, Chen LF, Chou PH, Chung CH. Increased prevalence and risk of anxiety disorders in adults with tinnitus: A population-based study in Taiwan. Gen Hosp Psychiatry. 2018; 50: 131-136.
216	Hwu HG, Chang IH, Yeh EK, Chang CJ, Yeh LL. Major depressive disorder in Taiwan defined by the Chinese diagnostic Interview Schedule. J Nerv Ment Dis. 1996; 184(8): 497-502.
217	Wu C-S, Yu S-H, Lee C-Y, Tseng H-Y, Chiu Y-F, Hsiung CA. Prevalence of and risk factors for minor and major depression among community-dwelling older adults in Taiwan. Int Psychogeriatr. 2017; 29(7): 1113–21.
218	Chen YL, Chen WJ, Lin KC, Shen LJ, Gau SS. Prevalence of DSM-5 mental disorders in a nationally representative sample of children in Taiwan: methodology and main findings. Epidemiol Psychiatr Sci. 2019; 29: e15.
219	Hwu HG, Yeh EK, Chang LY. Prevalence of psychiatric disorders in Taiwan defined by the Chinese Diagnostic Interview Schedule. Acta Psychiatr Scand. 1989; 79(2): 136-47.
220	Pan YJ, Yeh LL, Chan HY, Chang CK. Transformation of excess mortality in people with schizophrenia and bipolar disorder in Taiwan. Psychol Med. 2017; 47(14): 2483-2493.
221	Yang H-J, Soong W-T, Kuo P-H, Chang H-L, Chen WJ. Using the CES-D in a two-phase survey for depressive disorders among nonreferred adolescents in Taipei: a stratum-specific likelihood ratio analysis. J Affect Disord. 2004; 82(3): 419-30.

Studies from 34 province units in China used in causes of death for Mental disorders

1	Chinese Center for Disease Control and Prevention (CCDC). China Disease Surveillance Points 2004 - China CDC.
2	Chinese Center for Disease Control and Prevention (CCDC). China Disease Surveillance Points 2005 - China CDC.
3	Chinese Center for Disease Control and Prevention (CCDC). China Disease Surveillance Points 2006 - China CDC.
4	Chinese Center for Disease Control and Prevention (CCDC). China Disease Surveillance Points 2007 - China CDC.
5	Chinese Center for Disease Control and Prevention (CCDC). China Disease Surveillance Points 2013 - China CDC.
6	Chinese Center for Disease Control and Prevention (CCDC). China Disease Surveillance Points 2014 - China CDC.
7	Chinese Center for Disease Control and Prevention (CCDC). China Disease Surveillance Points 2015 - China CDC.
8	Chinese Center for Disease Control and Prevention (CCDC). China Disease Surveillance Points 2016 - China CDC.
9	Chinese Center for Disease Control and Prevention (CCDC). China Disease Surveillance Points 2017 - China CDC.
10	Chinese Center for Disease Control and Prevention (CCDC). China Disease Surveillance Points and Death Registration 2008 - China CDC.
11	Chinese Center for Disease Control and Prevention (CCDC). China Disease Surveillance Points and Death Registration 2009 - China CDC.
12	Chinese Center for Disease Control and Prevention (CCDC). China Disease Surveillance Points and Death Registration 2010 - China CDC.

13	Chinese Center for Disease Control and Prevention (CCDC). China Disease Surveillance Points and Death Registration 2011 - China CDC.
14	Chinese Center for Disease Control and Prevention (CCDC). China Disease Surveillance Points and Death Registration 2012 - China CDC.
15	Hong Kong Vital Registration - Deaths 1994 ICD9. as it appears in WHO Mortality Database Version November 2015
16	Hong Kong Vital Registration - Deaths 1995 ICD9. as it appears in WHO Mortality Database Version November 2015
17	Hong Kong Vital Registration - Deaths 1996 ICD9. as it appears in WHO Mortality Database Version November 2015
18	Hong Kong Vital Registration - Deaths 1997 ICD9. as it appears in WHO Mortality Database Version November 2015
19	Hong Kong Vital Registration - Deaths 1998 ICD9. as it appears in WHO Mortality Database Version November 2015
20	Hong Kong Vital Registration - Deaths 1999 ICD9. as it appears in WHO Mortality Database Version November 2015
21	Hong Kong Vital Registration - Deaths 2000 ICD9. as it appears in WHO Mortality Database Version November 2015
22	Hong Kong Vital Registration - Deaths 2001 ICD10. as it appears in WHO Mortality Database Version October 2017
23	Hong Kong Vital Registration - Deaths 2002 ICD10. as it appears in WHO Mortality Database Version October 2017
24	Hong Kong Vital Registration - Deaths 2003 ICD10. as it appears in WHO Mortality Database Version October 2017
25	Hong Kong Vital Registration - Deaths 2004 ICD10. as it appears in WHO Mortality Database Version October 2017
26	Hong Kong Vital Registration - Deaths 2005 ICD10. as it appears in WHO Mortality Database Version October 2017
27	Hong Kong Vital Registration - Deaths 2006 ICD10. as it appears in WHO Mortality Database Version October 2017
28	Hong Kong Vital Registration - Deaths 2007 ICD10. as it appears in WHO Mortality Database Version October 2017
29	Hong Kong Vital Registration - Deaths 2008 ICD10. as it appears in WHO Mortality Database Version October 2017
30	Hong Kong Vital Registration - Deaths 2009 ICD10. as it appears in WHO Mortality Database Version October 2017
31	Hong Kong Vital Registration - Deaths 2010 ICD10. as it appears in WHO Mortality Database Version October 2017
32	Hong Kong Vital Registration - Deaths 2011 ICD10. as it appears in WHO Mortality Database Version October 2017
33	Hong Kong Vital Registration - Deaths 2012 ICD10. as it appears in WHO Mortality Database Version October 2017
34	Hong Kong Vital Registration - Deaths 2013 ICD10. as it appears in WHO Mortality Database Version October 2017
35	Hong Kong Vital Registration - Deaths 2014 ICD10. as it appears in WHO Mortality Database Version April 2018
36	Hong Kong Vital Registration - Deaths 2015 ICD10. as it appears in WHO Mortality Database Version April 2018
37	Hong Kong Vital Registration - Deaths 2016 ICD10. as it appears in WHO Mortality Database Version May 2019
38	Hong Kong Vital Registration - Deaths 2017 ICD10. as it appears in WHO Mortality Database Version December 2019
39	Macau Vital Registration - Deaths 1994 ICD9. as it appears in WHO Mortality Database Version November 2015
40	Ministry of Health and Welfare (Taiwan). Taiwan Statistics of Causes of Death 2017. Taipei City, Taiwan (Province of China): Ministry of Health and Welfare (Taiwan),

	2018.
41	Department of Health (Taiwan). Taiwan Vital Registration - Deaths 1980.
42	Department of Health (Taiwan). Taiwan Vital Registration - Deaths 1981.
43	Department of Health (Taiwan). Taiwan Vital Registration - Deaths 1982.
44	Department of Health (Taiwan). Taiwan Vital Registration - Deaths 1983.
45	Department of Health (Taiwan). Taiwan Vital Registration - Deaths 1984.
46	Department of Health (Taiwan). Taiwan Vital Registration - Deaths 1985.
47	Department of Health (Taiwan). Taiwan Vital Registration - Deaths 1986.
48	Department of Health (Taiwan). Taiwan Vital Registration - Deaths 1987.
49	Department of Health (Taiwan). Taiwan Vital Registration - Deaths 1988.
50	Department of Health (Taiwan). Taiwan Vital Registration - Deaths 1989.
51	Department of Health (Taiwan). Taiwan Vital Registration - Deaths 1990.
52	Department of Health (Taiwan). Taiwan Vital Registration - Deaths 1991.
53	Department of Health (Taiwan). Taiwan Vital Registration - Deaths 1992. Taipei City, Taiwan (Province of China): Ministry of Health and Welfare (Taiwan).
54	Department of Health (Taiwan). Taiwan Vital Registration - Deaths 1993. Taipei City, Taiwan (Province of China): Ministry of Health and Welfare (Taiwan).
55	Department of Health (Taiwan). Taiwan Vital Registration - Deaths 1994. Taipei City, Taiwan (Province of China): Ministry of Health and Welfare (Taiwan).
56	Department of Health (Taiwan). Taiwan Vital Registration - Deaths 1995. Taipei City, Taiwan (Province of China): Ministry of Health and Welfare (Taiwan).
57	Department of Health (Taiwan). Taiwan Vital Registration - Deaths 1996. Taipei City, Taiwan (Province of China): Ministry of Health and Welfare (Taiwan).
58	Department of Health (Taiwan). Taiwan Vital Registration - Deaths 1997. Taipei City, Taiwan (Province of China): Ministry of Health and Welfare (Taiwan).
59	Department of Health (Taiwan). Taiwan Vital Registration - Deaths 1998. Taipei City, Taiwan (Province of China): Ministry of Health and Welfare (Taiwan).
60	Department of Health (Taiwan). Taiwan Vital Registration - Deaths 1999. Taipei City, Taiwan (Province of China): Ministry of Health and Welfare (Taiwan).
61	Department of Health (Taiwan). Taiwan Vital Registration - Deaths 2000. Taipei City, Taiwan (Province of China): Ministry of Health and Welfare (Taiwan).
62	Department of Health (Taiwan). Taiwan Vital Registration - Deaths 2001. Taipei City, Taiwan (Province of China): Ministry of Health and Welfare (Taiwan).
63	Department of Health (Taiwan). Taiwan Vital Registration - Deaths 2002. Taipei City, Taiwan (Province of China): Ministry of Health and Welfare (Taiwan).
64	Department of Health (Taiwan). Taiwan Vital Registration - Deaths 2003. Taipei City, Taiwan (Province of China): Ministry of Health and Welfare (Taiwan).
65	Department of Health (Taiwan). Taiwan Vital Registration - Deaths 2004. Taipei City, Taiwan (Province of China): Ministry of Health and Welfare (Taiwan).
66	Department of Health (Taiwan). Taiwan Vital Registration - Deaths 2005. Taipei City, Taiwan (Province of China): Ministry of Health and Welfare (Taiwan).
67	Department of Health (Taiwan). Taiwan Vital Registration - Deaths 2006. Taipei City, Taiwan (Province of China): Ministry of Health and Welfare (Taiwan).
68	Department of Health (Taiwan). Taiwan Vital Registration - Deaths 2007. Taipei City, Taiwan (Province of China): Ministry of Health and Welfare (Taiwan).
69	Department of Health (Taiwan). Taiwan Vital Registration - Deaths 2008. Taipei City, Taiwan (Province of China): Ministry of Health and Welfare (Taiwan).
70	Department of Health (Taiwan). Taiwan Vital Registration - Deaths 2009. Taipei City, Taiwan (Province of China): Ministry of Health and Welfare (Taiwan).
71	Department of Health (Taiwan). Taiwan Vital Registration - Deaths 2010. Taipei City, Taiwan (Province of China): Ministry of Health and Welfare (Taiwan).
72	Department of Health (Taiwan). Taiwan Vital Registration - Deaths 2011. Taipei City, Taiwan (Province of China): Ministry of Health and Welfare (Taiwan).
73	Department of Health (Taiwan). Taiwan Vital Registration - Deaths 2012. Taipei City, Taiwan (Province of China): Ministry of Health and Welfare (Taiwan).
74	Ministry of Health and Welfare (Taiwan). Taiwan Vital Registration - Deaths 2013.

	Taipei City, Taiwan (Province of China): Ministry of Health and Welfare (Taiwan).
75	Ministry of Health and Welfare (Taiwan). Taiwan Vital Registration - Deaths 2014. Taipei City, Taiwan (Province of China): Ministry of Health and Welfare (Taiwan).
76	Ministry of Health and Welfare (Taiwan). Taiwan Vital Registration - Deaths 2015. Taipei City, Taiwan (Province of China): Ministry of Health and Welfare (Taiwan).
77	Ministry of Health and Welfare (Taiwan). Taiwan Vital Registration - Deaths 2016. Taipei City, Taiwan (Province of China): Ministry of Health and Welfare (Taiwan).

Studies from 34 province units in China used in Nonfatal Health Outcomes for self-harm

	Citation
1	Chinese Center for Disease Control and Prevention (CCDC). China Injury Comprehensive Surveillance Study 2009-2011 - China CDC.
2	Chinese Center for Disease Control and Prevention (CCDC), Ministry of Health (China). China National Injury Surveillance System 2006 - China CDC.
3	Chinese Center for Disease Control and Prevention (CCDC), Ministry of Health (China). China National Injury Surveillance System 2007 - China CDC.
4	Chinese Center for Disease Control and Prevention (CCDC), Ministry of Health (China). China National Injury Surveillance System 2008 - China CDC.
5	Chinese Center for Disease Control and Prevention (CCDC), Ministry of Health (China). China National Injury Surveillance System 2009 - China CDC.
6	Chinese Center for Disease Control and Prevention (CCDC), Ministry of Health (China). China National Injury Surveillance System 2010 - China CDC.
7	Chinese Center for Disease Control and Prevention (CCDC), Ministry of Health (China). China National Injury Surveillance System 2011 - China CDC.
8	Chinese Center for Disease Control and Prevention (CCDC), Ministry of Health (China). China National Injury Surveillance System 2012 - China CDC.
9	Chinese Center for Disease Control and Prevention (CCDC), Ministry of Health (China). China National Injury Surveillance System 2013.
10	Chinese Center for Disease Control and Prevention (CCDC), Ministry of Health (China). China National Injury Surveillance System 2014.

Studies from 34 province units in China used in causes of death for Self-harm

	Citation
1	Johns Hopkins University. 2019 Novel Coronavirus COVID-19 (2019-nCoV) Data Repository by Johns Hopkins CSSE 2020-2022. Baltimore, Maryland: Johns Hopkins University.
2	Fan XL, Zhou YP, Peng WW, Yao JL, Li GH, Guo RP. A case control study on relationship between hepatocellular carcinoma and infection of HCV and HBV in Guangzhou. Chin J Cancer. 1995; 14: 328-30.
3	Zhou YP, Peng WW, Yao JL, Lu L, Li GH, Huang JF, Peng BG, Chen Q. A case control study on relationship between hepatocellular carcinoma and infection of HCV and HCV. Acad J SUMS. 1994; 15: 45-49.
4	Zhang JY, Dai M, Wang X, Lu WQ, Li DS, Zhang MX, Wang KJ, Dai LP, Han SG, Zhou YF, Zhuang H. A case-control study of hepatitis B and C virus infection as risk factors for hepatocellular carcinoma in Henan, China. Int J Epidemiol. 1998; 27(4): 574-8.
5	Pan WS, Tian X, Tang SZ. A case–control study of HCV infection and primary liver cancer. Chin J Public Health. 1995; 11: 291-4.
6	Ding BG, Fan DM, Mu LN, Yu SZ. A case–control study of hepatitis B virus, hepatitis C virus and liver cancer. Chin J Epidemiol. 2004; 25(22).
7	Su MH, Wu JZ, Luo GH, Huang LY, Chen MW. A case–control study of relationship in HBV, HCV infection and HCC in areas with low incidences in Guangxi. Clin Foc. 2002; 17: 209-10.
8	Zuo Q, Liu YJ, Liang WT. A case–control study of the synergistic action of HBV and HCV in the development of primary hepatocellular carcinoma. Chin J Public Health. 1997; 16: 173-275.

9	Okuno H, Xie Z-C, Lu B-Y, Qin X, Takasu M, Kano H, Shiozaki Y, Inoue K. A low prevalence of anti-hepatitis C virus antibody in patients with hepatocellular carcinoma in guangxi province, southern china . Cancer. 1994; 73(1): 58-62.
10	Wu JZ, Su MH, Chen MW, Luo GH, Liang RX, Wei ZL, Jiang JN, Huang LY. A matching case–control study on the relationship between HBV, HCV infection and HCC in endemic area of HCC in Guangxi, China. J Guangxi Med Univ. 2003; 20: 313-5.
11	Liu J-B, Hong F-C, Pan P, Zhou H, Yang F, Cai Y-M, Wen L-Z, Lai Y-H, Lin L-J, Zeegers MP. A risk model for congenital syphilis in infants born to mothers with syphilis treated in gestation: a prospective cohort study. Sex Transm Infect. 2010; 86(4): 2926.
12	Dai M, Shi Y, Zhang JY, Zhou YF, Zhang MX, He Z. A study of the interaction of HBV and HCV in PHC. Henan J Oncol. 1998; 11: 81-3.
13	Wang ZF. A study of the relationship between the primary carcinoma of liver and hepatitis B, C and TTV. Henan Med Res. 2003; 12: 43-44.
14	Wang CX, Feng XH, Yyuan RZ, Li DS, Wang HJ, Cheng SQ. A study of the relationship in HBV, HCV infection and primary hepatocellular carcinoma. Chin J Health Lab Tech. 1996; 6: 184-5.
15	Ochiai RL, Acosta CJ, Danovaro-Holliday MC, Baiqing D, Bhattacharya SK, Agtini MD, Bhutta ZA, Canh DG, Ali M, Shin S, Wain J, Page A-L, Albert MJ, Farrar J, Abu-Elyazeed R, Pang T, Galindo CM, von Seidlein L, Clemens JD, Domi Typhoid Study Group. A study of typhoid fever in five Asian countries: disease burden and implications for controls. Bull World Health Organ. 2008; 86(4): 260-8.
16	Aiqiang X, Zijian F, Wenbo X, Lixia W, Wanshen G, Qing X, Haijun S, Lee LA, Xiaofeng L. Active case-based surveillance for measles in China: lessons learned from Shandong and Henan provinces. J Infect Dis. 2003; 187(Suppl 1): S258-63.
17	Cui LH, Fang JN, Jin HZ, Quan ZY, Jin CJ. An analysis of the interaction of Hepatitis virus in primary hepatocellular carcinoma in Korean of Yanbian. Chin J Prev Control Chronic Dis. 1999; 7: 28-30.
18	Wang ZJ, Zhou YP, Cheng B, Liang ZN, Peng WW. An epidemiologic study on the aetiological factors of primary liver cancer in Shunde City of Guangdong province. Chin J Epidemiol. 1996; 17(3): 141-4.
19	Wang SS, Jiang PL, Pang HX, Peng GF. An epidemiological study on the etiology of primary hepatocellular carcinoma in Guangzhou, Guangdong Province. Chin J Epidemiol. 1999; 18: 33-6.
20	Li D, Cui LH, Jin DZ, Piao XX. Analysis of serum hepatitis B virus and hepatitis C virus infection markers in patients with primary carcinoma of liver. J Med Sci Yanbian Univ. 1999; 22: 185-8.
21	Yu X, Wang S, Guan J, Mahemuti, Purhati, Gou A, Liu Q, Jin X, Ghildyal R. Analysis of the cause of increased measles incidence in Xinjiang, China in 2004. Pediatr Infect Dis J. 2007; 26(6): 513-8.
22	Gao XH, Zhang CL, Li XL, Mei H, Qi F, Zhu XY. Analysis of the infection hepatitis B and C from sera of the patients suffered from primary hepatoma. J Dalian Med Univ. 1998; 20: 19-21.
23	Climate Change and African Political Stability Project (CCAPS). Armed Conflict Location and Event Dataset, Realtime - Robert S. Strauss Center as referenced in Raleigh, Clionadh, Andrew Linke, Havard Hegre and Joakim Karlsen. 2010. Introducing ACLED-Armed Conflict Location and Event Data. Journal of Peace Research 47(5), 651-60.
24	Zhou Y, van den Hof S, Wang S, Pang Y, Zhao B, Xia H, Anthony R, Ou X, Li Q, Zheng Y, Song Y, Zhao Y, van Soolingen D. Association between genotype and drug resistance profiles of Mycobacterium tuberculosis strains circulating in China in a national drug resistance survey. PLoS One. 2017; 12(3): e0174197.
25	Peace Research Institute Oslo (PRIO). Battle Deaths Dataset Version 3.1, 2009. Oslo, Norway: Peace Research Institute Oslo (PRIO), 2009.
26	Forman D, Bray F, Brewster DH, Gombe Mbalawa C, Kohler B, Piñeros M, Steliarova-Foucher E, Swaminathan R and Ferlay J, eds (2013). Cancer Incidence in Five Continents, Vol. X Summary Database (electronic version). Lyon, IARC. http://ci5.iarc.fr
27	Chang B, Li B, Sun Y, Teng G, Huang A, Li J, Zou Z. Changes in Etiologies of Hospitalized

	Patients with Liver Cirrhosis in Beijing 302 Hospital from 2002 to 2013. Mediators Inflamm. 2017; 2017: 5605981.
28	Liu CH, Li L, Chen Z, Wang Q, Hu YL, Zhu B, Woo PCY. Characteristics and treatment outcomes of patients with MDR and XDR tuberculosis in a TB referral hospital in Beijing: a 13-year experience. PLoS One. 2011; 6(4): e19399.
29	Department of Political Science, University of Chicago. Chicago Project on Security and Threats (CPOST). 2020. Database on Suicide Attacks (October 02, 2020 Release). [Data File].
30	China - Anshan City Cancer Registry 2008-2012 - CI5.
31	China - Beijing Cancer Registry 2008-2012 - CI5.
32	China - Benxi Cancer Registry 2008-2011 - CI5.
33	China - Cixian Cancer Registry 2003-2007 - CI5. as it appears in Cancer Incidence in Five Continents Volume X Summary Database 2003-2007
34	China - Cixian County Cancer Registry 2008-2012 - CI5.
35	China - Guangzhou Cancer Registry 2010-2012 - CI5.
36	China - Guanyun Cancer Registry 2008-2012 - CI5.
37	China - Haimen County Cancer Registry 2008-2012 - CI5.
38	China - Haining County Cancer Registry 2003-2007 - CI5. as it appears in Cancer Incidence in Five Continents Volume X Summary Database 2003-2007
39	China - Harbin City, Nangang District Cancer Registry 2008-2012 - CI5.
40	China - Hefei Cancer Registry 2010-2012 - CI5.
41	China - Hengdong Cancer Registry 2009-2012 - CI5.
42	China - Hong Kong Cancer Registry 2008-2012 - CI5.
43	China - Huaiyin District, Huai'an Cancer Registry 2009-2012 - CI5.
44	China - Huangzhou City Cancer Registry 2008-2012 - CI5.
45	China - Jiangmen Cancer Registry 2010-2012 - CI5.
46	China - Jianhu County Cancer Registry 2010-2012 - CI5.
47	China - Jiashan Cancer Registry 2003-2007 - CI5. as it appears in Cancer Incidence in Five Continents Volume X Summary Database 2003-2007
48	China - Jiashan County Cancer Registry 2008-2012 - CI5.
49	China - Jiaxing City Cancer Registry 2005-2007 - CI5. as it appears in Cancer Incidence in Five Continents Volume X Summary Database 2003-2007
50	China - Jiaxing City Cancer Registry 2008-2012 - CI5.
51	China - Lianyungang Cancer Registry 2008-2012 - CI5.
52	China - Linzhou County Cancer Registry 2008-2012 - CI5.
53	China - Liuzhou Cancer Registry 2009-2012 - CI5.
54	China - Maanshan Cancer Registry 2008-2012 - CI5.
55	China - Nangang District Harbin City Cancer Registry 2003-2007 - CI5. as it appears in Cancer Incidence in Five Continents Volume X Summary Database 2003-2007
56	China - Qidong County Cancer Registry 1983-1987 - CI5. as it appears in Cancer Incidence in Five Continents Volumes I-VIII 1950-1997
57	China - Qidong County Cancer Registry 1988-1992 - CI5. as it appears in Cancer Incidence in Five Continents Volumes I-VIII 1950-1997
58	China - Qidong County Cancer Registry 1993-1997 - CI5. as it appears in Cancer Incidence in Five Continents Volumes I-VIII 1950-1997
59	China - Qidong County Cancer Registry 2003-2007 - CI5. as it appears in Cancer Incidence in Five Continents Volume X Summary Database 2003-2007
60	China - Qidong County Cancer Registry 2008-2012 - CI5.
61	China - Shanghai Cancer Registry 1975 - CI5. as it appears in Cancer Incidence in Five Continents Volume IV
62	China - Shanghai Cancer Registry 1978-1982 - CI5. as it appears in Cancer Incidence in Five Continents Volumes I-VIII 1950-1997
63	China - Shanghai Cancer Registry 1983-1987 - CI5. as it appears in Cancer Incidence in Five Continents Volumes I-VIII 1950-1997
64	China - Shanghai Cancer Registry 1988 - CI5. as it appears in Cancer Incidence in Five Continents Time Trends Annual Dataset (Summary and Detailed Databases)

65	China - Shanghai Cancer Registry 1989 - CI5. as it appears in Cancer Incidence in Five Continents Time Trends Annual Dataset (Summary and Detailed Databases)
66	China - Shanghai City Cancer Registry 2008-2012 - CI5.
67	China - Shenyang Cancer Registry 2008-2012 - CI5.
68	China - Shexian County Cancer Registry 2008-2012 - CI5.
69	China - Sheyang Cancer Registry 2008-2012 - CI5.
70	China - Tianjin Cancer Registry 1981-1982 - CI5. as it appears in Cancer Incidence in Five Continents Volumes I-VIII 1950-1997
71	China - Tianjin Cancer Registry 1983-1987 - CI5. as it appears in Cancer Incidence in Five Continents Volumes I-VIII 1950-1997
72	China - Tongling City Cancer Registry 2008-2012 - CI5.
73	China - Wuhan City Cancer Registry 2008-2012 - CI5.
74	China - Wuxi Cancer Registry 2010-2012 - CI5.
75	China - Xianju Cancer Registry 2010-2012 - CI5.
76	China - Xiping Cancer Registry 2010-2012 - CI5.
77	China - Yangcheng County Cancer Registry 2003-2007 - CI5. as it appears in Cancer Incidence in Five Continents Volume X Summary Database 2003-2007
78	China - Yanshi Cancer Registry 2010-2012 - CI5.
79	China - Yanting County Cancer Registry 2003-2007 - CI5. as it appears in Cancer Incidence in Five Continents Volume X Summary Database 2003-2007
80	China - Yanting County Cancer Registry 2008-2012 - CI5.
81	China - Yueyanglou Cancer Registry 2009-2012 - CI5.
82	China - Zhongshan City Cancer Registry 2010-2012 - CI5.
83	China - Zhuhai City Cancer Registry 2010-2012 - CI5.
84	British Broadcasting Corporation (BBC). China bus fire kills dozens in Xiamen in Fujian province. British Broadcasting Corporation (BBC) [Internet]. 2013 Jun 7; Asia-China.
85	National Central Cancer Registry (China). China Cancer Registry Annual Report 2003-2007. Beijing, China: Military Medical Sciences Press (China).
86	National Central Cancer Registry (China). China Cancer Registry Annual Report 2014. Beijing, China: Tsinghua University Press (China), 2015.
87	National Central Cancer Registry (China). China Cancer Registry Annual Report 2015. Beijing, China: Tsinghua University Press (China), 2016.
88	National Central Cancer Registry (China). China Cancer Registry Annual Report 2016. Beijing, China: Tsinghua University Press (China), 2017.
89	National Central Cancer Registry (China). China Cancer Registry Annual Report 2017. Beijing, China: Tsinghua University Press (China), 2018.
90	National Central Cancer Registry (China). China Cancer Registry Incidence and Mortality 1990-2009.
91	National Central Cancer Registry (China). China Cancer Registry Incidence and Mortality 2011.
92	Chinese Center for Disease Control and Prevention (CCDC). China Disease Surveillance Points 1991 - China CDC.
93	Chinese Center for Disease Control and Prevention (CCDC). China Disease Surveillance Points 1992 - China CDC.
94	Chinese Center for Disease Control and Prevention (CCDC). China Disease Surveillance Points 1993 - China CDC.
95	Chinese Center for Disease Control and Prevention (CCDC). China Disease Surveillance Points 1994 - China CDC.
96	Chinese Center for Disease Control and Prevention (CCDC). China Disease Surveillance Points 1995 - China CDC.
97	Chinese Center for Disease Control and Prevention (CCDC). China Disease Surveillance Points 1996 - China CDC.
98	Chinese Center for Disease Control and Prevention (CCDC). China Disease Surveillance Points 1997 - China CDC.
99	Chinese Center for Disease Control and Prevention (CCDC). China Disease Surveillance Points 1998 - China CDC.

100	Chinese Center for Disease Control and Prevention (CCDC). China Disease Surveillance Points 1999 - China CDC.
101	Chinese Center for Disease Control and Prevention (CCDC). China Disease Surveillance Points 2000 - China CDC.
102	Chinese Center for Disease Control and Prevention (CCDC). China Disease Surveillance Points 2001 - China CDC.
103	Chinese Center for Disease Control and Prevention (CCDC). China Disease Surveillance Points 2002 - China CDC.
104	Chinese Center for Disease Control and Prevention (CCDC). China Disease Surveillance Points 2004 - China CDC.
105	Chinese Center for Disease Control and Prevention (CCDC). China Disease Surveillance Points 2005 - China CDC.
106	Chinese Center for Disease Control and Prevention (CCDC). China Disease Surveillance Points 2006 - China CDC.
107	Chinese Center for Disease Control and Prevention (CCDC). China Disease Surveillance Points 2007 - China CDC.
108	Chinese Center for Disease Control and Prevention (CCDC). China Disease Surveillance Points 2013 - China CDC.
109	Chinese Center for Disease Control and Prevention (CCDC). China Disease Surveillance Points 2014 - China CDC.
110	Chinese Center for Disease Control and Prevention (CCDC). China Disease Surveillance Points 2015 - China CDC.
111	Chinese Center for Disease Control and Prevention (CCDC). China Disease Surveillance Points 2016 - China CDC.
112	Chinese Center for Disease Control and Prevention (CCDC). China Disease Surveillance Points 2017 - China CDC.
113	Chinese Center for Disease Control and Prevention (CCDC). China Disease Surveillance Points and Death Registration 2008 - China CDC.
114	Chinese Center for Disease Control and Prevention (CCDC). China Disease Surveillance Points and Death Registration 2009 - China CDC.
115	Chinese Center for Disease Control and Prevention (CCDC). China Disease Surveillance Points and Death Registration 2010 - China CDC.
116	Chinese Center for Disease Control and Prevention (CCDC). China Disease Surveillance Points and Death Registration 2011 - China CDC.
117	Chinese Center for Disease Control and Prevention (CCDC). China Disease Surveillance Points and Death Registration 2012 - China CDC.
118	Health Commission of Hubei Province (China). China Epidemic Situation of Novel Coronavirus Pneumonia in Hubei Province 2020-2022. China: Health Commission of Hubei Province (China).
119	Joint United Nations Program on HIV/AIDS (UNAIDS), United Nations Children's Fund (UNICEF), World Health Organization (WHO). China Global AIDS Response Progress Reporting (GARPR) System - Antenatal Care Attendees Positive for Syphilis.
120	Joint United Nations Program on HIV/AIDS (UNAIDS), Ministry of Health (China). China HIV/AIDS Assessment 2007.
121	Ministry of Health (China). China Maternal and Child Mortality Data 1996-2012 - China CDC.
122	Ministry of Health (China). China National Maternal and Child Health Surveillance System Child Mortality By Cause 2013 - MCHS.
123	Ministry of Health (China), National Health Commission (China). China National Maternal and Child Health Surveillance System Child Mortality By Cause 2014 - MCHS.
124	Ministry of Health (China), National Health Commission (China). China National Maternal and Child Health Surveillance System Child Mortality By Cause 2015 - MCHS.
125	Ministry of Health (China), National Health Commission (China). China National Maternal and Child Health Surveillance System Child Mortality By Cause 2016 - MCHS.
126	Ministry of Health (China), National Health Commission (China). China National Maternal and Child Health Surveillance System Child Mortality By Cause 2017 - MCHS.

127	Ministry of Health (China). China National Maternal and Child Health Surveillance System Child Mortality Data By Cause 1996-2012 - MCHS.
128	Ministry of Health (China). China National Maternal and Child Health Surveillance System Maternal Mortality By Cause 1996-2005 - MCHS.
129	Ministry of Health (China). China National Maternal and Child Health Surveillance System Maternal Mortality By Cause 2006-2012 - MCHS.
130	Ministry of Health (China). China National Maternal and Child Health Surveillance System Maternal Mortality By Cause 2013 - MCHS.
131	Ministry of Health (China), National Health Commission (China). China National Maternal and Child Health Surveillance System Maternal Mortality By Cause 2014 - MCHS. [Unpublished].
132	Ministry of Health (China), National Health Commission (China). China National Maternal and Child Health Surveillance System Maternal Mortality By Cause 2015 - MCHS.
133	Ministry of Health (China), National Health Commission (China). China National Maternal and Child Health Surveillance System Maternal Mortality By Cause 2016 - MCHS. [Unpublished].
134	Ministry of Health (China), National Health Commission (China). China National Maternal and Child Health Surveillance System Maternal Mortality By Cause 2017 - MCHS. [Unpublished].
135	Chinese Center for Disease Control and Prevention (CCDC). China Notifiable Infectious Diseases 2004.
136	Chinese Center for Disease Control and Prevention (CCDC). China Notifiable Infectious Diseases 2004-2012.
137	Chinese Center for Disease Control and Prevention (CCDC). China Notifiable Infectious Diseases 2005.
138	Chinese Center for Disease Control and Prevention (CCDC). China Notifiable Infectious Diseases 2006.
139	Chinese Center for Disease Control and Prevention (CCDC). China Notifiable Infectious Diseases 2007.
140	Chinese Center for Disease Control and Prevention (CCDC). China Notifiable Infectious Diseases 2008.
141	Chinese Center for Disease Control and Prevention (CCDC). China Notifiable Infectious Diseases 2009.
142	Chinese Center for Disease Control and Prevention (CCDC). China Notifiable Infectious Diseases 2010.
143	Chinese Center for Disease Control and Prevention (CCDC). China Notifiable Infectious Diseases 2011.
144	Chinese Center for Disease Control and Prevention (CCDC). China Notifiable Infectious Diseases 2012.
145	Chinese Center for Disease Control and Prevention (CCDC). China Notifiable Infectious Diseases 2013 - China CDC.
146	Chinese Center for Disease Control and Prevention (CCDC). China Notifiable Infectious Diseases 2014 - China CDC.
147	World Health Organization (WHO). China WHO Leishmaniasis Country Profile 2014. Geneva, Switzerland: World Health Organization (WHO), 2016.
148	Cheung T-K, Lai C-L, Wong BC-Y, Fung J, Yuen M-F. Clinical features, biochemical parameters, and virological profiles of patients with hepatocellular carcinoma in Hong Kong. Aliment Pharmacol Ther. 2006; 24(4): 573-83.
149	Sun Y, Harley D, Vally H, Sleigh A. Comparison of characteristics and mortality in multidrug resistant (MDR) and non-MDR tuberculosis patients in China. BMC Public Health. 2015; 15(1): 1027.
150	Zhu L, Qin M, Du L, Jia W, Yang Q, Walker MC, Wen SW. Comparison of maternal mortality between migrating population and permanent residents in Shanghai, China, 1996-2005. BJOG. 2009; 116(3): 401-7.
151	Hu Y, Mathema B, Zhao Q, Zheng X, Li D, Jiang W, Wang W, Xu B. Comparison of the

	socio-demographic and clinical features of pulmonary TB patients infected with sub-lineages within the W-Beijing and non-Beijing Mycobacterium tuberculosis. Tuberc (Edinb). 2016; 97: 18–25.
152	Long J, Wang Y, Li M, Tong W-M, Jia J-D, Huang J. Correlation of TP53 mutations with HCV positivity in hepatocarcinogenesis: identification of a novel TP53 microindel in hepatocellular carcinoma with HCV infection . Oncol Rep. 2013; 30(1): 119-24.
153	Cheng W, Wu Y, Wen Y, Ma Y, Zhao D, Dou Z, Zhang W, Bulterys M, Zhang F. Cotrimoxazole prophylaxis and antiretroviral therapy: an observational cohort study in China. Bull World Health Organ. 2015; 93(3): 152–60.
154	Cystic Echinococcosis Endemicity Estimates identified through systematic review and personal communication, as provided by GBD 2015 expert. [Unpublished].
155	Shen X, DeRiemer K, Yuan Z ’an, Shen M, Xia Z, Gui X, Wang L, Mei J. Deaths among tuberculosis cases in Shanghai, China: who is at risk?. BMC Infect Dis. 2009; 9(1): 95.
156	Wang B-E, Ma W-M, Sulaiman A, Noer S, Sumoharjo S, Sumarsidi D, Tandon BN, Nakao K, Mishiro S, Miyakawa Y, Akahane Y, Suzuki H. Demographic, clinical, and virological characteristics of hepatocellular carcinoma in Asia: survey of 414 patients from four countries. J Med Virol. 2002; 67(3): 394-400.
157	Li XJ, Yang HX, Wang XQ, Tang NH, Xu CS. Detection of HBV and HCV in patients with primary liver cancer. J Fujian Med Univ. 2002; 36: 380-1.
158	Li Q, Fottler MD. Determinants of maternal mortality in rural China. Health Serv Manage Res. 1996; 9(1): 45-54.
159	Ming L, Thorgeirsson SS, Gail MH, Lu P, Harris CC, Wang N, Shao Y, Wu Z, Liu G, Wang X, Sun Z. Dominant role of hepatitis B virus and cofactor role of aflatoxin in hepatocarcinogenesis in Qidong, China. Hepatology. 2002; 36(5): 1214-20.
160	Zhang XH, Xu J, Chen DQ, Guo LF, Qiu LQ. Effectiveness of treatment to improve pregnancy outcomes among women with syphilis in Zhejiang Province, China. Sex Transm Infect. 2016; 92(7): 537-541.
161	Centre for Research on the Epidemiology of Disasters (CRED). EM-DAT: The OFDA/CRED International Disaster Database. Brussels, Belgium: Catholic University of Leuven.
162	Fu Q, Li SZ, Wu WP, Hou YY, Zhang S, Feng Y, Zhang LP, Tang LH. Endemic characteristics of infantile visceral leishmaniasis in the People's Republic of China. Parasites Vectors. 2013; 143.
163	Lili X, Jian H, Mengjun Z, Yinglan W, Donghua X, Aihua W, Fanjuan K, Hua W, Zhiyu L. Epidemiological analysis of maternal deaths in Hunan province in China between 2009 and 2014. PLoS One. 2018; 13(11): e0207920.
164	Zhao S, Li Z, Zhou S, Zheng C, Ma H. Epidemiological Feature of Visceral Leishmaniasis in China, 2004-2012. Iran J Public Health. 2015; 44(1): 51-9.
165	Shen J, Xu YC, Gao Z, Niu JY, Shen HB, Ye BF. Epidemiological statocellulaudy on the etiologic synergistic interaction of HCV and HBV in the development of hepatocacinoma. Chin Natl J New Gastroenterol. 1997; 14: 72-74.
166	Fung KT, Fung J, Lai CL, Yuen MF. Etiologies of chronic liver diseases in Hong Kong. Eur J Gastroenterol Hepatol. 2007; 19(8): 659-64.
167	Ye BF, Shen J, Xu YC, Niu JY, Chen JG, Zhang BC, Liu B, Jiang YH. Etiology study on the relationship between HBV, HCV and HCC. Chin J Epidemiol. 1994; 15: 131-4.
168	World Health Organization (WHO). Expanded programme on immunization. Measles outbreak. Wkly Epidemiol Rec. 1990; 65(49): 379-81.
169	Chen J, Yu B, Wang Y, Tang M, Hu Y, Cai T, Zhang F, Zinkernagel D von, Harwell JI, Huang ZJ. Expansion of HIV care and treatment in Yunnan Province, China: Treatment outcomes with scale up of combination antiretroviral therapy. AIDS Care. 2014; 26(5): 633–41.
170	Liu J, Tong C, Liu J, Jiang Y, Zhao X, Zhang Y, Liu H, Lu B, Wan K. First insight into the genotypic diversity of clinical Mycobacterium tuberculosis isolates from Gansu Province, China. PLoS One. 2014; 9(6): e99357.
171	Chen L, Pang Y, Ma L, Yang H, Ru H, Yang X, Yan S, Jia M, Xu L. First Insight into the Molecular Epidemiology of Mycobacterium tuberculosis Isolates from the Minority

	Enclaves of Southwestern China. Biomed Res Int. 2017; 2017: 2505172.
172	World Health Organization (WHO). FluNet Influenza Laboratory Surveillance Information. Geneva, Switzerland: World Health Organization (WHO).
173	Lu B, Zhao P, Liu B, Dong H, Yu Q, Zhao X, Wan K. Genetic diversity of Mycobacterium tuberculosis isolates from Beijing, China assessed by Spoligotyping, LSPs and VNTR profiles. BMC Infect Dis. 2012; 12: 372.
174	Yu Q, Su Y, Lu B, Ma Y, Zhao X, Yang X, Dong H, Liu Y, Lian L, Wan L, Wu Y, Wan K. Genetic diversity of Mycobacterium tuberculosis isolates from Inner Mongolia, China. PLoS One. 2013; 8(5): e57660.
175	Dong H, Shi L, Zhao X, Sang B, Lv B, Liu Z, Wan K. Genetic diversity of Mycobacterium tuberculosis isolates from Tibetans in Tibet, China. PLoS One. 2012; 7(3): e33904.
176	Yuan J-M, Fan Y, Ognjanovic S, Wang R, Van Den Berg D, Govindarajan S, Mimi CY. Genetic polymorphisms of epidermal growth factor in relation to risk of hepatocellular carcinoma: two case-control studies . BMC Gastroenterol. 2013; 13(1): 32.
177	Lu W, Lu B, Liu Q, Dong H, Shao Y, Jiang Y, Song H, Chen C, Li G, Xu W, Zhao X, Wan K, Zhu L. Genotypes of Mycobacterium tuberculosis isolates in rural China: using MIRU-VNTR and spoligotyping methods. Scand J Infect Dis. 2014; 46(2): 98–106.
178	Guo Y-L, Liu Y, Wang S-M, Li C-Y, Jiang G-L, Shi G-L, Song C-X. Genotyping and drug resistance patterns of Mycobacterium tuberculosis strains in five provinces of China. Int J Tuberc Lung Dis. 2011; 15(6): 789–94.
179	Ding X, Park YN, Taltavull TC, Thung SN, Jin X, Jin Y, Trung NS, Edamoto Y, Sata T, Abe K. Geographic characterization of hepatitis virus infections, genotyping of hepatitis B virus, and p53 mutation in hepatocellular carcinoma analyzed by in situ detection of viral genomes from carcinoma tissues: comparison among six different countries. Jpn J Infect Dis. 2003; 56(1): 12-8.
180	London School of Hygiene and Tropical Medicine. Global Atlas of Helminth Infections - Soil Transmitted Helminths. London, United Kingdom: London School of Hygiene and Tropical Medicine.
181	National Consortium for the Study of Terrorism and Responses to Terrorism (START). Global Terrorism Database. College Park , MD, United States of America: University of Maryland.
182	Au W-Y, Ma ESK, Lam VMS, Chan JLC, Pang A, Kwong Y-L. Glucose 6-phosphate dehydrogenase (G6PD) deficiency in elderly Chinese women heterozygous for G6PD variants. Am J Med Genet A. 2004; 129A(2): 208-11.
183	Lai HC, Lai MP, Leung KS. Glucose-6-phosphate dehydrogenase deficiency in Chinese. J Clin Pathol. 1968; 21(1): 44-7.
184	Yue PC, Strickland M. Glucose-6-phosphate-dehydrogenase deficiency and neonatal jaundice in Chinese male infants in Hong Kong. Lancet. 1965; 1(7381): 350-1.
185	Xu Z, Shen FM, Xu ZY, Huang QS. HCV infection and primary liver cell cancer. Tumor. 1990; 10: 115.
186	Qiao C-X. Health-related quality of life evaluated by tumor node metastasis staging system in patients with hepatocellular carcinoma . World J Gastroenterol. 2012; 18(21): 2689.
187	Gong J, Yang JY, Li RC, Wang SS, Li YP, Chen KL. Hepatitis C virus and primary liver cancer in a hyperendemic area of liver cancer. Chin J Public Health. 1993; 12: 328-330.
188	Zhao JF, Yang P, Su W, Cui Z, Wang SM. Hepatitis C virus infection and hepatocellular carcinoma. J Bengbu Med Coll. 2000; 25: 169-170.
189	Chen Y-D, Liu M-Y, Yu W-L, Li J-Q, Peng M, Dai Q, Liu X, Zhou Z-Q. Hepatitis C virus infections and genotypes in China. Hepatobiliary Pancreat Dis Int. 2002; 1(2): 194-201.
190	Hong Kong Cancer Registry 1974-1977 - CI5. as it appears in Cancer Incidence in Five Continents Volume IV
191	Hong Kong Cancer Registry 1978-1982 - CI5. as it appears in Cancer Incidence in Five Continents Volumes I-VIII 1950-1997
192	Hong Kong Cancer Registry 1983 - CI5. as it appears in Cancer Incidence in Five Continents Time Trends Annual Dataset (Summary and Detailed Databases)
193	Hong Kong Cancer Registry 1983-1987 - CI5. as it appears in Cancer Incidence in Five Continents Time Trends Annual Dataset (Summary and Detailed Databases)

194	Hong Kong Cancer Registry 1984 - CI5. as it appears in Cancer Incidence in Five Continents Time Trends Annual Dataset (Summary and Detailed Databases)
195	Hong Kong Cancer Registry 1985 - CI5. as it appears in Cancer Incidence in Five Continents Time Trends Annual Dataset (Summary and Detailed Databases)
196	Hong Kong Cancer Registry 1986 - CI5. as it appears in Cancer Incidence in Five Continents Time Trends Annual Dataset (Summary and Detailed Databases)
197	Hong Kong Cancer Registry 1987 - CI5. as it appears in Cancer Incidence in Five Continents Time Trends Annual Dataset (Summary and Detailed Databases)
198	Hong Kong Cancer Registry 1988 - CI5. as it appears in Cancer Incidence in Five Continents Time Trends Annual Dataset (Summary and Detailed Databases)
199	Hong Kong Cancer Registry 1988-1992 - CI5. as it appears in Cancer Incidence in Five Continents Volumes I-VIII 1950-1997
200	Hong Kong Cancer Registry 1989 - CI5. as it appears in Cancer Incidence in Five Continents Time Trends Annual Dataset (Summary and Detailed Databases)
201	Hong Kong Cancer Registry 1990 - CI5. as it appears in Cancer Incidence in Five Continents Time Trends Annual Dataset (Summary and Detailed Databases)
202	Hong Kong Cancer Registry 1991 - CI5. as it appears in Cancer Incidence in Five Continents Time Trends Annual Dataset (Summary and Detailed Databases)
203	Hong Kong Cancer Registry 1992 - CI5. as it appears in Cancer Incidence in Five Continents Time Trends Annual Dataset (Summary and Detailed Databases)
204	Hong Kong Cancer Registry 1993 - CI5. as it appears in Cancer Incidence in Five Continents Time Trends Annual Dataset (Summary and Detailed Databases)
205	Hong Kong Cancer Registry 1993-1997 - CI5. as it appears in Cancer Incidence in Five Continents Volumes I-VIII 1950-1997
206	Hong Kong Cancer Registry 1994 - CI5. as it appears in Cancer Incidence in Five Continents Time Trends Annual Dataset (Summary and Detailed Databases)
207	Hong Kong Cancer Registry 1995 - CI5. as it appears in Cancer Incidence in Five Continents Time Trends Annual Dataset (Summary and Detailed Databases)
208	Hong Kong Cancer Registry 1996 - CI5. as it appears in Cancer Incidence in Five Continents Time Trends Annual Dataset (Summary and Detailed Databases)
209	Hong Kong Cancer Registry 1997 - CI5. as it appears in Cancer Incidence in Five Continents Time Trends Annual Dataset (Summary and Detailed Databases)
210	Hong Kong Cancer Registry 1998 - CI5. as it appears in Cancer Incidence in Five Continents Time Trends Annual Dataset (Summary and Detailed Databases)
211	Hong Kong Cancer Registry 1998-2002 - CI5. as it appears in Cancer Incidence in Five Continents Volume IX Periodic Data 1998-2002
212	Hong Kong Cancer Registry 1999 - CI5. as it appears in Cancer Incidence in Five Continents Time Trends Annual Dataset (Summary and Detailed Databases)
213	Hong Kong Cancer Registry 2000 - CI5. as it appears in Cancer Incidence in Five Continents Time Trends Annual Dataset (Summary and Detailed Databases)
214	Hong Kong Cancer Registry 2001 - CI5. as it appears in Cancer Incidence in Five Continents Time Trends Annual Dataset (Summary and Detailed Databases)
215	Hong Kong Cancer Registry 2002 - CI5. as it appears in Cancer Incidence in Five Continents Time Trends Annual Dataset (Summary and Detailed Databases)
216	Hong Kong Cancer Registry. Hong Kong Cancer Registry Annual Tables 2003-2016. Kowloon, Hong Kong: Hong Kong Cancer Registry.
217	Census and Statistics Department (Hong Kong). Hong Kong Monthly Digest of Statistics April 2021. Hong Kong, Hong Kong: Census and Statistics Department (Hong Kong), 2021.
218	Census and Statistics Department (Hong Kong). Hong Kong Monthly Digest of Statistics August 2021. Hong Kong, Hong Kong: Census and Statistics Department (Hong Kong), 2021.
219	Census and Statistics Department (Hong Kong). Hong Kong Monthly Digest of Statistics February 2019. Hong Kong, Hong Kong: Census and Statistics Department (Hong Kong), 2019.
220	Census and Statistics Department (Hong Kong). Hong Kong Monthly Digest of Statistics

	February 2020. Hong Kong, Hong Kong: Census and Statistics Department (Hong Kong), 2020.
221	Census and Statistics Department (Hong Kong). Hong Kong Monthly Digest of Statistics February 2021. Hong Kong, Hong Kong: Census and Statistics Department (Hong Kong), 2021.
222	Census and Statistics Department (Hong Kong). Hong Kong Monthly Digest of Statistics July 2021. Hong Kong, Hong Kong: Census and Statistics Department (Hong Kong), 2021.
223	Census and Statistics Department (Hong Kong). Hong Kong Monthly Digest of Statistics June 2021. Hong Kong, Hong Kong: Census and Statistics Department (Hong Kong), 2021.
224	Census and Statistics Department (Hong Kong). Hong Kong Monthly Digest of Statistics May 2021. Hong Kong, Hong Kong: Census and Statistics Department (Hong Kong), 2021.
225	Hong Kong Vital Registration - Deaths 1980 ICD9. as it appears in WHO Mortality Database Version September 2016
226	Hong Kong Vital Registration - Deaths 1981 ICD9. as it appears in WHO Mortality Database Version September 2016
227	Hong Kong Vital Registration - Deaths 1982 ICD9. as it appears in WHO Mortality Database Version September 2016
228	Hong Kong Vital Registration - Deaths 1983 ICD9. as it appears in WHO Mortality Database Version September 2016
229	Hong Kong Vital Registration - Deaths 1984 ICD9. as it appears in WHO Mortality Database Version September 2016
230	Hong Kong Vital Registration - Deaths 1985 ICD9. as it appears in WHO Mortality Database Version September 2016
231	Hong Kong Vital Registration - Deaths 1986 ICD9. as it appears in WHO Mortality Database Version September 2016
232	Hong Kong Vital Registration - Deaths 1987 ICD9. as it appears in WHO Mortality Database Version September 2016
233	Hong Kong Vital Registration - Deaths 1988 ICD9. as it appears in WHO Mortality Database Version September 2016
234	Hong Kong Vital Registration - Deaths 1989 ICD9. as it appears in WHO Mortality Database Version September 2016
235	Hong Kong Vital Registration - Deaths 1990 ICD9. as it appears in WHO Mortality Database Version September 2016
236	Hong Kong Vital Registration - Deaths 1991 ICD9. as it appears in WHO Mortality Database Version September 2016
237	Hong Kong Vital Registration - Deaths 1992 ICD9. as it appears in WHO Mortality Database Version September 2016
238	Hong Kong Vital Registration - Deaths 1993 ICD9. as it appears in WHO Mortality Database Version September 2016
239	Hong Kong Vital Registration - Deaths 1994 ICD9. as it appears in WHO Mortality Database Version November 2015
240	Hong Kong Vital Registration - Deaths 1995 ICD9. as it appears in WHO Mortality Database Version November 2015
241	Hong Kong Vital Registration - Deaths 1996 ICD9. as it appears in WHO Mortality Database Version November 2015
242	Hong Kong Vital Registration - Deaths 1997 ICD9. as it appears in WHO Mortality Database Version November 2015
243	Hong Kong Vital Registration - Deaths 1998 ICD9. as it appears in WHO Mortality Database Version November 2015
244	Hong Kong Vital Registration - Deaths 1999 ICD9. as it appears in WHO Mortality Database Version November 2015
245	Hong Kong Vital Registration - Deaths 2000 ICD9. as it appears in WHO Mortality Database Version November 2015

246	Hong Kong Vital Registration - Deaths 2001 ICD10. as it appears in WHO Mortality Database Version October 2017
247	Hong Kong Vital Registration - Deaths 2002 ICD10. as it appears in WHO Mortality Database Version October 2017
248	Hong Kong Vital Registration - Deaths 2003 ICD10. as it appears in WHO Mortality Database Version October 2017
249	Hong Kong Vital Registration - Deaths 2004 ICD10. as it appears in WHO Mortality Database Version October 2017
250	Hong Kong Vital Registration - Deaths 2005 ICD10. as it appears in WHO Mortality Database Version October 2017
251	Hong Kong Vital Registration - Deaths 2006 ICD10. as it appears in WHO Mortality Database Version October 2017
252	Hong Kong Vital Registration - Deaths 2007 ICD10. as it appears in WHO Mortality Database Version October 2017
253	Hong Kong Vital Registration - Deaths 2008 ICD10. as it appears in WHO Mortality Database Version October 2017
254	Hong Kong Vital Registration - Deaths 2009 ICD10. as it appears in WHO Mortality Database Version October 2017
255	Hong Kong Vital Registration - Deaths 2010 ICD10. as it appears in WHO Mortality Database Version October 2017
256	Hong Kong Vital Registration - Deaths 2011 ICD10. as it appears in WHO Mortality Database Version October 2017
257	Hong Kong Vital Registration - Deaths 2012 ICD10. as it appears in WHO Mortality Database Version October 2017
258	Hong Kong Vital Registration - Deaths 2013 ICD10. as it appears in WHO Mortality Database Version October 2017
259	Hong Kong Vital Registration - Deaths 2014 ICD10. as it appears in WHO Mortality Database Version April 2018
260	Hong Kong Vital Registration - Deaths 2015 ICD10. as it appears in WHO Mortality Database Version April 2018
261	Hong Kong Vital Registration - Deaths 2016 ICD10. as it appears in WHO Mortality Database Version May 2019
262	Hong Kong Vital Registration - Deaths 2017 ICD10. as it appears in WHO Mortality Database Version December 2019
263	Calupitan J, Favila A. Hope fades in Philippines for dozens buried in landslides. Associated Press [Internet]. 2018 Sep 17.
264	Institute for Health Metrics and Evaluation (IHME). IHME COVID-19 Error Patch Dataset.
265	Institute for Health Metrics and Evaluation (IHME). IHME GBD Cancer Incidence and Mortality Estimates.
266	Zhu Q, Wang L, Lin W, Bulterys M, Yang W, Sun D, Cui Z, Kaplan J, Kleinman N, Wei X, Chung J, Wang Z. Improved survival with co-trimoxazole prophylaxis among people living with HIV/AIDS who initiated antiretroviral treatment in Henan Province, China. Curr HIV Res. 2014; 12(5): 359–65.
267	Yan T, Cai R, Mo O, Zhu D, Ouyang H, Huang L, Zhao M, Huang F, Li L, Liang X, Xu X. Incidence and complete molecular characterization of glucose-6-phosphate dehydrogenase deficiency in the Guangxi Zhuang autonomous region of southern China: description of four novel mutations. Haematologica. 2006; 91(10): 1321-8.
268	Yang H, Wang Q, Zheng L, Zhan XF, Lin M, Lin F, Tong X, Luo ZY, Huang Y, Yang LY. Incidence and molecular characterization of Glucose-6-Phosphate Dehydrogenase deficiency among neonates for newborn screening in Chaozhou, China. Int J Lab Hematol. 2015; 37(3): 410-9.
269	Zhang S, Lu Z, Liu H, Xiao X, Zhao Z, Bao G, Han J, Jing T, Chen G. Incidence of Japanese encephalitis, visceral leishmaniasis and malaria before and after the Wenchuan earthquake, in China. Addict Behav Rep. 2013; 128(1): 85-9.
270	Liu M, Jiang W, Liu Y, Zhang Y, Wei X, Wang W. Increased genetic diversity of the

	Mycobacterium tuberculosis W-Beijing genotype that predominates in eastern China. Infect Genet Evol. 2014; 22: 23–9.
271	Huang W, Yu H, Wang F, Li G. Infant mortality among various nationalities in the middle part of Guizhou, China. Soc Sci Med. 1997; 45(7): 1031-40.
272	Fan XL, Peng WW, Yao JL, Zhou YP, Lu L, Zhen XC, Chen Q. Infection of HBV, HCV and HDV in Hepatocellular Carcinoma. Chin J Infect Dis. 1995; 13: 129-32.
273	International Institute for Strategic Studies. International Institute for Strategic Studies Armed Conflict Database. London, United Kingdom: International Institute for Strategic Studies.
274	Zhang, T, Yang, Y, Yu, F, Zhao, Y, Lin, F, Minhas, V, Wood, C, He, N. Kaposi`s sarcoma associated herpesvirus infection among female sex workers and general population women in Shanghai, China: a cross-sectional study. BMC Infect Dis. 2014; 58.
275	Government of the Macao Special Administrative Region. Macao Monthly Bulletin of Statistics April 2021.
276	Government of the Macao Special Administrative Region. Macao Monthly Bulletin of Statistics December 2016.
277	Government of the Macao Special Administrative Region. Macao Monthly Bulletin of Statistics December 2017.
278	Government of the Macao Special Administrative Region. Macao Monthly Bulletin of Statistics December 2018.
279	Government of the Macao Special Administrative Region. Macao Monthly Bulletin of Statistics December 2019.
280	Government of the Macao Special Administrative Region. Macao Monthly Bulletin of Statistics December 2020.
281	Government of the Macao Special Administrative Region. Macao Monthly Bulletin of Statistics January 2016.
282	Government of the Macao Special Administrative Region. Macao Monthly Bulletin of Statistics January 2017.
283	Government of the Macao Special Administrative Region. Macao Monthly Bulletin of Statistics January 2018.
284	Government of the Macao Special Administrative Region. Macao Monthly Bulletin of Statistics January 2019.
285	Government of the Macao Special Administrative Region. Macao Monthly Bulletin of Statistics January 2020.
286	Government of the Macao Special Administrative Region. Macao Monthly Bulletin of Statistics January 2021.
287	Government of the Macao Special Administrative Region. Macao Monthly Bulletin of Statistics July 2021.
288	Government of the Macao Special Administrative Region. Macao Monthly Bulletin of Statistics June 2021.
289	Government of the Macao Special Administrative Region. Macao Monthly Bulletin of Statistics May 2021.
290	Macau Vital Registration - Deaths 1994 ICD9. as it appears in WHO Mortality Database Version November 2015
291	Zhu L, Qin M, Du L, Xie RH, Wong T, Wen SW. Maternal and congenital syphilis in Shanghai, China, 2002 to 2006. Int J Infect Dis. 2010; Suppl. 3: e45-8.
292	Qin J-B, Feng T-J, Yang T-B, Hong F-C, Lan L-N, Zhang C-L. Maternal and paternal factors associated with congenital syphilis in Shenzhen, China: a prospective cohort study. Eur J Clin Microbiol Infect Dis. 2014; 33(2): 22132.
293	You F, Huo K, Wang R, Xu D, Deng J, Wei Y, Shi F, Liu H, Cheng G, Zhang Z, Yang P, Sun T, Wang X, Jacobsson B, Zhu C. Maternal mortality in Henan Province, China: changes between 1996 and 2009. PLoS One. 2012; 7(10): e47153.
294	International Organization for Migration (IOM). Missing Migrants Project 2014-2020. Geneva, Switzerland: International Organization for Migration (IOM).
295	Zheng C, Li S, Luo Z, Pi R, Sun H, He Q, Tang K, Luo M, Li Y, Couvin D, Rastogi N, Sun Q. Mixed Infections and Rifampin Heteroresistance among Mycobacterium

	tuberculosis Clinical Isolates. J Clin Microbiol. 2015; 53(7): 2138–47.
296	Lin M, Han ZJ, Wang Q, Zheng L, Wang Y, Yang H, Huang Y, Lin F, Zhan XF, Lin CP, Wu JR, Luo ZY, Liu JB, Yan ZH, Zheng SY, Zheng JK, Lu M, Zhu JJ, Xie LX, Yang LY. Molecular epidemiological survey of hemoglobinopathies in the Wuxi region of Jiangsu Province, eastern China. Hemoglobin. 2013; 37(5): 454-66.
297	Zhang D, An J, Wang J, Hu C, Wang Z, Zhang R, Wang Y, Pang Y. Molecular typing and drug susceptibility of Mycobacterium tuberculosis isolates from Chongqing Municipality, China. Infect Genet Evol. 2013; 13: 310–6.
298	Zhao Y, Li H, Xing J, Yang H, Ma X, Xu J, Shi J, Yan G. Molecular Typing of Mycobacterium Tuberculosis Isolates Circulating in Henan, Central China. Exp Ther Med. 2012; 4(5): 949–53.
299	Lam STS, Cheng ML. Neonatal screening in Hong Kong and Macau. Southeast Asian J Trop Med Public Health. 2003; 73-5.
300	Xiong J, Wang J, Huang J, Sun W, Wang J, Chen D. Non-alcoholic steatohepatitis-related liver cirrhosis is increasing in China: a ten-year retrospective study. Clinics (Sao Paulo). 2015; 70(8): 563-8.
301	Khan MI, Ochiai RL, Von Seidlein L, Dong B, Bhattacharya SK, Agtini MD, Bhutta ZA, Do GC, Ali M, Kim DR, Favorov M, Clemens JD. Non-typhoidal Salmonella rates in febrile children at sites in five Asian countries: Non-typhoidal Salmonella in Asia. Trop Med Int Health. 2010; 15(8): 960–3.
302	Yao XY, Yu J, Chen SP, Xiao JW, Zheng QC, Liu HY, Zhang L, Xian Y, Zou L. Prevalence and genetic analysis of a-thalassemia and b-thalassemia in Chongqing area of China. Gene. 2013; 532(1): 120-4.
303	Yang JT, Zhao HG, Zhao SF, Li PZ. Prevalence of HCV and HBV infection in patients with primary hepatocellular carcinoma in Shanxi Province. Chin J Epidemiol. 1999; 20: 215-7.
304	Du L, Li Y, Jin H, Huang C, Gu Y, Zhu L, Xu B. Prevent Mother-to-Child Transmission (PMTCT) Programs and Enhancement of Maternal Healthcare Infrastructure to Improve Early Detection of Maternal Syphilis in Shanghai, China. Int J Environ Res Public Health. 2019; 16(6).
305	Yang S, Zhang B, Zhao J, Wang J, Flick L, Qian Z, Zhang D, Mei H. Progress on the maternal mortality ratio reduction in Wuhan, China in 2001-2012. PLoS One. 2014; 9(2): e89510.
306	Mao Y-M, Luo Z-Y, Li B, Hu T-Y. Prospective Study on the Survival of HCC Patients Treated with Transcatheter Arterial Lipiodol Chemoembolization . Asian Pac J Cancer Prev. 2012; 13(3): 1039-42.
307	Hong F-C, Yang Y-Z, Liu X-L, Feng T-J, Liu J-B, Zhang C-L, Lan L-N, Yao M-Z, Zhou H. Reduction in mother-to-child transmission of syphilis for 10 years in Shenzhen, China. Sex Transm Dis. 2014; 41(3): 188–93.
308	Tang S, Tan S, Yao L, Li F, Li L, Guo X, Liu Y, Hao X, Li Y, Ding X, Zhang Z, Tong L, Huang J. Risk factors for poor treatment outcomes in patients with MDR-TB and XDR-TB in China: retrospective multi-center investigation. PLoS One. 2013; 8(12): e82943.
309	Ochiai RL, Wang X, von Seidlein L, Yang J, Bhutta ZA, Bhattacharya SK, Agtini M, Deen JL, Wain J, Kim DR, Ali M, Acosta CJ, Jodar L, Clemens JD. Salmonella Paratyphi A Rates, Asia. Emerg Infect Dis. 2005; 11(11): 1764-6.
310	Jiang J, Li B, Cao W, Jiang X, Jia X, Chen Q, Wu J. Screening and prevention of neonatal glucose 6-phosphate dehydrogenase deficiency in Guangzhou, China. Genet Mol Res. 2014; 13(2): 4272–9.
311	Fung RH, Keung YK, Chung GS. Screening of pyruvate kinase deficiency and G6PD deficiency in Chinese newborn in Hong Kong. Arch Dis Child. 1969; 44(235): 373-6.
312	Zeng JH, Dai YS, Cao MQ, Hu XK. Serological markers of HCV and HBV and primary hepatocellular carcinoma. Mod Prev Med. 2003; 30: 324-5.
313	Wu Z, Rotheram-Borus MJ, Li L, Guan J, Detels R, Yin Y, Wu S, Liu Z, Lin C, Hsieh J, NIMH Collaborative HIV/STD Prevention Trial Group. Sexually transmitted diseases and risk behaviors among market vendors in China. Sex Transm Dis. 2007; 34(12): 1030-4.
314	Chen XS, Yin YP, Chen LP, Thuy NT, Zhang GY, Shi MQ, Hu LH, Yu YH. Sexually

	transmitted infections among pregnant women attending an antenatal clinic in Fuzhou, China. Sex Transm Dis. 2006; 33(5): 296-301.
315	Weatherall D. Sickle Cell and Thalassemias Prevalence Data, Personal Correspondence with David Weatherall. [Unpublished].
316	Xuan S-Y, Xin Y-N, Chen H, Shi G-J, Guan H-S, Li Y. Significance of hepatitis B virus surface antigen, hepatitis C virus expression in hepatocellular carcinoma and pericarcinomatous tissues . World J Gastroenterol. 2007; 13(12): 1870.
317	Feng XL, Zhu J, Zhang L, Song L, Hipgrave D, Guo S, Ronsmans C, Guo Y, Yang Q. Socio-economic disparities in maternal mortality in China between 1996 and 2006. BJOG. 2010; 117(12): 1527-36.
318	Pang Y, Zhou Y, Zhao B, Liu G, Jiang G, Xia H, Song Y, Shang Y, Wang S, Zhao Y. Spoligotyping and drug resistance analysis of Mycobacterium tuberculosis strains from national survey in China. PLoS One. 2012; 7(3): e32976.
319	Jiang W, Yu G, Liu P, Geng Q, Chen L, Lin Q, Ren X, Ye W, He Y, Guo Y, Duan S, Wen J, Li H, Qi Y, Jiang C, Zheng Y, Liu C, Si E, Zhang Q, Tian Q, Du C. Structure and function of glucose-6-phosphate dehydrogenase-deficient variants in Chinese population. Hum Genet. 2006; 119(5): 463-78.
320	Zhang X, Yu Y, Yang H, Xu H, Vermund SH, Liu K. Surveillance of Maternal Syphilis in China: Pregnancy Outcomes and Determinants of Congenital Syphilis. Med Sci Monit. 2018; 24: 7727-7735.
321	Yang, LG, Tucker, JD, Liu, FY, Ren, XQ, Hong, X, Wang, C, McLaughlin, MM, Bien, CH, Chen, XS, Yang, B. Syphilis screening among 27,150 pregnant women in South Chinese rural areas using point-of-care tests. PLoS One. 2013; 8(8): e72149.
322	Cheng JQ, Zhou H, Hong FC, Zhang D, Zhang YJ, Pan P, Cai YM. Syphilis screening and intervention in 500,000 pregnant women in Shenzhen, the People's Republic of China. Sex Transm Infect. 2007; 83(5): 347–50.
323	Yang TY, Yang XY, Ch'en WC, Qi SL, Jin YJ, Gan WJ, Qu Q. Thalassemia in China. Ann N Y Acad Sci. 1985; 445: 92-105.
324	Pew Research Center. The Future of the Global Muslim Population. Washington, DC, United States: Pew Research Center, 2011.
325	Liu J, Huang Y, Wang J, Guo N, Li J, Dong X, Ma H, Tiemuer M, Huang M, Wright DJ, Ness, P, Shan, H. The increasing prevalence of serologic markers for syphilis among Chinese blood donors in 2008 through 2010 during a syphilis epidemic. Transfusion. 2012; 52(8): 1741-9.
326	Yongjun T, Samuelson J, Qingsheng D, Ali MM, Li X, Yanjian M, Xiaoqing C, Jun L, Jian C, Bi L. The prevalence of sexually transmitted and other lower reproductive tract infections among rural women in Sichuan Province, China. Southeast Asian J Trop Med Public Health. 2009; 40(5): 1038-47.
327	Zhang ZQ, Huang TR, He ZF, Yu JH, Xu QF, Huang ZD. The relationship between HCV and HBV in the etiology of primary liver cancer. Guangxi Med J. 1993; 15: 171-4.
328	Yu SZ, Zi XL, Chen G, Li J. The relationship between viral hepatitis and primary liver cancer in four areas of China. Chin J Epidemiol. 1997; 18: 214-6.
329	Lu CH, Hou SJ. The relationship in HBV, HCV infection and cirrhosis and primary liver cancer. Clin Med. 1997; 17: 17-18.
330	Gao JD, Shao YF, Xu Y, Ming LH, Wu ZY, Liu GT, Wang XH, Gao WH, Sun YT, Feng XL, Liang LM, Zhang YH, Sun ZT. Tight association of hepatocellular carcinoma with HBV infection in North China. Hepatobiliary Pancreat Dis Int. 2005; 4(1): 46-9.
331	Jisheng Y, Friedman E, Guo J, Mosher S. Tombstone: The Great Chinese Famine, 1958-1962. New York: Farrar, Straus and Giroux (Macmillan), 2012.
332	Jin Y, Guo H, Wang X, Chen X, Jiang Z, Hu G, Hou J, Jiang S, Yang X, Liu Y, Xu L, Wang N. Traditional Chinese medicine could increase the survival of people living with HIV in rural central China: a retrospective cohort study, 2004-2012. Am J Chin Med. 2014; 42(6): 1333–44.
333	Du L, Qin M, Zhang L, Xu H, Zhu L. Trends in maternal mortality in resident vs. migrant women in Shanghai, China, 2000-2009: a register-based analysis. Reprod Health Matters. 2012; 20(39): 73-80.

334	Department of Peace and Conflict Research, Uppsala University. UCDP Georeferenced Event Dataset, Version 17.1, 2016. Uppsala, Sweden: Department of Peace and Conflict Research, Uppsala University, 2017.
335	United Nations Office on Drugs and Crime (UNODC). United Nations Office on Drugs and Crime Global Study on Homicide 2011. Vienna, Austria: United Nations Office on Drugs and Crime (UNODC), 2011.
336	Liu C, Xiao G-Q, Yan L-N, Li B, Li J, Wen T-F, Wang W-T, Xu M-Q, Yang J-Y. Value of ?-fetoprotein in association with clinicopathological features of hepatocellular carcinoma . World J Gastroenterol. 2013; 19(11): 1811.
337	United Nations Children's Fund (UNICEF), World Health Organization (WHO). WHO and UNICEF Reported Disease Incidence Time Series. Geneva, Switzerland: World Health Organization (WHO).
338	World Health Organization (WHO). WHO Distribution of Measles Cases by Country and by Month 2011-2020.
339	World Health Organization (WHO). WHO Global Health Observatory - Cholera: Number of Reported Deaths by Country 1949-2016. Geneva, Switzerland: World Health Organization (WHO).
340	World Health Organization (WHO). WHO Global Health Observatory - Visceral Leishmaniasis: Number of Reported Cases by Country 2005-2022. Geneva, Switzerland: World Health Organization (WHO).
341	World Health Organization (WHO). WHO Global Health Observatory Interactive Graph - Number of Cases of Visceral Leishmaniasis Reported 2013. Geneva, Switzerland: World Health Organization (WHO).
342	World Health Organization (WHO). WHO Global Project on Anti-Tuberculosis Drug Resistance Surveillance Data 1988-2015.
343	World Health Organization (WHO). WHO International Travel and Health, Annex 1, Countries With Risk of Yellow Fever Transmission and Countries Requiring Yellow Fever Vaccination 2017 Update. Geneva, Switzerland: World Health Organization (WHO), 2017.
344	World Health Organization (WHO). WHO Mortality Database Version April 2018. Geneva, Switzerland: World Health Organization (WHO).
345	World Health Organization (WHO). WHO Mortality Database Version December 2019. Geneva, Switzerland: World Health Organization (WHO).
346	World Health Organization (WHO). WHO Mortality Database Version May 2019. Geneva, Switzerland: World Health Organization (WHO).
347	World Health Organization (WHO). WHO Mortality Database Version November 2015. Geneva, Switzerland: World Health Organization (WHO).
348	World Health Organization (WHO). WHO Mortality Database Version October 2017. Geneva, Switzerland: World Health Organization (WHO).
349	World Health Organization (WHO). WHO Mortality Database Version September 2016. Geneva, Switzerland: World Health Organization (WHO).
350	World Health Organization (WHO). WHO Tuberculosis Case Notifications. Geneva, Switzerland: World Health Organization (WHO).
351	Wang Q, Tan Y, Ren Y, Dong L, Xie Z, Tang L, Cao D, Zhang W, Hu H, Wang H. Zinc finger protein ZBTB20 expression is increased in hepatocellular carcinoma and associated with poor prognosis . BMC Cancer. 2011; 11(1): 271.
352	Liang J, Zhu J, Wang Y, Wu Y, Dai L, Miao L, Zhou G. [Epidemiological analysis of the maternal mortality surveillance data (1996-2000) in China]. J Sichuan Univ Med Sci Ed. 2007; 38(1): 138-41.
353	Xiao X, Zhou Y, Sun W, Chen D. [Prevalence of syphilis during pregnancy and risk factors for maternal and perinatal infections: a 2009-2013 survey]. J South Med Univ. 2014; 34(1): 144–6.
354	Johns Hopkins University. 2019 Novel Coronavirus COVID-19 (2019-nCoV) Data Repository by Johns Hopkins CSSE 2020-2022. Baltimore, Maryland: Johns Hopkins University.
355	Chen C-H, Changchien C-S, Lee C-M, Tung W-C, Hung C-H, Hu T-H, Wang J-H, Wang J-C, Lu S-N. A study on sequence variations in pre-S/surface, X and enhancer II/core

	promoter/precore regions of occult hepatitis B virus in non-B, non-C hepatocellular carcinoma patients in Taiwan. Int J Cancer. 2009; 125(3): 621-9.
356	Tsai JF, Jeng JE, Ho MS, Chang WY, Hsieh MY, Lin ZY, Tsai JH. Additive effect modification of hepatitis B surface antigen and e antigen on the development of hepatocellular carcinoma . Br J Cancer. 1996; 73(12): 1498.
357	Climate Change and African Political Stability Project (CCAPS). Armed Conflict Location and Event Dataset, Realtime - Robert S. Strauss Center as referenced in Raleigh, Clionadh, Andrew Linke, Havard Hegre and Joakim Karlsen. 2010. Introducing ACLED-Armed Conflict Location and Event Data. Journal of Peace Research 47(5), 651-60.
358	Huang S-F, Su W-J, Dou H-Y, Feng J-Y, Lee Y-C, Huang R-M, Lin C-H, Hwang J-J, Lee J-J, Yu M-C. Association of Mycobacterium tuberculosis genotypes and clinical and epidemiological features - a multi-center study in Taiwan. Infect Genet Evol. 2012; 12(1): 28–37.
359	Kuo CH, Changchien CS, Yang CY, Sheen IS, Liaw YF. Bacteremia in patients with cirrhosis of the liver. Liver. 1991; 11(6): 334-9.
360	Peace Research Institute Oslo (PRIO). Battle Deaths Dataset Version 3.1, 2009. Oslo, Norway: Peace Research Institute Oslo (PRIO), 2009.
361	Tsai JF, Chuang LY, Jeng JE, Ho MS, Hsieh MY, Lin ZY, Wang LY. Betel quid chewing as a risk factor for hepatocellular carcinoma: a case-control study . Br J Cancer. 2001; 84(5): 709.
362	Chien Y-H, Lee N-C, Wu S-T, Liou J-J, Chen H-C, Hwu W-L. Changes in incidence and sex ratio of glucose-6-phosphate dehydrogenase deficiency by population drift in Taiwan. Southeast Asian J Trop Med Public Health. 2008; 39(1): 154-61.
363	Chang J-R, Chen Y-Y, Huang T-S, Huang W-F, Kuo S-C, Tseng F-C, Su I-J, Lin C-H, Chen Y-S, Sun J-R, Chiueh T-S, Dou H-Y. Clonal expansion of both modern and ancient genotypes of Mycobacterium tuberculosis in southern Taiwan. PLoS One. 2012; 7(8): e43018.
364	Chen Y-Y, Chang J-R, Wu C-D, Yeh Y-P, Yang S-J, Hsu C-H, Lin M-C, Tsai C-F, Lin M-S, Su I-J, Dou H-Y. Combining molecular typing and spatial pattern analysis to identify areas of high tuberculosis transmission in a moderate-incidence county in Taiwan. Sci Rep. 2017; 7(1): 5394.
365	Lee CH, Hsieh SY, Chang CJ, Lin YJ. Comparison of clinical characteristics of combined hepatocellular-cholangiocarcinoma and other primary liver cancers. J Gastroenterol Hepatol. 2013; 28(1): 122-7.
366	Tseng P-L, Wang J-H, Hung C-H, Tung H-D, Chen T-M, Huang W-S, Liu S-L, Hu T-H, Lee C-M, Lu S-N. Comparisons of noninvasive indices based on daily practice parameters for predicting liver cirrhosis in chronic hepatitis B and hepatitis C patients in hospital and community populations . Kaohsiung J Med Sci. 2013; 29(7): 385-95.
367	Cystic Echinococcosis Endemicity Estimates identified through systematic review and personal communication, as provided by GBD 2015 expert. [Unpublished].
368	Lin Y-S, Yen Y-F. Determinants of mortality before start of and during tuberculosis treatment among elderly patients: a population-based retrospective cohort study. Age Ageing. 2015; 44(3): 490–6.
369	Chen W, Macatula T, Lin C, Lin C, Lin S. Diabetes may not affect outcomes in hepatocellular carcinoma after radio-frequency ablation . Hepatogastroenterology. 2011; 58(106): 551-7.
370	Chen C-T, Chen J-Y, Wang J-H, Chang K-C, Tseng P-L, Kee K-M, Chen P-F, Tsai L-S, Chen S-C, Lin S-C, Lu S-N. Diabetes mellitus, metabolic syndrome and obesity are not significant risk factors for hepatocellular carcinoma in an HBV- and HCV-endemic area of Southern Taiwan. Kaohsiung J Med Sci. 2013; 29(8): 451-9.
371	Chen Y-Y, Tseng F-C, Chang J-R, Kuo S-C, Lee J-J, Yeh J-J, Chiueh T-S, Sun J-R, Su I-J, Dou H-Y. Distinct modes of transmission of tuberculosis in aboriginal and non-aboriginal populations in Taiwan. PLoS One. 2014; 9(11): e112633.
372	Lin K-J, Lin T-M, Wang C-H, Liu H-C, Lin Y-L, Eng H-L. Down-regulation of Toll-like receptor 7 expression in hepatitis-virus-related human hepatocellular carcinoma . Hum Pathol. 2013; 44(4): 534-41.

373	Su WJ, Lo HY, Chang CH, Chang LY, Chiu CH, Lee PI, Lu CY, Hsieh YC, Lai MS, Lin TY. Effectiveness of Pneumococcal Conjugate Vaccines of Different Valences Against Invasive Pneumococcal Disease Among Children in Taiwan: A Nationwide Study. Pediatr Infect Dis J. 2016; 35(4): e124-33.
374	Centre for Research on the Epidemiology of Disasters (CRED). EM-DAT: The OFDA/CRED International Disaster Database. Brussels, Belgium: Catholic University of Leuven.
375	Chen PL, Li CY, Hsieh TH, Chang CM, Lee HC, Lee NY, Wu CJ, Lee CC, Shih HI, Ko WC. Epidemiology, disease spectrum and economic burden of non-typhoidal Salmonella infections in Taiwan, 2006–2008. Epidemiol Infect. 2012; 140(12): 2256-63.
376	Chang YK, Chao SL, Huang LW. Gestational and congenital syphilis in Hualien. J Formos Med Assoc. 1992; 91(6): 620-3.
377	National Consortium for the Study of Terrorism and Responses to Terrorism (START). Global Terrorism Database. College Park , MD, United States of America: University of Maryland.
378	Tsai J-F, Jeng J-E, Chuang L-Y, Ho M-S, Ko Y-C, Lin Z-Y, Hsieh M-Y, Chen S-C, Chuang W-L, Wang L-Y, Yu M-L, Dai C-Y, Ho C. Habitual betel quid chewing as a risk factor for cirrhosis: a case-control study. Medicine (Baltimore). 2003; 82(5): 365-72.
379	Jeng J-E, Tsai J-F, Chuang L-Y, Ho M-S, Lin Z-Y, Hsieh M-Y, Chen S-C, Chuang W-L, Wang L-Y, Yu M-L, Dai C-Y, Chang J-G. Heat shock protein A1B 1267 polymorphism is highly associated with risk and prognosis of hepatocellular carcinoma: a case-control study. Medicine (Baltimore). 2008; 87(2): 87-98.
380	Wu C-C, Ho W-L, Chen J-T, Tang J-S, Yeh D-C, P'eng F-K. Hepatitis viral status in patients undergoing liver resection for hepatocellular carcinoma . Br J Surg. 1999; 86(11): 1391-6.
381	Weng Y-H, Chou Y-H, Lien R-I. Hyperbilirubinemia in healthy neonates with glucose-6-phosphate dehydrogenase deficiency. Early Hum Dev. 2003; 71(2): 129–36.
382	Institute for Health Metrics and Evaluation (IHME). IHME COVID-19 Error Patch Dataset.
383	Institute for Health Metrics and Evaluation (IHME). IHME GBD Cancer Incidence and Mortality Estimates.
384	Jeng J-E, Tsai H-R, Chuang L-Y, Tsai J-F, Lin Z-Y, Hsieh M-Y, Chen S-C, Chuang W-L, Wang L-Y, Yu M-L, Dai C-Y, Chang J-G. Independent and additive interactive effects among tumor necrosis factor-alpha polymorphisms, substance use habits, and chronic hepatitis B and hepatitis C virus infection on risk for hepatocellular carcinoma. Medicine (Baltimore). 2009; 88(6): 349-57.
385	Hung C-H, Wang J-H, Hu T-H, Chen C-H, Chang K-C, Yen Y-H, Kuo Y-H, Tsai M-C, Lu S-N, Lee C-M. Insulin resistance is associated with hepatocellular carcinoma in chronic hepatitis C infection. World J Gastroenterol. 2010; 16(18): 2265-71.
386	Wang L, Lee K, Lin C, Hsu L, Wang C, Hsu T, Ho W. Long-Term Survival of Patients with Pulmonary Arterial Hypertension at a Single Center in Taiwan. Acta Cardiol Sin. 2017; 33(5): 498-509.
387	Wu TP, Liang FW, Huang YL, Chen LH, Lu TH. Maternal Mortality in Taiwan: A Nationwide Data Linkage Study. PLoS One. 2015; 10(8): e0132547.
388	Dou H-Y, Tseng F-C, Lin C-W, Chang J-R, Sun J-R, Tsai W-S, Lee S-Y, Su I-J, Lu J-J. Molecular epidemiology and evolutionary genetics of Mycobacterium tuberculosis in Taipei. BMC Infect Dis. 2008; 8: 170.
389	Chen Y-Y, Chang J-R, Kuo S-C, Tseng F-C, Huang W-C, Huang T-S, Chen Y-S, Chiueh T-S, Sun J-R, Su I-J, Dou H-Y. Molecular epidemiology of tuberculosis in Kaohsiung City located at southern Taiwan, 2000-2008. PLoS One. 2015; 10(1): e0117061.
390	Sun CA, Farzadegan H, You SL, Lu SN, Wu MH, Wolfe L, Hardy W, Huang GT, Yang PM, Lee H, Chen CJ. Mutual confounding and interactive effects between hepatitis C and hepatitis B viral infections in hepatocellular carcinogenesis: a population-based case-control study in Taiwan . Cancer Epidemiol Biomark Prev. 1996; 5(3): 173-8.
391	Tung H-D, Wang J-H, Tseng P-L, Hung C-H, Kee K-M, Chen C-H, Chang K-C, Lee C-M, Changchien C-S, Chen Y-D, others. Neither diabetes mellitus nor overweight is a risk

	factor for hepatocellular carcinoma in a dual HBV and HCV endemic area: community cross-sectional and case-control studies . Am J Gastroenterol. 2010; 105(3): 624-31.
392	Chiang SH, Wu SJ, Wu KF, Hsiao KJ. Neonatal screening for glucose-6-phosphate dehydrogenase deficiency in Taiwan. Southeast Asian J Trop Med Public Health. 1999; 72-4.
393	Yen Y-F, Yen M-Y, Shih H-C, Hu B-S, Ho B-L, Li L-H, Hsiao J-C, Deng C-Y. Prognostic factors associated with mortality before and during anti-tuberculosis treatment. Int J Tuberc Lung Dis. 2013; 17(10): 1310–6.
394	Shiah H-S, Chen C-Y, Dai C-Y, Hsiao C-F, Lin Y-J, Su W-C, Chang J-Y, Whang-Peng J, Lin P-W, Huang J-D, Chen L-T. Randomised clinical trial: comparison of two everolimus dosing schedules in patients with advanced hepatocellular carcinoma . Aliment Pharmacol Ther. 2013; 37(1): 62-73.
395	Weatherall D. Sickle Cell and Thalassemias Prevalence Data, Personal Correspondence with David Weatherall. [Unpublished].
396	Chuang P-C, Liu H, Sola C, Chen Y-MA, Jou R. Spoligotypes of Mycobacterium tuberculosis isolates of a high tuberculosis burden aboriginal township in Taiwan. Infect Genet Evol. 2008; 8(5): 553–7.
397	Chen MF, Jeng LB, Lee WC, Chen TC. Surgical results in patients with dual hepatitis B- and C-related hepatocellular carcinoma compared with hepatitis B- or C-related hepatocellular carcinoma. Surgery. 1998; 123(5): 554-9.
398	Loomba R, Yang H-I, Su J, Brenner D, Barrett-Connor E, Iloeje U, Chen C-J. Synergism Between Obesity and Alcohol in Increasing the Risk of Hepatocellular Carcinoma: A Prospective Cohort Study . Am J Epidemiol. 2013; 177(4): 333-42.
399	Taiwan Cancer Registry. Taiwan Cancer Registry Incidence and Mortality 1980-2007.
400	Health Promotion Administration, Ministry of Health and Welfare (Taiwan), Taiwan Cancer Registry. Taiwan Cancer Registry Incidence and Population 2008-2016.
401	Centers for Disease Control, R.O.C (Taiwan). Taiwan HIV Statistics by Area, Age, and Gender 2008. Taipei, Taiwan (Province of China): Centers for Disease Control, R.O.C (Taiwan), 2019.
402	Centers for Disease Control, R.O.C (Taiwan). Taiwan HIV Statistics by Area, Age, and Gender 2009. Taipei, Taiwan (Province of China): Centers for Disease Control, R.O.C (Taiwan), 2019.
403	Centers for Disease Control, R.O.C (Taiwan). Taiwan HIV Statistics by Area, Age, and Gender 2010. Taipei, Taiwan (Province of China): Centers for Disease Control, R.O.C (Taiwan), 2019.
404	Centers for Disease Control, R.O.C (Taiwan). Taiwan HIV Statistics by Area, Age, and Gender 2011. Taipei, Taiwan (Province of China): Centers for Disease Control, R.O.C (Taiwan), 2019.
405	Centers for Disease Control, R.O.C (Taiwan). Taiwan HIV Statistics by Area, Age, and Gender 2012. Taipei, Taiwan (Province of China): Centers for Disease Control, R.O.C (Taiwan), 2019.
406	Centers for Disease Control, R.O.C (Taiwan). Taiwan HIV Statistics by Area, Age, and Gender 2013. Taipei, Taiwan (Province of China): Centers for Disease Control, R.O.C (Taiwan), 2019.
407	Centers for Disease Control, R.O.C (Taiwan). Taiwan HIV Statistics by Area, Age, and Gender 2014. Taipei, Taiwan (Province of China): Centers for Disease Control, R.O.C (Taiwan), 2019.
408	Centers for Disease Control, R.O.C (Taiwan). Taiwan HIV Statistics by Area, Age, and Gender 2015. Taipei, Taiwan (Province of China): Centers for Disease Control, R.O.C (Taiwan), 2019.
409	Centers for Disease Control, R.O.C (Taiwan). Taiwan HIV Statistics by Area, Age, and Gender 2016. Taipei, Taiwan (Province of China): Centers for Disease Control, R.O.C (Taiwan), 2019.
410	Centers for Disease Control, R.O.C (Taiwan). Taiwan HIV Statistics by Area, Age, and Gender 2017. Taipei, Taiwan (Province of China): Centers for Disease Control, R.O.C (Taiwan), 2019.

411	Centers for Disease Control, R.O.C (Taiwan). Taiwan HIV Statistics by Area, Age, and Gender 2018. Taipei, Taiwan (Province of China): Centers for Disease Control, R.O.C (Taiwan), 2019.
412	Centers for Disease Control, R.O.C (Taiwan). Taiwan HIV Statistics by Area, Age, and Gender 2019. Taipei, Taiwan (Province of China): Centers for Disease Control, R.O.C (Taiwan), 2019.
413	Ministry of the Interior (Taiwan), National Statistics Bureau (Taiwan). Taiwan Human Mortality Database Short-term Mortality Fluctuations (STMF). as it appears in Human Mortality Database Short-term Mortality Fluctuations (STMF)
414	Department of Household Registration, Ministry of the Interior (Taiwan). Taiwan Monthly Bulletin of Interior Statistics - Number and Rates of Birth, Death, Marriage and Divorce of the Current Resident Population. Taipei, Taiwan (Province of China): Department of Household Registration, Ministry of the Interior (Taiwan).
415	Ministry of Health and Welfare (Taiwan). Taiwan National Health Insurance Claims Data 2016.
416	Ministry of Health and Welfare (Taiwan). Taiwan Statistics of Causes of Death 2017. Taipei City, Taiwan (Province of China): Ministry of Health and Welfare (Taiwan), 2018.
417	Centers for Disease Control, R.O.C (Taiwan). Taiwan Syphilis Cases by Age Group, Sex, and Place of Living. Taipei, Taiwan (Province of China): Centers for Disease Control, R.O.C (Taiwan).
418	Department of Health (Taiwan). Taiwan Vital Registration - Deaths 1980.
419	Department of Health (Taiwan). Taiwan Vital Registration - Deaths 1981.
420	Department of Health (Taiwan). Taiwan Vital Registration - Deaths 1982.
421	Department of Health (Taiwan). Taiwan Vital Registration - Deaths 1983.
422	Department of Health (Taiwan). Taiwan Vital Registration - Deaths 1984.
423	Department of Health (Taiwan). Taiwan Vital Registration - Deaths 1985.
424	Department of Health (Taiwan). Taiwan Vital Registration - Deaths 1986.
425	Department of Health (Taiwan). Taiwan Vital Registration - Deaths 1987.
426	Department of Health (Taiwan). Taiwan Vital Registration - Deaths 1988.
427	Department of Health (Taiwan). Taiwan Vital Registration - Deaths 1989.
428	Department of Health (Taiwan). Taiwan Vital Registration - Deaths 1990.
429	Department of Health (Taiwan). Taiwan Vital Registration - Deaths 1991.
430	Department of Health (Taiwan). Taiwan Vital Registration - Deaths 1992. Taipei City, Taiwan (Province of China): Ministry of Health and Welfare (Taiwan).
431	Department of Health (Taiwan). Taiwan Vital Registration - Deaths 1993. Taipei City, Taiwan (Province of China): Ministry of Health and Welfare (Taiwan).
432	Department of Health (Taiwan). Taiwan Vital Registration - Deaths 1994. Taipei City, Taiwan (Province of China): Ministry of Health and Welfare (Taiwan).
433	Department of Health (Taiwan). Taiwan Vital Registration - Deaths 1995. Taipei City, Taiwan (Province of China): Ministry of Health and Welfare (Taiwan).
434	Department of Health (Taiwan). Taiwan Vital Registration - Deaths 1996. Taipei City, Taiwan (Province of China): Ministry of Health and Welfare (Taiwan).
435	Department of Health (Taiwan). Taiwan Vital Registration - Deaths 1997. Taipei City, Taiwan (Province of China): Ministry of Health and Welfare (Taiwan).
436	Department of Health (Taiwan). Taiwan Vital Registration - Deaths 1998. Taipei City, Taiwan (Province of China): Ministry of Health and Welfare (Taiwan).
437	Department of Health (Taiwan). Taiwan Vital Registration - Deaths 1999. Taipei City, Taiwan (Province of China): Ministry of Health and Welfare (Taiwan).
438	Department of Health (Taiwan). Taiwan Vital Registration - Deaths 2000. Taipei City, Taiwan (Province of China): Ministry of Health and Welfare (Taiwan).
439	Department of Health (Taiwan). Taiwan Vital Registration - Deaths 2001. Taipei City, Taiwan (Province of China): Ministry of Health and Welfare (Taiwan).
440	Department of Health (Taiwan). Taiwan Vital Registration - Deaths 2002. Taipei City, Taiwan (Province of China): Ministry of Health and Welfare (Taiwan).
441	Department of Health (Taiwan). Taiwan Vital Registration - Deaths 2003. Taipei City, Taiwan (Province of China): Ministry of Health and Welfare (Taiwan).

442	Department of Health (Taiwan). Taiwan Vital Registration - Deaths 2004. Taipei City, Taiwan (Province of China): Ministry of Health and Welfare (Taiwan).
443	Department of Health (Taiwan). Taiwan Vital Registration - Deaths 2005. Taipei City, Taiwan (Province of China): Ministry of Health and Welfare (Taiwan).
444	Department of Health (Taiwan). Taiwan Vital Registration - Deaths 2006. Taipei City, Taiwan (Province of China): Ministry of Health and Welfare (Taiwan).
445	Department of Health (Taiwan). Taiwan Vital Registration - Deaths 2007. Taipei City, Taiwan (Province of China): Ministry of Health and Welfare (Taiwan).
446	Department of Health (Taiwan). Taiwan Vital Registration - Deaths 2008. Taipei City, Taiwan (Province of China): Ministry of Health and Welfare (Taiwan).
447	Department of Health (Taiwan). Taiwan Vital Registration - Deaths 2009. Taipei City, Taiwan (Province of China): Ministry of Health and Welfare (Taiwan).
448	Department of Health (Taiwan). Taiwan Vital Registration - Deaths 2010. Taipei City, Taiwan (Province of China): Ministry of Health and Welfare (Taiwan).
449	Department of Health (Taiwan). Taiwan Vital Registration - Deaths 2011. Taipei City, Taiwan (Province of China): Ministry of Health and Welfare (Taiwan).
450	Department of Health (Taiwan). Taiwan Vital Registration - Deaths 2012. Taipei City, Taiwan (Province of China): Ministry of Health and Welfare (Taiwan).
451	Ministry of Health and Welfare (Taiwan). Taiwan Vital Registration - Deaths 2013. Taipei City, Taiwan (Province of China): Ministry of Health and Welfare (Taiwan).
452	Ministry of Health and Welfare (Taiwan). Taiwan Vital Registration - Deaths 2014. Taipei City, Taiwan (Province of China): Ministry of Health and Welfare (Taiwan).
453	Ministry of Health and Welfare (Taiwan). Taiwan Vital Registration - Deaths 2015. Taipei City, Taiwan (Province of China): Ministry of Health and Welfare (Taiwan).
454	Ministry of Health and Welfare (Taiwan). Taiwan Vital Registration - Deaths 2016. Taipei City, Taiwan (Province of China): Ministry of Health and Welfare (Taiwan).
455	Pew Research Center. The Future of the Global Muslim Population. Washington, DC, United States: Pew Research Center, 2011.
456	Chang IC, Huang SF, Chen PJ, Chen CL, Chen CL, Wu CC, Tsai CC, Lee PH, Chen MF, Lee CM, Yu HC, Lo GH, Yeh CT, Hong CC, Eng HL, Wang J, Tseng HH, Hsiao CH, Wu HD, Yen TC, Liaw YF. The Hepatitis Viral Status in Patients With Hepatocellular Carcinoma: a Study of 3843 Patients From Taiwan Liver Cancer Network. Medicine (Baltimore). 2016; 95(15): e3284.
457	Wang C-S, Yao W-J, Chang T-T, Wang S-T, Chou P. The Impact of Type 2 Diabetes on the Development of Hepatocellular Carcinoma in Different Viral Hepatitis Statuses . Cancer Epidemiol Biomarkers Prev. 2009; 18(7): 2054-60.
458	Jeng J-E, Tsai J-F, Chuang L-Y, Ho M-S, Lin Z-Y, Hsieh M-Y, Chen S-C, Chuang W-L, Wang L-Y, Yu M-L, Dai C-Y, Chang J-G. Tumor necrosis factor-alpha 308.2 polymorphism is associated with advanced hepatic fibrosis and higher risk for hepatocellular carcinoma. Neoplasia. 2007; 9(11): 987-92.
459	Tseng C-S, Tang K-S, Lo H-W, Ker C-G, Teng H-C, Huang C-S. UDP-Glucuronosyltransferase 1A7 Genetic Polymorphisms Are Associated with Hepatocellular Carcinoma Risk and Onset Age . Am J Gastroenterol. 2005; 100(8): 1758-63.
460	United Nations Office on Drugs and Crime (UNODC). United Nations Office on Drugs and Crime Global Study on Homicide 2011. Vienna, Austria: United Nations Office on Drugs and Crime (UNODC), 2011.
461	Jiang J, Ma X, Song C, Lin B, Cao W, Wu S, Hsiao K-J. Using the fluorescence spot test for neonatal screening of G6PD deficiency. Southeast Asian J Trop Med Public Health. 2003; 140-2.
462	World Health Organization (WHO). WHO International Travel and Health, Annex 1, Countries With Risk of Yellow Fever Transmission and Countries Requiring Yellow Fever Vaccination 2017 Update. Geneva, Switzerland: World Health Organization (WHO), 2017.
463	World Health Organization (WHO). WHO Mortality Database Version February 2014. Geneva, Switzerland: World Health Organization (WHO).
