# Supplementary material for: The disability-adjusted life years attributable to mental disorders and self-harm in China from 1990–2021: Findings from the global burden of disease study 2021
Source: PLOS Ment Health. 2025 Apr 9;2(4):e0000146. doi: 10.1371/journal.pmen.0000146 (PMC12798377; doi:10.1371/journal.pmen.0000146)
Supplement: S1 Table — (PDF) [file pmen.0000146.s004.pdf]

Table S1 Changing DALYs of all mental disorders and self-harm in China in 5 years age group, 1990-2021.

	DALY numbers [95% UI]			Age-standardised DALY rates [95% UI]		
	2021	1990 vs. 2021	2019 vs. 2021	2021	1990 vs. 2021	2019 vs. 2021
self-harm						
<5	-	-	-		-	-
5-9	-	-	-	-	-	-
10-15	50873 [42473, 66770]	-83.3% [-86.9, -73.3]	-3.3% [-15.1, 10.2]	59.0 [49.3, 77.5]	-80.2% [-84.5, -68.3]	-6.4% [-17.9, 6.6]
15-19	150703 [123046, 186318]	-87.1% [-89.8, -78.6]	-0.8% [-18.4, 20.5]	201.8 [164.8, 249.5]	-78.1% [-82.6, -63.7]	-3.9% [-21, 16.7]
20-24	265497 [215763, 335121]	-82.8% [-87.1, -72.2]	-6.7% [-24.6, 15.8]	362.8 [294.9, 458.0]	-68.9% [-76.7, -49.8]	-0.3% [-19.5, 23.7]
25-29	296442 [246994, 367277]	-75.2% [-80.9, -58.2]	-13.6% [-27.7, 4.7]	342.8 [285.6, 424.7]	-68.5% [-75.7, -46.9]	0.3% [-16.1, 21.4]
30-34	411069 [339546, 514046]	-59.2% [-68, -32.3]	-1.2% [-17.9, 19.7]	339.3 [280.3, 424.3]	-70.2% [-76.7, -50.7]	1.6% [-15.6, 23.1]
35-39	357821 [294666, 446429]	-68.5% [-75.3, -46.5]	10.7% [-9.7, 36.8]	337.7 [278.1, 421.3]	-72.9% [-78.7, -53.9]	-0.3% [-18.7, 23.2]
40-44	314751 [253812, 398342]	-61.5% [-70.4, -37.2]	-6.2% [-26, 18.2]	343.9 [277.3, 435.2]	-71.8% [-78.3, -54]	-0.8% [-21.8, 24.9]
45-49	346641 [276378, 447146]	-39% [-54.7, -4.2]	-7.3% [-27.9, 18.6]	314.2 [250.5, 405.3]	-71.5% [-78.8, -55.2]	-0.8% [-22.8, 26.9]
50-54	427590 [340639, 545038]	-27.9% [-45.9, 9.2]	-1.9% [-22.9, 25.3]	353.8 [281.8, 451.0]	-71.5% [-78.6, -56.9]	-0.7% [-22, 26.8]
55-59	421376 [339290, 527592]	-20.4% [-38.4, 16.6]	18.6% [-7.3, 51]	383.3 [308.6, 479.9]	-68.6% [-75.7, -54]	0.4% [-21.5, 27.8]
60-64	290466 [235535, 357654]	-28% [-44.9, 3.9]	-7.6% [-27.1, 15.8]	397.9 [322.6, 489.9]	-65.1% [-73.3, -49.7]	-2.4% [-23, 22.3]
65-69	327275 [267232, 405199]	0.9% [-20, 45.4]	10.3% [-12.2, 37.2]	426.7 [348.4, 528.3]	-64.1% [-71.6, -48.3]	0.2% [-20.3, 24.7]
70-74	288931 [232950, 356953]	7.3% [-15.8, 56.6]	10.3% [-10.8, 34.8]	542.1 [437.1, 669.8]	-62.1% [-70.3, -44.7]	-1.2% [-20, 20.8]
75-79	198157 [163479, 238995]	17.7% [-5.9, 75.3]	9.4% [-10.2, 33.4]	598.3 [493.6, 721.6]	-59.5% [-67.7, -39.7]	0.2% [-17.7, 22.2]
80-84	130191 [107356, 155096]	59.4% [30.3, 141.1]	6% [-10.1, 25.2]	657.8 [542.4, 783.6]	-57.3% [-65.1, -35.5]	0.9% [-14.4, 19.2]
85-89	75543 [62189, 91896]	158.1% [115.2, 296.2]	14.1% [0.6, 31.7]	793.0 [652.8, 964.7]	-54.3% [-61.9, -29.8]	2% [-10.1, 17.8]
90-94	19499 [15906, 23201]	324.1% [250.7, 535.9]	14.9% [0.2, 31.6]	665.1 [542.5, 791.3]	-55.6% [-63.3, -33.5]	0.5% [-12.4, 15.1]
95	3043 [2346, 3741]	653.3% [515.2, 1056.2]	17.5% [3.8, 36.3]	476.1 [367.1, 585.4]	-52.3% [-61, -26.7]	-0.6% [-12.3, 15.2]
Mental disorders						
<5	135652 [100215, 181593]	-29.6% [-33.1, -26.2]	-11.7% [-14.2, -9.1]	174.7 [129.0, 233.8]	1.3% [-3.7, 6.3]	0.1% [-2.8, 3]
5-9	530763 [367603, 702723]	-4.2% [-8.5, 0.2]	8.1% [3.5, 12.7]	554.2 [383.8, 733.8]	4.3% [-0.4, 9.1]	-2.5% [-6.6, 1.7]
10-15	1059560 [727187, 1437973]	-12.8% [-16.1, -9.2]	1% [-3.4, 4.5]	1229.3 [843.7, 1668.3]	3.5% [-0.5, 7.8]	-2.3% [-6.5, 1.1]
15-19	1067206 [770830, 1415236]	-42.6% [-45.1, -40]	4.8% [1, 8.5]	1429.2 [1032.3, 1895.3]	-2.6% [-6.9, 1.8]	1.5% [-2.1, 5.2]

20-24	1078512 [778429, 1409139]	-49.5% [-52.2, -46.7]	0.9% [-3.2, 4.8]	1473.9 [1063.8, 1925.7]	-8.9% [-13.8, -3.8]	7.8% [3.4, 12]
25-29	1386459 [1031157, 1796443]	-27.5% [-30.3, -24.6]	-6.7% [-10.1, -3.2]	1603.2 [1192.3, 2077.2]	-7.9% [-11.5, -4.2]	8.3% [4.3, 12.3]
30-34	2083811 [1547651, 2691828]	29.3% [24.6, 34.8]	4.1% [1, 7.5]	1720.0 [1277.4, 2221.8]	-5.8% [-9.2, -1.8]	7.1% [3.9, 10.6]
35-39	1909000 [1436129, 2450630]	10% [6.3, 14.1]	17.4% [14.2, 20.9]	1801.6 [1355.3, 2312.7]	-5.2% [-8.4, -1.7]	5.8% [2.9, 8.9]
40-44	1708091 [1273284, 2188680]	31.6% [27.1, 35.6]	-1.4% [-4.1, 1.2]	1866.1 [1391.1, 2391.1]	-3.6% [-6.8, -0.6]	4.2% [1.3, 7]
45-49	2149181 [1612920, 2732956]	114.8% [108.4, 121.2]	-3.1% [-5.6, -0.7]	1948.1 [1462.0, 2477.3]	0.5% [-2.5, 3.5]	3.7% [1, 6.3]
50-54	2434753 [1813765, 3110790]	164.3% [156.1, 172.7]	2.2% [-0.3, 4.9]	2014.5 [1500.7, 2573.9]	4.4% [1.1, 7.7]	3.4% [0.9, 6.2]
55-59	2276194 [1701508, 2937755]	174.2% [166.1, 183.2]	22.6% [19.4, 26.3]	2070.4 [1547.6, 2672.1]	8.2% [5, 11.7]	3.8% [1.1, 6.9]
60-64	1519287 [1121621, 1989666]	127.6% [120.2, 136.4]	-2.3% [-5.3, 0.6]	2081.1 [1536.4, 2725.4]	10.2% [6.6, 14.4]	3.2% [0.1, 6.3]
65-69	1572379 [1180770, 2033197]	209.6% [199.2, 220]	12.6% [9.3, 16.2]	2049.9 [1539.4, 2650.7]	10.1% [6.4, 13.8]	2.3% [-0.7, 5.6]
70-74	1063481 [793656, 1370386]	212.4% [202.1, 224.2]	13.3% [10, 16.9]	1995.4 [1489.1, 2571.3]	10.3% [6.7, 14.5]	1.5% [-1.4, 4.7]
75-79	638438 [459420, 828957]	219.8% [207.2, 231.8]	9.3% [6, 12.8]	1927.7 [1387.2, 2503.0]	9.9% [5.6, 14]	0.2% [-2.9, 3.3]
80-84	367190 [269076, 477048]	305.5% [288.9, 322.1]	4.1% [0.8, 8]	1855.3 [1359.5, 2410.3]	8.5% [4.1, 13]	-1% [-4.1, 2.8]
85-89	168920 [126054, 218366]	495.7% [473.8, 519]	10.4% [6.8, 14.6]	1773.3 [1323.3, 2292.4]	5.5% [1.6, 9.6]	-1.3% [-4.5, 2.5]
90-94	49715 [36072, 64965]	878.9% [841.7, 920]	12.6% [8.4, 16.9]	1695.6 [1230.3, 2215.7]	2.4% [-1.5, 6.7]	-1.6% [-5.2, 2.2]
95	10357 [7325, 13947]	1464.7% [1392.6, 1541.1]	16.3% [11.2, 21.1]	1620.5 [1146.1, 2182.3]	-0.9% [-5.4, 4]	-1.7% [-6, 2.4]
Depressive disorders						
<5	133 [55, 249]	-16.9% [-21.8, -11.6]	-5.4% [-11.9, 1.1]	0.2 [0.1, 0.3]	19.6% [12.6, 27.2]	7.2% [-0.1, 14.6]
5-9	10221 [5119, 17714]	-7.5% [-16.6, 1.5]	4.7% [-6.1, 15.3]	10.7 [5.3, 18.5]	0.7% [-9.2, 10.6]	-5.5% [-15.3, 4]
10-15	78053 [45356, 119348]	-29.8% [-36.8, -22.7]	-10.9% [-17.3, -3.8]	90.6 [52.6, 138.5]	-16.7% [-25, -8.2]	-13.8% [-20, -6.9]
15-19	180086 [113026, 266792]	-59.3% [-62.9, -55.5]	-5.7% [-11.7, 0.2]	241.2 [151.4, 357.3]	-30.9% [-37, -24.6]	-8.6% [-14.4, -2.8]
20-24	253125 [162121, 374929]	-64.9% [-67.9, -61.8]	-6.8% [-11.5, -1.7]	345.9 [221.6, 512.4]	-36.7% [-42.1, -31.2]	-0.4% [-5.4, 5]
25-29	337214 [222055, 488393]	-47% [-50.5, -43.1]	-12.8% [-17.3, -7.9]	389.9 [256.8, 564.7]	-32.6% [-37, -27.7]	1.1% [-4, 6.8]
30-34	539245 [347745, 785128]	0.3% [-6.6, 7.5]	-0.6% [-5.6, 4.3]	445.1 [287.0, 648.0]	-26.9% [-31.9, -21.7]	2.2% [-2.9, 7.3]
35-39	546081 [355656, 762797]	-10.1% [-16.5, -3.5]	14.8% [9.6, 20.2]	515.4 [335.6, 719.9]	-22.5% [-28.1, -16.8]	3.4% [-1.2, 8.2]
40-44	553254 [357449, 784147]	15.9% [7.9, 24.3]	-1.9% [-6.6, 3.2]	604.4 [390.5, 856.7]	-15% [-20.9, -8.9]	3.7% [-1.3, 9]
45-49	787628 [541852, 1076194]	107.3% [94.1, 118.9]	-3.1% [-7.4, 1.1]	713.9 [491.2, 975.5]	-3% [-9.2, 2.4]	3.8% [-0.9, 8.2]
50-54	981901 [683774, 1347951]	173.2% [156.4, 189.6]	1.5% [-2.9, 5.7]	812.4 [565.8, 1115.3]	7.8% [1.2, 14.3]	2.8% [-1.7, 6.9]
55-59	991147 [678227, 1358838]	197.9% [180.8, 216.9]	21% [15.5, 26.3]	901.5 [616.9, 1236.0]	17.5% [10.8, 25]	2.4% [-2.2, 6.9]

60-64	700978 [481567, 969040]	152% [137.1, 167.7]	-4.8% [-9.2, -0.2]	960.2 [659.6, 1327.4]	22% [14.8, 29.6]	0.6% [-4, 5.5]
65-69	752422 [529075, 1030625]	238.9% [221.2, 260.8]	8.3% [3.7, 13.2]	980.9 [689.8, 1343.6]	20.5% [14.2, 28.3]	-1.6% [-5.8, 2.8]
70-74	523222 [363317, 711484]	238.8% [222.2, 258.7]	8% [3.1, 13.1]	981.7 [681.7, 1335.0]	19.6% [13.8, 26.6]	-3.2% [-7.7, 1.4]
75-79	320951 [218827, 448428]	244% [226.5, 263.7]	3.6% [-1.2, 8.5]	969.1 [660.7, 1354.0]	18.2% [12.2, 25]	-5.1% [-9.4, -0.6]
80-84	187799 [129914, 261602]	328.6% [304.1, 353.6]	-1.7% [-6.1, 3.1]	948.9 [656.4, 1321.8]	14.7% [8.2, 21.4]	-6.5% [-10.7, -1.9]
85-89	89096 [63571, 122817]	511.5% [478.3, 548.4]	4.9% [0, 10.2]	935.3 [667.4, 1289.3]	8.3% [2.4, 14.8]	-6.2% [-10.6, -1.5]
90-94	27384 [19173, 38567]	874.2% [822.6, 935.2]	7.7% [2.2, 13.5]	934.0 [653.9, 1315.4]	1.9% [-3.5, 8.3]	-5.9% [-10.7, -0.8]
95	6002 [3638, 8908]	1445.6% [1338.5, 1565.1]	12.1% [5.1, 19.2]	939.2 [569.2, 1393.9]	-2.1% [-8.9, 5.5]	-5.2% [-11.1, 0.8]
Major depressive						
<5	103 [38, 214]	-17% [-22.4, -10.9]	-4.5% [-11.8, 2.4]	0.1 [0.0, 0.3]	19.5% [11.7, 28.2]	8.2% [-0.1, 16.1]
5-9	7982 [3298, 15093]	-9.3% [-19.6, 1.6]	3.9% [-7.4, 14.8]	8.3 [3.4, 15.8]	-1.2% [-12.5, 10.7]	-6.3% [-16.4, 3.6]
10-15	64421 [35432, 99293]	-32.5% [-40.5, -24.7]	-13% [-20.5, -4.8]	74.7 [41.1, 115.2]	-19.9% [-29.3, -10.6]	-15.9% [-23.1, -7.9]
15-19	145538 [87816, 226398]	-62% [-65.7, -58]	-7.4% [-14.3, -0.2]	194.9 [117.6, 303.2]	-35.5% [-41.8, -28.8]	-10.2% [-16.9, -3.2]
20-24	192258 [118543, 296324]	-68.4% [-71.6, -65.5]	-7.2% [-12.8, -0.6]	262.7 [162.0, 405.0]	-43% [-48.8, -37.7]	-0.9% [-6.8, 6.2]
25-29	230839 [144099, 354941]	-53.6% [-57.3, -50]	-13.8% [-19.5, -6.9]	266.9 [166.6, 410.4]	-41% [-45.7, -36.4]	0% [-6.6, 8]
30-34	337289 [203405, 532300]	-13.5% [-19.8, -6.2]	-2.8% [-8.7, 3.7]	278.4 [167.9, 439.4]	-37% [-41.6, -31.7]	-0.1% [-6.1, 6.6]
35-39	320716 [196701, 484617]	-22.6% [-30.6, -14.2]	10.7% [3.4, 18.5]	302.7 [185.6, 457.3]	-33.3% [-40.2, -26.1]	-0.3% [-6.9, 6.7]
40-44	318759 [190753, 481466]	3.8% [-6.6, 14.1]	-6.4% [-12, -0.5]	348.2 [208.4, 526.0]	-23.9% [-31.5, -16.3]	-1.1% [-7, 5.1]
45-49	472534 [301405, 683118]	101.4% [83.4, 119.6]	-7.1% [-12.4, -1.7]	428.3 [273.2, 619.2]	-5.8% [-14.2, 2.8]	-0.6% [-6.2, 5.2]
50-54	613670 [394285, 875979]	184% [160.2, 207.9]	-2% [-7.3, 4.5]	507.8 [326.2, 724.8]	12.1% [2.7, 21.6]	-0.8% [-6.2, 5.7]
55-59	640899 [407167, 921361]	227.2% [202.5, 256.4]	17.8% [11.1, 25.3]	582.9 [370.3, 838.0]	29.1% [19.3, 40.6]	-0.3% [-5.9, 6.1]
60-64	464482 [293993, 651249]	183% [161.3, 207.9]	-7.1% [-12.2, -1.1]	636.2 [402.7, 892.1]	37% [26.5, 49]	-1.8% [-7.3, 4.5]
65-69	517000 [343023, 708607]	274.6% [250.6, 301]	5.6% [0.1, 11.9]	674.0 [447.2, 923.8]	33.2% [24.7, 42.6]	-4% [-9, 1.6]
70-74	377701 [257118, 521107]	268.2% [246.6, 294]	5.5% [-0.1, 11.8]	708.7 [482.4, 977.8]	30% [22.4, 39.1]	-5.5% [-10.5, 0.1]
75-79	244137 [157199, 346566]	267.9% [246, 293.8]	1.4% [-4.6, 7.2]	737.2 [474.7, 1046.4]	26.4% [18.9, 35.3]	-7.1% [-12.6, -1.7]
80-84	149068 [98763, 212549]	349.7% [322.1, 381.4]	-3.5% [-8.9, 2.5]	753.2 [499.0, 1073.9]	20.4% [13, 28.8]	-8.1% [-13.3, -2.4]
85-89	72000 [50143, 103081]	529.1% [489, 575.6]	3.6% [-2.1, 9.9]	755.8 [526.4, 1082.1]	11.4% [4.3, 19.6]	-7.4% [-12.5, -1.7]
90-94	22210 [14751, 32651]	885.9% [824.3, 955.4]	6.6% [0.5, 13.4]	757.5 [503.1, 1113.6]	3.2% [-3.3, 10.4]	-6.8% [-12.1, -0.8]
95	4858 [2783, 7731]	1451.1% [1321.7, 1591.8]	11.2% [3.8, 19]	760.1 [435.5, 1209.6]	-1.7% [-9.9, 7.2]	-6% [-12.2, 0.6]

Dysthymia						
<5	30 [9, 57]	-16.7% [-28.9, 1.1]	-8.5% [-22.2, 7.4]	0.0 [0.0, 0.1]	19.9% [2.3, 45.5]	3.7% [-11.8, 21.7]
5-9	2239 [1082, 3802]	-0.8% [-23.2, 31.6]	7.7% [-18.9, 39.9]	2.3 [1.1, 4.0]	8% [-16.4, 43.3]	-2.9% [-26.9, 26.2]
10-15	13632 [7341, 22516]	-13.6% [-22.4, -3.5]	0.8% [-9.7, 11.3]	15.8 [8.5, 26.1]	2.6% [-7.9, 14.5]	-2.4% [-12.6, 7.7]
15-19	34548 [20505, 54269]	-41.9% [-46.2, -37.4]	2.2% [-5.3, 9.9]	46.3 [27.5, 72.7]	-1.4% [-8.7, 6.3]	-0.9% [-8.2, 6.5]
20-24	60867 [36205, 94004]	-46.1% [-50.1, -41.3]	-5.4% [-12.9, 2.5]	83.2 [49.5, 128.5]	-2.8% [-10, 6]	1.1% [-7, 9.5]
25-29	106375 [65497, 157722]	-23.1% [-28.6, -17.4]	-10.7% [-17.1, -3.8]	123.0 [75.7, 182.4]	-2.3% [-9.2, 4.9]	3.6% [-3.8, 11.6]
30-34	201956 [128354, 290648]	36.7% [27.6, 45]	3.3% [-3.8, 10.5]	166.7 [105.9, 239.9]	-0.4% [-7, 5.6]	6.2% [-1, 13.7]
35-39	225365 [141502, 327543]	16.5% [9.8, 24.7]	21.4% [13, 30.2]	212.7 [133.5, 309.1]	0.5% [-5.3, 7.5]	9.3% [1.8, 17.3]
40-44	234494 [142665, 350329]	37.8% [28.6, 49.3]	5% [-3, 14.4]	256.2 [155.9, 382.7]	1% [-5.7, 9.4]	11% [2.5, 20.8]
45-49	315094 [197504, 469955]	116.7% [101.8, 134.1]	3.7% [-4.1, 11.2]	285.6 [179.0, 426.0]	1.4% [-5.6, 9.5]	11% [2.7, 19.1]
50-54	368231 [236640, 541606]	156.8% [140.6, 173.2]	8.1% [1.2, 14.5]	304.7 [195.8, 448.1]	1.4% [-5, 7.9]	9.4% [2.5, 15.8]
55-59	350248 [228579, 511520]	155.9% [140.6, 172]	27.3% [20.1, 35.2]	318.6 [207.9, 465.3]	1% [-5.1, 7.3]	7.8% [1.7, 14.5]
60-64	236496 [150822, 353884]	107.3% [92.7, 121.9]	-0.1% [-7.8, 7.9]	323.9 [206.6, 484.7]	0.3% [-6.7, 7.4]	5.6% [-2.6, 14]
65-69	235421 [148012, 352225]	180.3% [159.4, 201.6]	14.6% [5.7, 24.4]	306.9 [193.0, 459.2]	-0.3% [-7.7, 7.3]	4.1% [-4, 13]
70-74	145522 [91509, 217847]	180.7% [162.1, 200.3]	15% [7.2, 24.2]	273.0 [171.7, 408.7]	-0.9% [-7.4, 6]	3.1% [-3.9, 11.3]
75-79	76814 [49083, 114965]	185.1% [165.8, 206.6]	11.2% [4.1, 19.7]	231.9 [148.2, 347.1]	-2% [-8.7, 5.3]	1.9% [-4.6, 9.7]
80-84	38731 [24251, 60014]	263.2% [234.3, 294.5]	5.5% [-2.6, 15.1]	195.7 [122.5, 303.2]	-2.8% [-10.5, 5.6]	0.4% [-7.3, 9.6]
85-89	17096 [11072, 25982]	447.1% [403.9, 498.8]	11% [1.9, 21.3]	179.5 [116.2, 272.8]	-3.1% [-10.8, 6]	-0.8% [-8.9, 8.5]
90-94	5174 [3267, 7864]	827.2% [754.4, 915.9]	12.6% [4.6, 22.4]	176.5 [111.4, 268.2]	-3% [-10.6, 6.3]	-1.5% [-8.5, 7.1]
95	1145 [661, 1799]	1422.4% [1277, 1589.4]	16.3% [5.3, 28.3]	179.1 [103.4, 281.5]	-3.5% [-12.8, 7]	-1.7% [-11, 8.5]
Anxiety disorders						
<5	12934 [7661, 19591]	-17.5% [-29.2, -3.1]	-12.8% [-25.3, 0.6]	16.7 [9.9, 25.2]	18.8% [1.9, 39.5]	-1.1% [-15.3, 14.1]
5-9	207606 [127762, 308542]	-3.9% [-11.5, 3.7]	4.2% [-5.4, 13.5]	216.8 [133.4, 322.2]	4.6% [-3.6, 12.9]	-6% [-14.7, 2.4]
10-15	457719 [276528, 677670]	-12.2% [-18.4, -6.1]	0.5% [-8.5, 8.4]	531.0 [320.8, 786.2]	4.2% [-3.1, 11.4]	-2.8% [-11.5, 4.9]
15-19	447601 [285309, 665602]	-39.2% [-43, -35]	10.2% [1.9, 18.6]	599.4 [382.1, 891.4]	3.1% [-3.3, 10.2]	6.8% [-1.2, 15]
20-24	373935 [234047, 552320]	-44% [-47.3, -40.2]	15.8% [5.2, 26.1]	511.0 [319.9, 754.8]	1% [-5, 7.9]	23.7% [12.4, 34.7]
25-29	394863 [244403, 588572]	-21.1% [-26.2, -15.2]	12.4% [2.4, 22.7]	456.6 [282.6, 680.6]	0.2% [-6.2, 7.8]	30.4% [18.7, 42.3]
30-34	541597 [356485, 792563]	41.6% [33.2, 51.4]	23.5% [12.9, 34.8]	447.0 [294.2, 654.2]	3.1% [-3, 10.3]	27% [16.1, 38.7]

35-39	470265 [304111, 681107]	18.8% [10.6, 27.5]	32.9% [23, 44.1]	443.8 [287.0, 642.8]	2.4% [-4.7, 9.9]	19.7% [10.8, 29.8]
40-44	408758 [246853, 610684]	39.1% [30.6, 48.8]	5% [-1.7, 12.8]	446.6 [269.7, 667.2]	2% [-4.3, 9.1]	11% [3.9, 19.2]
45-49	507958 [318218, 753517]	119.9% [106.1, 134.3]	1.9% [-5.1, 8.9]	460.4 [288.4, 683.0]	2.9% [-3.5, 9.6]	9% [1.6, 16.6]
50-54	574986 [375961, 820446]	160.9% [144.4, 179.4]	7.3% [0.4, 15.2]	475.7 [311.1, 678.8]	3% [-3.5, 10.3]	8.6% [1.6, 16.6]
55-59	541990 [361783, 773572]	161.3% [143.8, 179.7]	31.6% [23.2, 40.6]	493.0 [329.1, 703.6]	3.1% [-3.8, 10.3]	11.4% [4.3, 19]
60-64	366697 [240406, 541937]	109.9% [96.1, 124.3]	6.5% [-0.6, 13.7]	502.3 [329.3, 742.3]	1.6% [-5.1, 8.6]	12.6% [5, 20.1]
65-69	394541 [257326, 577560]	184.4% [165.4, 206.2]	24.3% [15.7, 33.8]	514.4 [335.5, 753.0]	1.2% [-5.6, 8.9]	12.9% [5.1, 21.6]
70-74	276605 [186638, 391068]	185.6% [165.8, 206.4]	25.9% [17.5, 34.5]	519.0 [350.2, 733.8]	0.8% [-6.1, 8.2]	12.8% [5.2, 20.6]
75-79	172386 [116915, 240307]	191.9% [172.6, 210.8]	21.7% [13.9, 30.1]	520.5 [353.0, 725.6]	0.3% [-6.3, 6.8]	11.5% [4.4, 19.3]
80-84	102402 [70599, 143531]	273.5% [249.4, 299.5]	15.4% [8, 24.2]	517.4 [356.7, 725.2]	0% [-6.5, 6.9]	9.9% [2.8, 18.2]
85-89	46487 [31174, 65241]	463.3% [424.7, 505.7]	21.3% [13.4, 29.5]	488.0 [327.3, 684.9]	-0.3% [-7.1, 7.3]	8.5% [1.4, 15.8]
90-94	12795 [8426, 17924]	869.7% [804.4, 938]	23.2% [13.9, 32.7]	436.4 [287.4, 611.3]	1.5% [-5.4, 8.6]	7.7% [-0.4, 16]
95	2324 [1541, 3343]	1449.9% [1334, 1572.3]	26.8% [16.3, 38.1]	363.7 [241.1, 523.1]	-1.8% [-9.1, 6]	7.2% [-1.7, 16.7]
Schizophrenia						
<5	-	-	-	-	-	-
5-9	-	-	-	-	-	-
10-15	7259 [3941, 11649]	-9.7% [-16, -2.7]	4.6% [-3.5, 13.4]	8.4 [4.6, 13.5]	7.2% [-0.3, 15.4]	1.2% [-6.6, 9.8]
15-19	48651 [30780, 70526]	-37.8% [-45.6, -30.1]	4.7% [-4, 14.2]	65.2 [41.2, 94.4]	5.5% [-7.7, 18.6]	1.5% [-7, 10.7]
20-24	156609 [99347, 229082]	-42% [-45.7, -38.1]	-4.9% [-11.2, 1]	214.0 [135.8, 313.1]	4.6% [-2.1, 11.6]	1.5% [-5.1, 7.8]
25-29	294309 [208613, 404707]	-18% [-22, -13.6]	-12.5% [-16.5, -8.4]	340.3 [241.2, 468.0]	4.2% [-0.9, 9.8]	1.5% [-3.1, 6.2]
30-34	482450 [341625, 634129]	43.8% [37.7, 50]	-1.4% [-5.6, 2.5]	398.2 [282.0, 523.4]	4.7% [0.3, 9.2]	1.4% [-2.9, 5.4]
35-39	438606 [305317, 574952]	21.9% [16.9, 27.2]	12.7% [8.3, 17]	413.9 [288.1, 542.6]	5.1% [0.8, 9.7]	1.5% [-2.4, 5.4]
40-44	368210 [265321, 466616]	42.6% [35.7, 48.9]	-3.8% [-7.3, 0.1]	402.3 [289.9, 509.8]	4.5% [-0.5, 9.2]	1.7% [-2, 5.8]
45-49	414243 [300234, 526345]	121.8% [111.7, 131.7]	-5% [-8.8, -1.3]	375.5 [272.1, 477.1]	3.8% [-1, 8.4]	1.6% [-2.4, 5.7]
50-54	412007 [299874, 521005]	161.8% [148.9, 174.8]	0.5% [-4.4, 5.3]	340.9 [248.1, 431.1]	3.3% [-1.7, 8.5]	1.7% [-3.2, 6.5]
55-59	327680 [240727, 412730]	159.4% [146.6, 173.3]	19.9% [14.5, 25.3]	298.0 [219.0, 375.4]	2.3% [-2.7, 7.8]	1.6% [-3, 6.1]
60-64	184028 [134401, 231864]	111.7% [99.9, 124.1]	-3.9% [-8.6, 0.7]	252.1 [184.1, 317.6]	2.5% [-3.2, 8.5]	1.5% [-3.4, 6.4]
65-69	157654 [116266, 201715]	189.6% [172.9, 208.6]	11.9% [6.6, 18]	205.5 [151.6, 263.0]	3% [-2.9, 9.8]	1.6% [-3.2, 7.2]
70-74	86969 [64314, 109809]	194.8% [174.4, 214.9]	13.8% [7.9, 20.1]	163.2 [120.7, 206.0]	4.1% [-3.1, 11.2]	2% [-3.3, 7.6]

75-79	41150 [30215, 51654]	202.7% [180.1, 224.8]	10.5% [4.3, 17.7]	124.2 [91.2, 156.0]	4% [-3.8, 11.6]	1.3% [-4.4, 7.8]
80-84	18106 [13272, 22538]	296.2% [256.7, 335.2]	6.2% [-1.8, 14.9]	91.5 [67.1, 113.9]	6% [-4.5, 16.5]	1.1% [-6.6, 9.4]
85-89	6315 [4625, 8209]	526.1% [458.5, 599.5]	12.8% [3.3, 24.5]	66.3 [48.6, 86.2]	10.9% [-1.1, 23.9]	0.8% [-7.7, 11.3]
90-94	1390 [997, 1786]	1039.3% [887.7, 1200.3]	16.2% [3.8, 30.8]	47.4 [34.0, 60.9]	19.2% [3.4, 36.1]	1.6% [-9.3, 14.4]
95	210 [145, 286]	2024.6% [1688.6, 2506.1]	20.1% [2.4, 42.7]	32.9 [22.7, 44.7]	34.6% [13.3, 65.1]	1.6% [-13.5, 20.6]
Bipolar disorder						
<5	-	-	-	-	-	-
5-9	-	-	-	-	-	-
10-15	7199 [3990, 11909]	-13.8% [-26.1, 1]	3.5% [-11.5, 21]	8.4 [4.6, 13.8]	2.3% [-12.3, 19.9]	0.1% [-14.3, 17.1]
15-19	30137 [17596, 47641]	-40% [-45.5, -33.3]	4.1% [-4, 15.2]	40.4 [23.6, 63.8]	1.7% [-7.6, 13.1]	0.9% [-7, 11.7]
20-24	39170 [24149, 61654]	-45% [-49.6, -40.5]	-6.8% [-14.8, 0.7]	53.5 [33.0, 84.3]	-0.8% [-9.1, 7.4]	-0.4% [-9, 7.6]
25-29	49404 [31079, 74652]	-21.6% [-28, -14.7]	-14.7% [-21.2, -8]	57.1 [35.9, 86.3]	-0.4% [-8.5, 8.4]	-1% [-8.5, 6.8]
30-34	68127 [42754, 103165]	37.2% [25.5, 49.2]	-3.2% [-10.6, 4]	56.2 [35.3, 85.2]	-0.1% [-8.6, 8.7]	-0.4% [-8, 7]
35-39	58217 [37042, 87421]	15.9% [7, 26.3]	11% [3.6, 20.9]	54.9 [35.0, 82.5]	-0.1% [-7.8, 8.9]	0% [-6.7, 8.9]
40-44	49728 [30109, 74039]	36.3% [24, 47.5]	-4.7% [-11.7, 3.2]	54.3 [32.9, 80.9]	-0.1% [-9.1, 8.1]	0.7% [-6.7, 9.1]
45-49	59940 [36358, 92296]	113% [97.4, 132.1]	-6.9% [-13.4, 0.9]	54.3 [33.0, 83.7]	-0.3% [-7.6, 8.6]	-0.3% [-7.3, 8.1]
50-54	67463 [41755, 102637]	152.4% [134.5, 173.8]	-1.8% [-8.9, 6.1]	55.8 [34.5, 84.9]	-0.4% [-7.4, 8.1]	-0.6% [-7.8, 7.4]
55-59	62029 [37214, 93888]	152.6% [132.9, 174.1]	17.8% [9.5, 26.8]	56.4 [33.8, 85.4]	-0.4% [-8.1, 8.1]	-0.2% [-7.2, 7.4]
60-64	38426 [23338, 57845]	106.7% [91.6, 123.5]	-5.9% [-12.8, 1.5]	52.6 [32.0, 79.2]	0.1% [-7.3, 8.2]	-0.6% [-7.9, 7.2]
65-69	36710 [23190, 55918]	182.9% [161.2, 207.5]	10% [2.2, 18.4]	47.9 [30.2, 72.9]	0.6% [-7.1, 9.4]	0% [-7.2, 7.5]
70-74	23140 [14799, 34797]	184.8% [161.3, 210.5]	11.9% [2.3, 23.2]	43.4 [27.8, 65.3]	0.6% [-7.8, 9.6]	0.2% [-8.3, 10.4]
75-79	13037 [8256, 19313]	187.9% [164.1, 216]	9.1% [-0.2, 19.9]	39.4 [24.9, 58.3]	-1.1% [-9.2, 8.6]	0% [-8.6, 9.9]
80-84	7027 [4477, 10198]	268.4% [235.7, 309.8]	4.8% [-4.9, 14.5]	35.5 [22.6, 51.5]	-1.4% [-10.2, 9.7]	-0.3% [-9.5, 9]
85-89	3017 [1828, 4411]	457.6% [401, 520.5]	11.6% [1.6, 22.5]	31.7 [19.2, 46.3]	-1.3% [-11.3, 9.9]	-0.2% [-9.1, 9.5]
90-94	815 [480, 1207]	836.8% [733.8, 948.5]	14.2% [2.4, 28.7]	27.8 [16.4, 41.2]	-2% [-12.7, 9.7]	-0.1% [-10.5, 12.5]
95	153 [88, 239]	1462.9% [1267, 1700.9]	17.7% [2.5, 34.2]	23.9 [13.8, 37.5]	-1% [-13.4, 14.1]	-0.5% [-13.4, 13.4]
Eating disorders						
<5	-	-	-	-	-	-
5-9	1621 [645, 3373]	22.6% [13.3, 34.6]	11.7% [4.1, 20.7]	1.7 [0.7, 3.5]	33.5% [23.4, 46.5]	0.7% [-6.1, 8.9]

10-15	16654 [9433, 28180]	34% [15.4, 54.6]	5.9% [-2.9, 15.7]	19.3 [10.9, 32.7]	59% [36.9, 83.5]	2.5% [-6, 11.9]
15-19	50956 [27106, 88464]	-0.6% [-9.5, 8.6]	7.6% [2, 13.7]	68.2 [36.3, 118.5]	68.7% [53.4, 84.3]	4.3% [-1.1, 10.2]
20-24	62553 [30327, 107593]	-3.5% [-10, 3.6]	-0.4% [-6, 6]	85.5 [41.4, 147.0]	74% [62.4, 86.8]	6.4% [0.4, 13.3]
25-29	65802 [35680, 111281]	35.2% [23.9, 46.6]	-8.6% [-13.6, -3.2]	76.1 [41.3, 128.7]	71.8% [57.5, 86.3]	6% [0.2, 12.3]
30-34	75719 [41527, 122873]	127.6% [107, 153.6]	1.5% [-4.5, 8.1]	62.5 [34.3, 101.4]	65.8% [50.8, 84.7]	4.4% [-1.8, 11.2]
35-39	47590 [25363, 78678]	96.1% [76.9, 118.1]	15.3% [6.6, 24.1]	44.9 [23.9, 74.3]	69% [52.5, 88]	3.9% [-4, 11.8]
40-44	21097 [11460, 33721]	150.6% [115.6, 186.6]	-1% [-9.3, 8.2]	23.0 [12.5, 36.8]	83.7% [58, 110]	4.7% [-4.1, 14.3]
45-49	10777 [5587, 18171]	339.5% [264.2, 442.3]	-2.4% [-14.2, 10.8]	9.8 [5.1, 16.5]	105.6% [70.4, 153.7]	4.4% [-8.2, 18.6]
50-54	-	-	-	-	-	-
55-59	-	-	-	-	-	-
60-64	-	-	-	-	-	-
65-69	-	-	-	-	-	-
70-74	-	-	-	-	-	-
75-79	-	-	-	-	-	-
80-84	-	-	-	-	-	-
85-89	-	-	-	-	-	-
90-94	-	-	-	-	-	-
95	-	-	-	-	-	-
Anorexia nervosa						
<5	-	-	-	-	-	-
5-9	1519 [557, 3311]	21% [11.5, 32.7]	11.4% [3.2, 21]	1.6 [0.6, 3.5]	31.7% [21.4, 44.5]	0.5% [-6.9, 9.2]
10-15	9045 [5098, 14495]	23.5% [4.5, 47.1]	4.1% [-9.6, 18.2]	10.5 [5.9, 16.8]	46.6% [24, 74.6]	0.7% [-12.5, 14.3]
15-19	18230 [9838, 31442]	-11.1% [-19.3, 0.8]	3.9% [-4.8, 12.2]	24.4 [13.2, 42.1]	50.7% [36.8, 70.9]	0.7% [-7.8, 8.7]
20-24	16375 [8904, 28148]	-16% [-24.8, -5.4]	-5.9% [-14.8, 4.4]	22.4 [12.2, 38.5]	51.5% [35.6, 70.6]	0.5% [-9, 11.5]
25-29	13297 [7542, 22571]	17.7% [3.1, 36]	-13.4% [-24.2, -1.7]	15.4 [8.7, 26.1]	49.6% [31, 72.8]	0.4% [-12.1, 14.1]
30-34	11045 [6186, 18631]	100.5% [69.3, 143.7]	-1.9% [-14.5, 13.8]	9.1 [5.1, 15.4]	46% [23.3, 77.5]	0.9% [-12, 17]
35-39	6159 [3476, 10178]	68.6% [35.1, 110.6]	12.8% [-5.6, 32.2]	5.8 [3.3, 9.6]	45.3% [16.5, 81.5]	1.6% [-15, 19.1]
40-44	3271 [1906, 5204]	96.5% [57.5, 146.5]	-3.9% [-21.6, 18.4]	3.6 [2.1, 5.7]	44.1% [15.5, 80.7]	1.5% [-17.2, 25.1]
45-49	1989 [1133, 3132]	223.6% [186, 275.9]	-4.5% [-14.4, 5.2]	1.8 [1.0, 2.8]	51.4% [33.8, 75.9]	2.2% [-8.4, 12.6]

50-54	-	-	-	-	-	-
55-59	-	-	-	-	-	-
60-64	-	-	-	-	-	-
65-69	-	-	-	-	-	-
70-74	-	-	-	-	-	-
75-79	-	-	-	-	-	-
80-84	-	-	-	-	-	-
85-89	-	-	-	-	-	-
90-94	-	-	-	-	-	-
95	-	-	-	-	-	-
Bulimia nervosa						
<5	-	-	-	-	-	-
5-9	103 [32, 255]	53.9% [45.9, 66.5]	15% [11.9, 19.1]	0.1 [0.0, 0.3]	67.5% [58.8, 81.3]	3.8% [0.9, 7.4]
10-15	7609 [3249, 15821]	49% [22.8, 81.5]	8.1% [-6.9, 24.4]	8.8 [3.8, 18.4]	76.8% [45.7, 115.4]	4.6% [-9.9, 20.4]
15-19	32726 [14117, 71005]	6.5% [-3.7, 16]	9.8% [3.1, 19.1]	43.8 [18.9, 95.1]	80.6% [63.4, 96.8]	6.5% [-0.1, 15.4]
20-24	46179 [18567, 88523]	1.8% [-6.4, 12.6]	1.8% [-5.6, 9.3]	63.1 [25.4, 121.0]	83.6% [68.9, 103.1]	8.7% [0.8, 16.7]
25-29	52505 [25466, 93156]	40.5% [27.7, 55.3]	-7.3% [-13.7, -0.1]	60.7 [29.4, 107.7]	78.5% [62.3, 97.4]	7.5% [0.1, 15.9]
30-34	64673 [32612, 111197]	132.9% [109.4, 165]	2.2% [-4.6, 9.5]	53.4 [26.9, 91.8]	69.7% [52.5, 93]	5.1% [-1.9, 12.6]
35-39	41431 [20326, 69623]	101% [80, 127.6]	15.7% [5.7, 25.4]	39.1 [19.2, 65.7]	73.2% [55.1, 96.2]	4.2% [-4.8, 12.9]
40-44	17826 [8690, 29566]	164% [121.7, 207.7]	-0.4% [-10.8, 10.8]	19.5 [9.5, 32.3]	93.5% [62.5, 125.6]	5.3% [-5.7, 17.1]
45-49	8789 [3872, 15866]	378.2% [274.5, 537.2]	-2% [-15.7, 15.6]	8.0 [3.5, 14.4]	123.7% [75.2, 198.1]	5% [-9.8, 23.8]
50-54	-	-	-	-	-	-
55-59	-	-	-	-	-	-
60-64	-	-	-	-	-	-
65-69	-	-	-	-	-	-
70-74	-	-	-	-	-	-
75-79	-	-	-	-	-	-
80-84	-	-	-	-	-	-
85-89	-	-	-	-	-	-

90-94	-	-	-	-	-	-
95	-	-	-	-	-	-
Autism spectrum disorders						
<5	108236 [73496, 153357]	-27.3% [-31, -23.8]	-11.6% [-14.4, -8.8]	139.4 [94.6, 197.5]	4.7% [-0.7, 9.7]	0.2% [-3, 3.4]
5-9	130984 [87820, 185033]	-3.2% [-8.9, 2.3]	11.2% [5.9, 16.3]	136.8 [91.7, 193.2]	5.4% [-0.8, 11.3]	0.3% [-4.5, 4.9]
10-15	116151 [78531, 164527]	-9.7% [-14.7, -4.5]	3.6% [-0.7, 8.1]	134.8 [91.1, 190.9]	7.2% [1.2, 13.3]	0.3% [-3.9, 4.6]
15-19	99589 [67659, 140946]	-36.3% [-39.7, -32.8]	3.6% [-0.3, 7.9]	133.4 [90.6, 188.8]	8.1% [2.2, 14]	0.5% [-3.4, 4.6]
20-24	96372 [65863, 136056]	-40.2% [-43.5, -36.8]	-5.7% [-10.1, -1.6]	131.7 [90.0, 185.9]	7.9% [1.9, 14]	0.7% [-4, 5.1]
25-29	112203 [76530, 159856]	-15.9% [-20.9, -10.8]	-12.9% [-16.9, -8.9]	129.7 [88.5, 184.8]	6.9% [0.5, 13.4]	1% [-3.5, 5.7]
30-34	154468 [105490, 218285]	45% [37.5, 53]	-2.1% [-6.1, 2.1]	127.5 [87.1, 180.2]	5.6% [0.2, 11.5]	0.7% [-3.4, 5]
35-39	132658 [89063, 188335]	23.4% [17.2, 31.2]	11.1% [6.6, 16.1]	125.2 [84.1, 177.7]	6.3% [1, 13.1]	0.1% [-4, 4.5]
40-44	112248 [76136, 158856]	44.6% [36.4, 53.1]	-4.4% [-8.2, -0.3]	122.6 [83.2, 173.5]	6% [-0.1, 12.2]	1.1% [-3, 5.3]
45-49	130800 [89601, 186028]	124.8% [113, 138]	-6.4% [-10.4, -2.2]	118.6 [81.2, 168.6]	5.2% [-0.3, 11.4]	0.2% [-4.1, 4.7]
50-54	139927 [93995, 195617]	167% [152, 182.4]	-0.5% [-4.4, 3.8]	115.8 [77.8, 161.9]	5.4% [-0.5, 11.5]	0.8% [-3.3, 5.1]
55-59	122826 [84078, 174428]	168.9% [153.7, 182.3]	18.1% [13.7, 22.7]	111.7 [76.5, 158.7]	6.1% [0.1, 11.3]	0% [-3.7, 3.9]
60-64	78733 [52827, 110516]	125.7% [113.2, 140]	-4.8% [-8.8, -0.4]	107.8 [72.4, 151.4]	9.3% [3.2, 16.2]	0.5% [-3.6, 5.2]
65-69	75960 [52199, 107501]	219.2% [201.4, 238.2]	10.8% [5.8, 15.6]	99.0 [68.1, 140.2]	13.5% [7.2, 20.3]	0.7% [-3.9, 5]
70-74	47140 [31904, 65717]	246.3% [223.1, 268.5]	12.4% [8.2, 16.9]	88.4 [59.9, 123.3]	22.3% [14.1, 30.1]	0.7% [-3.1, 4.7]
75-79	24973 [16878, 35150]	287.1% [261.6, 313.7]	9.9% [5.3, 14.8]	75.4 [51.0, 106.1]	33% [24.3, 42.2]	0.7% [-3.5, 5.2]
80-84	12080 [8257, 16961]	448.4% [404.5, 499.8]	6.2% [0.5, 11.7]	61.0 [41.7, 85.7]	46.8% [35, 60.5]	1.1% [-4.4, 6.3]
85-89	4343 [2937, 6225]	815% [722, 929.2]	13.6% [7.4, 20.1]	45.6 [30.8, 65.3]	62% [45.6, 82.3]	1.6% [-4, 7.4]
90-94	915 [587, 1316]	1550.3% [1353.7, 1783.8]	15.6% [8.6, 23.8]	31.2 [20.0, 44.9]	72.7% [52.1, 97.1]	1.1% [-5.1, 8.2]
95	131 [80, 199]	2957.2% [2538, 3627.8]	19.3% [10.1, 30.6]	20.5 [12.5, 31.2]	93.7% [67.1, 136.2]	0.8% [-6.9, 10.4]
Attention-deficit/hyperactivity disorder						
<5	4595 [2368, 7992]	-3.2% [-13.3, 8.6]	-6.8% [-16.3, 3.7]	5.9 [3.0, 10.3]	39.4% [24.8, 56.3]	5.7% [-5.1, 17.5]
5-9	51782 [27220, 89386]	12.1% [2.6, 23.2]	10.7% [1.8, 21]	54.1 [28.4, 93.3]	22.1% [11.7, 34.1]	-0.1% [-8.1, 9.2]
10-15	60807 [31639, 103840]	1% [-7.9, 11.4]	4.5% [-4, 13]	70.5 [36.7, 120.5]	19.8% [9.4, 32.2]	1.1% [-7.1, 9.3]
15-19	40368 [21632, 65837]	-33.9% [-40, -27.7]	6.5% [-1.5, 16.2]	54.1 [29.0, 88.2]	12.1% [1.7, 22.6]	3.2% [-4.6, 12.6]
20-24	27882 [15699, 44534]	-41.5% [-46.7, -35.9]	-2.6% [-9.2, 5.3]	38.1 [21.5, 60.9]	5.6% [-3.8, 15.6]	4% [-3, 12.5]

25-29	24367 [12708, 40517]	-19.4% [-26.5, -12.1]	-10.1% [-16.4, -3.4]	28.2 [14.7, 46.9]	2.4% [-6.7, 11.6]	4.3% [-3, 12.1]
30-34	26182 [13442, 43530]	34.1% [21.5, 46.1]	0.4% [-6.7, 9.2]	21.6 [11.1, 35.9]	-2.4% [-11.5, 6.5]	3.3% [-4, 12.3]
35-39	18225 [9587, 29634]	10.5% [0.1, 20.7]	13.2% [5.1, 21.7]	17.2 [9.0, 28.0]	-4.7% [-13.7, 4.1]	2% [-5.4, 9.7]
40-44	12722 [6693, 21115]	27.2% [13.6, 40.7]	-3.4% [-11.6, 4.8]	13.9 [7.3, 23.1]	-6.7% [-16.8, 3.1]	2.1% [-6.6, 10.7]
45-49	12147 [6381, 19740]	94.6% [75.8, 115.3]	-5.6% [-13, 3]	11.0 [5.8, 17.9]	-9% [-17.7, 0.7]	1% [-6.8, 10.2]
50-54	10151 [5213, 16478]	128.1% [104.7, 153.2]	0.7% [-8.9, 11.1]	8.4 [4.3, 13.6]	-10% [-19.2, 0]	1.9% [-7.7, 12.5]
55-59	6036 [3098, 10136]	123.1% [98.5, 145.6]	18.9% [7.5, 33.3]	5.5 [2.8, 9.2]	-12% [-21.7, -3.1]	0.7% [-9, 12.8]
60-64	2263 [1108, 3893]	79.7% [56, 106.7]	-4.6% [-17.2, 9]	3.1 [1.5, 5.3]	-13% [-24.5, 0.1]	0.8% [-12.5, 15.2]
65-69	1158 [506, 2148]	146.1% [100.5, 196.4]	10.9% [-8.4, 34.9]	1.5 [0.7, 2.8]	-12.5% [-28.7, 5.4]	0.8% [-16.8, 22.6]
70-74	348 [108, 761]	148.6% [83.1, 218.9]	11.4% [-17.3, 48.4]	0.7 [0.2, 1.4]	-12.2% [-35.3, 12.6]	-0.2% [-25.9, 33]
75-79	73 [14, 183]	158.7% [97.1, 214.2]	9.8% [-11.3, 37.6]	0.2 [0.0, 0.6]	-11.1% [-32.3, 8]	0.6% [-18.7, 26.1]
80-84	4 [0, 17]	214.8% [124.4, 288.9]	5.3% [-18.6, 34.1]	0.0 [0.0, 0.1]	-15.7% [-39.9, 4.1]	0.3% [-22.5, 27.6]
85-89	-	351.4% [170.7, 511.1]	13.6% [-28.1, 59.8]	0.0 [0.0, 0.0]	-20.1% [-52.1, 8.2]	1.6% [-35.7, 42.8]
90-94	-	653.5% [210.6, 1265.2]	17.4% [-43.8, 137.5]	0.0 [0.0, 0.0]	-21.1% [-67.5, 42.9]	2.7% [-50.9, 107.6]
95	-	1237.2% [376, 3004.2]	26.2% [-53.3, 252.7]	0.0 [0.0, 0.0]	-15.3% [-69.8, 96.7]	6.6% [-60.5, 198.2]
Conduct disorder						
<5	-	-	-	-	-	-
5-9	112052 [57251, 186880]	-4.5% [-12, 4.4]	10.9% [5.8, 16.1]	117.0 [59.8, 195.1]	4% [-4.1, 13.6]	0% [-4.6, 4.7]
10-15	297623 [156607, 489718]	-11.4% [-17.8, -4.6]	3.2% [0.7, 5.8]	345.3 [181.7, 568.2]	5.1% [-2.4, 13.2]	-0.1% [-2.5, 2.4]
15-19	137524 [70202, 221553]	-37.4% [-43.6, -31.3]	3.2% [0.2, 6.8]	184.2 [94.0, 296.7]	6.1% [-4.2, 16.6]	0% [-2.9, 3.5]
20-24	7852 [3550, 14427]	-41.9% [-56.3, -21.2]	-6.2% [-20.7, 11.4]	10.7 [4.9, 19.7]	4.8% [-21.2, 42.2]	0.2% [-15.3, 18.9]
25-29	-	-	-	-	-	-
30-34	-	-	-	-	-	-
35-39	-	-	-	-	-	-
40-44	-	-	-	-	-	-
45-49	-	-	-	-	-	-
50-54	-	-	-	-	-	-
55-59	-	-	-	-	-	-
60-64	-	-	-	-	-	-

65-69	-	-	-	-	-	-
70-74	-	-	-	-	-	-
75-79	-	-	-	-	-	-
80-84	-	-	-	-	-	-
85-89	-	-	-	-	-	-
90-94	-	-	-	-	-	-
95	-	-	-	-	-	-
Idiopathic developmental intellectual disability						
<5	9753 [1496, 21429]	-58.3% [-72.6, -53.6]	-13.4% [-18.8, -8.1]	12.6 [1.9, 27.6]	-40% [-60.5, -33.3]	-1.9% [-8, 4.2]
5-9	16495 [4502, 32752]	-38.6% [-53, -33.3]	8.8% [1, 16.2]	17.2 [4.7, 34.2]	-33.2% [-48.8, -27.3]	-1.8% [-8.9, 4.8]
10-15	14556 [3840, 28943]	-41.3% [-54.7, -35.2]	1.1% [-6, 7.1]	16.9 [4.5, 33.6]	-30.3% [-46.2, -23.1]	-2.2% [-9, 3.6]
15-19	11927 [3129, 23870]	-57.9% [-67.8, -51.6]	1.1% [-6.3, 7.5]	16.0 [4.2, 32.0]	-28.6% [-45.4, -17.9]	-2% [-9.1, 4.2]
20-24	10875 [2792, 21986]	-60.2% [-68.7, -53]	-7.8% [-14.9, -0.3]	14.9 [3.8, 30.0]	-28.2% [-43.5, -15.2]	-1.6% [-9.1, 6.5]
25-29	12651 [3469, 25310]	-41.6% [-53.9, -29.9]	-14.8% [-21.1, -8.5]	14.6 [4.0, 29.3]	-25.8% [-41.4, -11]	-1.2% [-8.5, 6.2]
30-34	16995 [5054, 33033]	3.4% [-15.8, 22.6]	-4.3% [-11.5, 2.5]	14.0 [4.2, 27.3]	-24.7% [-38.7, -10.7]	-1.6% [-8.9, 5.4]
35-39	13784 [3938, 27315]	-13.3% [-30.1, 4.5]	8.6% [-0.6, 16.3]	13.0 [3.7, 25.8]	-25.3% [-39.7, -9.9]	-2.2% [-10.5, 4.8]
40-44	10924 [3022, 21617]	2.9% [-18.6, 26.1]	-8% [-16.7, -0.9]	11.9 [3.3, 23.6]	-24.6% [-40.3, -7.5]	-2.8% [-12, 4.7]
45-49	12030 [3473, 23648]	61.7% [31.3, 108.1]	-8.1% [-15.8, 1.2]	10.9 [3.1, 21.4]	-24.3% [-38.6, -2.6]	-1.6% [-9.8, 8.3]
50-54	11673 [3272, 23290]	92% [52.8, 156.9]	-2.8% [-11, 7.5]	9.7 [2.7, 19.3]	-24.2% [-39.7, 1.4]	-1.6% [-9.9, 8.8]
55-59	9191 [2520, 18913]	91.6% [48.2, 143.7]	16.8% [6, 27.6]	8.4 [2.3, 17.2]	-24.4% [-41.5, -3.9]	-1.1% [-10.2, 8]
60-64	5094 [1284, 10650]	51% [11.5, 104.2]	-7.4% [-16.6, 2.3]	7.0 [1.8, 14.6]	-26.9% [-46.1, -1.1]	-2.2% [-11.9, 8.1]
65-69	4503 [1158, 9628]	100.6% [36.2, 173.4]	7.8% [-0.9, 16.6]	5.9 [1.5, 12.6]	-28.7% [-51.6, -2.8]	-2.1% [-10, 5.9]
70-74	2632 [687, 5734]	90.4% [18.8, 163.9]	9% [0, 18.4]	4.9 [1.3, 10.8]	-32.8% [-58.1, -6.8]	-2.3% [-10.4, 6.1]
75-79	1438 [398, 3040]	87% [19.9, 147.2]	5.7% [-3.3, 14.3]	4.3 [1.2, 9.2]	-35.7% [-58.8, -15.1]	-3.2% [-11.4, 4.8]
80-84	802 [271, 1611]	149.5% [83.4, 211.9]	2.9% [-5.3, 11.6]	4.1 [1.4, 8.1]	-33.2% [-50.9, -16.5]	-2.1% [-9.9, 6.3]
85-89	360 [125, 702]	302.4% [201.9, 397.2]	9.5% [0.2, 18.7]	3.8 [1.3, 7.4]	-28.7% [-46.5, -12]	-2.1% [-10.4, 6.1]
90-94	103 [40, 193]	636.8% [470.3, 793.3]	12% [0.4, 23.6]	3.5 [1.4, 6.6]	-22.9% [-40.3, -6.5]	-2.1% [-12.2, 8.1]
95	20 [8, 37]	1249.6% [855.4, 1600]	16% [5, 28.2]	3.2 [1.3, 5.8]	-14.5% [-39.5, 7.7]	-2% [-11.3, 8.4]
Other mental						

<5	-	-	-	-	-	-
5-9	-	-	-	-	-	-
10-15	3539 [1931, 5825]	-15.1% [-19, -10.2]	3.1% [-1.2, 7]	4.1 [2.2, 6.8]	0.8% [-3.9, 6.5]	-0.2% [-4.4, 3.5]
15-19	20367 [11022, 33872]	-40.4% [-46.2, -33.8]	3.1% [-8.1, 15.3]	27.3 [14.8, 45.4]	1.1% [-8.7, 12.3]	-0.1% [-10.9, 11.8]
20-24	50139 [27587, 79181]	-44% [-47.3, -40.2]	-6.2% [-11.6, 0.5]	68.5 [37.7, 108.2]	1% [-5, 7.9]	0.2% [-5.6, 7.3]
25-29	95645 [59374, 146205]	-20.8% [-24.8, -15.8]	-13.6% [-18, -9.1]	110.6 [68.7, 169.1]	0.6% [-4.4, 7]	0.2% [-4.9, 5.5]
30-34	179028 [112070, 277705]	37.5% [32.1, 43.1]	-2.5% [-6.5, 1.4]	147.8 [92.5, 229.2]	0.1% [-3.8, 4.2]	0.2% [-3.8, 4.3]
35-39	183573 [115879, 284620]	16.4% [11.4, 21.3]	10.9% [6.7, 15.9]	173.2 [109.4, 268.6]	0.3% [-4, 4.5]	-0.1% [-3.9, 4.3]
40-44	171149 [108169, 263899]	36.1% [30.7, 41.6]	-5.4% [-9, -1.7]	187.0 [118.2, 288.3]	-0.2% [-4.2, 3.8]	0% [-3.9, 3.9]
45-49	213656 [135919, 325228]	112.5% [104.8, 120.1]	-6.8% [-10.2, -3.4]	193.7 [123.2, 294.8]	-0.6% [-4.2, 3]	-0.2% [-3.9, 3.5]
50-54	236645 [150636, 358820]	151.3% [142.2, 160.8]	-1.2% [-4.3, 2]	195.8 [124.6, 296.9]	-0.8% [-4.4, 3]	0% [-3.1, 3.2]
55-59	215297 [136763, 327157]	151% [141.5, 159.9]	17.8% [13.9, 21.7]	195.8 [124.4, 297.6]	-1% [-4.7, 2.5]	-0.3% [-3.6, 3]
60-64	143068 [90484, 218485]	105.5% [96.8, 113.9]	-5.4% [-8.4, -2.1]	196.0 [123.9, 299.3]	-0.6% [-4.7, 3.5]	-0.1% [-3.3, 3.5]
65-69	149430 [96665, 224088]	180.9% [170.5, 191.7]	10.1% [6.1, 14]	194.8 [126.0, 292.1]	-0.1% [-3.8, 3.8]	0% [-3.6, 3.5]
70-74	103424 [66741, 154601]	184.5% [174.8, 194.2]	11.4% [7.3, 15.3]	194.1 [125.2, 290.1]	0.4% [-3, 3.9]	-0.2% [-3.9, 3.3]
75-79	64431 [42120, 95372]	193.6% [184.4, 204.7]	8.8% [5.6, 12.1]	194.5 [127.2, 288.0]	0.9% [-2.3, 4.7]	-0.3% [-3.3, 2.7]
80-84	38970 [25387, 56898]	277.4% [265.7, 291]	5% [1.5, 8.8]	196.9 [128.3, 287.5]	1% [-2.1, 4.6]	0% [-3.4, 3.6]
85-89	19302 [12810, 28233]	464.3% [446.1, 487]	11.6% [8, 15.2]	202.6 [134.5, 296.4]	-0.1% [-3.3, 3.9]	-0.2% [-3.4, 3]
90-94	6313 [4230, 9167]	842.4% [806.1, 876.3]	13.9% [10.1, 17.9]	215.3 [144.3, 312.6]	-1.4% [-5.2, 2.2]	-0.4% [-3.7, 3.1]
95	1515 [982, 2234]	1444.6% [1380, 1506.2]	17.7% [13.3, 22.2]	237.1 [153.6, 349.6]	-2.1% [-6.2, 1.8]	-0.5% [-4.2, 3.3]
