# Supplementary material for: The disability-adjusted life years attributable to mental disorders and self-harm in China from 1990–2021: Findings from the global burden of disease study 2021
Source: PLOS Ment Health. 2025 Apr 9;2(4):e0000146. doi: 10.1371/journal.pmen.0000146 (PMC12798377; doi:10.1371/journal.pmen.0000146)
Supplement: S2 Table — (PDF) [file pmen.0000146.s005.pdf]

Table S2 Changing DALYs of all mental disorders and self-harm in 34 provincial units in China, 1990-2021.

		**Self-harm**	**Mental disorders**	**Depressive disorders**	**Major depressive disorder**	**Dysthymia**	**Anxiety disorders**	**Schizophrenia**	**Bipolar disorder**
**North China**									
Beijing	2021	87.4 [64.1, 161.7]	1333.0 [990.6, 1686.7]	356.7 [235.1, 497.6]	215.9 [130.5, 319.9]	140.8 [92.3, 199.6]	339.0 [207.4, 520.0]	189.8 [140.3, 246.1]	36.1 [23.2, 53.1]
	1990 vs. 2021	-70.6% [-78.8, -55.4]	5.7% [-1.7, 14.9]	6.5% [-7.2, 23.6]	11.2% [-12.6, 42.7]	0.1% [-6, 6.9]	6.8% [-18.1, 38.7]	3.3% [-5.8, 11.8]	-0.2% [-11.1, 12.6]
	2019 vs. 2021	2.2% [-22.3, 36.1]	3.7% [-3.9, 12.4]	4.9% [-8.9, 22.5]	2.8% [-18.2, 28.7]	8.3% [1.1, 15.8]	8.2% [-16.3, 40.4]	2% [-5.6, 9.7]	-0.6% [-11, 10.5]
Tianjin	2021	155.5 [116.2, 234.2]	1268.5 [943.8, 1615.4]	322.8 [215.3, 445.3]	182.2 [112.2, 268.5]	140.6 [92.7, 197.4]	385.2 [252.3, 566.5]	189.9 [138.5, 244.0]	36.3 [23.5, 53.1]
	1990 vs. 2021	-59.4% [-70.3, -41.7]	1.9% [-5.9, 10.8]	-2.1% [-15.9, 12.9]	-3.5% [-26.4, 22.4]	-0.4% [-6.8, 6]	1.6% [-20.1, 27.3]	3.8% [-4.6, 12.5]	-0.2% [-10.6, 11.8]
	2019 vs. 2021	-1.8% [-26.9, 34.8]	1.7% [-6.4, 10.7]	4.4% [-8.6, 19.9]	1.4% [-20.1, 27.2]	8.4% [1.8, 15]	0.3% [-21.7, 26.5]	2.1% [-5.4, 10.6]	0.2% [-8.9, 11.1]
Hebei	2021	264.9 [205.2, 344.4]	1483.3 [1101.3, 1885.4]	444.3 [293.9, 629.8]	292.7 [180.1, 433.9]	151.6 [98.2, 214.9]	368.3 [222.5, 544.4]	233.5 [172.9, 299.5]	89.6 [58.3, 130.0]
	1990 vs. 2021	-47.2% [-63.1, -26.0]	1.2% [-6.1, 9.5]	-1.8% [-15.5, 13.9]	-3% [-23.8, 21.8]	0.5% [-5.5, 6.8]	4.1% [-21.2, 34]	0.1% [-7, 7.9]	-0.1% [-7.3, 8.5]
	2019 vs. 2021	3.6% [-20.9, 36.5]	2.5% [-5.5, 11.9]	-0.5% [-13.8, 16.6]	-1.7% [-21.3, 23.1]	2% [-3.8, 8.8]	11% [-15.3, 44.1]	0.2% [-6.9, 8.1]	-0.2% [-6.6, 6.9]
Shanxi	2021	258.7 [191.5, 342.2]	1384.7 [1012.7, 1783.0]	393.0 [268.8, 552.3]	252.2 [160.0, 374.8]	140.8 [91.3, 197.9]	420.9 [269.3, 610.6]	185.5 [137.4, 238.8]	36.0 [23.3, 52.5]
	1990 vs. 2021	-64.4% [-75.0, -48.6]	0.5% [-7.6, 9.5]	-7% [-20.3, 7.3]	-10.8% [-29.4, 10.8]	0.7% [-5.5, 7.1]	3.1% [-21.8, 29.3]	3.4% [-4.4, 12.5]	0.1% [-10.3, 12.1]
	2019 vs. 2021	-1.3% [-27.5, 38.1]	3.3% [-5.1, 12.6]	-1.6% [-15.2, 13.3]	-6.4% [-25.8, 15.6]	8.3% [1.3, 15.7]	12.5% [-16.1, 43.1]	1.7% [-5.8, 9.5]	-0.4% [-9.7, 9.5]
Inner Mongolia	2021	252.2 [196.2, 320.2]	1409.0 [1037.8, 1805.8]	409.0 [282.8, 556.6]	267.9 [176.4, 379.6]	141.1 [92.3, 197.3]	427.2 [269.2, 637.4]	186.5 [138.1, 238.8]	36.1 [23.1, 52.4]
	1990 vs. 2021	-68.5% [-77.3, -52.2]	0.5% [-7.8, 10.5]	-9.5% [-22.5, 5.3]	-14.1% [-32.1, 8.3]	1% [-5.6, 7.9]	4.9% [-19.8, 33.7]	4.2% [-3.9, 12.3]	0.1% [-10.8, 12.7]
	2019 vs. 2021	-0.5% [-24.5, 33.6]	4.1% [-4.6, 15.2]	-0.7% [-14.3, 15.7]	-4.9% [-23.8, 18.8]	8.4% [1.5, 16.3]	14.2% [-12.5, 47.8]	1.8% [-6, 9.5]	-0.2% [-10.6, 12.5]
**East China**									
Shanghai	2021	104 [78.7, 166.2]	1250.1 [945.3, 1595.9]	327.3 [222.6, 448.4]	187.1 [115.4, 270.4]	140.2 [92.0, 198.9]	308.3 [191.5, 483.3]	224.7 [167.0, 281.6]	36.3 [23.3, 53.0]
	1990 vs. 2021	-65.0% [-75.6, -50.3]	1.4% [-5.6, 9.2]	-0.7% [-13.7, 13.8]	-1% [-21.9, 24.3]	-0.4% [-6.5, 6.2]	1.5% [-22.9, 30]	0.2% [-7.2, 8.3]	-0.2% [-10.8, 11.4]
	2019 vs. 2021	0.3% [-24.7, 36.9]	2.1% [-5.5, 10.3]	1.2% [-10.7, 16.1]	-3.4% [-22.8, 22.3]	8.1% [1.2, 15.3]	6.2% [-20.3, 36.2]	0.2% [-7, 8]	-0.3% [-10.8, 10.5]
Jiangsu	2021	175.9 [135.2, 231.5]	1355.2 [1008.6, 1748.7]	391.8 [269.7, 556.1]	250.9 [159.8, 377.7]	141.0 [92.1, 198.5]	418.3 [257.9, 601.6]	158.5 [118.8, 196.8]	36.3 [23.2, 52.7]
	1990 vs. 2021	-68.7% [-76.8, -53.3]	-1.9% [-10.7, 8.2]	-9.7% [-22.5, 6.5]	-14.5% [-33.2, 9.1]	0.2% [-6.2, 6.7]	1.9% [-23.1, 31.7]	-5% [-12.6, 3.2]	0.6% [-11.1, 11.9]

	2019 vs. 2021	-0.3% [-27.2, 32.9]	2.6% [-6.3, 13.3]	-2% [-16, 14.1]	-6.9% [-27.5, 17.3]	8.1% [0.7, 16.2]	12.1% [-14.8, 45.5]	-3.5% [-10.8, 4.3]	0.3% [-10, 11.2]
Zhejiang	2021	199.7 [154.7, 273.4]	1514.3 [1137.0, 1970.1]	518.9 [339.9, 733.4]	377.3 [236.8, 538.3]	141.7 [92.6, 201.2]	428.5 [276.7, 611.5]	194.5 [146.3, 243.1]	32.8 [21.1, 47.3]
	1990 vs. 2021	-68.4% [-76.9, -49.4]	-2.4% [-10.2, 8.3]	-10.3% [-24.7, 7.9]	-13.5% [-32.3, 10.9]	-0.3% [-6.6, 6.3]	1.1% [-21.1, 30]	-2.3% [-10.2, 5.3]	0.5% [-9.1, 13.4]
	2019 vs. 2021	0.0% [-24.1, 39.5]	-0.3% [-8.8, 10.3]	-2.2% [-17.4, 16.8]	-2.1% [-22.7, 24.7]	-2.5% [-7.9, 3.6]	1.1% [-21.6, 29.3]	0.1% [-7.2, 7.7]	0% [-9.8, 11.2]
Anhui	2021	353.8 [261.5, 449.1]	1384.3 [1011.6, 1819.9]	391.7 [256.4, 550.6]	250.5 [156.1, 374.3]	141.1 [92.1, 199.6]	420.2 [268.3, 626.7]	183.6 [134.1, 230.7]	36.1 [23.5, 52.1]
	1990 vs. 2021	-77.2% [-83.0, -61.2]	-4.4% [-12.1, 5.5]	-19.2% [-31.6, -3.4]	-27.4% [-43.3, -7.3]	0.9% [-5, 7.7]	2.8% [-19.8, 31.3]	-2.2% [-9.6, 5.2]	0.4% [-10.8, 11.4]
	2019 vs. 2021	-0.6% [-25.8, 34.1]	3.3% [-5.2, 14.2]	-1.3% [-16.5, 16.7]	-6% [-27.2, 20.1]	8.1% [1.5, 15.7]	12.5% [-11.7, 44.8]	0.1% [-7, 8]	-0.4% [-9.5, 10.9]
Fujian	2021	258.5 [202.3, 334.7]	1464.1 [1056.5, 1898.1]	427.8 [291.0, 604.0]	286.9 [181.8, 428.3]	141.0 [91.3, 198.2]	436.8 [266.5, 648.6]	212.1 [153.4, 272.8]	36.2 [22.1, 53.1]
	1990 vs. 2021	-73.2% [-80.0, -56.6]	-0.9% [-9.9, 8.6]	-10.4% [-23.2, 5.5]	-14.9% [-33.3, 6.9]	0.5% [-6.1, 6.7]	7.1% [-19.2, 37]	-5.3% [-13, 3.4]	0.3% [-10.9, 12]
	2019 vs. 2021	-0.4% [-25.4, 32.9]	5.6% [-4.3, 16.5]	2.2% [-12.4, 19.6]	-0.6% [-21.3, 24.1]	8.4% [1.7, 15.3]	17.4% [-12.1, 50.4]	0.3% [-7.6, 7.9]	-0.4% [-10.3, 11.2]
Jiangxi	2021	217.2 [169.5, 300.5]	1404.4 [1040.8, 1784.6]	410.1 [275.5, 563.5]	269.1 [172.3, 391.2]	141.0 [91.5, 199.2]	420.8 [275.3, 630.2]	185.5 [136.0, 240.6]	36.3 [22.7, 53.3]
	1990 vs. 2021	-81.1% [-86.1, -66.6]	-0.6% [-9.1, 8.5]	-11.6% [-23.4, 3.1]	-17% [-34.4, 5.8]	0.8% [-5.2, 7.5]	3.4% [-20.9, 30.8]	4.2% [-3.8, 13.4]	1.1% [-9.6, 12.2]
	2019 vs. 2021	0.2% [-26.3, 31.6]	3.6% [-5, 13.2]	-1.3% [-15.2, 15.8]	-5.6% [-25, 19.4]	8.1% [1, 16]	13.1% [-14.1, 43.8]	1.8% [-5.9, 9.8]	0.1% [-10.7, 13.1]
Shandong	2021	287.8 [214.6, 373.1]	1712.5 [1283.9, 2223.7]	494.7 [330.0, 715.3]	335.6 [216.6, 496.6]	159.1 [103.8, 226.3]	506.3 [316.9, 755.1]	305.6 [228.7, 376.0]	28.7 [19.0, 41.8]
	1990 vs. 2021	-74.2% [-81.5, -50.0]	0.9% [-8.3, 11.1]	-13.4% [-27, 2.2]	-18.6% [-36.6, 3]	0.2% [-6.4, 6.6]	0.4% [-24, 30.7]	26.9% [17.2, 35.8]	0.3% [-11.5, 15.1]
	2019 vs. 2021	-0.8% [-26.8, 35.0]	2.9% [-6, 13.3]	-1.8% [-16.6, 16.5]	-3.6% [-24.4, 23.2]	2% [-4.8, 9.1]	5.9% [-19.9, 37.2]	9.5% [2, 17.1]	0% [-11.4, 11.6]
Taiwan	2021	479.8 [446.5, 505.4]	1490.0 [1107.1, 1937.6]	389.9 [249.8, 542.9]	248.0 [148.7, 376.1]	141.9 [89.3, 208.8]	454.4 [287.1, 694.5]	202.1 [143.3, 277.9]	39.4 [23.4, 58.1]
	1990 vs. 2021	34.4% [24.2, 42.7]	3.7% [-6, 15.5]	10.4% [-5.6, 26.8]	16.7% [-9.8, 44.2]	0.8% [-2.1, 4.2]	-1.6% [-25.9, 27.9]	6.2% [-2.7, 15.8]	0.3% [-8.9, 11.4]
	2019 vs. 2021	-4.3% [-9.3, -0.9]	3.1% [-5.4, 13.9]	4% [-10.5, 21.4]	6.5% [-16.5, 33.8]	0% [-3, 3.1]	6.5% [-18.8, 37.2]	0.7% [-7, 9]	0.3% [-9.6, 11]
**South China**									
Guangdong	2021	164.4 [127.4, 229.5]	1401.7 [1053.8, 1764.2]	454.9 [306.4, 631.6]	315.0 [195.5, 452.0]	139.9 [91.7, 196.5]	270.7 [164.5, 417.7]	251.0 [191.3, 305.4]	36.1 [23.2, 52.9]
	1990 vs. 2021	-68.8% [-77.6, -54.7]	1.1% [-6, 8.8]	-3.3% [-18.7, 13.2]	-4.7% [-25.8, 18.2]	-0.2% [-6.3, 6]	3.9% [-19.2, 32.3]	1.3% [-5.3, 7.7]	0.3% [-10.6, 11.6]
	2019 vs. 2021	-0.5% [-24.2, 29.9]	3.7% [-3.4, 11.1]	3.3% [-12.3, 20.1]	1.2% [-20.4, 24.3]	8.2% [0.7, 15.1]	13.7% [-14.4, 44.9]	0.1% [-5.6, 6.5]	-0.1% [-10.4, 10.5]
Guangxi	2021	203.3 [149.5, 339.4]	1390.7 [1029.2, 1819.0]	404.6 [272.4, 579.4]	264.2 [170.5, 399.4]	140.4 [90.9, 195.1]	421.1 [268.7, 636.9]	185.9 [135.6, 241.9]	29.4 [18.5, 42.3]
	1990 vs. 2021	-61.3% [-73.0, -41.3]	0.8% [-8.2, 10.1]	-5.6% [-20.6, 11.2]	-8.5% [-29.8, 16.4]	0.4% [-5.7, 7.2]	3.7% [-22.5, 30.8]	3.5% [-4.3, 12.6]	0.4% [-11.3, 11.5]
	2019 vs. 2021	-0.7% [-25.3, 32.5]	3.5% [-5.6, 13.7]	-1.6% [-16.9, 15.4]	-6.1% [-26.7, 19.2]	8.1% [1.2, 15.2]	13.3% [-15.1, 45.3]	1.8% [-5.9, 10.3]	-0.3% [-11.6, 11.8]

Hainan	2021	250.1 [188.3, 331.3]	1464.8 [1084.8, 1878.2]	431.1 [291.9, 600.8]	291.5 [182.3, 424.5]	139.6 [91.1, 196.4]	440.1 [280.4, 640.8]	186.6 [138.3, 241.7]	36.0 [22.3, 52.6]
	1990 vs. 2021	-63.7% [-74.4, -40.8]	1.2% [-7.8, 11.3]	-9.1% [-22.7, 6.9]	-12.9% [-31.5, 9.6]	-0.2% [-6.6, 6.8]	8% [-18.2, 36.9]	3.6% [-4.6, 12.4]	0.2% [-10.3, 11.7]
	2019 vs. 2021	-0.4% [-28.6, 34.8]	6.1% [-3.6, 16.3]	2.1% [-13.6, 20.2]	-0.5% [-22.7, 25.2]	8.1% [0.3, 16.1]	19.1% [-10.6, 52.6]	2% [-6.1, 9.7]	0% [-9.6, 11.9]
Hong Kong	2021	395.1 [159.3, 519.5]	1437.1 [1052.2, 1883.7]	547.8 [350.1, 776.9]	402.8 [247.8, 602.7]	145.0 [94.6, 206.4]	304.4 [183.8, 463.9]	191.7 [138.3, 247.9]	36.7 [23.1, 55.2]
	1990 vs. 2021	-15.8% [-43.9, 7.6]	5.3% [-3.4, 13.8]	8.1% [-10, 25.9]	10% [-14.6, 34.5]	3% [-3.7, 10.2]	7.1% [-17.3, 36.6]	2.8% [-4.5, 12.1]	0.6% [-9.8, 11.9]
	2019 vs. 2021	-2.8% [-18.9, 15.3]	3.3% [-5.2, 11.8]	1.4% [-15.9, 18]	-1% [-22.6, 21.6]	8.6% [0.9, 15.8]	12.2% [-14.3, 44.4]	1.5% [-6.4, 10.3]	0.1% [-11.3, 12.7]
Macao	2021	231.5 [138.7, 312.1]	1462.6 [1079.5, 1886.6]	427.6 [294.4, 593.5]	283.4 [182.6, 417.6]	144.1 [93.5, 204.8]	442.5 [271.6, 648.5]	192.1 [141.7, 247.9]	36.5 [23.4, 53.5]
	1990 vs. 2021	-60.2% [-70.1, -43.6]	3.3% [-5.9, 14.8]	-1.8% [-16.8, 16.4]	-3.4% [-24.9, 23.6]	1.7% [-4.7, 8.8]	6.5% [-18.8, 39.7]	3.4% [-4.2, 12.3]	0.6% [-9.2, 11.9]
	2019 vs. 2021	-6.0% [-12.1, 1.3]	4.7% [-4.3, 15.7]	0.2% [-13.9, 17.5]	-3.6% [-23.9, 22.7]	8.7% [1.6, 16.8]	16.3% [-12.7, 52.8]	1.7% [-6.6, 10.4]	0.1% [-10.5, 11.5]
**Northwest China**									
Shaanxi	2021	185.2 [137.3, 291]	1418.1 [1070.6, 1838.4]	416.0 [281.4, 586.1]	274.6 [172.4, 412.1]	141.3 [92.1, 199.4]	429.1 [276.9, 636.8]	186.3 [136.0, 242.2]	36.2 [22.3, 53.1]
	1990 vs. 2021	-72.6% [-81.2, -54.0]	0% [-8.1, 9]	-10.6% [-24.9, 4.9]	-15.5% [-33.7, 6.4]	0.7% [-6.5, 7.1]	5% [-16.7, 31.1]	4.3% [-3.7, 13.2]	0.3% [-11, 12.5]
	2019 vs. 2021	-0.2% [-29.4, 37.6]	4.3% [-4.5, 13.9]	-0.6% [-14.9, 16.3]	-4.6% [-25.1, 20.1]	8.3% [1.2, 16.2]	14.7% [-9.6, 44.5]	2.1% [-5.3, 10.1]	-0.3% [-10.6, 12]
Gansu	2021	307.2 [242.6, 388.8]	1608.8 [1185.7, 2081.9]	467.7 [323.3, 661.5]	324.9 [209.8, 485.0]	142.8 [94.8, 202.9]	540.5 [342.2, 815.3]	184.6 [133.8, 238.4]	28.6 [18.0, 42.4]
	1990 vs. 2021	-68.0% [-76.7, -52.1]	-1.6% [-10.7, 9.2]	-14% [-26.8, 2.1]	-19.2% [-36.3, 1.6]	0.8% [-5.9, 7.9]	4.5% [-19.6, 35.5]	3.9% [-3.9, 12.4]	0.7% [-10.3, 14.2]
	2019 vs. 2021	0.2% [-23.3, 30.7]	1.1% [-7.8, 12.1]	-0.9% [-15.3, 16.1]	-0.3% [-21.8, 25.7]	-2.3% [-8.2, 4.2]	3.1% [-20.7, 35.4]	2% [-5.3, 10]	0% [-11.2, 13.7]
Qinghai	2021	363.3 [273.3, 488.7]	1243.4 [943.6, 1575.7]	406.5 [283.9, 563.2]	264.0 [170.3, 387.9]	142.6 [92.9, 202.8]	280.3 [175.2, 420.2]	184.3 [133.9, 238.9]	28.6 [18.4, 42.2]
	1990 vs. 2021	-61.0% [-72.0, -41.9]	-1.2% [-8.3, 7.1]	-9.9% [-22.6, 4.2]	-14.6% [-32.6, 6.9]	0.2% [-6.4, 7]	1.2% [-22.9, 29.8]	4.4% [-3.3, 13.3]	1.1% [-10.6, 14.5]
	2019 vs. 2021	-2.5% [-28.5, 36.3]	0% [-7.3, 7.5]	-4.8% [-18.5, 11.4]	-6.1% [-25.6, 17.6]	-2.3% [-8.2, 4.3]	5.6% [-19.4, 38]	2.1% [-5.6, 9.6]	0% [-9.5, 11.5]
Ningxia	2021	200.4 [147.1, 299.1]	1562.3 [1157.3, 1984.7]	408.8 [278.9, 566.0]	267.7 [170.0, 389.3]	141.2 [91.5, 198.0]	583.5 [374.9, 832.6]	185.5 [135.4, 241.7]	36.2 [22.8, 53.8]
	1990 vs. 2021	-68.7% [-79.1, -50.7]	1.2% [-8.8, 12.5]	-6.9% [-20.5, 10.3]	-10.5% [-29.8, 15.5]	0.7% [-5.6, 7.5]	3.8% [-19.6, 33.1]	3.9% [-3.4, 12.6]	0.3% [-10, 12.1]
	2019 vs. 2021	-1.1% [-30.2, 38.3]	1% [-9.2, 12]	-1.1% [-14.2, 17.4]	-5.5% [-24.3, 21.8]	8.3% [1.1, 16.1]	2.4% [-21.4, 33.4]	1.7% [-6.2, 9.7]	0.1% [-11, 11.3]
Xinjiang	2021	236.9 [171.4, 391.5]	1455.0 [1070.0, 1906.6]	473.5 [320.8, 685.7]	333.4 [214.7, 497.4]	140.1 [90.8, 196.1]	427.2 [271.2, 618.4]	173.9 [128.8, 223.7]	35.9 [22.9, 51.7]
	1990 vs. 2021	-57.0% [-68.6, -33.9]	-0.6% [-9.6, 10.4]	-12.9% [-26.8, 1.8]	-17.5% [-36.1, 3.4]	0.6% [-5.7, 7.4]	5.4% [-17.5, 36.5]	15.5% [1.8, 30.1]	0% [-10.6, 13.1]
	2019 vs. 2021	1.1% [-23.6, 37.8]	6.6% [-3, 17.6]	1.7% [-13.2, 19.4]	-0.8% [-21.7, 25]	8.1% [0.9, 15.5]	14.6% [-10.8, 48.2]	17.3% [5.4, 31.3]	-0.5% [-12.2, 11.4]
**Northeast China**									

Liaoning	2021	243.5 [187.6, 324.5]	1248.0 [927.8, 1604.4]	379.4 [256.7, 523.8]	238.0 [150.1, 348.6]	141.4 [92.0, 199.7]	308.4 [186.8, 457.5]	187.1 [135.3, 237.5]	36.2 [22.8, 53.3]
	1990 vs. 2021	-39.8% [-55.9, -19.2]	2.8% [-4.6, 11.1]	-0.9% [-15.9, 14.9]	-1.7% [-23.2, 22.6]	0.4% [-5.9, 7.1]	5.4% [-19.1, 32.3]	3.1% [-4.5, 12.3]	-0.1% [-11.6, 11.9]
	2019 vs. 2021	-1.4% [-24.5, 30.5]	2.1% [-5.2, 10.6]	-1.7% [-15.3, 12.8]	-6.7% [-26.7, 15.5]	8.2% [0.8, 15.7]	9.9% [-14.2, 38.9]	1.8% [-5.6, 10.3]	-0.3% [-10.7, 11.3]
Jilin	2021	225.6 [171.4, 316.9]	1431.0 [1056.2, 1864.5]	413.8 [269.6, 579.1]	272.3 [174.2, 409.3]	141.5 [91.2, 199.3]	438.6 [275.1, 659.7]	185.2 [134.8, 237.5]	36.1 [23.0, 52.3]
	1990 vs. 2021	-68.8% [-77.5, -54.0]	2.4% [-6.6, 13]	-4.7% [-18.8, 11.7]	-7.4% [-27.3, 16.3]	0.8% [-5.8, 6.9]	7.3% [-18.3, 36.6]	3.8% [-4.8, 12.2]	0.4% [-10.8, 12]
	2019 vs. 2021	0.1% [-27.5, 39.5]	5.6% [-3.7, 17]	1.8% [-12.8, 18.7]	-1.2% [-22.8, 23]	8.2% [1.5, 15]	17% [-10.8, 52.6]	1.6% [-6, 10.1]	-0.3% [-9.8, 10.3]
Heilongjiang	2021	245.2 [187.6, 330]	1418.8 [1048.9, 1843.3]	420.3 [285.0, 583.8]	279.0 [178.5, 414.0]	141.3 [92.3, 199.0]	450.0 [278.2, 670.3]	182.2 [133.9, 232.8]	36.2 [23.4, 52.8]
	1990 vs. 2021	-65.5% [-75.1, -51.4]	3% [-5.6, 13.4]	-3.6% [-18.6, 13.6]	-5.7% [-26.9, 19.1]	0.8% [-6, 7.8]	10.4% [-16.5, 39.1]	-0.2% [-7.3, 8.4]	0.2% [-10.2, 12.5]
	2019 vs. 2021	0.0% [-24.3, 30.6]	7% [-2.1, 17.2]	3.7% [-11.9, 21.1]	1.4% [-21.1, 27]	8.5% [1.9, 15]	20.2% [-8.2, 53.4]	0.4% [-7.1, 7.5]	0.1% [-10.6, 12.5]
**Central China**									
Henan	2021	282.7 [219.2, 359.5]	1456.9 [1101.7, 1884.8]	435.7 [289.9, 604.1]	293.7 [182.2, 442.4]	142.0 [92.6, 199.7]	422.1 [278.8, 627.7]	186.7 [140.0, 243.4]	36.2 [22.9, 52.9]
	1990 vs. 2021	-65.8% [-74.8, -44.5]	-1.5% [-9.8, 7.8]	-12.7% [-26.4, 3.4]	-18.1% [-36.7, 3.8]	0.8% [-5.6, 7.5]	3.1% [-22.8, 34.4]	3.7% [-4.3, 13.3]	0.2% [-10.7, 10.9]
	2019 vs. 2021	-0.6% [-24.3, 32.5]	3.4% [-5.7, 13.1]	-1.3% [-16.3, 15.3]	-5.3% [-27, 17.7]	8.3% [0.9, 15.2]	12.7% [-15.9, 47.6]	2% [-5.7, 10.2]	-0.4% [-11, 10.8]
Hubei	2021	490.5 [249.6, 632.9]	1462.3 [1069.4, 1910.6]	465.2 [307.6, 644.7]	324.5 [206.8, 475.7]	140.7 [91.9, 196.4]	432.8 [274.4, 655.9]	182.4 [136.9, 226.0]	36.1 [23.4, 53.1]
	1990 vs. 2021	-70.2% [-77.2, -56.3]	-2.7% [-11.4, 7]	-14.6% [-28.9, 0.5]	-19.8% [-39, 0.7]	0.4% [-5.7, 7.1]	6.1% [-19.7, 40.3]	-2.8% [-9.5, 5.4]	0.6% [-9.9, 12.2]
	2019 vs. 2021	-1.1% [-24.5, 31.7]	4.7% [-4.4, 15.6]	0.5% [-15.7, 17.8]	-2.6% [-24.7, 21.5]	8.2% [1, 15.7]	16.4% [-10.1, 56.3]	0.4% [-6.9, 8.4]	0.1% [-9.8, 12]
Hunan	2021	238.8 [185.8, 319]	1735.8 [1284.3, 2285.1]	471.4 [323.2, 667.1]	322.7 [208.5, 492.2]	148.8 [97.7, 212.1]	694.6 [448.3, 1012.3]	185.1 [134.6, 240.9]	29.3 [19.0, 42.4]
	1990 vs. 2021	-78.0% [-84.1, -60.8]	-2.6% [-12.6, 8.4]	-15.8% [-28.7, -0.4]	-20.4% [-37.3, 0.9]	-3.7% [-10.1, 3.5]	2.9% [-18.7, 30.8]	4.2% [-3.5, 12.8]	0.8% [-11.3, 14.1]
	2019 vs. 2021	0.7% [-24.3, 33.3]	7.8% [-3.3, 19.5]	4.4% [-11.3, 22.1]	1.9% [-20, 27.4]	10.1% [2.6, 16.7]	17% [-9.7, 48]	2.2% [-5.6, 10.9]	-0.1% [-12.2, 11.4]
**Southwest China**									
Chongqing	2021	262.7 [196.2, 350.8]	1423.5 [1041.7, 1824.0]	411.3 [276.6, 569.8]	270.4 [171.2, 399.9]	140.9 [91.8, 199.9]	428.4 [272.7, 611.8]	186.0 [134.6, 241.7]	35.9 [23.0, 52.0]
	1990 vs. 2021	-72.7% [-81.7, -56.7]	0.5% [-7.7, 10.5]	-8.9% [-21.8, 8]	-13.4% [-31.7, 11.8]	1.1% [-5.1, 7.7]	5.2% [-20.1, 34.8]	4.3% [-4, 13.4]	0.1% [-9.9, 13.2]
	2019 vs. 2021	-1.8% [-28.2, 39.4]	4.3% [-4.5, 15.3]	-0.5% [-14.3, 16.6]	-4.5% [-24, 20.8]	8.2% [0.6, 15.8]	14.7% [-12.2, 46.6]	2% [-5.2, 11.1]	-0.7% [-10.4, 10.2]
Sichuan	2021	299.1 [229.6, 392.6]	1324.3 [992.7, 1726.0]	396.4 [264.4, 563.7]	255.6 [159.4, 383.9]	140.8 [91.7, 197.3]	371.8 [243.8, 563.3]	196.1 [143.7, 253.4]	39.3 [26.0, 57.7]
	1990 vs. 2021	-65.7% [-75.0, -43.8]	0.1% [-7.6, 9.8]	-5% [-19.5, 10.9]	-7.9% [-30.3, 17.5]	0.7% [-5.7, 7.9]	4.1% [-20.5, 34.8]	0.4% [-10.2, 10]	0.9% [-8.8, 11.4]
	2019 vs. 2021	-2.3% [-27.7, 34.2]	2.3% [-5.8, 12.1]	-0.7% [-14.5, 16.6]	-5.1% [-25.7, 19.4]	8.3% [1, 15.2]	9.1% [-19.1, 41.9]	0.3% [-6.9, 8.3]	-0.3% [-11.3, 9.2]

Guizhou	2021	346.3 [259.5, 458.2]	1395.9 [1025.6, 1798.1]	433.9 [294.3, 614.0]	293.2 [179.4, 428.9]	140.6 [91.6, 199.6]	430.4 [275.8, 637.2]	183.5 [133.3, 236.5]	36.0 [23.8, 52.3]
	1990 vs. 2021	-62.1% [-73.3, -43.6]	0.8% [-7.2, 10.7]	-6.9% [-20.2, 10.1]	-10.1% [-29.4, 14.1]	0.7% [-6.1, 7.8]	6.2% [-18.1, 35.8]	4.7% [-3.1, 13.6]	0.6% [-9.5, 11.1]
	2019 vs. 2021	0.7% [-25.7, 43.3]	4.8% [-3.7, 15.6]	0.6% [-14, 18.8]	-2.7% [-23.7, 24]	8.2% [1.6, 15]	15.1% [-11.6, 46.4]	2.3% [-5.4, 12.2]	-0.3% [-9.8, 10.6]
Yunnan	2021	496.6 [378.5, 620.5]	1442.8 [1074.6, 1835.5]	412.4 [272.1, 589.0]	272.4 [175.0, 411.9]	140.0 [91.5, 197.0]	434.6 [276.5, 628.9]	213.9 [159.1, 270.0]	35.9 [23.1, 53.4]
	1990 vs. 2021	-65.3% [-74.9, -39.6]	-0.4% [-8.6, 10]	-8.1% [-21.6, 8.2]	-11.8% [-31.5, 11.3]	0.1% [-6.7, 6.3]	7% [-18.5, 35.8]	-7.4% [-15.2, -0.4]	0.1% [-12.4, 13.6]
	2019 vs. 2021	-1.1% [-27.3, 35.3]	4.1% [-3.9, 14.1]	2.1% [-13.1, 18.7]	-0.8% [-22.3, 23.6]	8.1% [1.2, 15.3]	16.7% [-11.8, 48.3]	-6.7% [-13.2, 1]	0% [-10.2, 10.9]
Tibet	2021	245.5 [174.1, 493.7]	1322.6 [986.4, 1691.4]	384.5 [267.4, 530.4]	244.5 [159.4, 354.6]	139.9 [91.5, 195.9]	402.1 [251.9, 582.3]	158.6 [115.7, 208.5]	35.8 [23.2, 53.3]
	1990 vs. 2021	-70.4% [-79.8, -53.9]	-3.2% [-11.8, 6.7]	-12% [-23.1, 0.9]	-17.3% [-33.8, 2.5]	-0.9% [-7.6, 5.8]	-2.2% [-25.9, 26.9]	0.5% [-7.9, 8.9]	0.7% [-10, 12]
	2019 vs. 2021	-0.3% [-26.2, 41.0]	0.8% [-7.9, 11.1]	-5.4% [-17.8, 10.1]	-11.7% [-30, 9.7]	8.1% [1.2, 15.6]	7.8% [-17.9, 42]	0.4% [-8.2, 10.3]	0% [-10.9, 11.1]

		**Eating disorders**	**Anorexia nervosa**	**Bulimia nervosa**	**Autism spectrum disorders**	**Attention-deficit and hyperactivity disorder**	**Conduct disorder**	**Idiopathic developmental intellectual disability**	**Other mental disorders**
**North China**									
Beijing	2021	37.1 [22.6, 59.1]	9.4 [5.2, 16.5]	27.6 [15.1, 46.6]	176.6 [121.2, 250.0]	29.8 [15.6, 49.6]	58.3 [32.0, 91.0]	5.3 [0.5, 13.6]	104.1 [67.3, 158.2]
	1990 vs. 2021	67.9% [48.6, 91.4]	67.5% [31.9, 118]	68% [43.8, 97.2]	5.7% [-4.3, 17.4]	-4.5% [-17.4, 6.7]	3% [-6.9, 15.4]	-34.8% [-54.9, -23.8]	0.3% [-4.4, 5.8]
	2019 vs. 2021	4.8% [-6.8, 17.4]	0.3% [-14.8, 17.2]	6.4% [-8, 21.8]	-0.1% [-8.2, 8.4]	0.8% [-10.7, 14.9]	-0.4% [-7.8, 7.6]	-1.7% [-18.9, 18.3]	0% [-5.3, 5.3]
Tianjin	2021	37.2 [22.5, 57.7]	9.0 [5.2, 14.7]	28.2 [15.4, 46.9]	104.4 [70.4, 148.4]	21.2 [10.6, 36.7]	59.0 [31.8, 92.4]	8.4 [1.5, 17.8]	104.1 [66.4, 155.1]
	1990 vs. 2021	74.1% [54.1, 99.4]	50.1% [17.2, 87.2]	83.5% [59.1, 113.6]	3.6% [-6.9, 14.4]	0.9% [-14.6, 18.9]	5.1% [-4.6, 16.9]	-36% [-53.1, -28.1]	-0.1% [-4.9, 5.3]
	2019 vs. 2021	4.9% [-5.2, 17.3]	1.2% [-17.6, 23.7]	6.2% [-6.3, 20.8]	0.6% [-8.8, 10.6]	0.8% [-11.3, 17.1]	0.2% [-7.8, 8.8]	-1.5% [-13.3, 11.5]	-0.2% [-5.6, 5.4]
Hebei	2021	27.6 [15.3, 46.1]	6.2 [3.1, 11.0]	21.4 [10.5, 39.1]	118.2 [81.3, 165.0]	26.3 [14.6, 43.2]	58.7 [31.9, 93.2]	13.3 [3.2, 27.4]	103.6 [67.5, 157.1]
	1990 vs. 2021	19.9% [2.4, 39.3]	40.8% [9.7, 86.1]	15% [-4.8, 36.6]	3.6% [-7, 15]	21.9% [6.5, 39.7]	4.7% [-5.7, 15.8]	-25.7% [-41.1, -16.8]	-0.8% [-5.8, 3.7]
	2019 vs. 2021	3.7% [-9.9, 19.3]	-0.1% [-19.5, 25.3]	4.9% [-11.1, 22.5]	0.5% [-9, 9.5]	1.7% [-10.7, 14.6]	0% [-8, 8.5]	-0.9% [-10.9, 11]	0% [-4.9, 5.3]
Shanxi	2021	27.0 [16.2, 43.0]	6.9 [4.0, 11.2]	20.0 [11.0, 33.4]	119.2 [81.4, 167.3]	26.1 [14.6, 42.5]	58.1 [31.3, 92.0]	15.1 [4.1, 29.9]	104.0 [67.0, 158.2]
	1990 vs. 2021	67.8% [44.9, 94.7]	48.4% [13.2, 92.7]	75.7% [46.6, 112.2]	3.9% [-6.5, 15.1]	20.2% [5, 37.1]	3.3% [-6.3, 14.2]	-16.6% [-31.9, 7.5]	-0.4% [-5.8, 4.8]
	2019 vs. 2021	4.4% [-7.6, 16.2]	0.8% [-18.7, 26.2]	5.8% [-8.8, 20.1]	0% [-11, 9.6]	1.7% [-11.4, 14.5]	-0.3% [-7.6, 7.3]	-1.2% [-10.8, 9.5]	-0.1% [-4.5, 4.6]
Inner Mongolia	2021	31.6 [19.2, 50.3]	7.8 [4.7, 12.6]	23.8 [13.5, 40.4]	119.0 [81.0, 167.9]	26.2 [14.4, 42.1]	58.3 [31.3, 92.3]	10.9 [2.5, 22.3]	104.3 [67.0, 159.3]
	1990 vs. 2021	93.1% [68, 129.9]	64.4% [28.8, 117.7]	105% [72, 144.1]	4.9% [-6.8, 16.3]	21.4% [5.3, 39.9]	4.5% [-5.9, 16]	-34.2% [-53.5, -9.7]	-0.4% [-5.5, 4.7]

	2019 vs. 2021	3.6% [-7.6, 15.8]	0.9% [-18.9, 25.8]	4.6% [-8.9, 19]	0.7% [-8.1, 9.5]	1.6% [-11.6, 16.1]	0.2% [-8.6, 10]	-1.1% [-12.2, 11.2]	-0.1% [-5.1, 4.8]
**East China**									
Shanghai	2021	34.8 [21.0, 55.0]	7.5 [4.3, 12.9]	27.3 [15.1, 45.3]	114.1 [76.3, 158.7]	33.3 [18.4, 54.1]	58.7 [31.5, 93.9]	8.0 [1.3, 17.7]	104.5 [66.0, 160.4]
	1990 vs. 2021	48.5% [29.9, 68.6]	38.1% [7.9, 78.4]	51.6% [31, 78.9]	3.3% [-7.6, 13.5]	-6.1% [-16.9, 9.3]	4.2% [-6.8, 14.4]	-13.8% [-30.6, -2.4]	0.6% [-4, 6]
	2019 vs. 2021	4.1% [-7.3, 17.5]	-1.6% [-22.4, 22.9]	5.7% [-7.7, 21.7]	0.5% [-8.8, 9.8]	3.8% [-9.1, 18.9]	0% [-7.3, 8]	-1.4% [-14.7, 12.7]	-0.1% [-5.4, 5.1]
Jiangsu	2021	34.9 [20.8, 54.9]	8.6 [5.0, 13.8]	26.3 [14.6, 43.9]	108.4 [74.0, 153.6]	34.1 [18.9, 55.9]	59.0 [31.5, 93.6]	10.1 [2.4, 21.5]	103.8 [67.5, 157.5]
	1990 vs. 2021	78.7% [55.2, 105.1]	55.1% [22, 95.5]	88.1% [58.9, 124.4]	4.7% [-5.9, 16.6]	-3.5% [-14.6, 8.8]	5.2% [-5.3, 17.2]	-35.8% [-51.1, -27.5]	0.1% [-5, 6.1]
	2019 vs. 2021	5.5% [-6.2, 18.5]	1.1% [-17.3, 23.8]	7% [-8.7, 22.8]	0.5% [-6.9, 9.1]	1% [-10.5, 15.2]	0% [-8.8, 9.1]	-2.1% [-16.8, 11.8]	0% [-4.9, 5.2]
Zhejiang	2021	33.4 [20.1, 51.6]	8.5 [5.1, 13.4]	24.9 [13.6, 41.8]	105.3 [72.1, 149.0]	26.6 [14.6, 43.5]	59.0 [32.0, 92.1]	10.7 [2.5, 21.8]	104.5 [66.8, 157.8]
	1990 vs. 2021	68.4% [48.2, 92.5]	49.9% [19.1, 90.5]	75.9% [50.3, 108.8]	5.3% [-5.2, 17.9]	22.9% [7.5, 41.5]	5.1% [-5.5, 16.7]	-28.8% [-44.1, -21.2]	0.4% [-4.6, 5.7]
	2019 vs. 2021	4.6% [-5.8, 16.9]	0.3% [-18.1, 24.4]	6.1% [-6.3, 20.6]	0.5% [-8.9, 10.4]	1.9% [-9.9, 15.1]	0.6% [-6.9, 9.2]	-1.2% [-12.6, 13.4]	-0.2% [-5.4, 4.4]
Anhui	2021	27.3 [16.4, 43.9]	7.0 [4.1, 11.5]	20.3 [11.3, 33.7]	120.9 [83.7, 172.7]	26.5 [14.4, 43.9]	59.3 [31.9, 92.0]	14.8 [4.2, 29.9]	103.9 [67.0, 159.0]
	1990 vs. 2021	79.3% [54.8, 107.4]	56.4% [18.5, 110]	88.8% [59.1, 122.1]	6.4% [-2.9, 18.6]	22.2% [6.7, 41.5]	5.4% [-4, 16.4]	-26.9% [-48.5, -3.9]	0% [-5.1, 5.9]
	2019 vs. 2021	5.2% [-8.8, 19.3]	1.4% [-19.7, 25.7]	6.6% [-9.5, 26.3]	0.8% [-8, 11.7]	1.6% [-10.5, 15.4]	-0.1% [-7.9, 9.6]	-2% [-15.4, 9.7]	0.1% [-5.4, 5.3]
Fujian	2021	26.4 [13.6, 45.6]	8.4 [5.1, 13.4]	18.0 [6.5, 36.3]	122.2 [85.0, 170.7]	29.0 [15.6, 49.0]	58.9 [32.4, 92.0]	10.4 [2.1, 21.9]	104.2 [66.6, 158.7]
	1990 vs. 2021	71.2% [41.7, 101.2]	58% [24.7, 98.8]	78.2% [42.1, 124.9]	7.6% [-2.5, 19.6]	3.8% [-10.3, 25.1]	5.3% [-5.9, 17.1]	-34.6% [-51.3, -26.6]	0.3% [-5.5, 5.7]
	2019 vs. 2021	12.1% [-4.7, 29.9]	1% [-18.1, 23.4]	18.1% [-3.8, 47.4]	0.8% [-7.8, 10.4]	1.6% [-11.2, 17.7]	-0.3% [-8.1, 9]	-2.7% [-14.5, 9.3]	-0.1% [-5.3, 5.2]
Jiangxi	2021	27.2 [16.4, 42.5]	6.9 [4.1, 11.2]	20.2 [11.3, 33.8]	118.8 [81.7, 168.0]	26.6 [14.5, 43.7]	59.5 [32.3, 93.9]	15.5 [4.6, 30.5]	104.1 [65.9, 158.8]
	1990 vs. 2021	77.5% [54.6, 102.6]	54.3% [20.6, 100.3]	87.1% [59.9, 123.5]	7.5% [-2.8, 18.1]	22.7% [6.9, 41.6]	6.2% [-4.7, 18]	-24.8% [-43.2, -1]	0.3% [-5.4, 6.9]
	2019 vs. 2021	5% [-6.4, 19.4]	1.8% [-20.3, 26.8]	6.1% [-7.9, 24.5]	0.6% [-9.1, 10.2]	1.7% [-10, 15]	-0.1% [-6.5, 7.5]	-2.1% [-14.1, 10.1]	0% [-5.1, 5.8]
Shandong	2021	31.9 [18.7, 51.2]	8.0 [4.6, 12.9]	23.8 [13.4, 40.9]	146.5 [101.6, 205.1]	26.5 [14.6, 43.5]	59.2 [32.1, 91.5]	9.5 [1.8, 20.8]	103.7 [65.9, 157.8]
	1990 vs. 2021	75.9% [54.6, 103.9]	54% [24.9, 93.1]	84.7% [55.4, 122.9]	6.6% [-3.2, 18.2]	23.2% [7.5, 40]	5.7% [-4.9, 17.7]	-37.5% [-55.2, -29.1]	-0.1% [-5.3, 5.1]
	2019 vs. 2021	5.2% [-7.5, 18.4]	1.3% [-17.6, 23.2]	6.6% [-8.4, 22.4]	0.4% [-8.5, 10.4]	2% [-11, 15.5]	0.5% [-7.4, 8.6]	-1.9% [-16.5, 11.7]	-0.2% [-5.1, 5.7]
Taiwan	2021	42.2 [25.1, 66.4]	10.4 [6.0, 16.5]	31.8 [17.6, 52.7]	172.2 [119.4, 241.7]	12.1 [6.5, 20.1]	62.9 [33.4, 97.0]	4.7 [0.3, 12.6]	110.2 [71.1, 166.8]
	1990 vs. 2021	36.1% [21.5, 53.4]	24.4% [0.6, 54.4]	40.4% [22.9, 62.7]	4.9% [-3.1, 13.3]	0.2% [-7.4, 8.3]	1.7% [-7.4, 12.6]	-59.2% [-88.5, -46.2]	-1% [-6.1, 4.5]
	2019 vs. 2021	0.5% [-9.7, 11.4]	-0.3% [-18.2, 19.7]	0.8% [-11.4, 14]	0.5% [-8, 8.1]	0.4% [-11.6, 15.5]	-0.1% [-5.8, 5.1]	-3.1% [-21.7, 25.8]	-0.1% [-5, 5.6]

**South China**									
Guangdong	2021	31.0 [19.0, 48.7]	6.8 [3.7, 11.3]	24.2 [13.4, 39.9]	154.3 [108.0, 211.1]	30.3 [17.4, 47.5]	59.3 [32.3, 92.2]	9.6 [1.9, 20.8]	104.5 [66.5, 157.9]
	1990 vs. 2021	61.2% [40.8, 83.3]	39.6% [7.5, 79.7]	68.5% [45, 95.9]	7.2% [-3.5, 18.5]	-15.1% [-29, 1.9]	5.5% [-5.5, 16.2]	-27.3% [-45.2, -18.2]	0.9% [-4.6, 6.3]
	2019 vs. 2021	5.3% [-5.6, 18.5]	1.1% [-19.1, 28.1]	6.5% [-8.6, 22.2]	0.2% [-9.7, 8]	5.9% [-7.4, 23.2]	-0.2% [-8.6, 8.1]	-1.6% [-16.6, 14.3]	0% [-5.7, 5.7]
Guangxi	2021	26.9 [16.2, 40.9]	6.9 [3.9, 10.9]	20.0 [11.3, 32.8]	118.5 [79.3, 167.6]	26.0 [14.8, 40.7]	58.6 [31.8, 92.2]	15.6 [4.4, 31.5]	104.0 [66.3, 158.5]
	1990 vs. 2021	71.9% [47, 100.8]	52.1% [17.9, 93.9]	80% [50, 117.4]	4.7% [-4.6, 15.2]	-6.9% [-20.3, 10.7]	3.8% [-6.8, 16.9]	-18.5% [-32.9, 3]	-0.1% [-5.3, 5.3]
	2019 vs. 2021	4.8% [-7.9, 17.9]	-0.2% [-20, 27.2]	6.6% [-7.3, 24.2]	0.3% [-8.6, 10.3]	1.1% [-10.2, 15]	0% [-8.8, 8.9]	-1.5% [-13.2, 10.7]	-0.1% [-5.1, 5]
Hainan	2021	27.9 [16.9, 44.7]	7.1 [4.3, 11.4]	20.8 [11.8, 35.3]	139.1 [94.6, 196.2]	26.8 [14.8, 44.1]	59.5 [32.4, 92.4]	13.4 [3.7, 27.5]	104.4 [67.7, 159.0]
	1990 vs. 2021	55.4% [35.6, 80.3]	37.8% [7.7, 74.1]	62.4% [37.7, 94.9]	5.9% [-4.3, 16.8]	22.9% [7.5, 41.2]	5.4% [-4.7, 17.4]	-24.1% [-38.8, -14.5]	0.4% [-4.8, 5.6]
	2019 vs. 2021	4.8% [-7.2, 19.1]	0.9% [-18.1, 29]	6.2% [-7.3, 22.2]	0.2% [-9.3, 11.2]	1.8% [-11.6, 14.5]	-0.3% [-7, 6]	-2.5% [-14.3, 10.6]	0% [-5.1, 6]
Hong Kong	2021	44.9 [27.2, 71.6]	11.4 [6.5, 18.3]	33.4 [19.0, 56.1]	122.7 [84.7, 174.4]	23.1 [12.2, 38.6]	58.0 [31.0, 93.3]	5.8 [0.9, 13.6]	101.9 [65.9, 156.2]
	1990 vs. 2021	22.7% [8.7, 38.5]	10.7% [-7.8, 35.5]	27.5% [10.8, 48.7]	-1.5% [-9.8, 9.3]	-5.1% [-18.4, 11.2]	2.5% [-8.1, 13.1]	32.7% [12.4, 111.6]	-2.6% [-7.7, 2.6]
	2019 vs. 2021	2.5% [-7.8, 13.3]	-1.1% [-19.8, 20.2]	3.7% [-8.1, 17.7]	0.4% [-8.3, 10.5]	1% [-10.7, 17.3]	0.3% [-7.8, 9.4]	3.4% [-12.4, 20.8]	-0.2% [-5.7, 5.4]
Macao	2021	51.2 [30.3, 81.1]	12.2 [7.0, 20.0]	39.0 [21.4, 65.4]	122.9 [83.1, 170.3]	25.7 [13.7, 42.1]	57.8 [31.1, 90.6]	4.1 [0.5, 10.1]	102.3 [66.2, 156.6]
	1990 vs. 2021	29.1% [16.1, 44]	14.6% [-7.7, 43.2]	34.5% [18.9, 54.7]	2.8% [-6.3, 12.8]	21.5% [3.9, 41.3]	4% [-6.6, 15]	22.3% [3.8, 101.5]	-1.2% [-6.4, 4.3]
	2019 vs. 2021	-2.6% [-12.9, 7.5]	-4.9% [-21.7, 16]	-1.9% [-13.8, 11.1]	0.4% [-8.1, 9.9]	1.6% [-10.9, 15.6]	0.1% [-7.7, 8.4]	17.2% [2, 48.9]	-0.5% [-5.6, 4.5]
**Northwest China**									
Shaanxi	2021	29.1 [17.6, 46.5]	7.4 [4.3, 12.0]	21.7 [11.9, 36.6]	118.5 [81.6, 166.1]	26.3 [14.6, 44.0]	58.6 [32.4, 90.9]	13.7 [3.4, 27.5]	104.4 [67.3, 156.8]
	1990 vs. 2021	87.6% [62.4, 113.3]	61.9% [25, 109.2]	98.4% [67.6, 138.7]	5.6% [-4.4, 16.6]	21.4% [3.6, 40.7]	4.3% [-6.5, 16.5]	-29.1% [-46.8, -2.5]	0% [-5, 5.1]
	2019 vs. 2021	4.1% [-7.9, 17.1]	0.8% [-21, 24.7]	5.3% [-10.1, 22]	0.4% [-6.9, 9.7]	1.5% [-11.8, 15]	-0.2% [-9.1, 8.9]	-2.2% [-12.3, 9.5]	-0.1% [-4.7, 5.4]
Gansu	2021	24.9 [14.7, 39.5]	6.5 [3.7, 10.2]	18.4 [10.3, 30.8]	158.7 [109.1, 222.1]	26.1 [14.5, 43.1]	58.4 [31.7, 90.8]	15.5 [4.1, 31.2]	103.9 [67.0, 154.9]
	1990 vs. 2021	71.5% [49.1, 97.1]	50.4% [16.9, 97.6]	80.4% [49.6, 121.3]	4% [-6, 15.4]	20.6% [5.8, 37.2]	4.6% [-6.4, 16.4]	-20.2% [-38.6, 9.2]	-0.3% [-5.3, 5]
	2019 vs. 2021	5.1% [-6.2, 19.2]	0.5% [-20.6, 22.4]	6.8% [-7.9, 25.1]	0.5% [-8.9, 10.2]	1.7% [-10.9, 15.1]	-0.1% [-7.5, 7.8]	-1.5% [-14, 12.8]	0% [-4.9, 5.6]
Qinghai	2021	27.6 [16.8, 42.5]	7.1 [4.2, 11.7]	20.5 [11.1, 34.9]	112.6 [76.9, 159.7]	26.1 [14.4, 43.1]	58.0 [31.7, 91.4]	15.3 [4.3, 30.2]	104.2 [67.1, 161.1]
	1990 vs. 2021	73.1% [49.4, 98.7]	53.9% [17.6, 102]	80.9% [50.6, 113]	3.1% [-6.5, 14]	20.6% [4.8, 37.9]	3.4% [-6.8, 15.6]	-20.1% [-43.9, 10.7]	0.1% [-5.2, 5.2]
	2019 vs. 2021	4.6% [-6.6, 17.3]	1.4% [-15.2, 20.8]	5.7% [-7.4, 22]	0.4% [-7.4, 9.6]	1.4% [-10.3, 14.6]	-0.1% [-7.9, 8.3]	-2.2% [-14.9, 10.4]	-0.2% [-4.7, 5.1]

Ningxia	2021	28.8 [17.9, 45.2]	7.6 [4.6, 12.0]	21.3 [11.8, 34.9]	117.8 [83.2, 165.0]	26.1 [14.3, 42.5]	58.1 [31.9, 91.1]	13.4 [3.5, 27.4]	104.0 [66.4, 155.2]
	1990 vs. 2021	82.5% [59.4, 111.7]	62.7% [28.2, 113.2]	90.8% [62.1, 129.8]	5.5% [-5, 17]	21.2% [6.7, 38.2]	4.2% [-5.6, 15.8]	-26.6% [-44.5, -3.2]	-0.4% [-5.4, 5.2]
	2019 vs. 2021	5.1% [-9.1, 19.2]	1.3% [-18.3, 29]	6.5% [-10.1, 24]	0.8% [-7.4, 11.3]	1.7% [-11.2, 14.6]	0% [-7.9, 8.3]	-1.9% [-12.4, 8.8]	-0.2% [-4.8, 5.4]
Xinjiang	2021	28.0 [17.2, 43.7]	7.1 [4.2, 11.4]	20.9 [11.7, 34.9]	113.6 [78.0, 160.7]	26.1 [14.4, 42.6]	58.0 [31.6, 89.7]	14.7 [4.0, 29.4]	104.0 [67.0, 160.1]
	1990 vs. 2021	61% [39.5, 85]	42.2% [11.1, 85.7]	68.5% [40.1, 102.1]	4% [-6.1, 14.5]	21.6% [6.3, 38.7]	3.6% [-7, 15.3]	-25.1% [-43.5, -15.5]	-0.3% [-5.5, 4.9]
	2019 vs. 2021	5.2% [-6.9, 18.6]	0.3% [-19.5, 23.4]	7% [-8, 22.8]	1% [-8.3, 11.4]	1.7% [-9.7, 14.1]	0.1% [-6.7, 9]	-1.7% [-12, 9.3]	0% [-5.1, 5.3]
**Northeast China**									
Liaoning	2021	30.9 [18.2, 48.9]	8.1 [4.6, 13.1]	22.8 [12.3, 38.5]	120.8 [81.6, 169.7]	11.7 [4.8, 23.0]	58.4 [31.2, 92.1]	11.4 [2.4, 24.1]	103.8 [67.3, 156.8]
	1990 vs. 2021	55.8% [34.4, 82.1]	40.4% [10, 76.5]	62.1% [35.3, 92.3]	3.8% [-5.8, 14.8]	11.3% [-11.6, 31.5]	4.1% [-7.5, 15.6]	-19.1% [-34.7, -9.6]	-0.4% [-6.1, 5]
	2019 vs. 2021	3.8% [-7.8, 15.5]	0.5% [-19, 24.8]	5% [-9.8, 22.7]	0.3% [-8.4, 9.1]	1% [-14.1, 18.8]	-0.1% [-9.1, 8.5]	0.2% [-10.9, 13.9]	0.2% [-5.1, 5.6]
Jilin	2021	30.0 [18.2, 47.2]	7.8 [4.7, 12.5]	22.2 [12.4, 36.9]	127.4 [86.9, 179.1]	26.1 [14.5, 42.7]	58.0 [31.6, 92.7]	11.9 [2.6, 24.7]	103.8 [67.0, 157.6]
	1990 vs. 2021	75.4% [53.5, 99.4]	57.1% [22.9, 103.8]	83% [56.4, 115.8]	3.1% [-7.1, 13.8]	21.3% [6, 37.6]	3.8% [-7.1, 15.3]	-31.5% [-47.4, -23.6]	-0.2% [-5.5, 5]
	2019 vs. 2021	4.7% [-5.7, 16.2]	0.1% [-18.9, 23.8]	6.5% [-7.3, 19.7]	0.5% [-7.7, 9.7]	1.9% [-11, 15]	-0.2% [-9.1, 8.2]	-1.6% [-14.9, 11]	-0.1% [-5.1, 5.2]
Heilongjiang	2021	28.1 [17.3, 44.9]	7.6 [4.5, 11.7]	20.5 [11.4, 34.4]	99.2 [66.3, 142.6]	26.1 [14.3, 43.6]	58.0 [31.3, 90.4]	15.1 [4.3, 28.5]	103.5 [66.5, 158.0]
	1990 vs. 2021	55.8% [35.9, 78.3]	44.6% [14.6, 88.3]	60.4% [34.8, 92.3]	0.3% [-9.4, 11.6]	21% [5.5, 38.7]	3.9% [-7.5, 15.8]	-12.1% [-23.5, -0.5]	-0.6% [-6, 5]
	2019 vs. 2021	4.5% [-8.1, 18.9]	0.9% [-17.7, 27.7]	5.8% [-8.9, 21.2]	0.3% [-8.9, 10.8]	1.6% [-11, 15.7]	0% [-7.5, 9.5]	-0.9% [-10, 11]	-0.3% [-5, 5.1]
**Central China**									
Henan	2021	27.1 [16.8, 42.2]	6.5 [3.5, 10.8]	20.7 [11.6, 34.6]	147.8 [102.6, 209.4]	26.3 [14.5, 42.6]	59.0 [32.1, 92.5]	12.6 [3.2, 26.0]	103.4 [65.8, 155.8]
	1990 vs. 2021	75.8% [53.2, 107.4]	46.8% [15.2, 93.1]	87.3% [58.5, 129.2]	5.6% [-4.4, 17.5]	22% [6.1, 39.8]	5.1% [-5.6, 17.2]	-26.2% [-43.8, -4.8]	-0.7% [-5.5, 4.8]
	2019 vs. 2021	4.7% [-7.6, 18.6]	1.1% [-20.7, 28.4]	5.9% [-8.3, 22]	0.7% [-9.2, 11.7]	1.4% [-10.3, 15.7]	-0.2% [-8.7, 9.5]	-1.8% [-14.2, 14.6]	0.1% [-5.5, 5.6]
Hubei	2021	30.4 [18.6, 48.4]	8.0 [4.8, 12.5]	22.5 [12.4, 38.9]	121.4 [83.2, 172.4]	17.9 [8.7, 30.7]	59.3 [33.1, 95.0]	12.8 [3.3, 25.5]	104.0 [66.3, 156.6]
	1990 vs. 2021	87.1% [64.1, 121.7]	65.1% [26.4, 113.5]	96.4% [65.2, 133.4]	6.8% [-3.8, 18.3]	5.6% [-16.5, 27.1]	5.7% [-5.5, 17]	-33.2% [-51.7, -19.1]	-0.1% [-5, 5.1]
	2019 vs. 2021	5.2% [-7.4, 18.6]	0.6% [-21.2, 24.6]	7% [-7.8, 23.2]	0.2% [-9.8, 9.4]	0.8% [-12.1, 13.2]	-0.1% [-6.1, 5.7]	-2.8% [-14.1, 8.6]	0% [-5, 5.8]
Hunan	2021	30.3 [18.0, 47.2]	9.1 [5.6, 14.1]	21.2 [11.7, 35.4]	129.5 [88.1, 181.5]	25.4 [14.5, 40.1]	52.5 [28.8, 82.9]	14.0 [3.8, 27.7]	103.7 [66.4, 158.0]
	1990 vs. 2021	61.7% [38.5, 90.7]	20% [-3.2, 47.9]	90.1% [61.6, 131]	3.8% [-6.8, 15.6]	-3.8% [-16, 11.3]	13% [-1.9, 29.1]	-28.8% [-46, -11.9]	-0.3% [-5.2, 5.4]
	2019 vs. 2021	4.4% [-7, 17.4]	-0.1% [-19.1, 23.5]	6.5% [-7.3, 25]	0.2% [-8.7, 10.5]	0% [-10.4, 13.2]	-0.2% [-8.4, 9.3]	-1.8% [-13.3, 9]	-0.1% [-5.1, 5.3]

**Southwest China**									
Chongqing	2021	31.0 [19.0, 48.9]	7.7 [4.6, 12.8]	23.3 [12.9, 38.7]	131.8 [90.5, 187.4]	26.1 [14.5, 43.1]	58.4 [31.1, 92.9]	10.9 [2.1, 22.8]	103.7 [66.7, 156.7]
	1990 vs. 2021	76.6% [54.4, 104.1]	53.4% [21.4, 93]	85.8% [55.4, 122.3]	4.5% [-6.3, 16.4]	20.8% [5.8, 37.8]	4.3% [-6.4, 16.6]	-38% [-57.8, -28.4]	-0.4% [-5.7, 5]
	2019 vs. 2021	5.6% [-6.5, 19.6]	1.8% [-17.4, 25.1]	6.9% [-7.8, 22.7]	0.2% [-8.8, 9.1]	1.5% [-11, 14.5]	-0.1% [-9.3, 9.3]	-4% [-17.3, 9.2]	0.2% [-5.2, 5.2]
Sichuan	2021	27.2 [16.2, 43.3]	7.0 [4.2, 11.0]	20.2 [11.0, 34.2]	86.8 [57.3, 124.9]	28.4 [16.0, 44.5]	58.1 [32.0, 91.6]	16.5 [5.5, 31.0]	103.8 [66.6, 158.9]
	1990 vs. 2021	86.8% [60.8, 115.5]	61.7% [24.1, 106.8]	97.4% [63.9, 132]	1.4% [-8.1, 10.8]	-7.8% [-21.1, 5.6]	3.7% [-6.1, 15.5]	-28.4% [-45.3, -8.8]	-0.4% [-5.5, 5]
	2019 vs. 2021	4.5% [-7.7, 18.5]	0.7% [-19, 26.4]	5.9% [-10.1, 23.4]	0.3% [-10.6, 10.9]	0.6% [-10, 12]	-0.2% [-8.5, 8.6]	-1.7% [-11, 8.2]	0.2% [-4.6, 5.2]
Guizhou	2021	25.7 [15.3, 41.4]	6.6 [3.8, 10.9]	19.1 [10.4, 32.5]	78.1 [51.5, 112.4]	26.3 [14.5, 43.1]	58.5 [32.5, 92.4]	20.1 [8.0, 36.9]	103.5 [66.0, 158.2]
	1990 vs. 2021	90.5% [66.6, 120.1]	65.4% [30.2, 111.9]	101% [67.6, 136.8]	-0.2% [-9.9, 11.8]	21.4% [6.1, 37.3]	4.1% [-5.2, 16.4]	-33.4% [-49.8, -16.6]	-0.2% [-5.5, 4.8]
	2019 vs. 2021	6% [-6.5, 20.3]	1.8% [-19.4, 30.5]	7.6% [-7.9, 25.5]	0.7% [-8.4, 10.6]	1.6% [-10.9, 15.1]	-0.2% [-8.3, 7.9]	-2.8% [-12.8, 7.7]	-0.3% [-5.7, 4.6]
Yunnan	2021	25.4 [15.4, 40.3]	6.6 [3.9, 10.6]	18.8 [10.6, 31.7]	114.5 [77.5, 159.6]	26.2 [14.3, 42.7]	58.2 [32.1, 89.9]	17.7 [5.6, 34.5]	104.0 [66.9, 159.6]
	1990 vs. 2021	65.4% [42.4, 92.2]	47% [13, 99.1]	72.9% [45.3, 108.3]	5.5% [-4.2, 17.5]	21.7% [7, 40.2]	4.2% [-6, 16.2]	-18.6% [-38.7, 5.1]	0.4% [-5, 6]
	2019 vs. 2021	5% [-6.9, 17.9]	0.5% [-17.4, 23.6]	6.7% [-8.5, 22.1]	0.6% [-8.5, 9.6]	1.6% [-11.1, 15.7]	-0.1% [-7.7, 8.8]	-2.3% [-12, 8]	-0.2% [-5.1, 5.3]
Tibet	2021	25.8 [15.4, 40.3]	6.5 [4.1, 10.4]	19.3 [10.4, 32.0]	109.8 [74.2, 157.1]	26.2 [14.1, 43.0]	57.8 [31.4, 88.8]	17.3 [5.6, 34.0]	104.6 [67.2, 158.0]
	1990 vs. 2021	77.5% [54.7, 108.1]	53.1% [16.7, 104]	87.6% [59.8, 128.9]	3.8% [-6.7, 14.3]	22.8% [7.4, 39.6]	4.1% [-5.5, 16.2]	-28.8% [-48.5, -7]	1.7% [-4.2, 7.5]
	2019 vs. 2021	5.4% [-6.5, 22.4]	1.1% [-20.6, 32.1]	6.9% [-8.1, 24.5]	0.9% [-8, 10.6]	1.5% [-12.1, 15]	0.1% [-8.8, 8.3]	-2.7% [-15.5, 9.2]	0.1% [-5.4, 5.9]
