# Supplementary material for: The disability-adjusted life years attributable to mental disorders and self-harm in China from 1990–2021: Findings from the global burden of disease study 2021
Source: PLOS Ment Health. 2025 Apr 9;2(4):e0000146. doi: 10.1371/journal.pmen.0000146 (PMC12798377; doi:10.1371/journal.pmen.0000146)

Figure S1 Trends of age-sex-specific mortality, DALYs, YLDs, YLLs, prevalence and incidence rate of self harm in China, 1990-2021

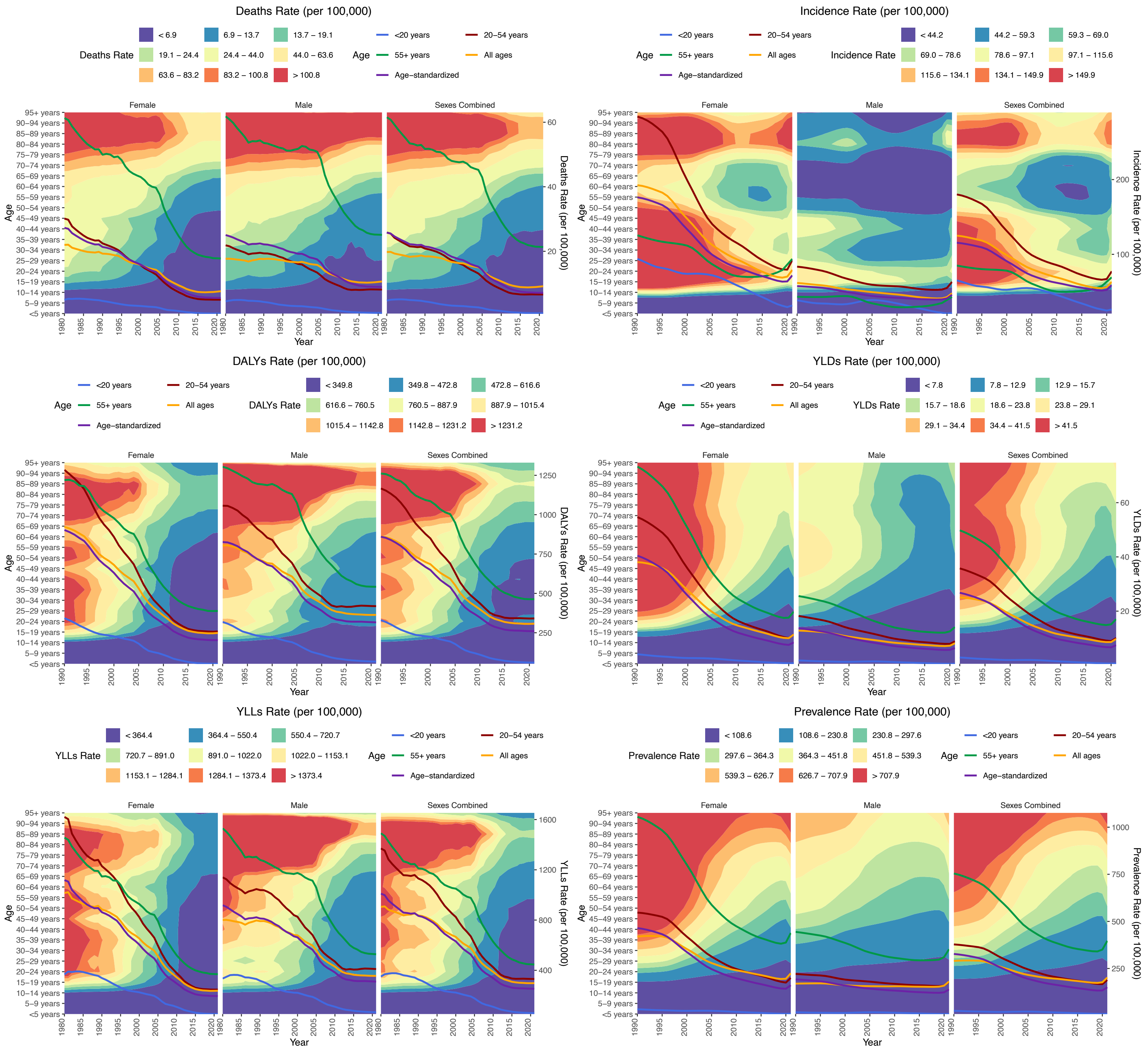

Figure S1 Trends of age-sex-specific mortality, DALYs, YLDs, YLLs, prevalence and incidence rate of mental disorders in China, 1990-2021

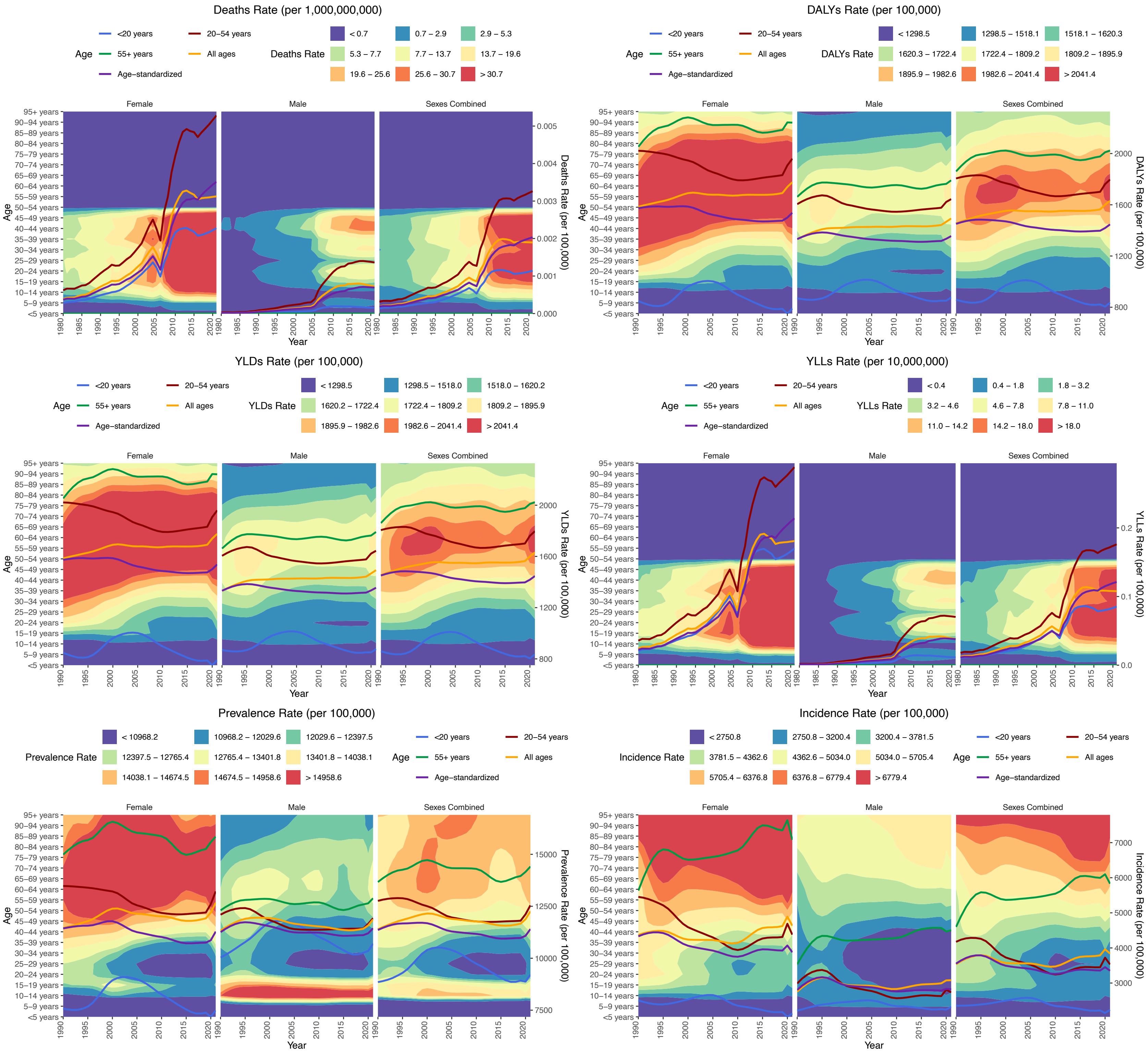

Figure S1 Trends of age-sex-specific DALYs, YLDs, prevalence and incidence rate of depressive disorders in China, 1990-2021

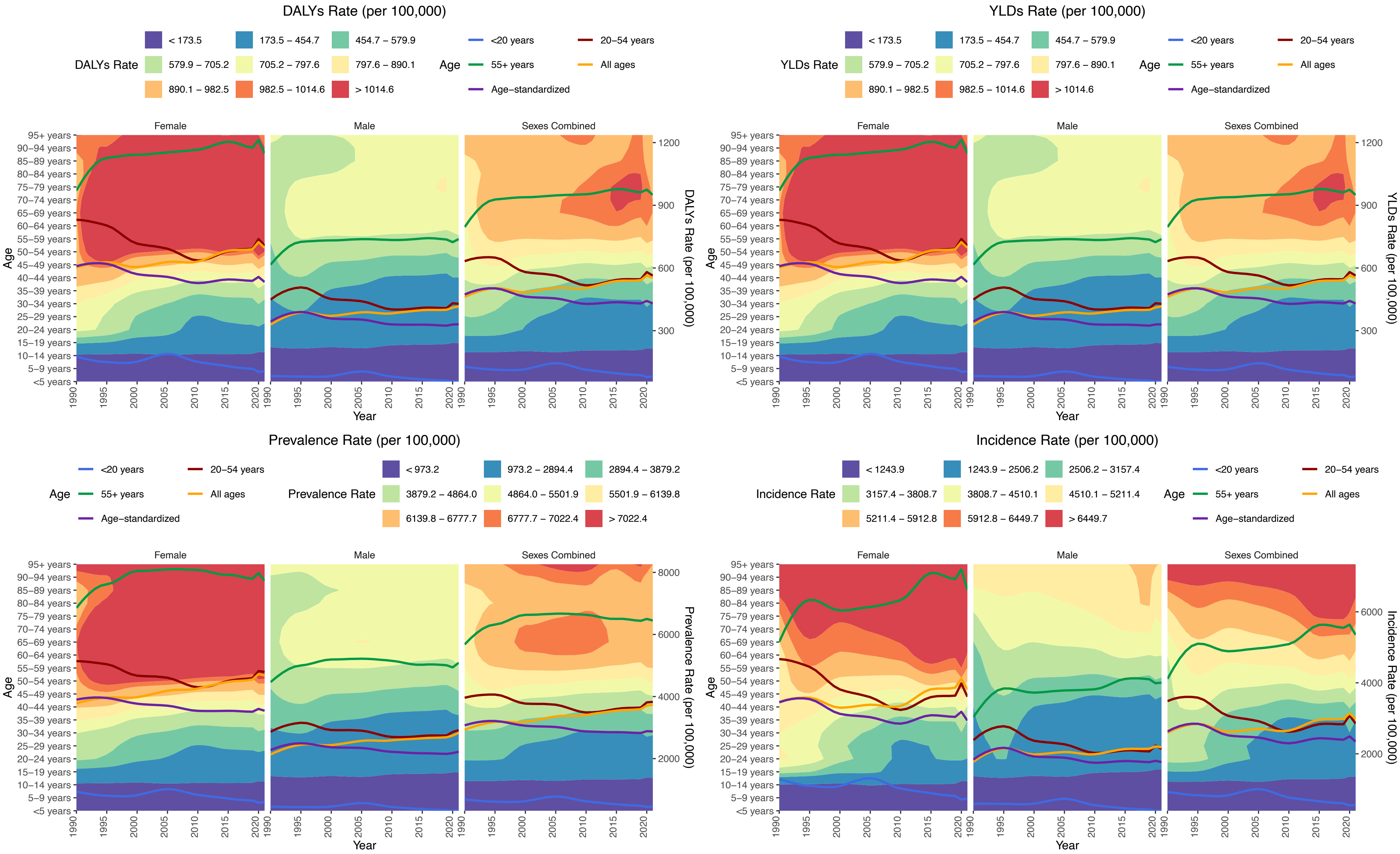

Figure S1 Trends of age-sex-specific DALYs, YLDs, prevalence and incidence rate of major depressive disorder in China, 1990-2021

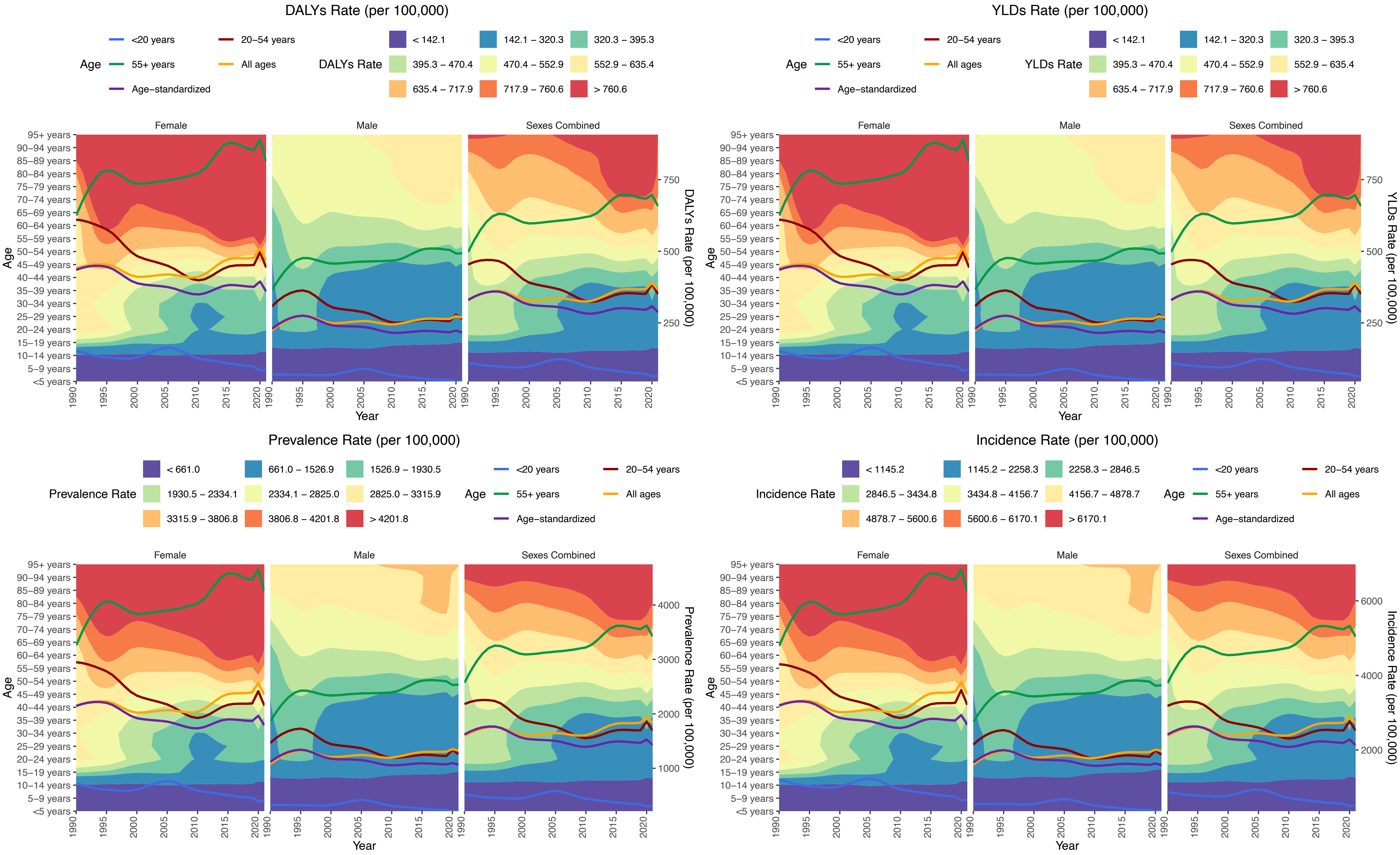

Figure S1 Trends of age-sex-specific DALYs, YLDs, prevalence and incidence rate of dysthymia in China, 1990-2021

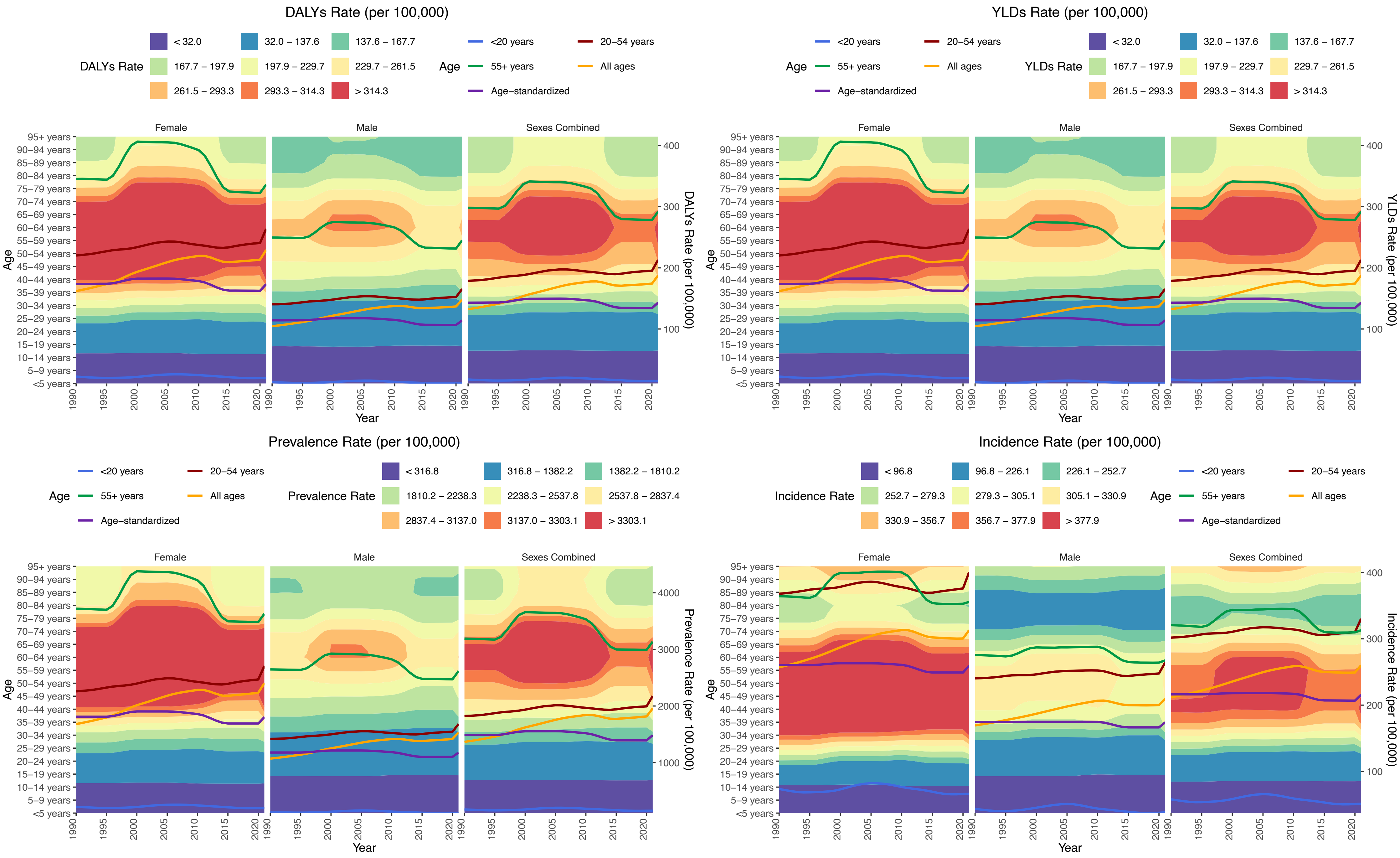

Figure S1 Trends of age-sex-specific DALYs, YLDs, prevalence and incidence rate of anxiety disorders in China, 1990-2021

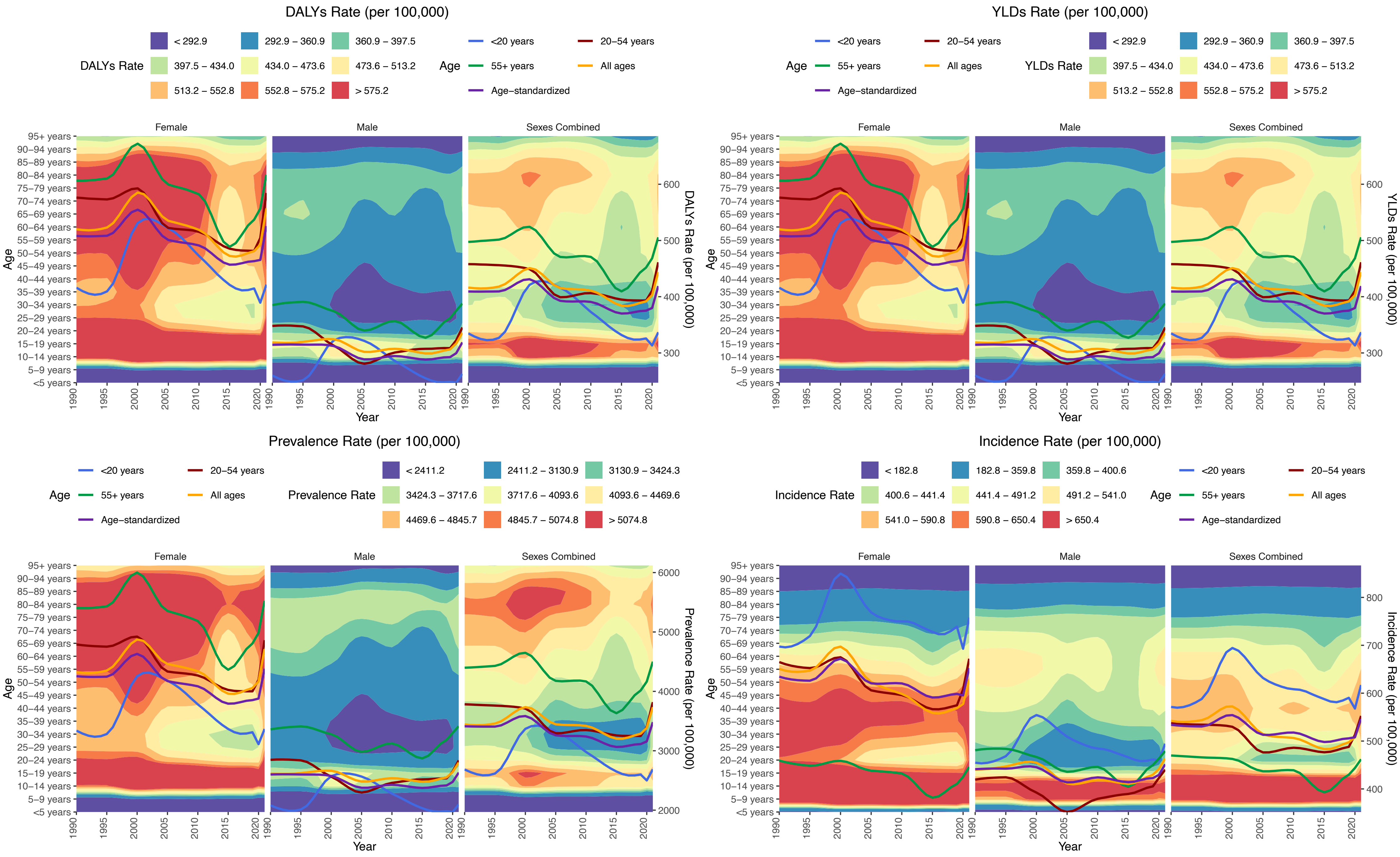

Figure S1 Trends of age-sex-specific DALYs, YLDs, prevalence and incidence rate of schizophrenia in China, 1990-2021

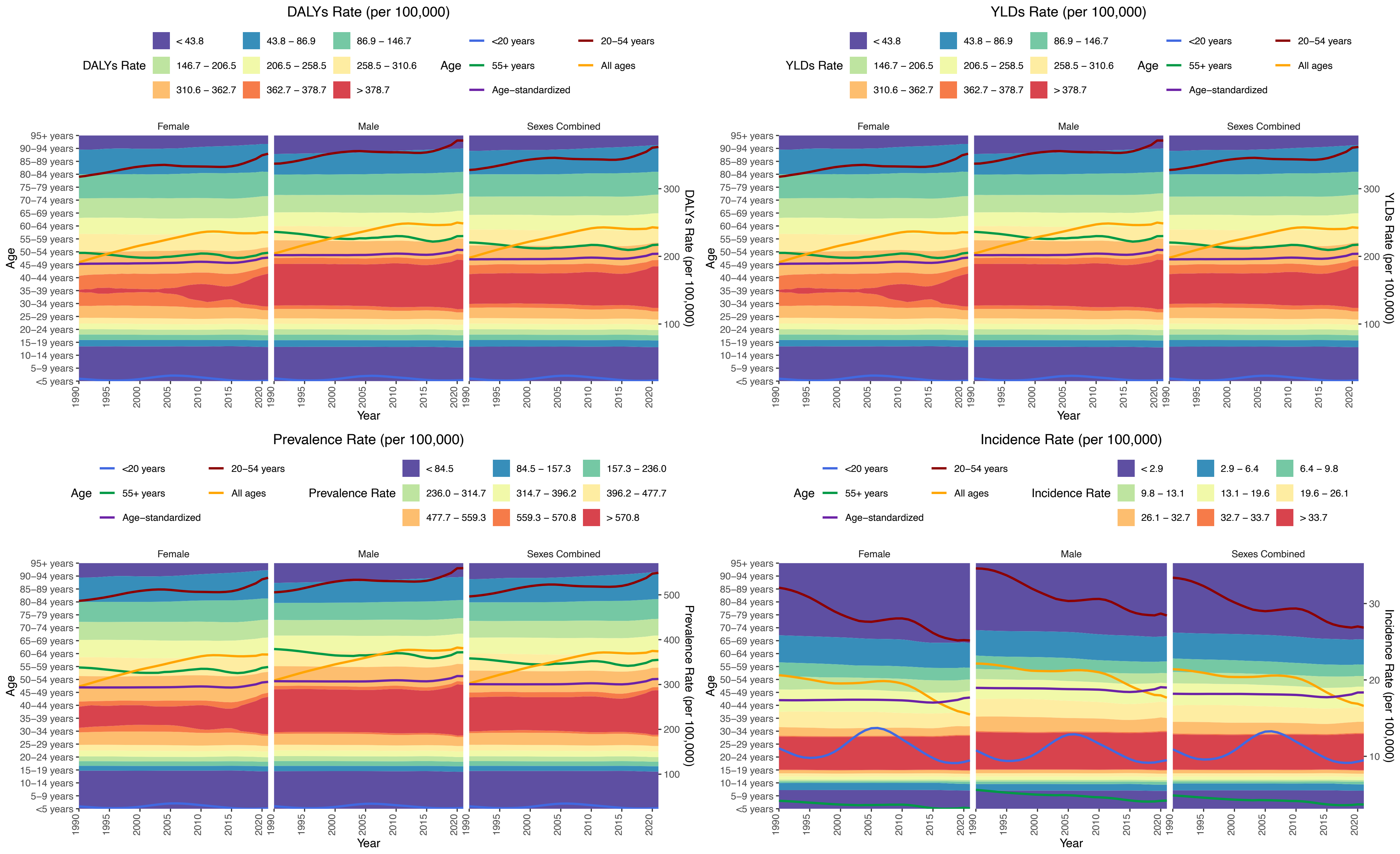

Figure S1 Trends of age-sex-specific DALYs, YLDs, prevalence and incidence rate of bipolar disorder in China, 1990-2021

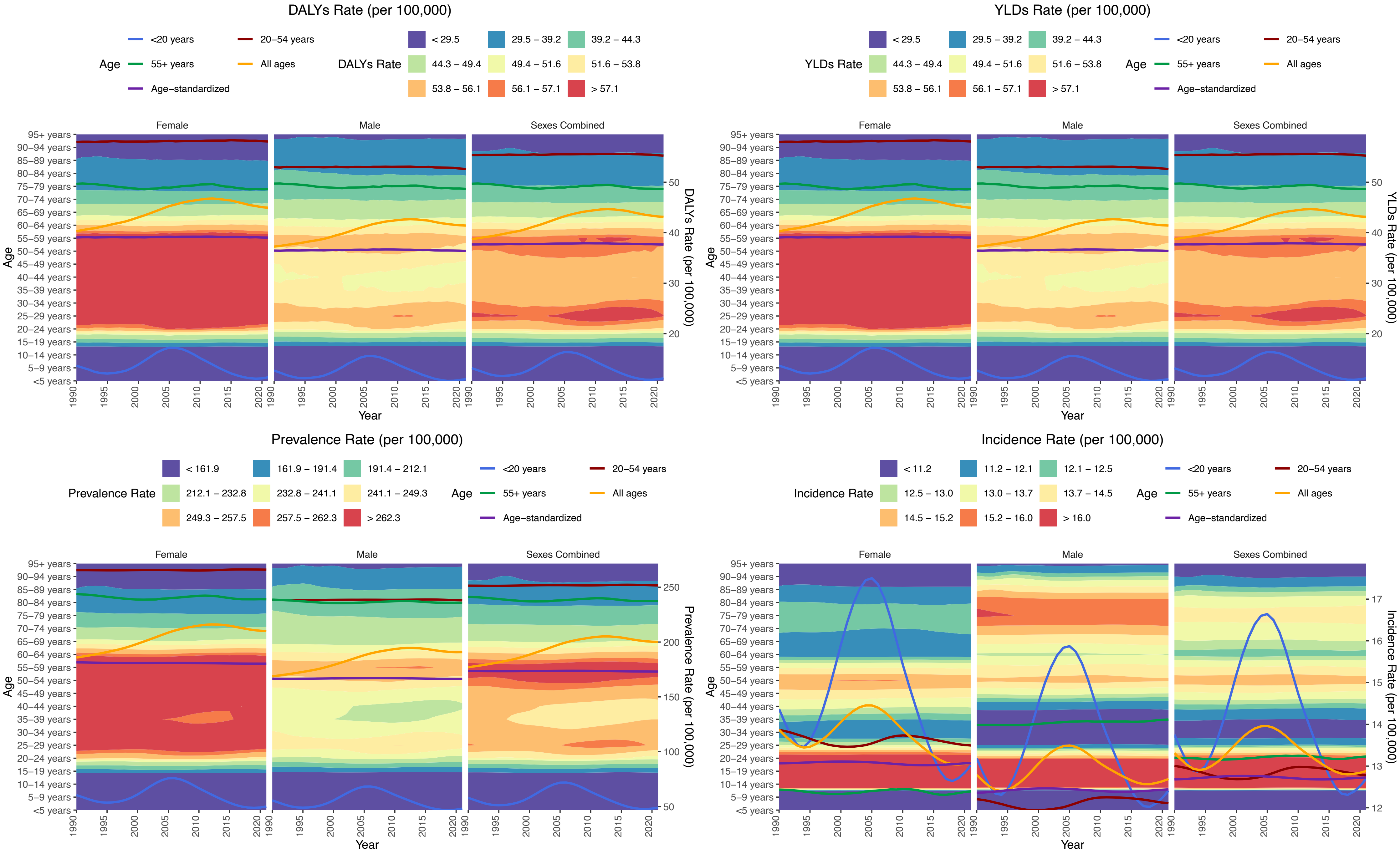

Figure S1 Trends of age-sex-specific mortality, DALYs, YLDs, YLLs, prevalence and incidence rate of eating disorders in China, 1990-2021

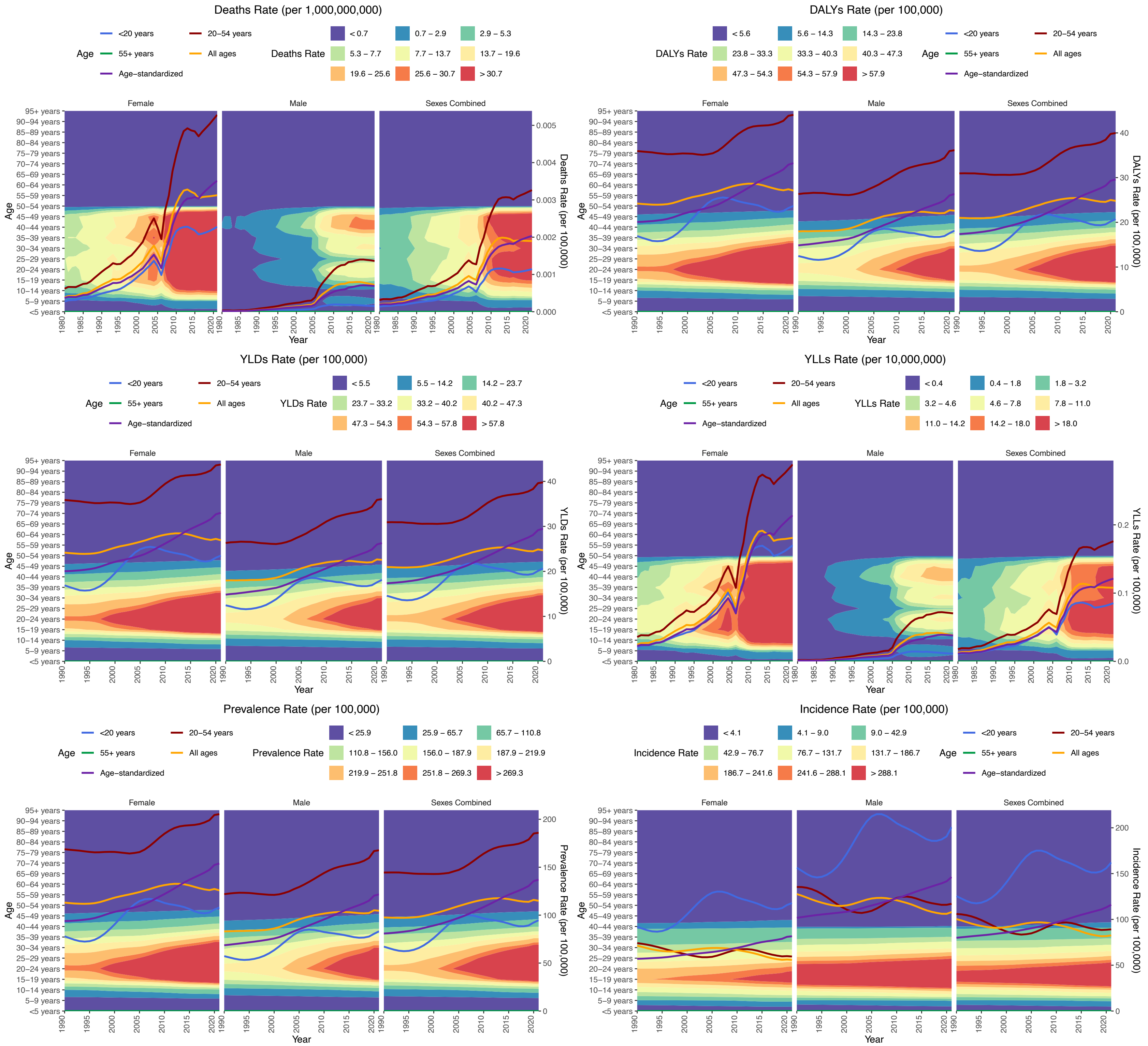

Figure S1 Trends of age-sex-specific mortality, DALYs, YLDs, YLLs, prevalence and incidence rate of anorexia nervosa in China, 1990-2021

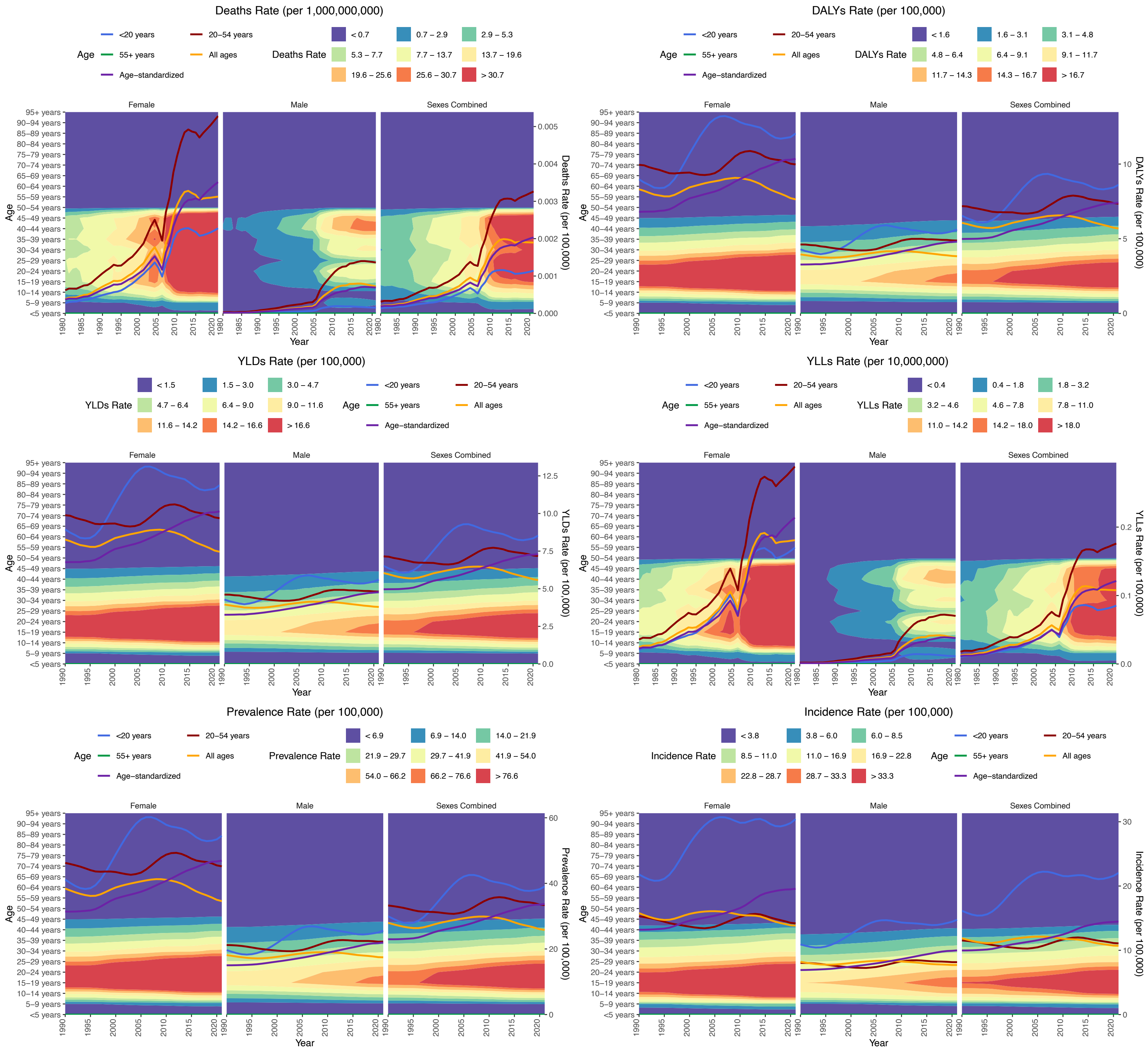

Figure S1 Trends of age-sex-specific DALYs, YLDs, prevalence and incidence rate of bulimia nervosa in China, 1990-2021

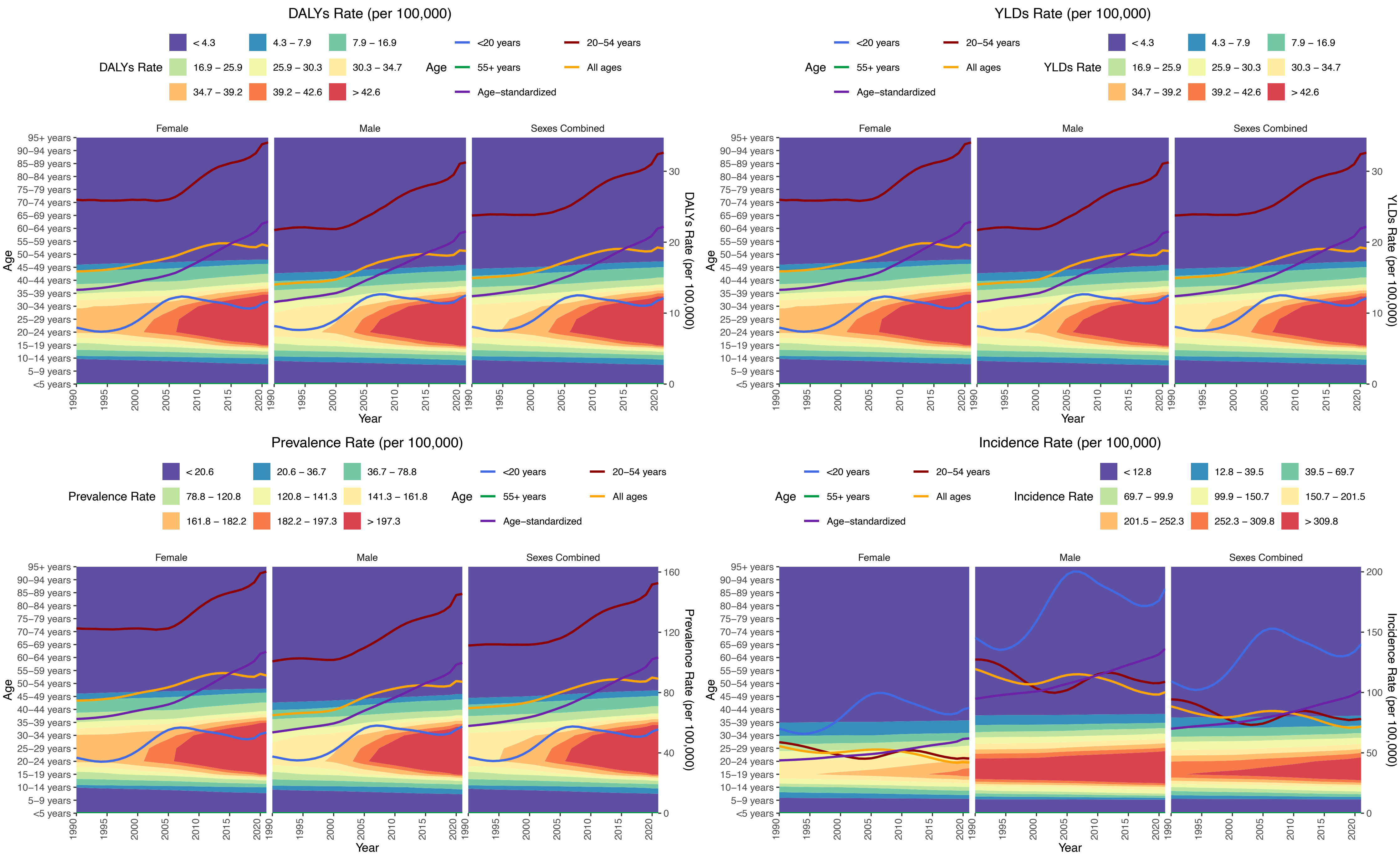

Figure S1 Trends of age-sex-specific DALYs, YLDs, prevalence and incidence rate of autism spectrum disorders in China, 1990-2021

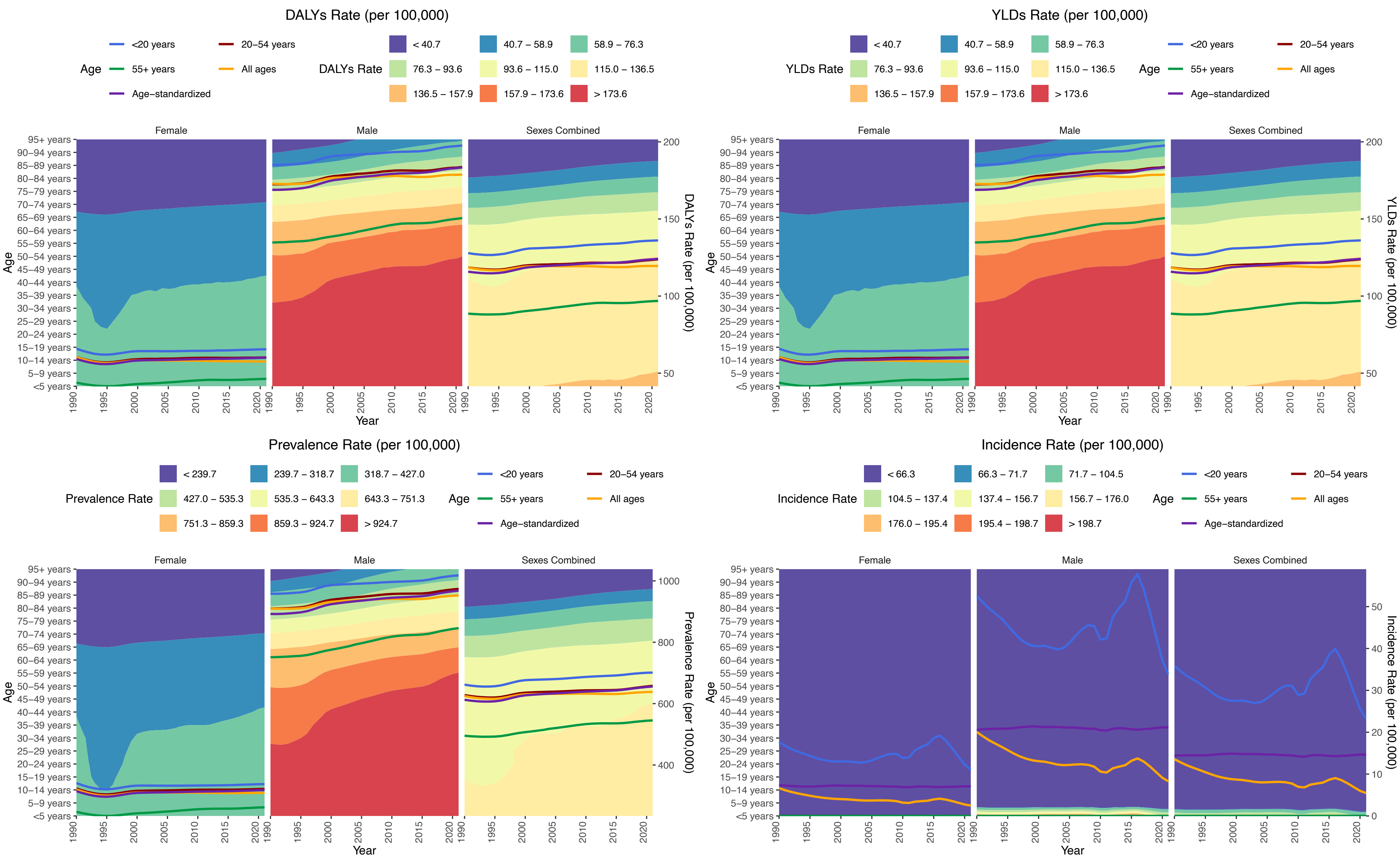

Figure S1 Trends of age-sex-specific DALYs, YLDs, prevalence and incidence rate of attention deficit and hyperactivity in China, 1990-2021

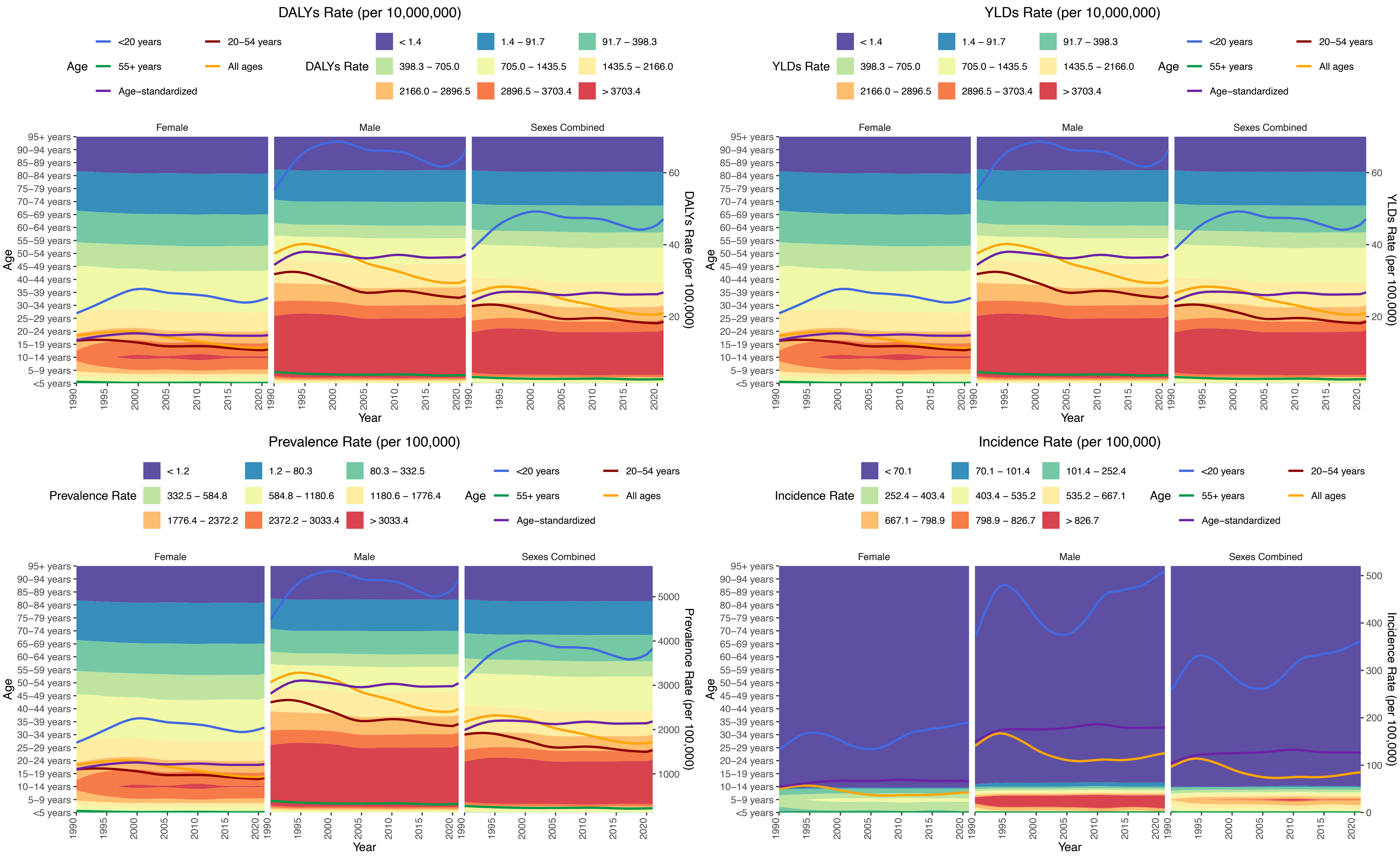

Figure S1 Trends of age-sex-specific DALYs, YLDs, prevalence and incidence rate of conduct disorder in China, 1990-2021

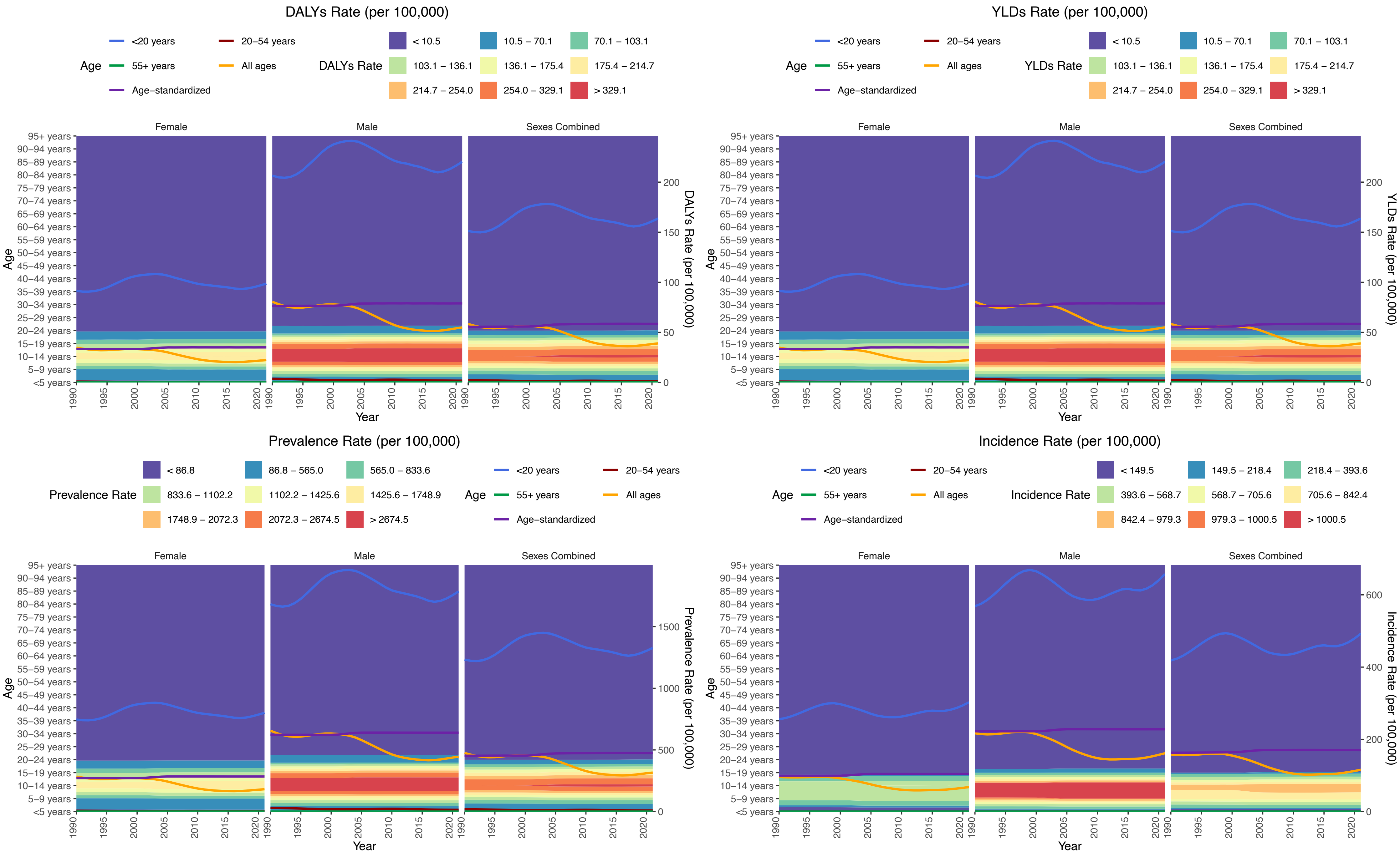

Figure S1 Trends of age-sex-specific DALYs, YLDs, prevalence rate of idiopathic developmental intellectual disability in China, 1990-2021

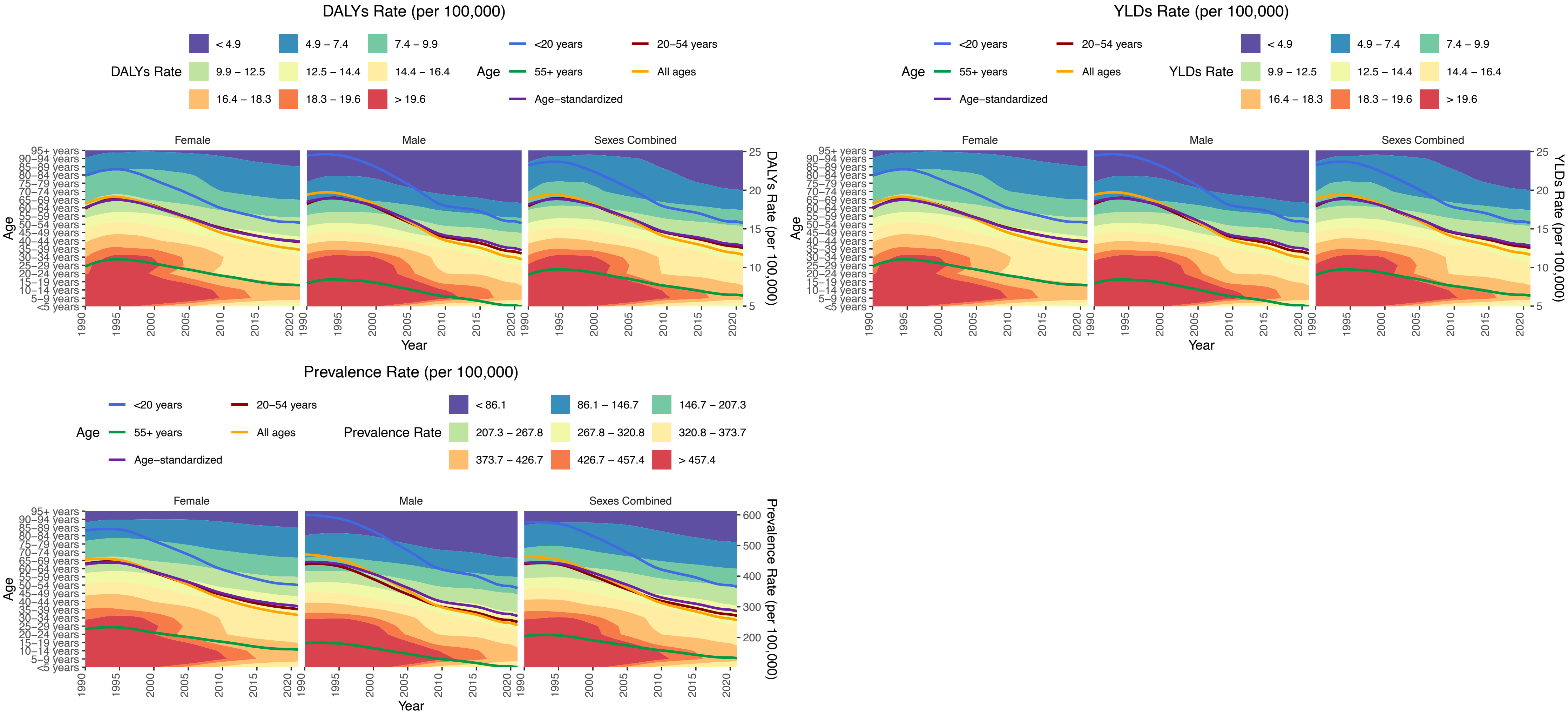

Figure S1 Trends of age-sex-specific DALYs, YLDs, prevalence rate of other mental disorders in China, 1990-2021

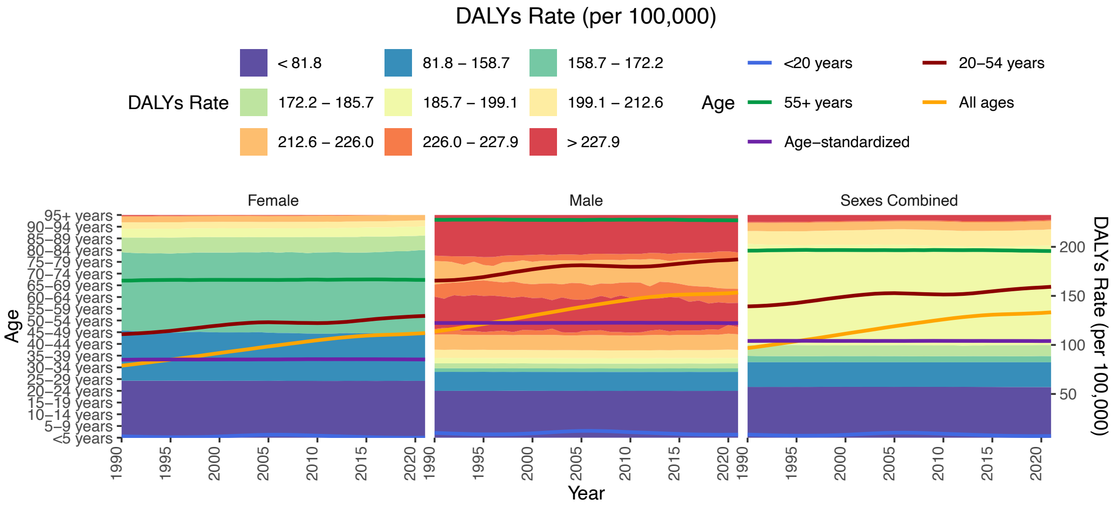

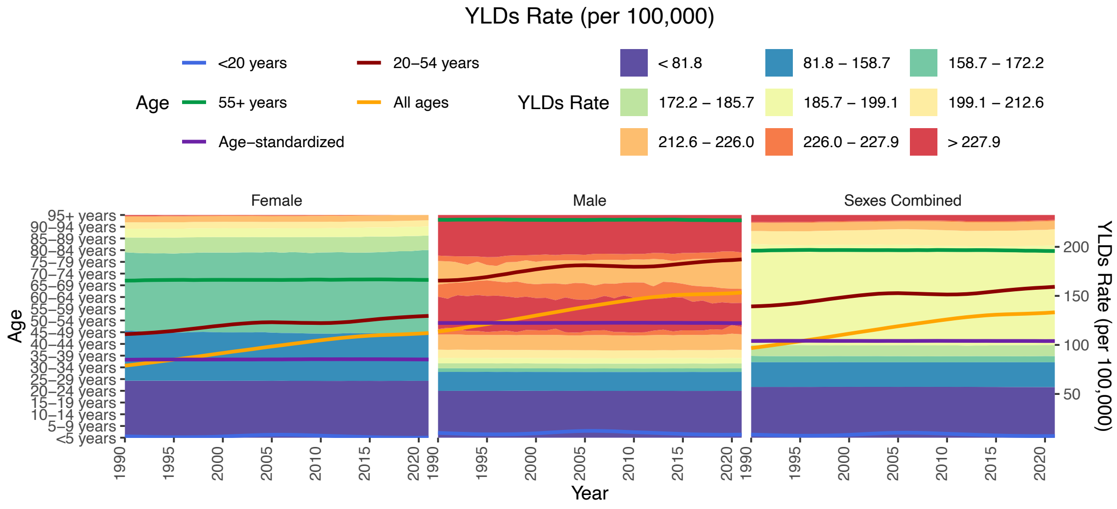

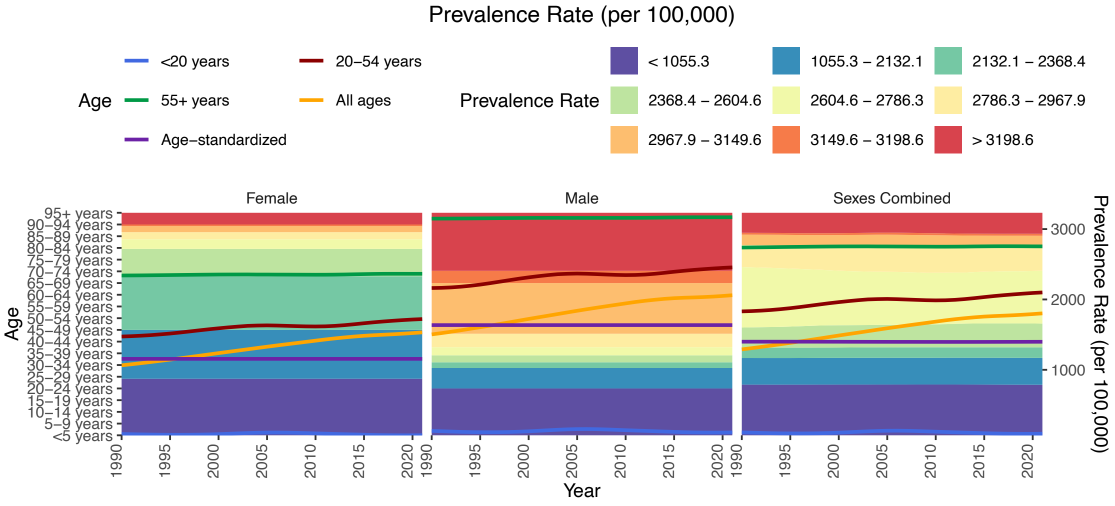

Supplement: S1 Fig — (PDF) [file pmen.0000146.s006.pdf]
