# Supplementary material for: The disability-adjusted life years attributable to mental disorders and self-harm in China from 1990–2021: Findings from the global burden of disease study 2021
Source: PLOS Ment Health. 2025 Apr 9;2(4):e0000146. doi: 10.1371/journal.pmen.0000146 (PMC12798377; doi:10.1371/journal.pmen.0000146)

Figure S2 Spatial distribution of mortality, incidence, DALYs, YLDs, YLLs, prevalence rate of self harm in China, 2021

National Distribution of Deaths Rate Attributed to
Self−harm among Females and Males (Age−standardized), 2021

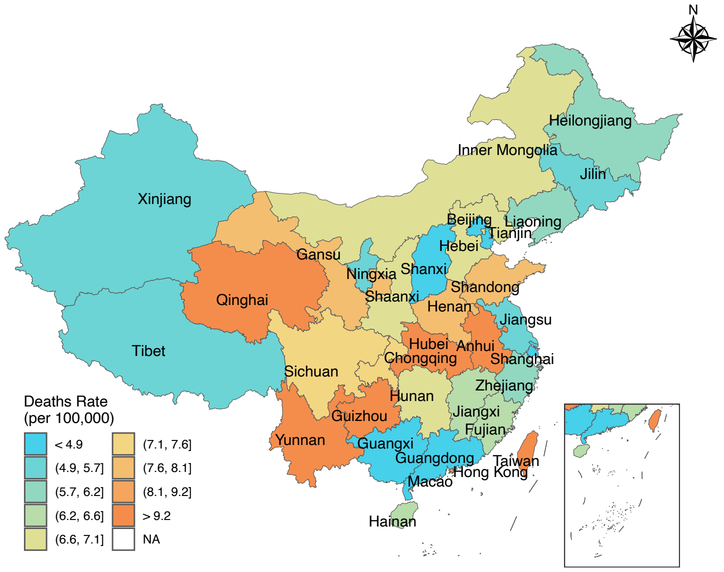


National Distribution of Incidence Rate Attributed to
Self−harm among Females and Males (Age−standardized), 2021

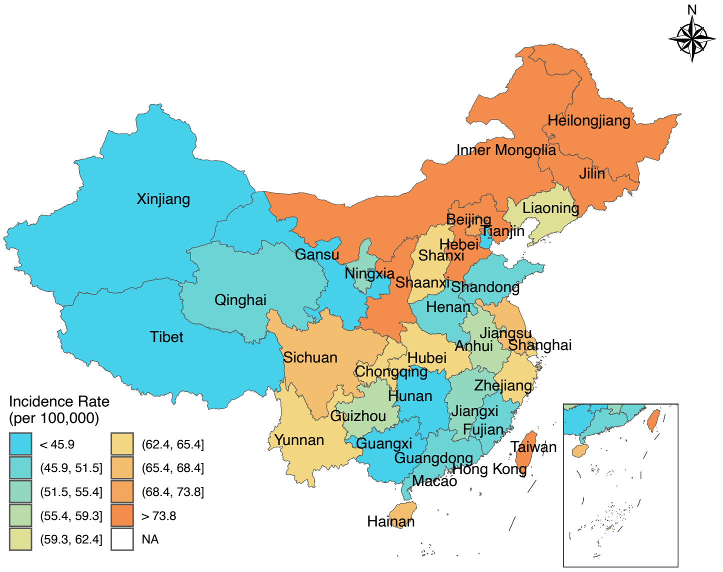


National Distribution of DALYs Rate Attributed to
Self−harm among Females and Males (Age−standardized), 2021

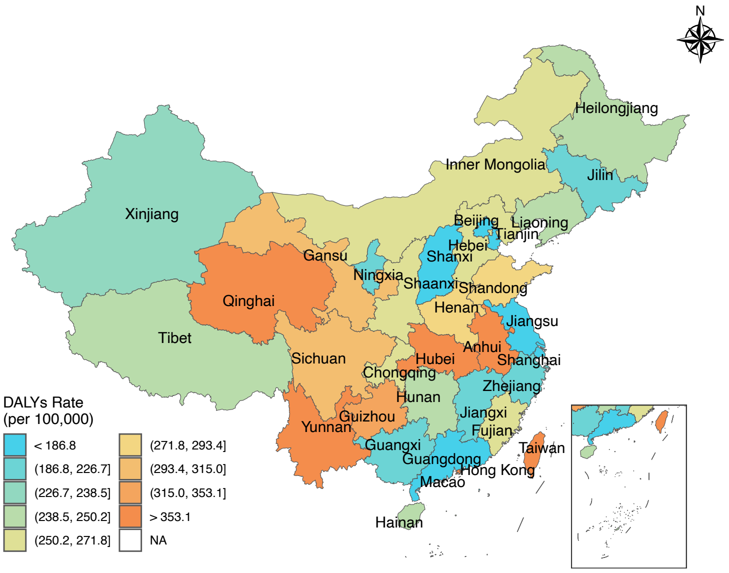


National Distribution of YLDs Rate Attributed to
Self−harm among Females and Males (Age−standardized), 2021

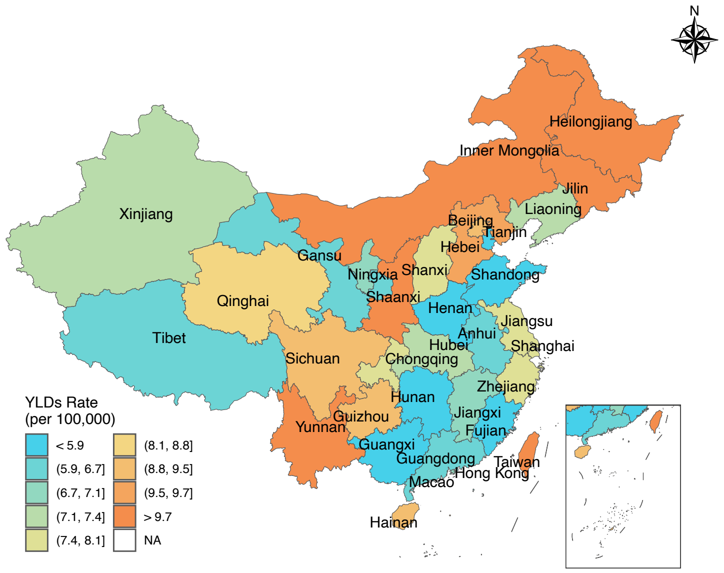


National Distribution of YLLs Rate Attributed to
Self−harm among Females and Males (Age−standardized), 2021

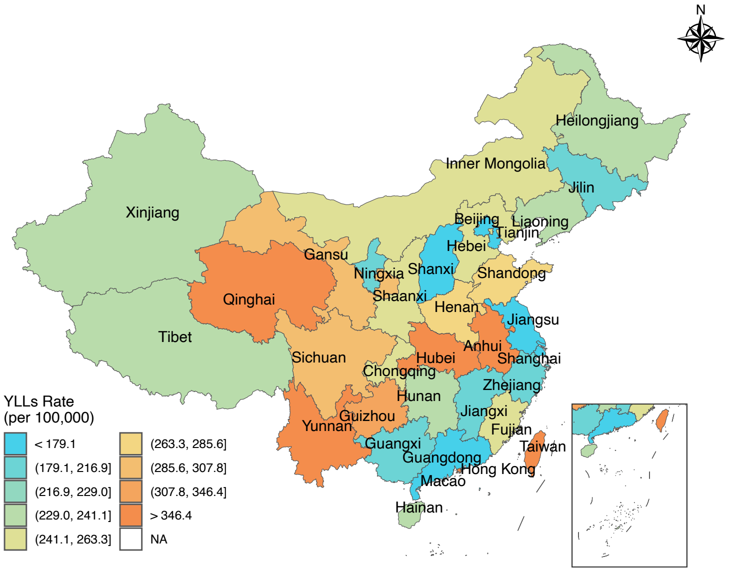


National Distribution of Prevalence Rate Attributed to
Self−harm among Females and Males (Age−standardized), 2021

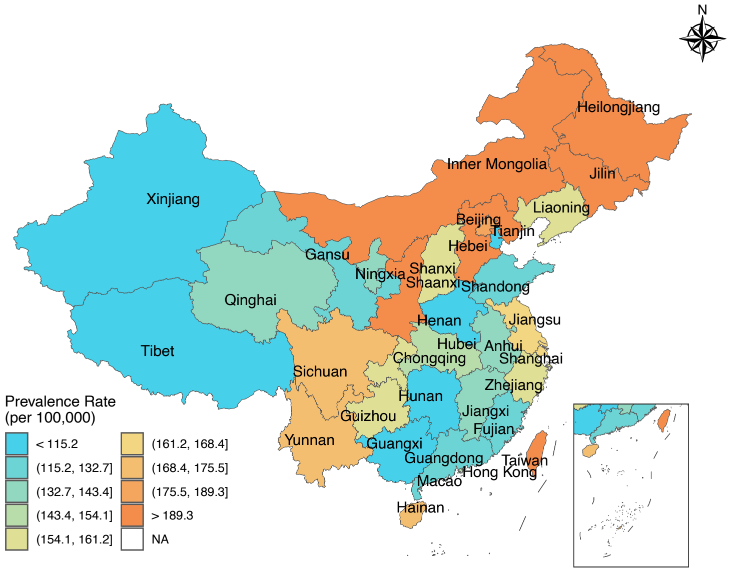

## Figure S2 Spatial distribution of mortality, incidence, DALYs, YLDs, YLLs, prevalence rate of mental dissorders in China, 2021

National Distribution of Deaths Rate Attributed to
Mental disorders among Females and Males (Age−standardized), 2021

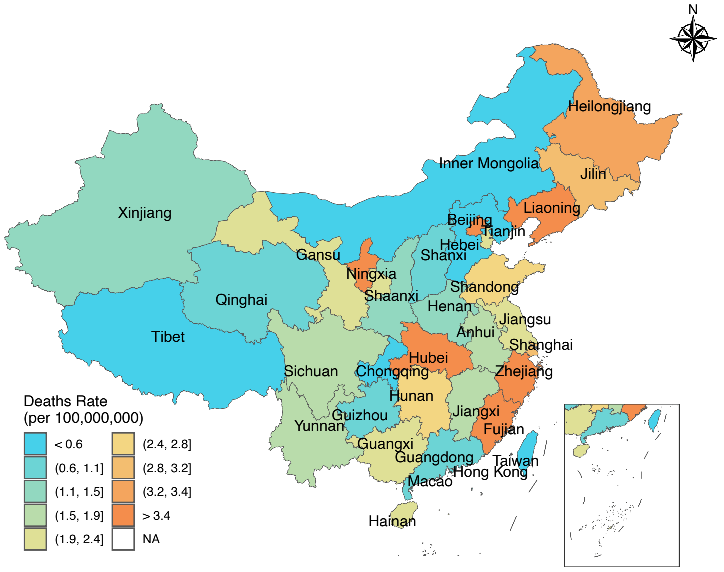


National Distribution of DALYs Rate Attributed to
Mental disorders among Females and Males (Age−standardized), 2021

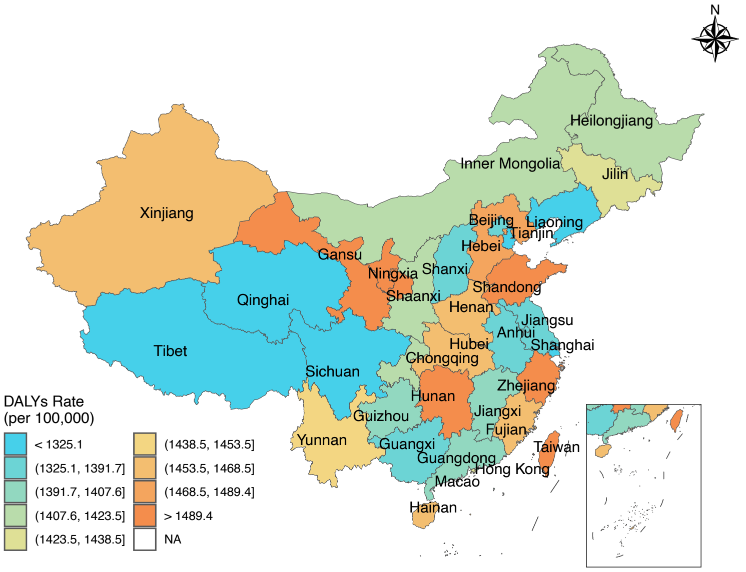


National Distribution of YLDs Rate Attributed to
Mental disorders among Females and Males (Age−standardized), 2021

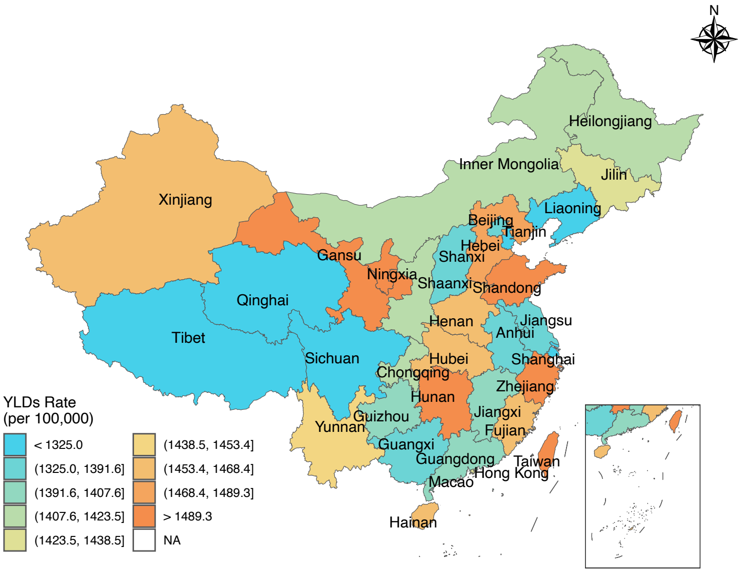


National Distribution of YLLs Rate Attributed to
Mental disorders among Females and Males (Age−standardized), 2021

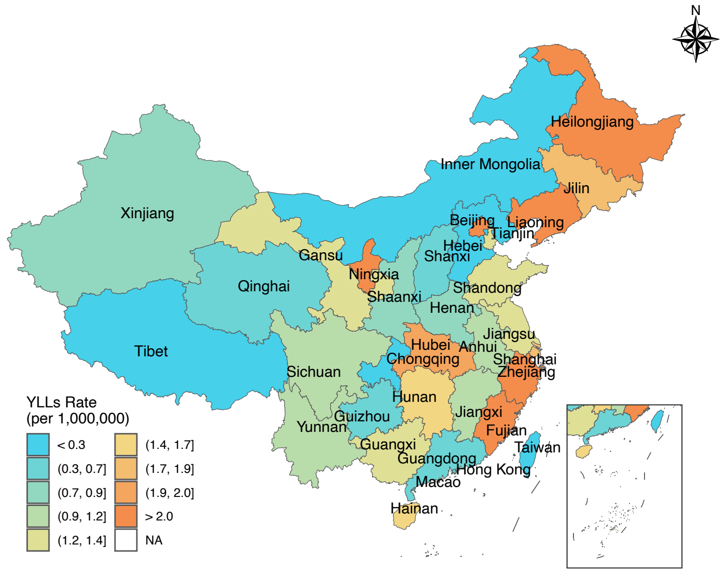


National Distribution of Prevalence Rate Attributed to
Mental disorders among Females and Males (Age−standardized), 2021

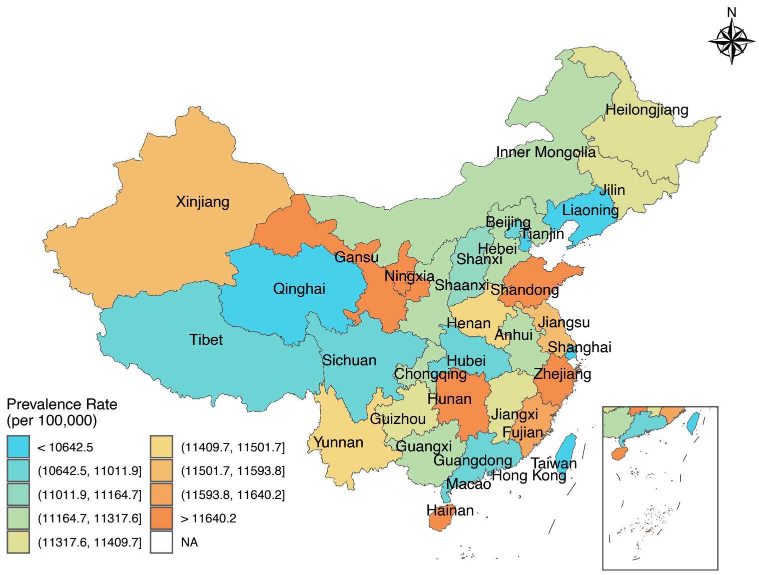


National Distribution of Incidence Rate Attributed to
Mental disorders among Females and Males (Age−standardized), 2021

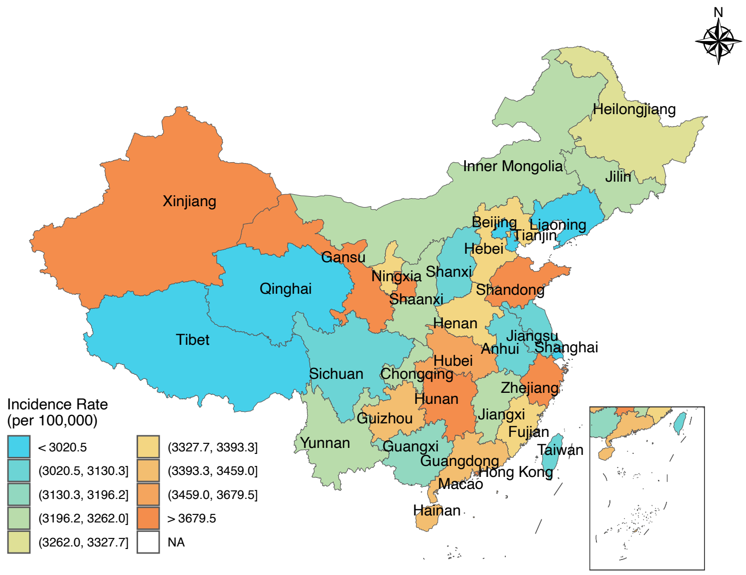

Figure S2 Spatial distribution of DALYs, YLDs, incidence, prevalence rate of depressive disorders in China, 2021

National Distribution of DALYs Rate Attributed to
Depressive disorders among Females and Males (Age−standardized), 2021

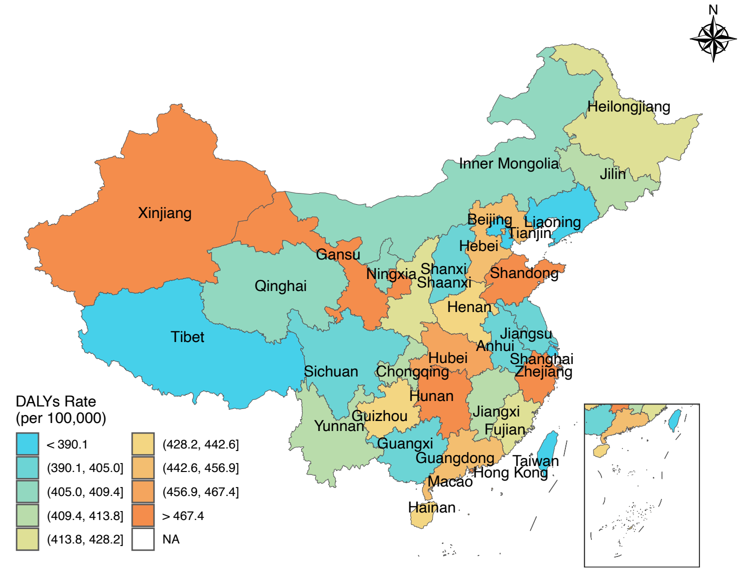


National Distribution of YLDs Rate Attributed to
Depressive disorders among Females and Males (Age−standardized), 2021

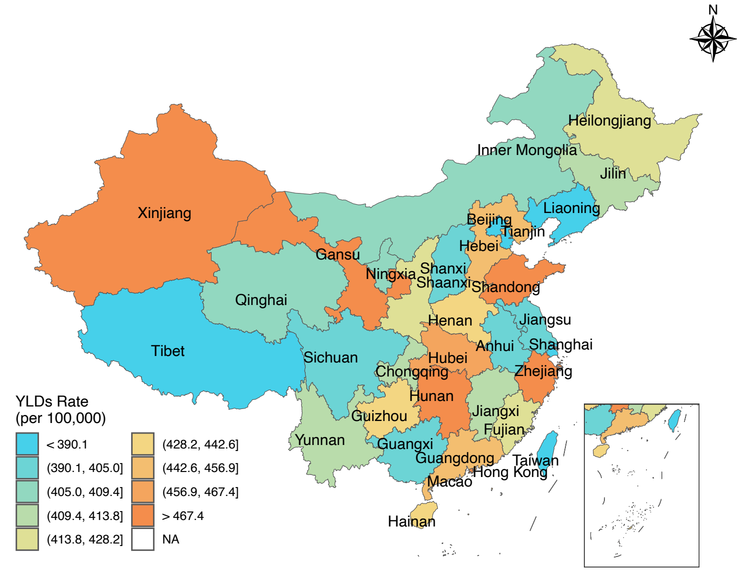


National Distribution of Incidence Rate Attributed to
Depressive disorders among Females and Males (Age−standardized), 2021

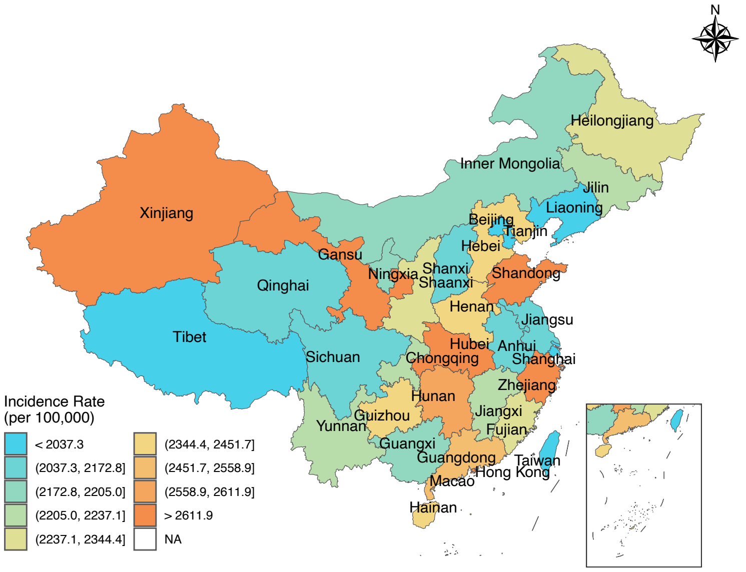


National Distribution of Prevalence Rate Attributed to
Depressive disorders among Females and Males (Age−standardized), 2021

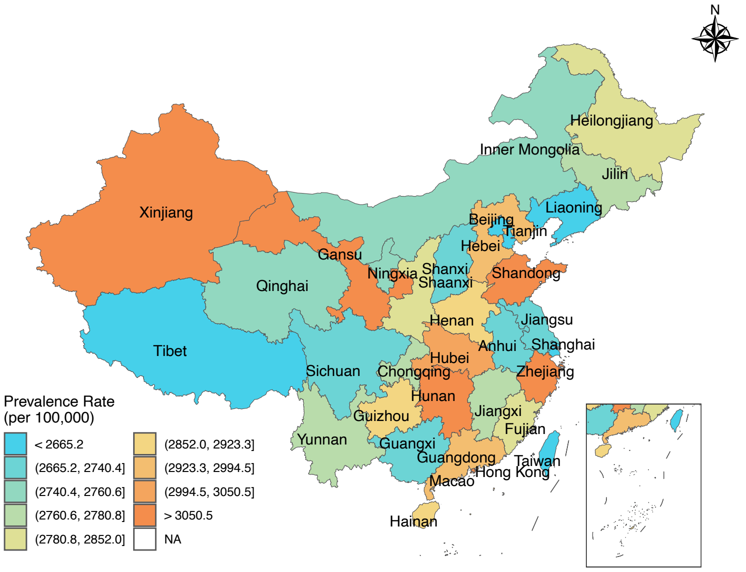

Figure S2 Spatial distribution of DALYs, YLDs, incidence, prevalence rate of major depressive disorder in China, 2021

National Distribution of DALYs Rate Attributed to
Major depressive disorder among Females and Males (Age−standardized), 2021

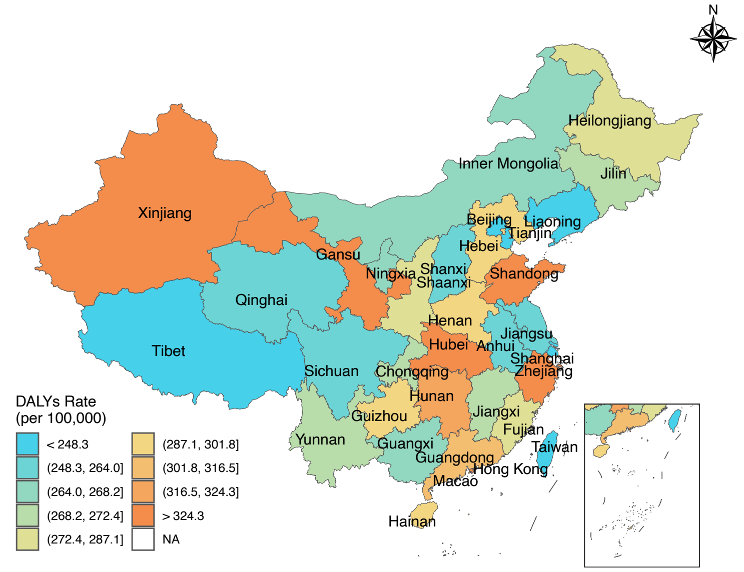


National Distribution of YLDs Rate Attributed to
Major depressive disorder among Females and Males (Age−standardized), 2021

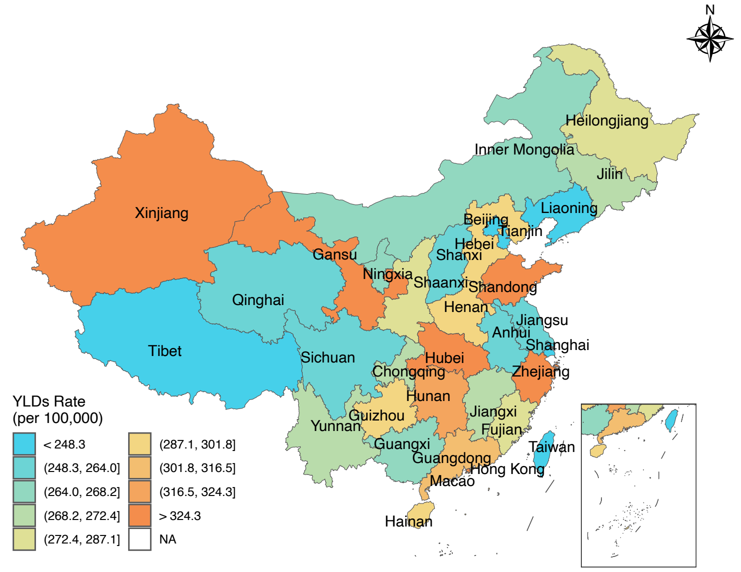


National Distribution of Incidence Rate Attributed to
Major depressive disorder among Females and Males (Age−standardized), 2021

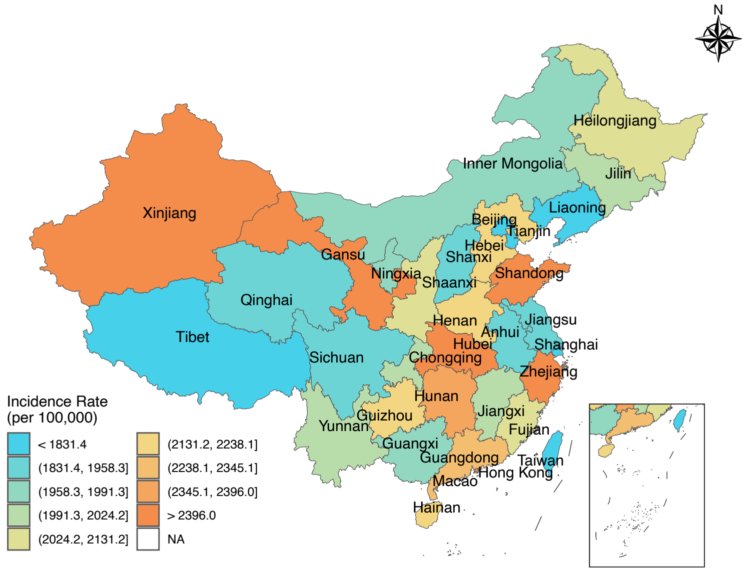


National Distribution of Prevalence Rate Attributed to
Major depressive disorder among Females and Males (Age−standardized), 2021

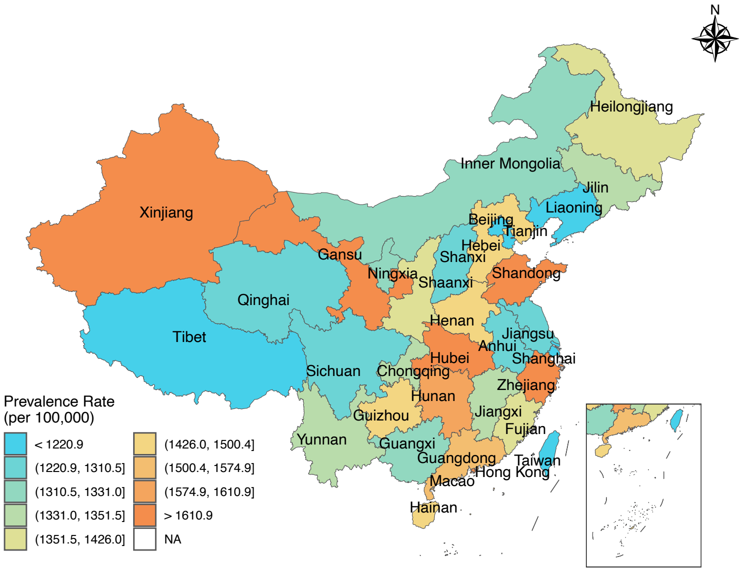

Figure S2 Spatial distribution of DALYs, YLDs, incidence, prevalence rate of dysthymia in China, 2021

National Distribution of DALYs Rate Attributed to
Dysthymia among Females and Males (Age−standardized), 2021

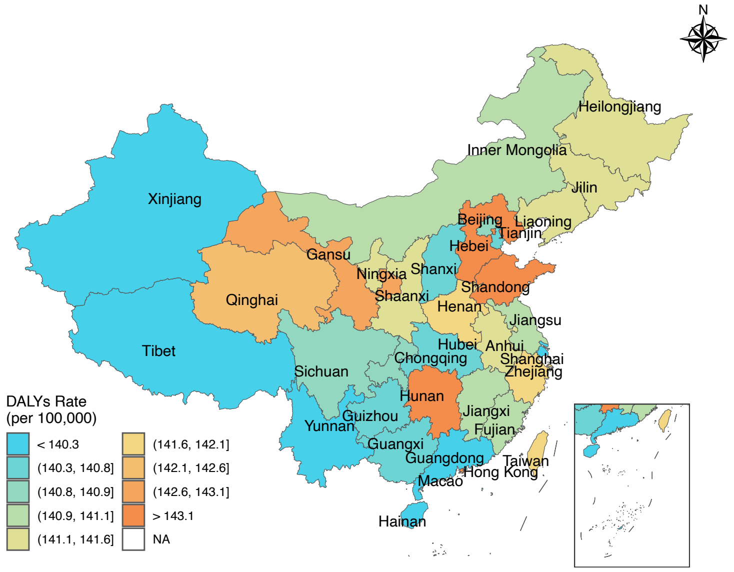


National Distribution of YLDs Rate Attributed to
Dysthymia among Females and Males (Age−standardized), 2021

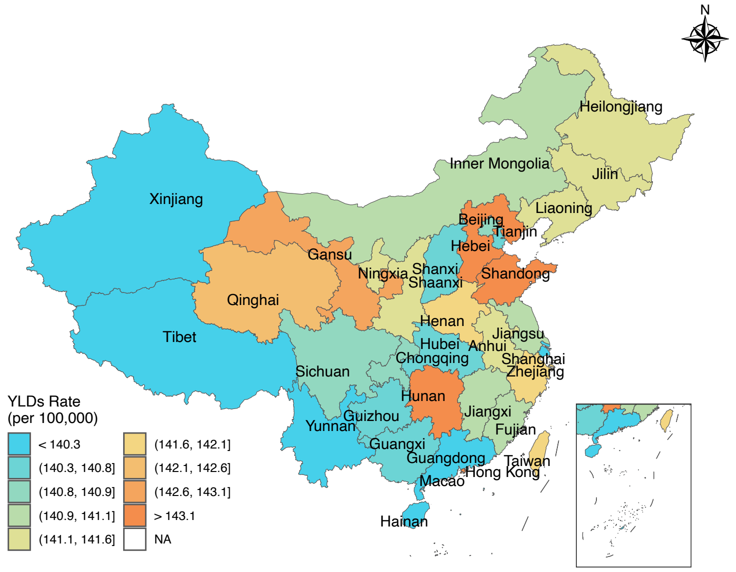


National Distribution of Incidence Rate Attributed to
Dysthymia among Females and Males (Age−standardized), 2021

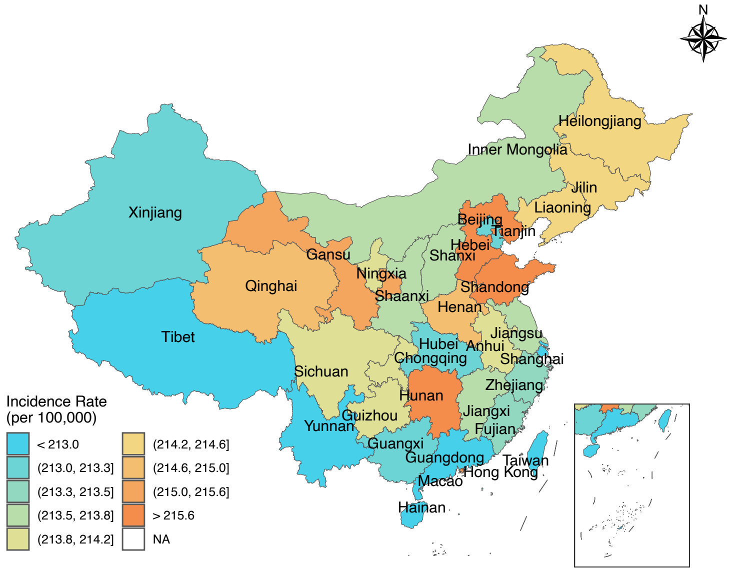


National Distribution of Prevalence Rate Attributed to
Dysthymia among Females and Males (Age−standardized), 2021

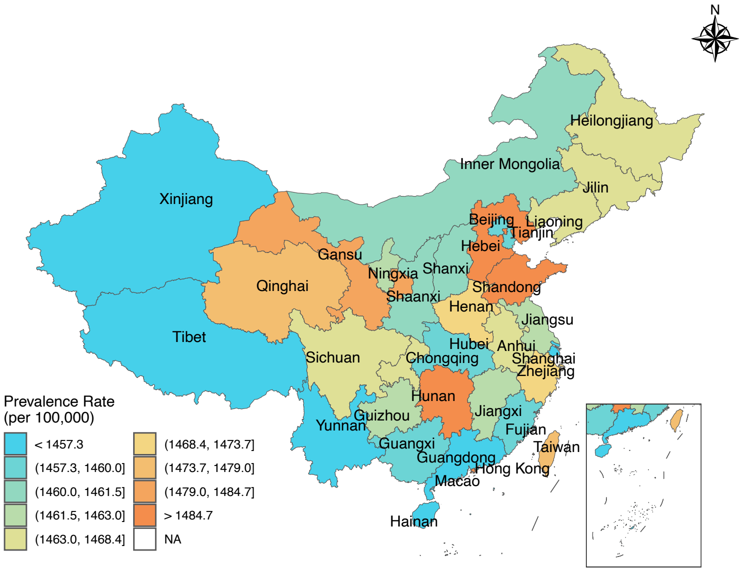

Figure S2 Spatial distribution of DALYs, YLDs, incidence, prevalence rate of anxiety disorders in China, 2021

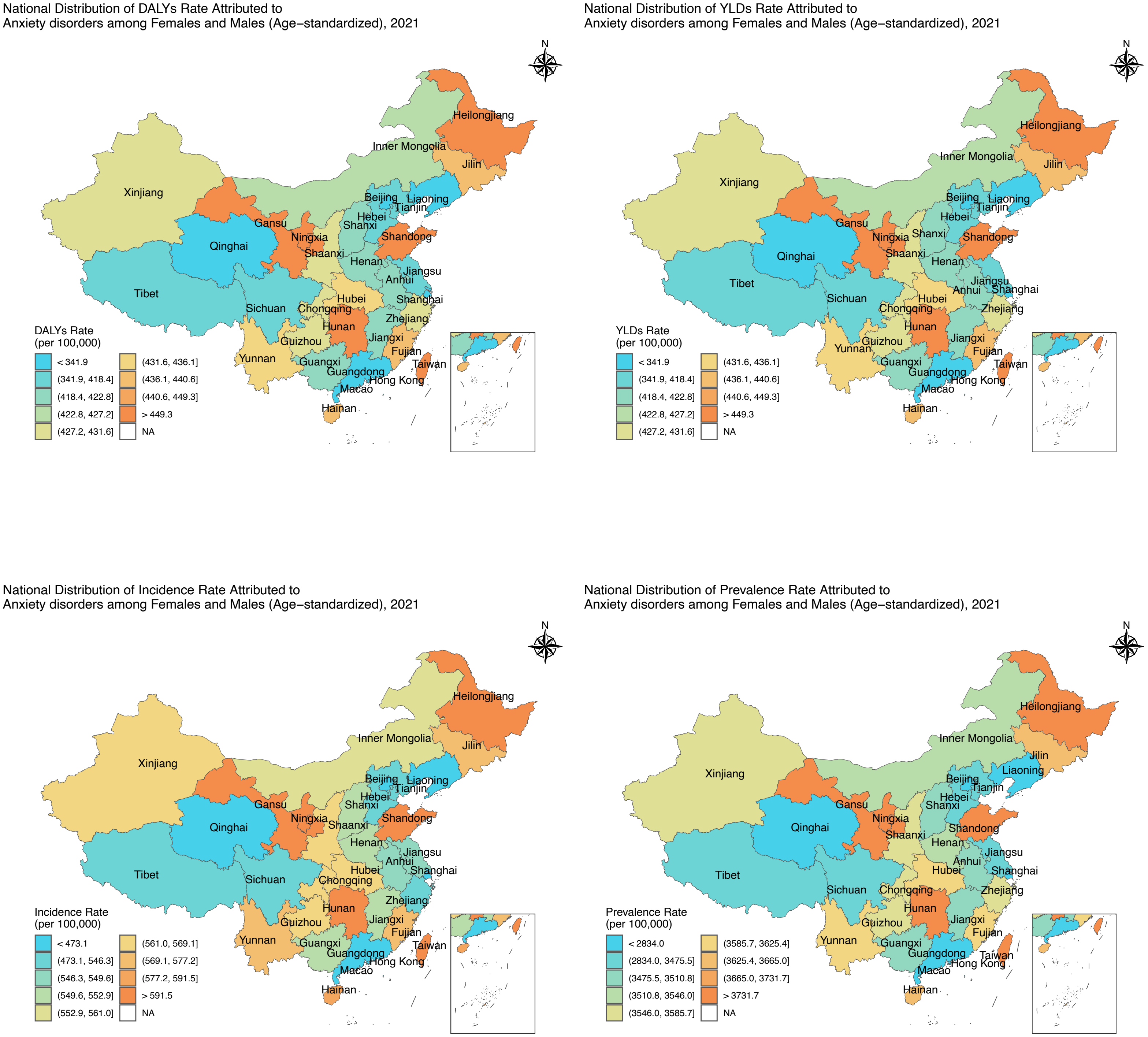

Figure S2 Spatial distribution of DALYs, YLDs, incidence, prevalence rate of schizophrenia in China, 2021

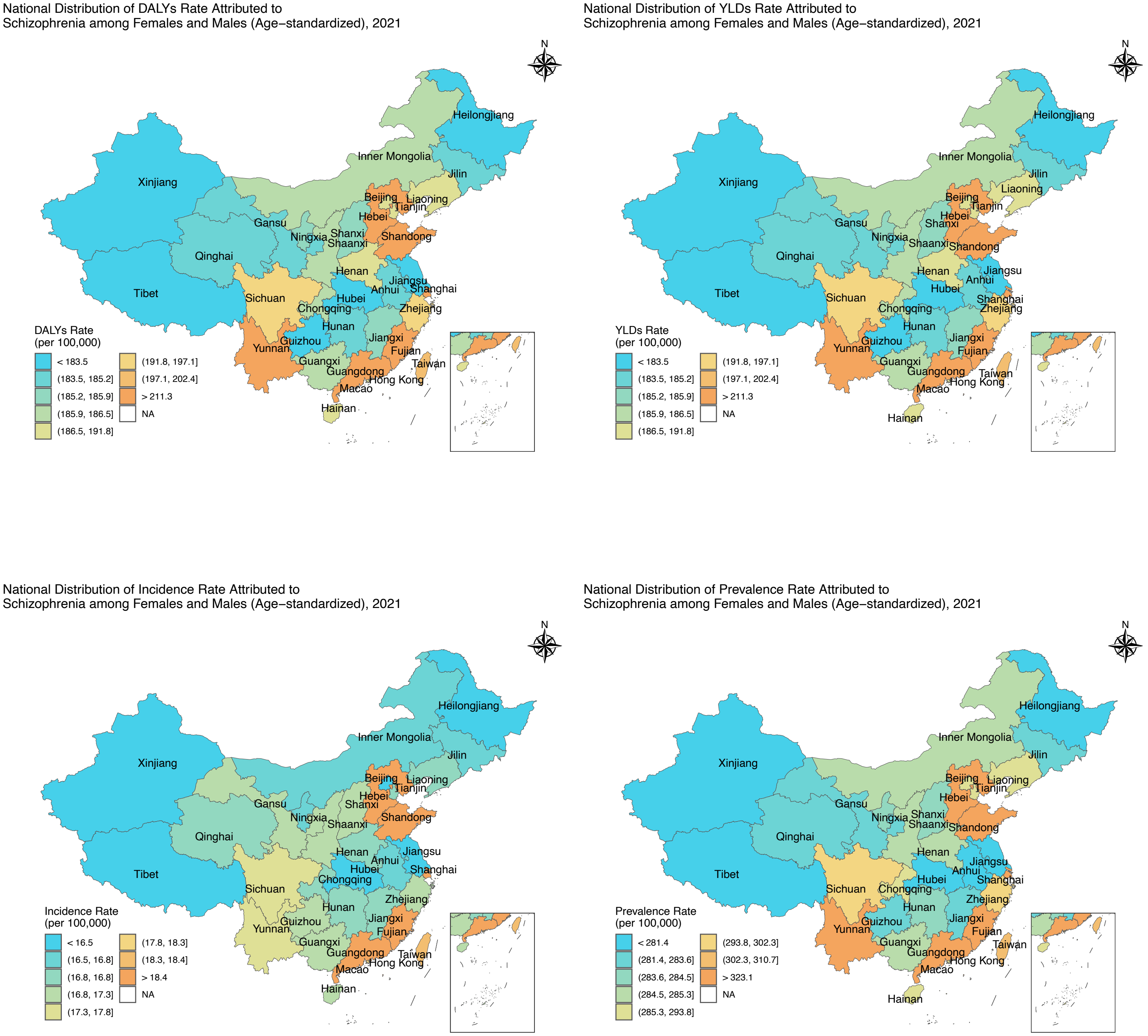

Figure S2 Spatial distribution of DALYs, YLDs, incidence, prevalence rate of bipolar disorder in China, 2021

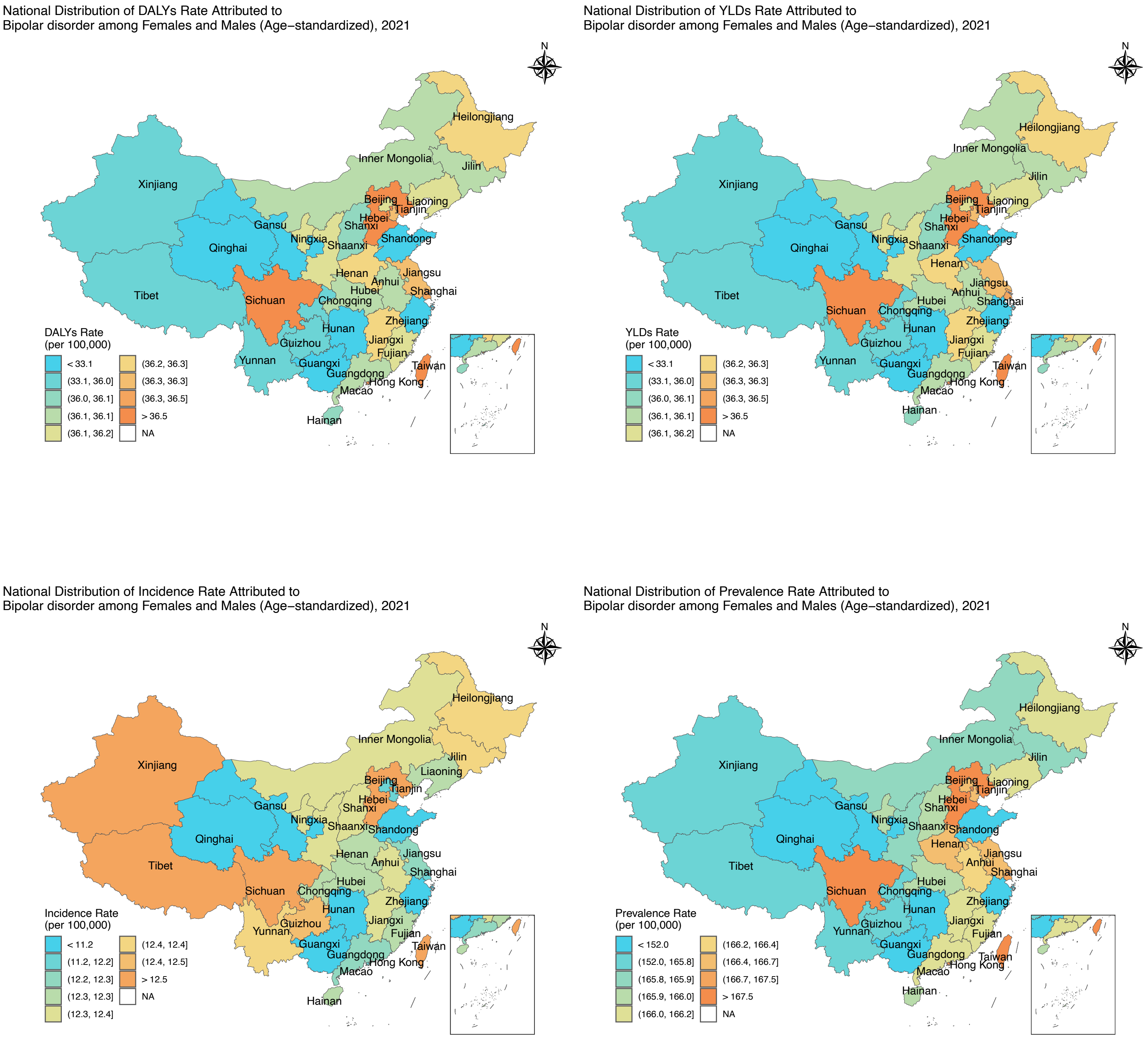

Figure S2 Spatial distribution of mortality, incidence, DALYs, YLDs, YLLs, prevalence rate of eating disorders in China, 2021

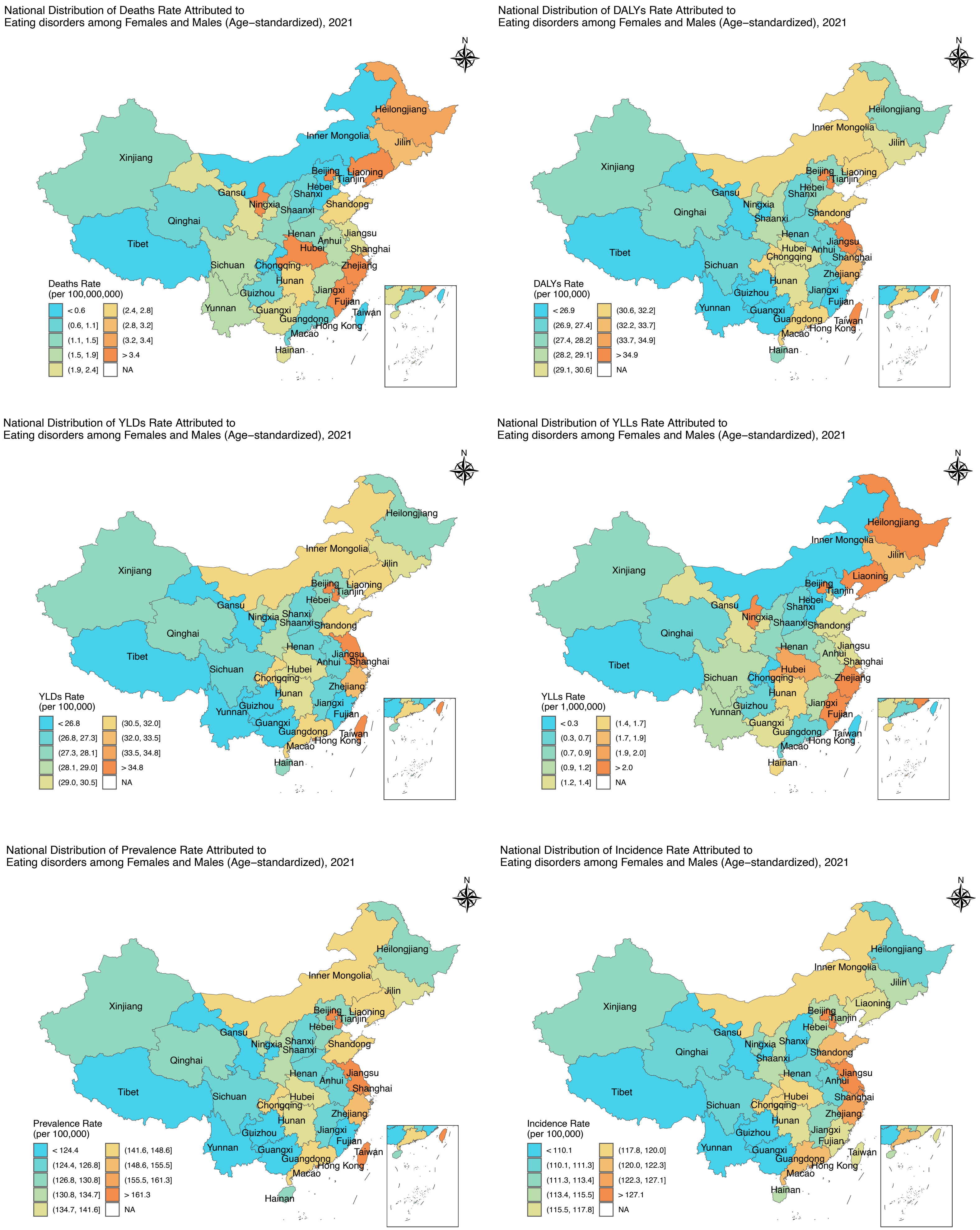

Figure S2 Spatial distribution of mortality, incidence, DALYs, YLDs, YLLs, prevalence rate of anorexia nervosa in China, 2021

National Distribution of Deaths Rate Attributed to
Anorexia nervosa among Females and Males (Age−standardized), 2021

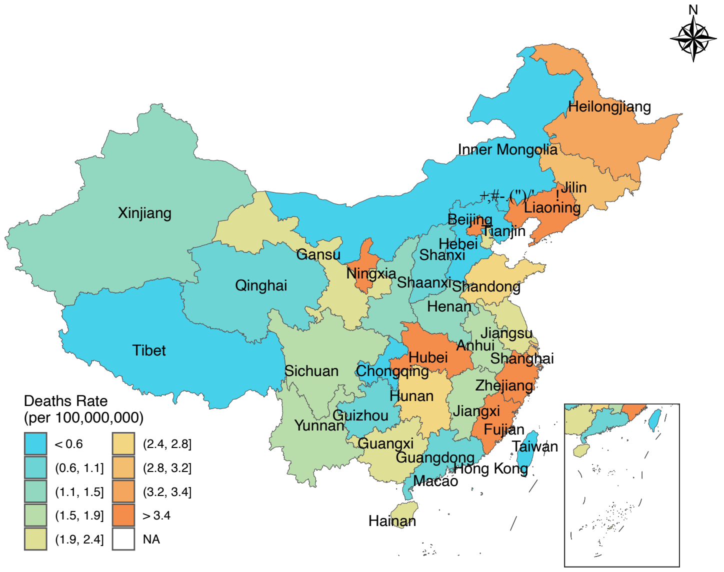


National Distribution of DALYs Rate Attributed to
Anorexia nervosa among Females and Males (Age−standardized), 2021

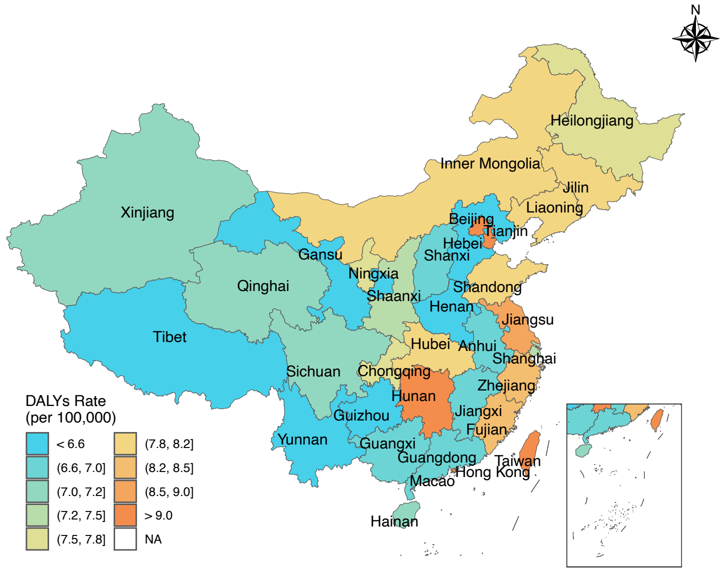


National Distribution of YLDs Rate Attributed to
Anorexia nervosa among Females and Males (Age−standardized), 2021

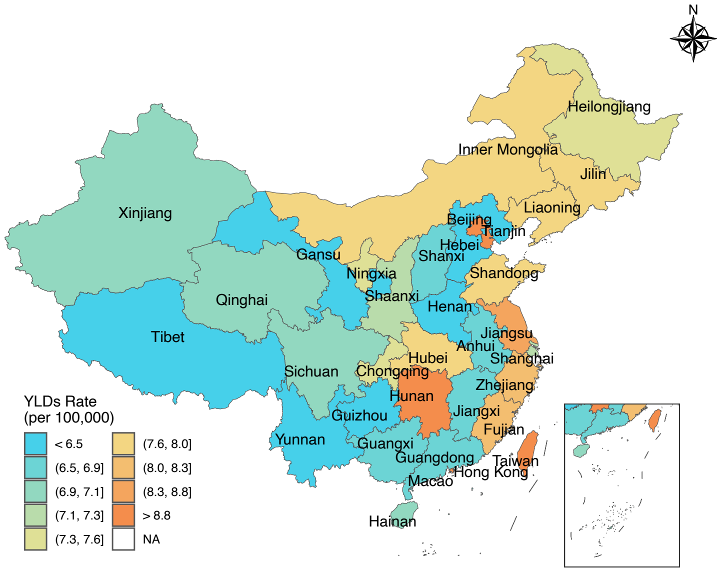


National Distribution of YLLs Rate Attributed to
Anorexia nervosa among Females and Males (Age−standardized), 2021

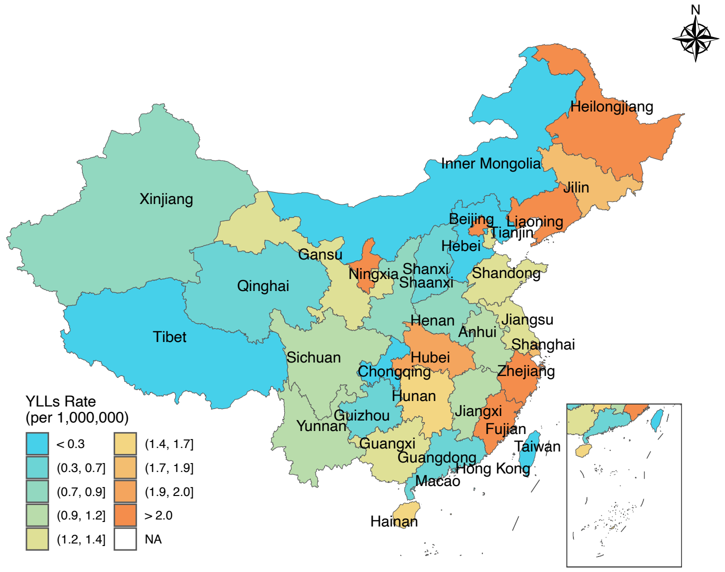


National Distribution of Prevalence Rate Attributed to
Anorexia nervosa among Females and Males (Age−standardized), 2021

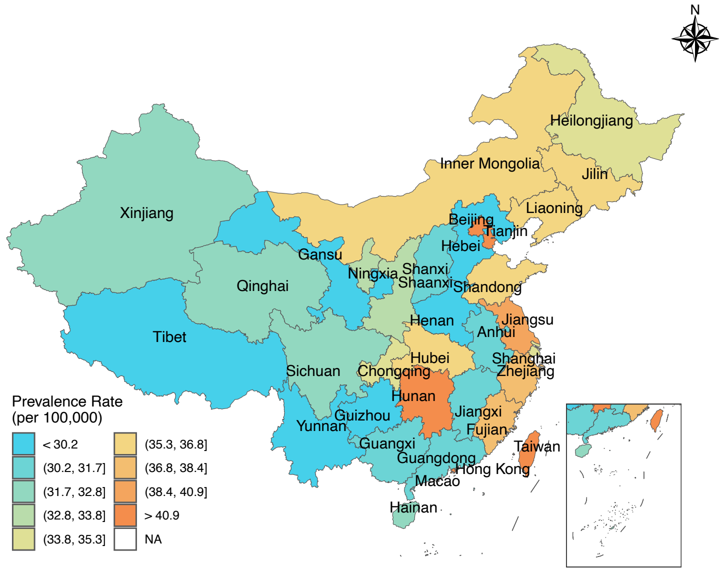


National Distribution of Incidence Rate Attributed to
Anorexia nervosa among Females and Males (Age−standardized), 2021

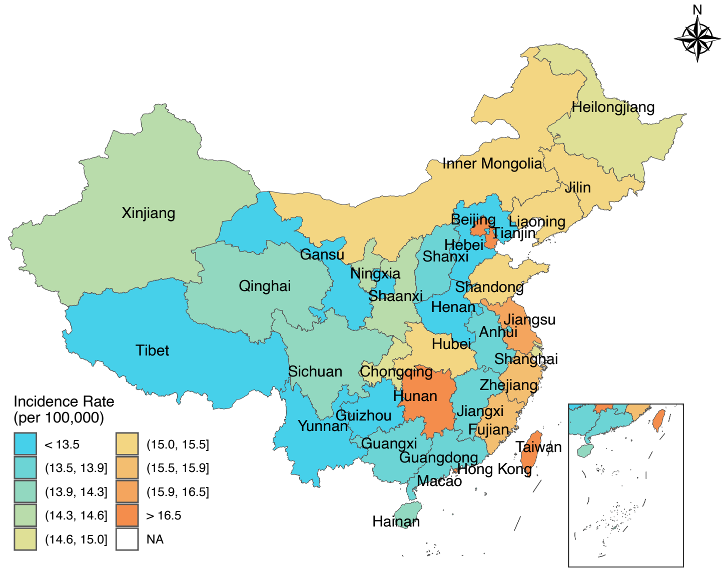

Figure S2 Spatial distribution of DALYs, YLDs, incidence, prevalence rate of bulimia nervosa in China, 2021

National Distribution of DALYs Rate Attributed to
Bulimia nervosa among Females and Males (Age−standardized), 2021

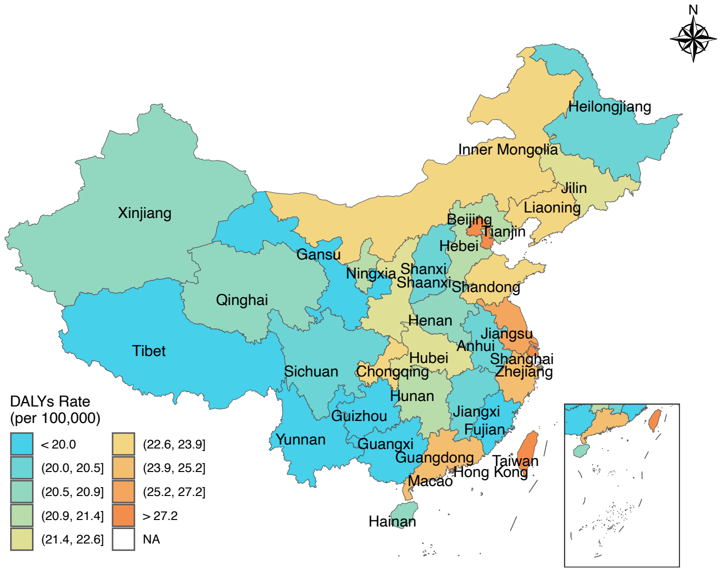


National Distribution of YLDs Rate Attributed to
Bulimia nervosa among Females and Males (Age−standardized), 2021

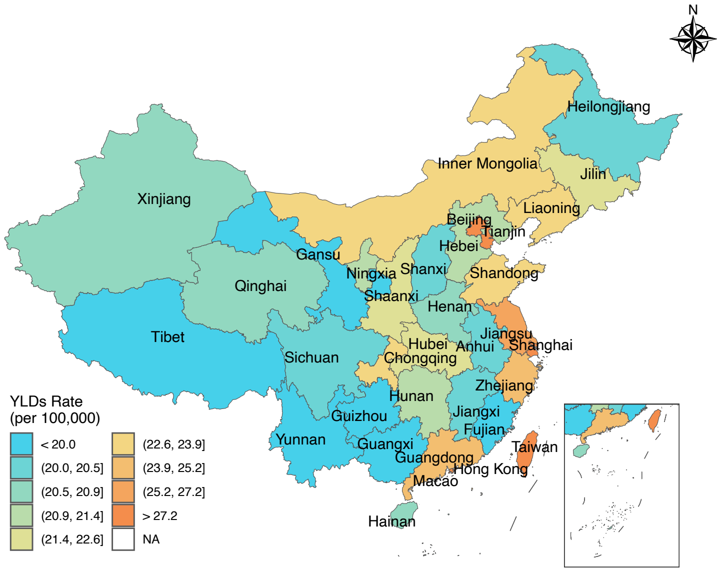


National Distribution of Incidence Rate Attributed to
Bulimia nervosa among Females and Males (Age−standardized), 2021

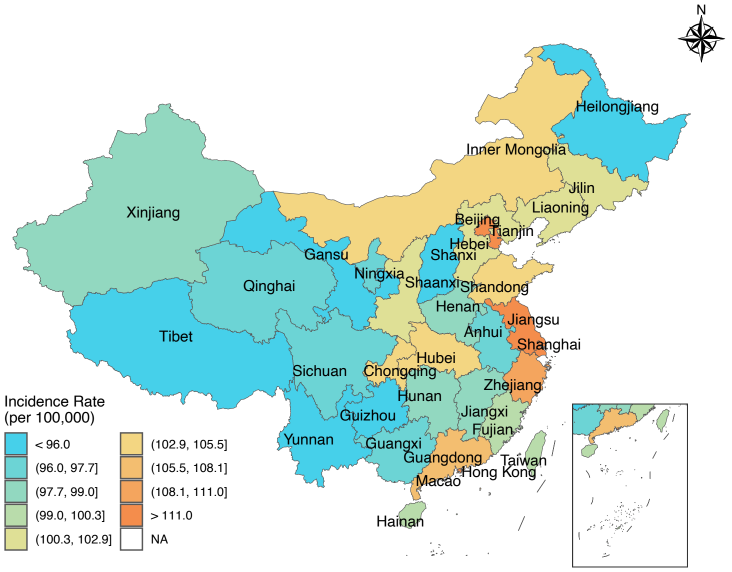


National Distribution of Prevalence Rate Attributed to
Bulimia nervosa among Females and Males (Age−standardized), 2021

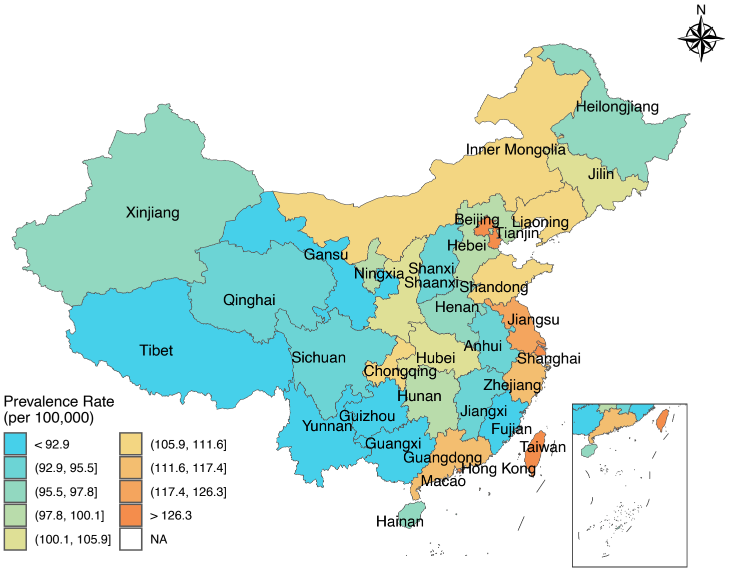

Figure S2 Spatial distribution of DALYs, YLDs, incidence, prevalence rate of autism spectrum disorders in China, 2021

National Distribution of DALYs Rate Attributed to
Autism spectrum disorders among Females and Males (Age−standardized), 2021

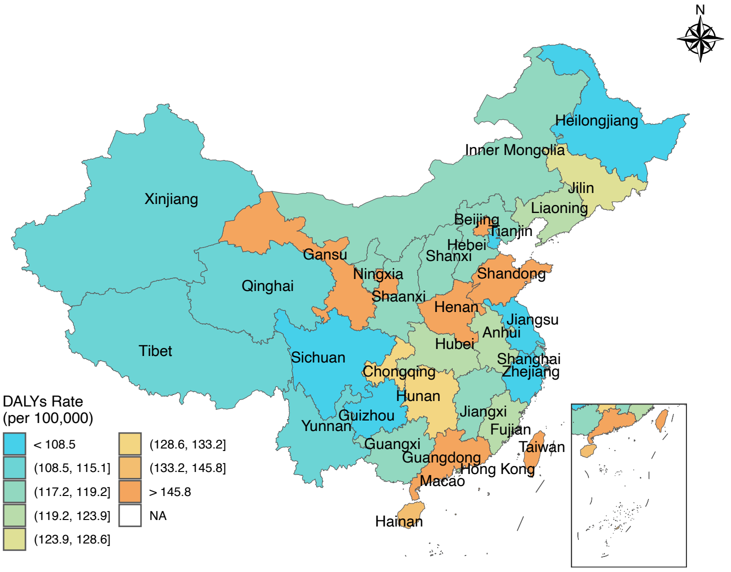


National Distribution of YLDs Rate Attributed to
Autism spectrum disorders among Females and Males (Age−standardized), 2021

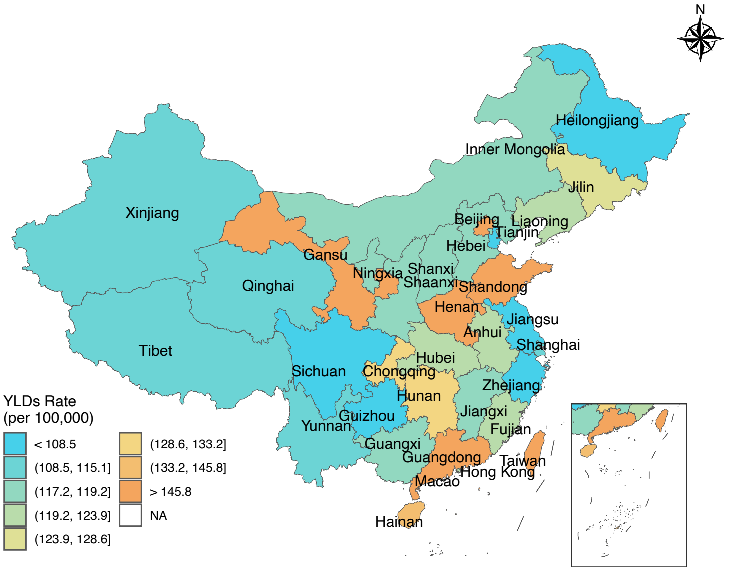


National Distribution of Incidence Rate Attributed to
Autism spectrum disorders among Females and Males (Age−standardized), 2021

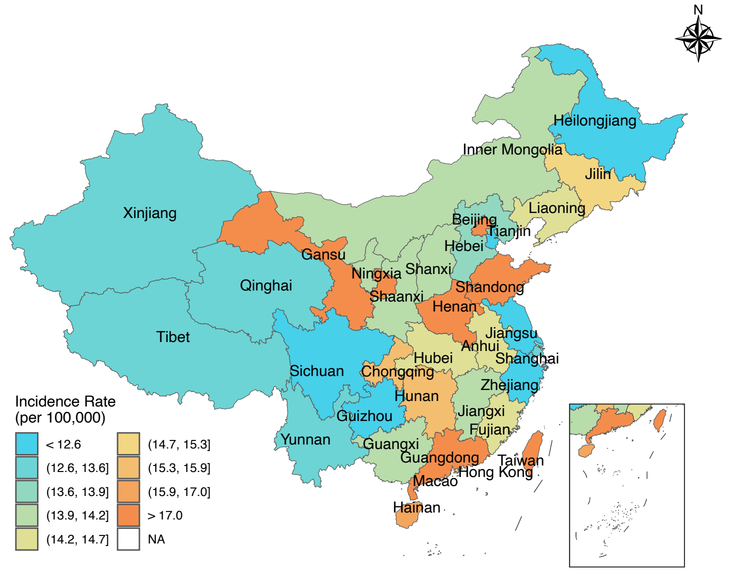


National Distribution of Prevalence Rate Attributed to
Autism spectrum disorders among Females and Males (Age−standardized), 2021

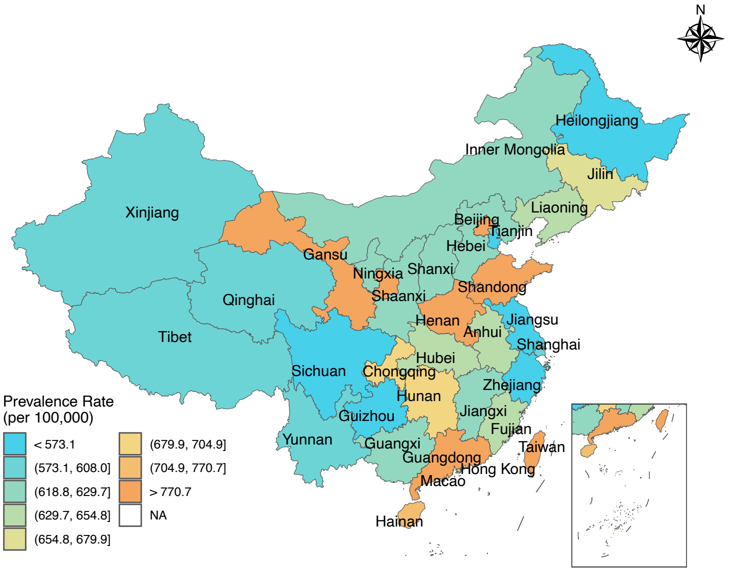

Figure S2 Spatial distribution of DALYs, YLDs, incidence, prevalence rate of attention deficit and hyperactivity in China, 2021

National Distribution of DALYs Rate Attributed to
Attention−deficit and hyperactivity disorder among Females and Males (Age−standardized), 2021

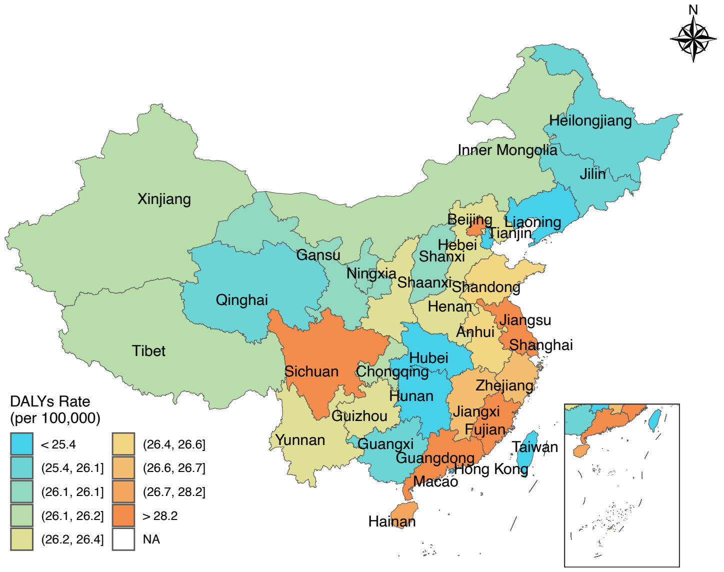


National Distribution of YLDs Rate Attributed to
Attention−deficit and hyperactivity disorder among Females and Males (Age−standardized), 2021

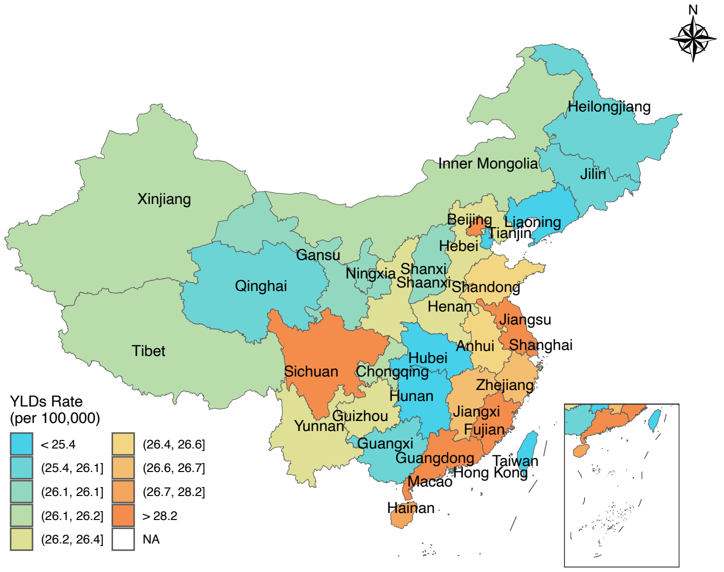


National Distribution of Incidence Rate Attributed to
Attention−deficit and hyperactivity disorder among Females and Males (Age−standardized), 2021

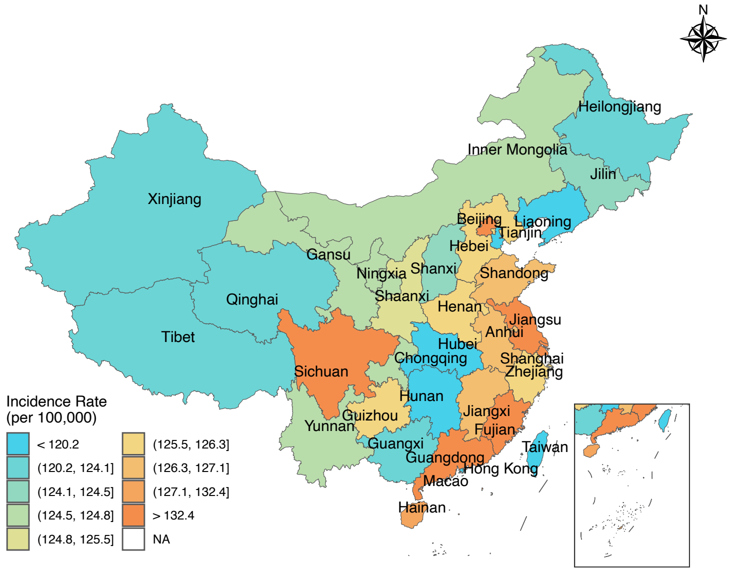


National Distribution of Prevalence Rate Attributed to
Attention−deficit and hyperactivity disorder among Females and Males (Age−standardized), 2021

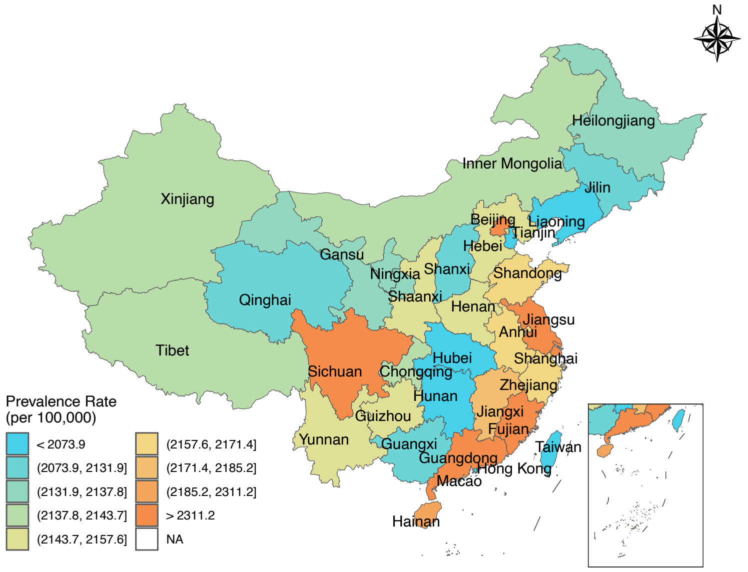

Figure S2 Spatial distribution of DALYs, YLDs, incidence, prevalence rate of conduct disorder in China, 2021

National Distribution of DALYs Rate Attributed to
Conduct disorder among Females and Males (Age−standardized), 2021

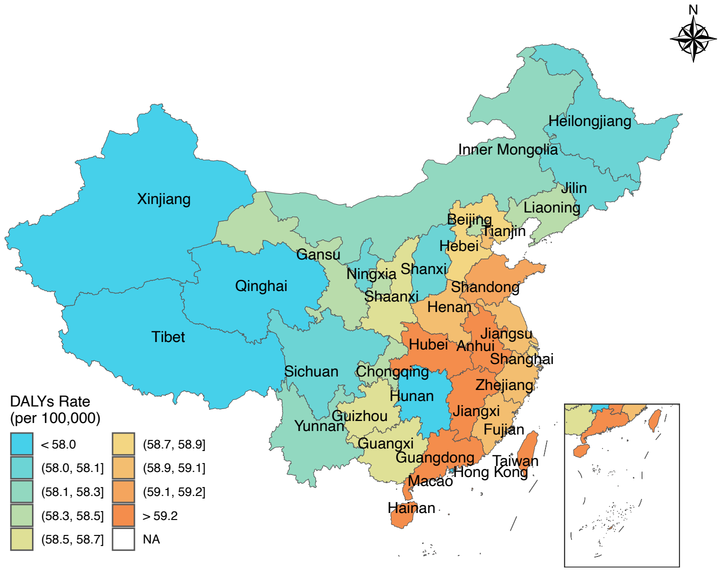


National Distribution of YLDs Rate Attributed to
Conduct disorder among Females and Males (Age−standardized), 2021

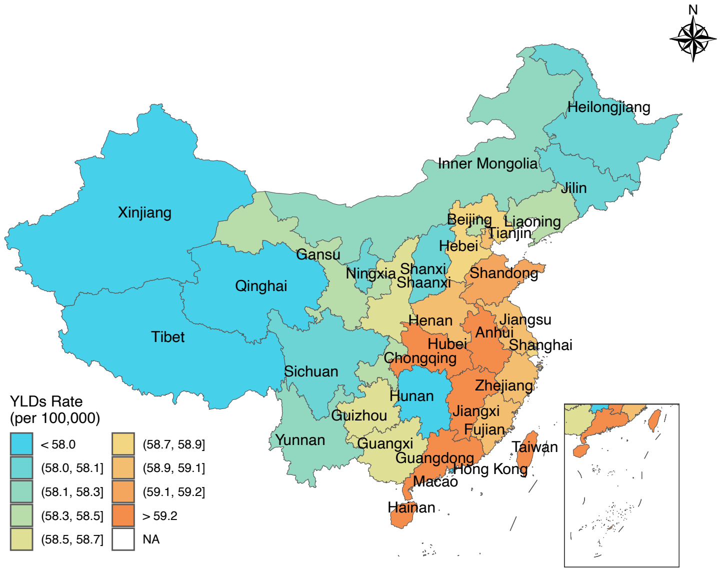


National Distribution of Incidence Rate Attributed to
Conduct disorder among Females and Males (Age−standardized), 2021

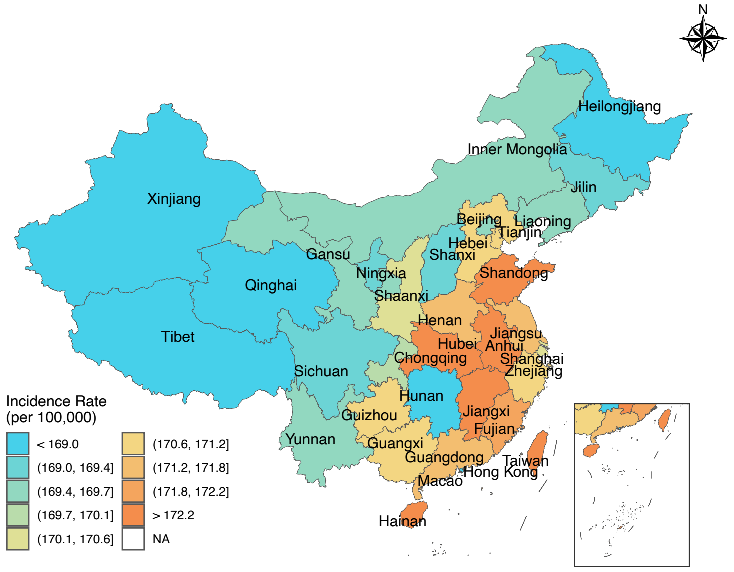


National Distribution of Prevalence Rate Attributed to
Conduct disorder among Females and Males (Age−standardized), 2021

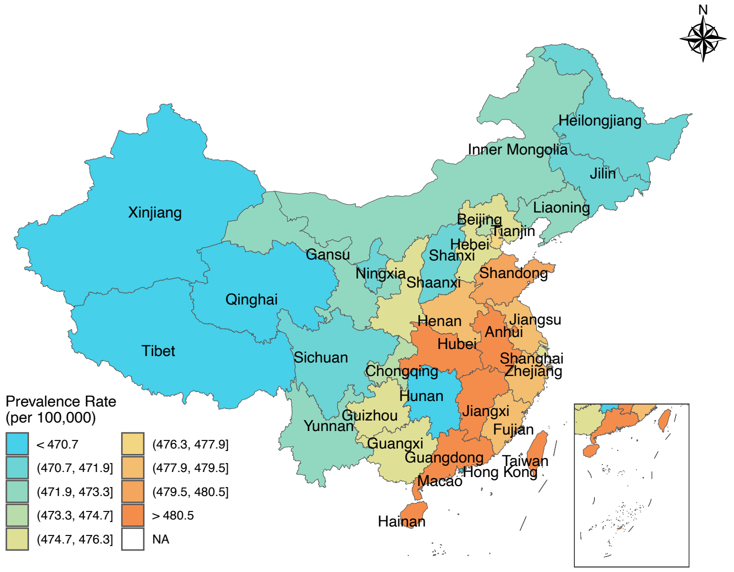

Figure S2 Spatial distribution of DALYs, YLDs, prevalence rate of idiopathic developmental intellectual disability in China, 2021

National Distribution of DALYs Rate Attributed to
Idiopathic developmental intellectual disability among Females and Males (Age−standardized), 2021

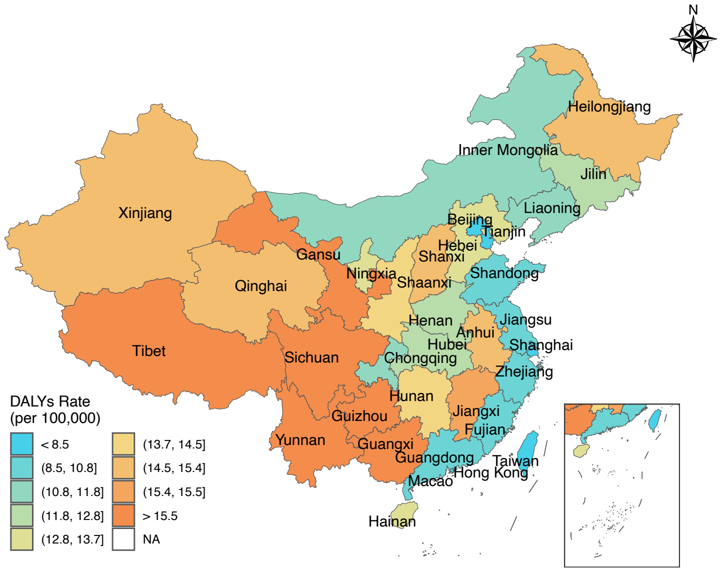


National Distribution of YLDs Rate Attributed to
Idiopathic developmental intellectual disability among Females and Males (Age−standardized), 202

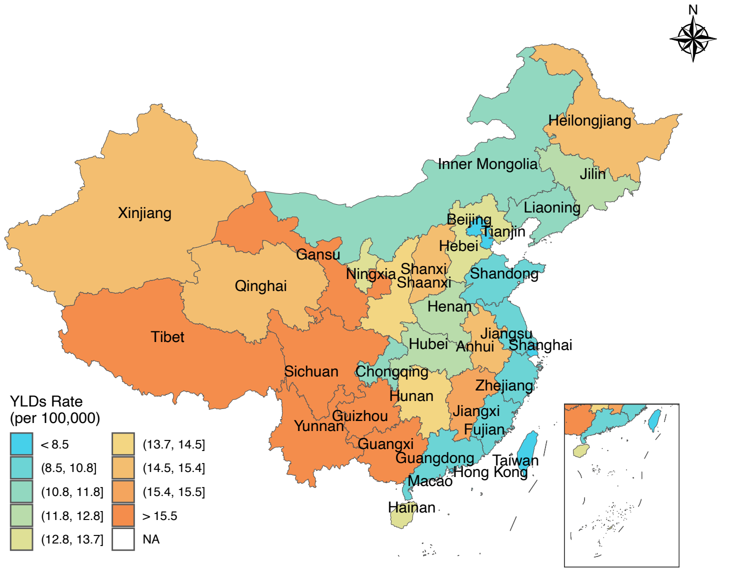


National Distribution of Prevalence Rate Attributed to
Idiopathic developmental intellectual disability among Females and Males (Age−standardized), 2021

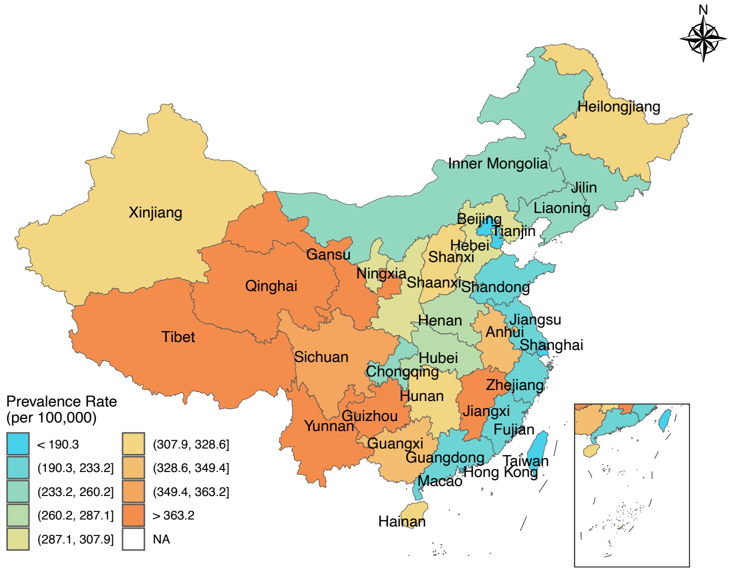

Figure S2 Spatial distribution of DALYs, YLDs, prevalence rate of other mental disorders in China, 2021

National Distribution of DALYs Rate Attributed to
Other mental disorders among Females and Males (Age−standardized), 2021

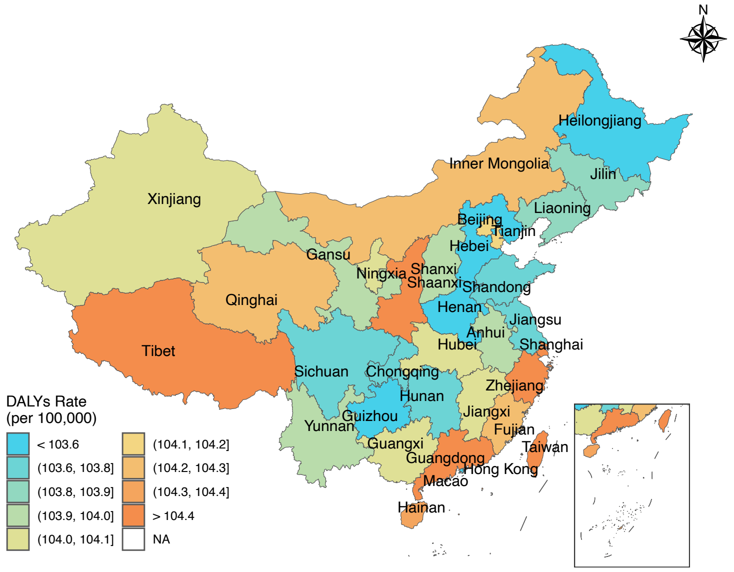


National Distribution of YLDs Rate Attributed to
Other mental disorders among Females and Males (Age−standardized), 2021

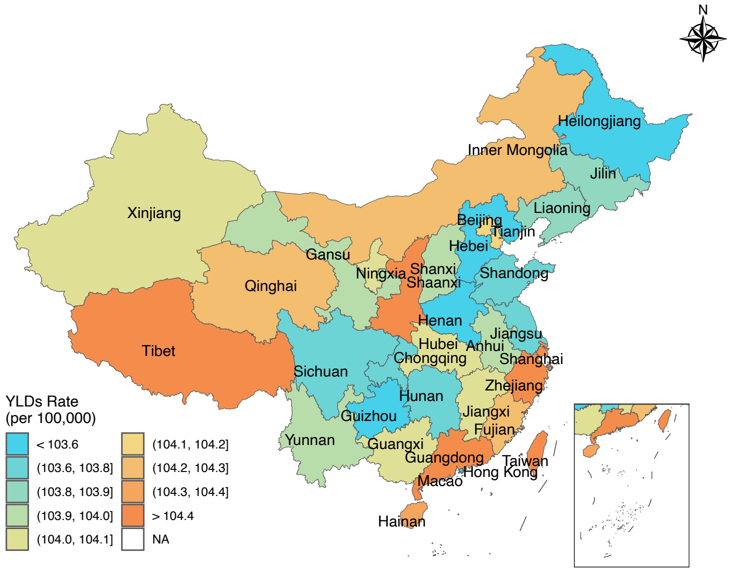


National Distribution of Prevalence Rate Attributed to
Other mental disorders among Females and Males (Age−standardized), 2021

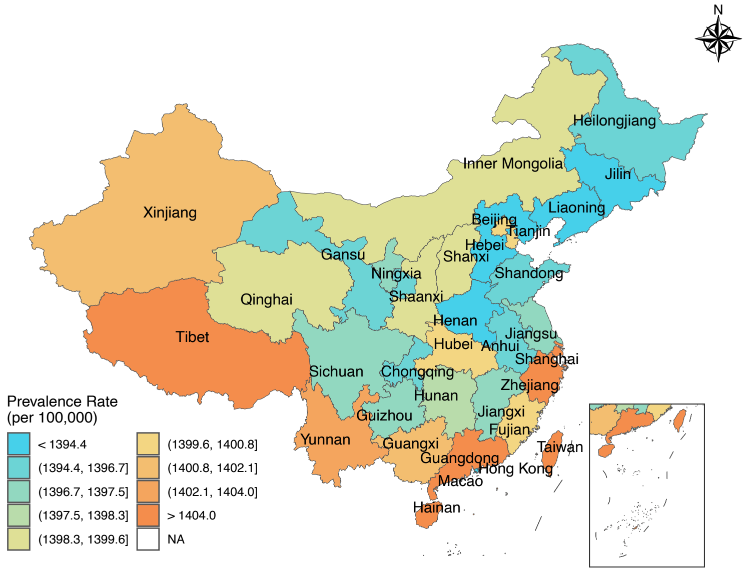

Supplement: S2 Fig — (PDF) [file pmen.0000146.s007.pdf]
